# Supplementary material for: A Vitamin B2‐Photocatalysed Approach to Methionine Analogues
Source: Angew Chem Int Ed Engl. 2022 Nov 10;61(50):e202212158. doi: 10.1002/anie.202212158 (PMC10100050; doi:10.1002/anie.202212158)
Supplement: Supplementary file 1 — Supporting Information [file ANIE-61-0-s001.pdf]

Angewandte Chemie
Eine Zeitschrift der Gesellschaft Deutscher Chemiker

Supporting Information

A Vitamin B_2-Photocatalysed Approach to Methionine Analogues

*O. J. Knowles , L. O. Johannissen, G. E. M. Crisenza, S. Hay, D. Leys, D. J. Procter**

Supplementary Data

Table of contents:

General Experimental 2
Pictures of Reaction Set-Up 3
Reaction Optimisation 4
Synthesis of Sulfides 9
Small Molecule Scope 10
Possible origin of diastereocontrol in couplings of substituted tetrahydrothiopyran substrates 32
Methionine Analogue Scope 33
Product Derivatisation 41
Diastereoselective Synthesis 43
Synthesis of Karady-Beckwith Alkene 43
Karady-Beckwith Example 47
Residue-Exchange Strategy 50
Peptide Starting Materials Synthesis 50
Residue Exchange Scope 60
N-Protecting group compatibility in dehydroamino acid substrates 68
Unsuccessful Examples 70
Mechanistic Studies 71
Deuterium labelling studies 71
Deuterium Control Experiment 74
UV/Vis Spectroscopy 75
Cyclic Voltammetry (CV) Studies 76
Stern-Volmer Quenching Studies 78
^{1}H- and ^{13}C-NMR spectra 81
Computational Analysis 146
References 153

General Experimental

All experiments were performed under an atmosphere of nitrogen, using anhydrous solvents, unless stated otherwise. All solvents and reagents were purchased from commercial sources and used as supplied. Photochemical reactions were subjected to irradiation from a 34W Kessil blue LED bulb, with the reaction tube placed approximately 4 cm from the bulb. ^{1}H NMR spectra were recorded on NMR spectrometers at 400 MHz and 500 MHz and ^{13}C NMR at 100 MHz and 125 MHz. ^{1}H NMR chemical shifts (δH) and ^{13}C NMR chemical shifts (δC) are quoted in parts per million (ppm) downfield from trimethylsilane (TMS) and coupling constants (J) are quoted in Hertz (Hz). Abbreviations for NMR data are s (singlet), d (doublet), t (triplet), q (quartet), m (multiplet). Infrared (IR) spectra were recorded as evaporated films or neat on a FTIR spectrometer and mass spectra were obtained using positive or negative electrospray ionisation (ESI), atmospheric pressure chemical ionization (APCI), or atmospheric solid analysis probe (ASAP) techniques. Column chromatography was carried out using silica gel 60 Angstrom (Å), 240-400 mesh. Thin layer chromatography (TLC) was performed on aluminium sheets pre-coated with silica gel, 0.20 mm (Macherey-Nagel, Polygram® Sil G/UV254). TLC plates were visualized by UV absorption, phosphomolybdic acid, vanillin or potassium permanganate solution and heating. Preparative TLC was performed on 20 × 20 cm glass plates 500 µm or 2000 µm thickness.

Pictures of Reaction Set-Up

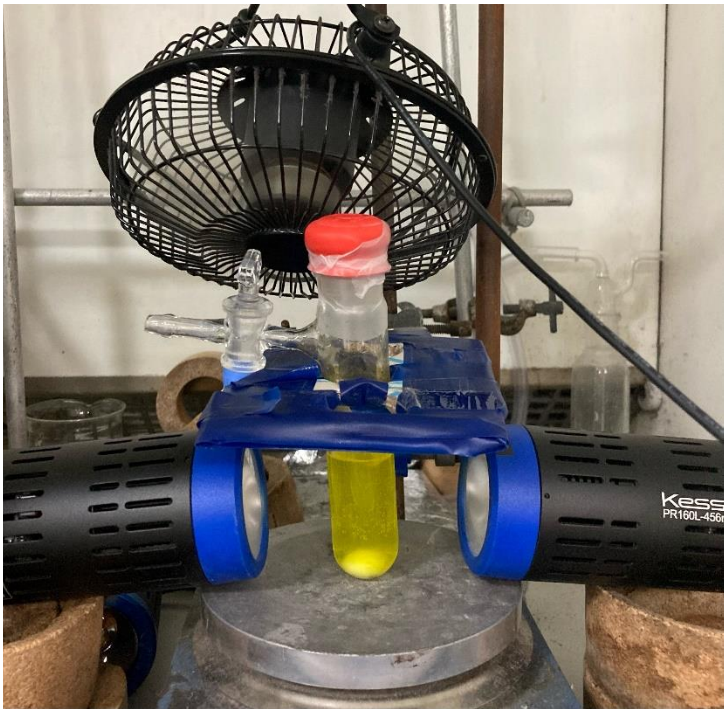

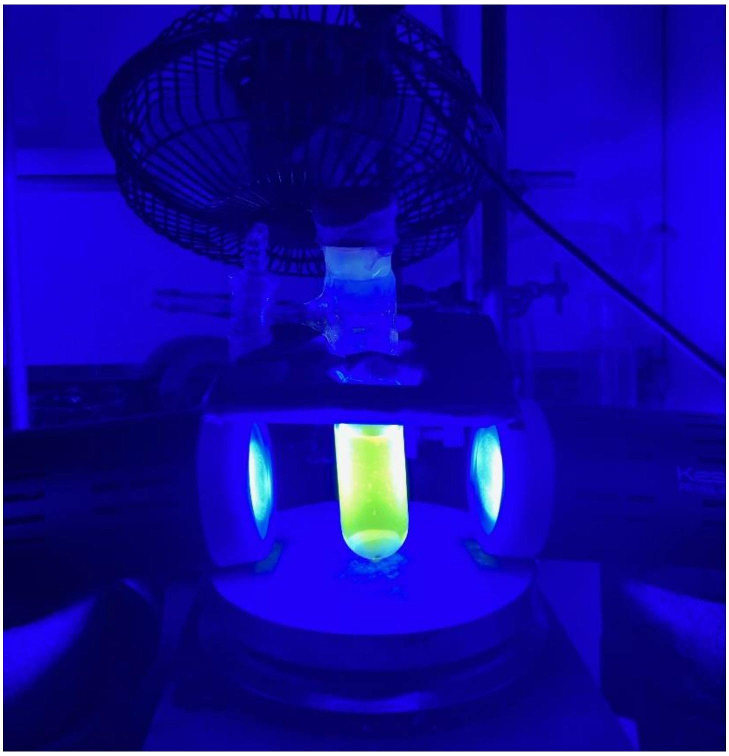

Reaction Optimisation

Solvent Screen

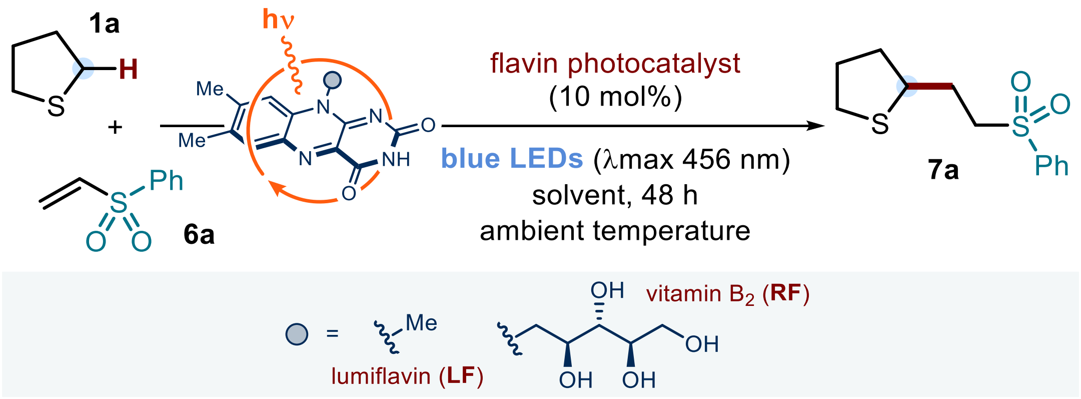

Entry	Solvent	Yield (%)
1	Water	12
2	MeOH	5
3	Acetone	0
4	DMF	<5
5	DMSO	<5
6	EtOAc	<5
7	Toluene	0
8	DCE	0
9[a]	Water	40
10[b]	Water	27
11	CH_2Cl_2	0

Reaction conditions: tetrahydrothiophene (3.0 equiv), phenyl vinyl sulfone (1.0 equiv.), riboflavin (10 mol%) in solvent (2.5 mL) with blue Kessel LED lamps. Reactions performed on a 0.05 mmol scale. NMR yield determined by ^{1}H-NMR spectroscopy using $MeNO_2$ as an internal standard. [a] Reaction concentration = 5 mM, solvent volume = 10 mL [b] Reaction concentration = 2.5 mM, solvent volume = 20 mL.

Cosolvent Screen

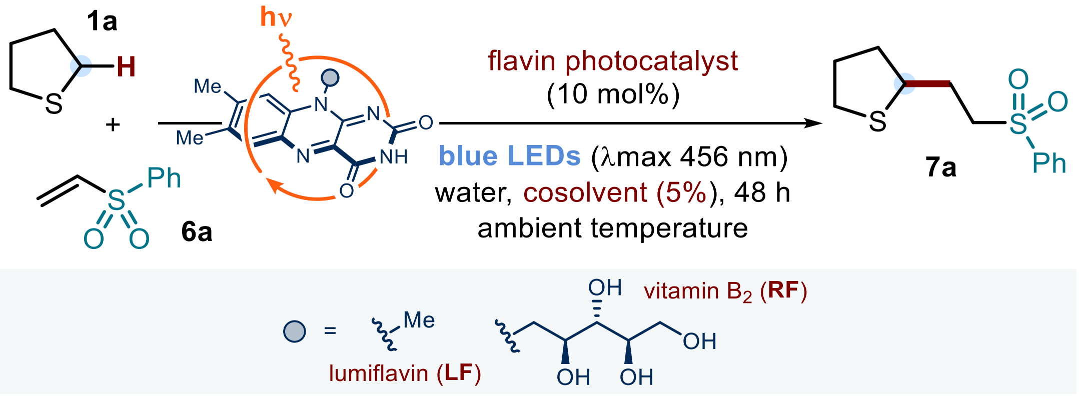

Entry	Cosolvent	Yield (%)
1	Methyl Acetate	49
2	Methyl Trifluoroacetate	0
3	Acetone	60
4	Sulfolane	48
5	1,4-dioxane	42
6	Trifluoroethanol	55
7	Ethanol	49
8	Ethyl Acetate	60
9	DMF	62

Reaction conditions: tetrahydrothiophene (3.0 equiv), phenyl vinyl sulfone (1.0 equiv.), riboflavin (10 mol%) in water (9.5 mL) and cosolvent (0.5 mL) with blue Kessel LED lamps. Reactions performed on a 0.05 mmol scale. NMR yield determined by ^{1}H-NMR spectroscopy using $MeNO_2$ as an internal standard.

HAT Donor Screen

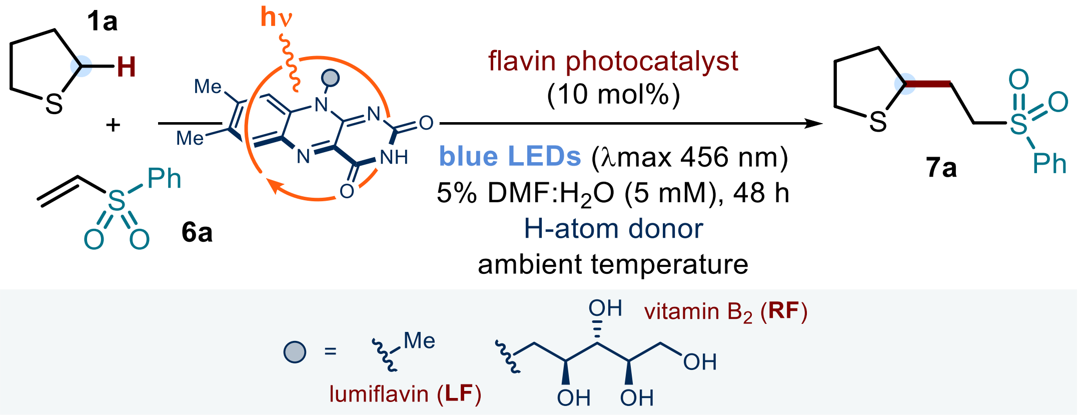

Entry	HAT donor	Yield (%)
1	None	62
2	Hantzsch ester	0
3	TTMSS	50
5	L-ascorbic acid	0
6	cyclohexa-1,4-diene	0

Reaction conditions: tetrahydrothiophene (3.0 equiv), phenyl vinyl sulfone (1.0 equiv.), riboflavin (10 mol%) in water (9.5 mL) and DMF (0.5 mL) with blue Kessel LED lamps. Reactions performed on a 0.05 mmol scale. NMR yield determined by ^{1}H-NMR spectroscopy using $MeNO_2$ as an internal standard.

Stoichiometry Screen

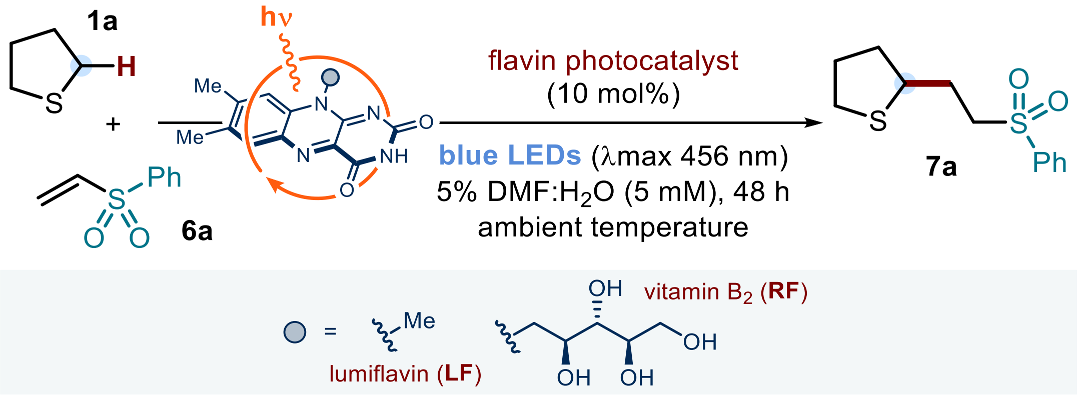

Entry	Sulfide equiv.	Alkene equiv.	Yield (%)
1	1	1	32
2	2	1	53
3	3	1	62
4	5	1	60
5	10	1	73
6	1	3	24
7	1	10	46

Reaction conditions: tetrahydrothiophene (X equiv), phenyl vinyl sulfone (X equiv.), riboflavin (10 mol%) in water (9.5 mL) and DMF (0.5 mL) with blue Kessel LED lamps. Reactions performed on a 0.05 mmol scale. NMR yield determined by ^{1}H-NMR spectroscopy using $MeNO_2$ as an internal standard.

Photocatalyst Screen

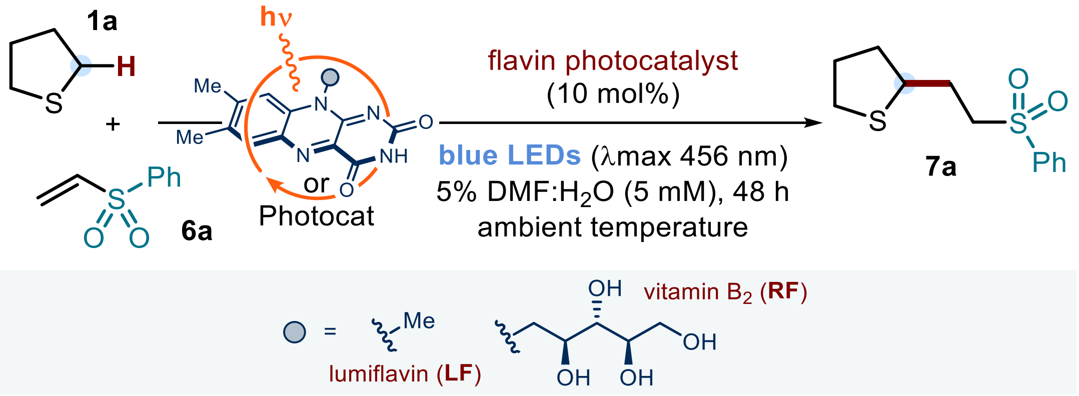

Entry	Photocatalyst	Yield (%)
1	Riboflavin	(76)
2	Lumiflavin	65
3	Riboflavin Tetracetate	50
4	Eosin Y	0
5	Rhodamine B	0

Reaction conditions: tetrahydrothiophene (10.0 equiv), phenyl vinyl sulfone (1.0 equiv.), photocatalyst (10 mol%) in water (9.5 mL) and DMF (0.5 mL) with blue Kessel LED lamps. Reactions performed on a 0.05 mmol scale. NMR yield determined by ^{1}H-NMR spectroscopy using $MeNO_2$ as an internal standard. Isolated yields in parentheses.

Synthesis of Sulfides

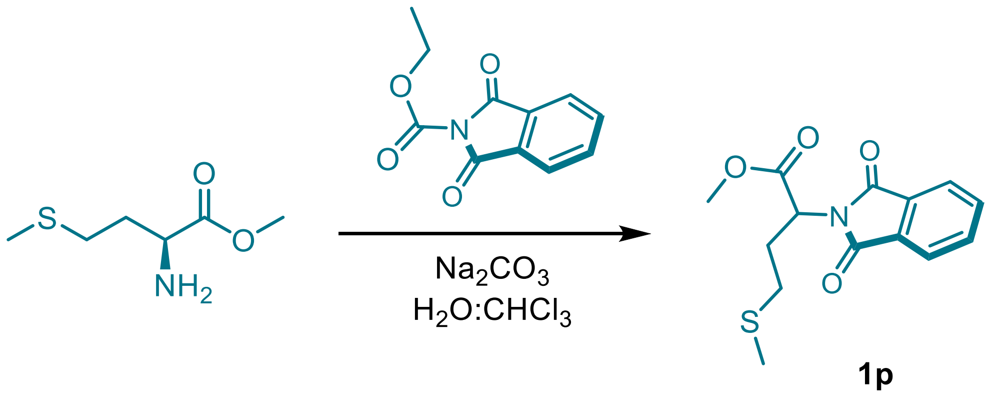

1p

Methyl (S)-2-(1,3-dioxoisoindolin-2-yl)-4-(methylthio)butanoate (1p)

L-Methionine methyl ester (1 g, 5.00 mmol), N-carbethoxyphthalimide (1.10 g, 5.00 mmol) and Na_2CO_3 (0.529 g, 5.00 mmol) were added to a 1:1 mixture of water and chloroform (10 mL) and stirred at room temperature for 2 hours. Upon consumption of starting materials (as determined by TLC), the reaction mixture was acidified with 1 M HCL, extracted with chloroform, dried over $MgSO_4$ and evaporated under reduced pressure to yield a white residue. The crude product was purified by column chromatography (30% EtOAc / Hexane) to yield a white solid (805 mg, 2.75 mmol, 55%). **^{1}H NMR** (500 MHz, $CDCl_3$) δ 2.08 (s, 3H, SCH_3), 2.42 – 2.61 (m, 4H, SCH_2 + SCH_2CH_2), 3.74 (s, 3H, OCH_3), 5.10 (dd, J = 8.3, 5.9 Hz, 1H, NC*H*), 7.72 – 7.79 (m, 2H, Ar C*H*), 7.84 – 7.91 (m, 2H, Ar C*H*). **^{13}C NMR** (126 MHz, $CDCl_3$) δ 15.40 (SCH_3), 28.1 (SCH_2CH_2), 30.8 (SCH_2), 50.8 (NCH), 52.9 (OCH_3), 123.7 (Ar *C*H), 131.8 (Ar *C*), 134.3 (Ar *C*H), 167.7 (*C*O), 169.6 (*C*O). **HRMS** $C_{14}H_{15}O_4SNa$ ($M+Na^+$) predicted 316.0614, found 316.1005.

The data are in accordance with the literature.[1]

Small Molecule Scope

General Method A: C-H type alpha alkylation of sulfides with alkenes:

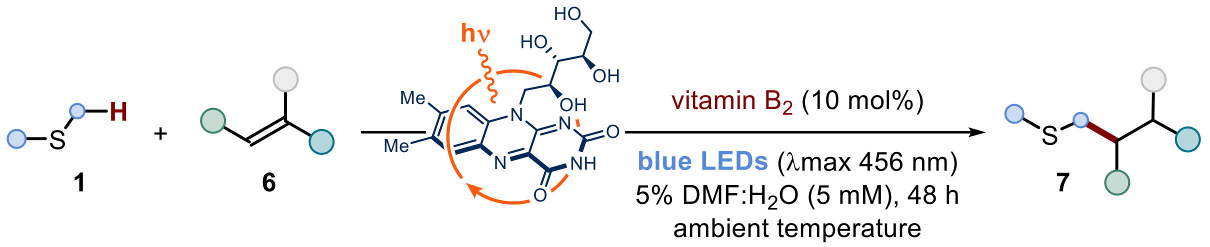

A 40 mL oven-dried Schlenk tube with a magnetic stirrer bar was charged with riboflavin (3.7 mg, 0.01 mmol, 0.1 equiv.), alkene (0.1 mmol, 1 equiv.) and sulfide (1 mmol, 10 equiv.) and placed under a nitrogen atmosphere. The reagents were dissolved in degassed water (19 mL) and dry DMF (1 mL), and irradiated with blue LEDs (453 nm) for 48 h. After completion of the reaction, the reaction mixture was extracted withCH_2Cl_2 (3 × 50 mL), washed with 10% LiCl solution (1 × 50 mL), dried over $MgSO_4$ and evaporated under reduced pressure. The crude residue was purified by chromatography.

Sulfide Scope

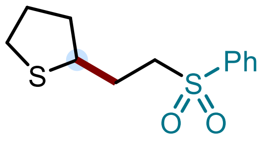

7a

2-(2-(phenylsulfonyl)ethyl)tetrahydrothiophene (7a)

7a was prepared according to general method a, using phenyl vinyl sulfone (16.8 mg, 0.1 mmol) and tetrahydrothiophene (88.1 mg, 1 mmol). The crude product was purified using preparative TLC (30% EtOAc: Hexane) to give a colourless oil (19.5 mg, 0.076 mmol, 76%). **^{1}H NMR** (500 MHz, $CDCl_3$) δ 1.53 – 1.63 (m, 1H, $SCH_2CH_2CH_aH_b$), 1.81 – 1.96 (m, 2H, $SO_2CH_2CH_aH_b$, $SCH_2CH_aH_b$), 1.97 – 2.15 (m, 3H, $SO_2CH_2CH_aH_b$, $SCH_2CH_aH_b$, $SCH_2CH_2CH_aH_b$), 2.78 – 2.89 (m, 2H, SCH_2), 3.12 (ddd, J = 13.8, 11.2, 4.9 Hz, 1H, $SO_2CH_aH_b$), 3.22 (ddd, J = 13.9, 11.2, 5.0 Hz, 1H, $SO_2CH_aH_b$), 3.36 (dq, J = 9.0, 6.2 Hz, 1H, SC*H*), 7.57 (t, J = 7.8 Hz, 2H, Ar *H*), 7.63 – 7.69 (m, 1H, Ar *H*), 7.87 – 7.93 (m, 2H, Ar *H*). **^{13}C NMR** (126 MHz, $CDCl_3$) δ 30.1 (SCH_2CH_2), 30.2 ($SO_2CH_2CH_2$), 32.4 (SCH_2), 37.1 ($SCH_2CH_2CH_2$), 47.2 (SCH), 55.4 (SO_2CH_2), 128.2 (Ar *C*H), 129.4 (Ar *C*H), 133.8 (Ar *C*H), 139.1 (Ar *C*). **$ν_{max}$** (thin film/cm^{-1}):

2984, 1147, 1372, 938, 847, 787, 634, 608. **HRMS** $C_{12}H_{17}O_2S_2$ (M+H$^+$) Expected 257.0664, Found 257.0655.

The data are in accordance with the literature.[2]

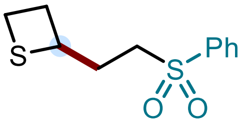

7b

2-(2-(phenylsulfonyl)ethyl)thietane (7b)

7b was prepared according to general method a, using phenyl vinyl sulfone (16.8 mg, 0.1 mmol) and thietane (74.1 mg, 1 mmol). The crude product was purified using column chromatography (30% EtOAc: Hexane) to give an off-white oil (11.1 mg, 0.046 mmol, 46%). **^{1}H NMR** (400 MHz, $CDCl_3$) δ 2.05 – 2.26 (m, 2H, $SO_2CH_2CH_2$), 2.48 – 2.61 (m, 1H, $SCH_2CH_aH_b$), 2.91 – 3.24 (m, 5H, SCH_2, $SCH_2CH_aH_b$, SO_2CH_2), 3.64 – 3.79 (m, 1H, SC*H*), 7.54 – 7.60 (m, 2H, Ar C*H*).), 7.61 – 7.71 (m, 1H, Ar C*H*).), 7.89 – 7.95 (m, 2H, Ar C*H*). **^{13}C NMR** (101 MHz, $CDCl_3$) δ 21.6 (SCH_2), 31.6 ($SO_2CH_2CH_2$), 32.8 (SCH_2CH_2), 39.9 (S*C*H), 53.4 (SO_2CH_2), 128.1 (Ar C*H*), 129.4 (Ar C*H*), 133.8 (Ar C*H*), 139.1 (Ar *C*). **v_{max}** (thin film/cm^{-1}): 3053, 1422, 1265, 1155, 896, 744, 706. **HRMS** $C_{11}H_{15}O_2S_2$ (M+H$^+$) Expected 243.0508, Found 243.507.

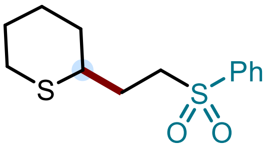

7c

(2-(2-(phenylsulfonyl)ethyl)tetrahydro-2H-thiopyran (7c)

7c was prepared according to general method a, using phenyl vinyl sulfone (16.8 mg, 0.1 mmol) and tetrahydrothiopyran (102 mg, 101 µL, 1 mmol). The crude product was purified using preparative TLC (30% EtOAc: Hexane) to give an off-white oil (17.2 mg, 0.064 mmol, 64%). **^{1}H NMR** (500 MHz, $CDCl_3$) δ 1.23 – 1.49 (m, 2H, $SCH_2CH_2CH_aH_bCH_aH_b$), 1.50 – 1.65 (m, 1H, $SCH_2CH_aH_b$), 1.75 – 2.05 (m, 5H, $SO_2CH_2CH_2$, $SCH_2CH_aH_bCH_aH_bCH_aHb$), 2.51 – 2.62 (m, 2H, SCH_2), 2.71 (tdd, *J* = 9.6, 4.8, 2.7 Hz, 1H,

SCH), 3.16 (ddd, J = 14.0, 10.9, 5.2 Hz, 1H, $SO_2CH_aH_b$), 3.29 (ddd, J = 14.0, 11.0, 5.1 Hz, 1H, $SO_2CH_aH_b$), 7.57 (dd, J = 8.5, 7.1 Hz, 2H, Ar H), 7.62 – 7.70 (m, 1H, Ar H), 7.88 – 7.95 (m, 2H, Ar H). **^{13}C NMR** (126 MHz, $CDCl_3$) δ 25.5 ($SCH_2CH_2CH_2CH_2$), 27.0 ($SCH_2CH_2CH_2$), 28.5 (SCH_2, $SO_2CH_2CH_2$), 34.3 (SCH_2CH_2), 41.0 (SCH), 53.9 (SO_2CH_2), 128.1 (Ar CH), 129.3 (Ar CH), 133.8 (Ar CH), 139.1 (Ar C). **ν_{max}** (thin film/cm^{-1}): 3054, 1422, 1264, 1151, 896, 737, 706. **HRMS** $C_{13}H_{19}O_2S_2$ (M+H$^+$) Expected 271.0821, Found 271.0834.

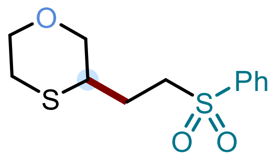

7d

3-(2-(phenylsulfonyl)ethyl)-1,4-oxathiane (7d)

7d was prepared according to general method a, using phenyl vinyl sulfone (16.8 mg, 0.1 mmol) and 1,4-oxathiane (104 mg, 93.5 μL, 1 mmol). The crude product was purified using preparative TLC (30% EtOAc: Hexane) to give an off-white oil (11.4 mg, 0.042 mmol, 42%). **^{1}H NMR** (500 MHz, $CDCl_3$) δ 1.92 – 2.07 (m, 2H, $SO_2CH_2CH_2$), 2.52 – 2.63 (m, 2H, SCH_2), 2.71 – 2.80 (m, 1H, SCH), 3.17 (ddd, J = 14.0, 9.9, 6.4 Hz, 1H, $SO_2CH_aH_b$), 3.33 (ddd, J = 14.0, 9.9, 5.9 Hz, 1H, $SO_2CH_aH_b$), 3.55 (dd, J = 11.7, 6.9 Hz, 1H, $SCHCH_aH_bO$), 3.77 (ddd, J = 11.7, 6.2, 4.2 Hz, 1H, $SCH_2CH_aH_bO$), 3.90 – 3.97 (m, 1H, $SCH_2CH_aH_bO$), 3.99 (dd, J = 11.8, 2.8 Hz, 1H, $SCHCH_aH_bO$), 7.55 – 7.63 (m, 2H, Ar H), 7.63 – 7.71 (m, 1H, Ar H), 7.90 – 7.95 (m, 2H, Ar H). **^{13}C NMR** (126 MHz, $CDCl_3$) δ 24.5 ($SO_2CH_2CH_2$), 25.4 (SCH_2), 37.7 (SCH), 53.8 (SO_2CH_2), 68.5 (SCH_2CH_2O), 73.2 ($SCHCH_2O$), 128.1 (Ar CH), 129.4 (Ar CH), 133.9 (Ar CH), 139.0 (Ar C). **ν_{max}** (thin film/cm^{-1}): 3053, 1421, 1264, 1151, 896, 755, 705. **HRMS** $C_{12}H_{17}O_3S_2$ (M+H$^+$) Expected 273.0614, Found 273.0600.

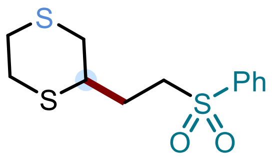

7e

2-(2-(phenylsulfonyl)ethyl)-1,4-dithiane (7e)

7e was prepared according to general method a, using phenyl vinyl sulfone (16.8 mg, 0.1 mmol) and 1,4-dithiane (120 mg, 1 mmol). The crude product was purified using preparative TLC (chloroform) to give an off-white oil (11.5 mg, 0.04 mmol, 40%). **^{1}H NMR** (500 MHz, $CDCl_3$) δ 1.98 – 2.16 (m, 2H, $SO_2CH_2CH_2$), 2.70 (dd, *J* = 13.8, 8.5 Hz, 1H, $SO_2CH_aH_b$), 2.76 – 2.82 (m, 2H, SCH_2CH_2S), 2.82 – 2.87 (m, 2H, SCH_2CH_2S), 2.90 (dd, *J* = 13.8, 2.5 Hz, 1H, $SCHCH_aH_b$), 2.93 – 2.99 (m, 1H, SC*H*), 3.11 – 3.22 (m, 1H, $SO_2CH_aH_b$), 3.33 (ddd, *J* = 13.8, 10.2, 5.5 Hz, 1H, $SO_2CH_aH_b$), 7.54 – 7.63 (m, 2H, Ar *H*), 7.64 – 7.71 (m, 1H, Ar *H*), 7.89 – 7.95 (m, 2H, Ar *H*). **^{13}C NMR** (126 MHz, $CDCl_3$) δ 27.6 ($SO_2CH_2CH_2$), 28.4 (SCH_2CH_2S), 28.7 (SCH_2CH_2S), 34.7 ($SCHCH_2S$), 39.6 (SCH), 53.6 (SO_2CH_2), 128.1 (Ar *C*H), 129.4 (Ar *C*H), 133.9 (Ar *C*H), 139.0 (Ar *C*). **v_{max}** (thin film/cm^{-1}): 2910, 1446, 3302, 1302, 1264, 1148, 1087, 895, 732, 702, 599, 535. **HRMS** $C_{12}H_{17}O_2S_3$ (M+H$^+$) Expected 289.0385, Found 289.0389.

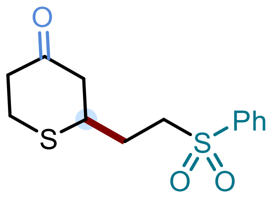

7f

2-(2-(phenylsulfonyl)ethyl)tetrahydro-4*H*-thiopyran-4-one (7f)

7f was prepared according to general method a, using phenyl vinyl sulfone (16.8 mg, 0.1 mmol) and tetrahydrothiopyran-4-one (117 mg, 1 mmol). The crude product was purified using preparative TLC (30% EtOAc: Hexane) to give an off-white oil (10.7 mg, 0.038 mmol, 38%). **^{1}H NMR** (400 MHz, $CDCl_3$) δ 1.95 (dtd, *J* = 14.9, 10.0, 5.2 Hz, 1H, $SO_2CH_2CH_aH_b$), 2.01 – 2.13 (m, 1H, $SO_2CH_2CH_aH_b$), 2.44 (ddd, *J* = 13.9, 9.5, 0.9 Hz, 1H, $SCHCH_aH_bC(O)$), 2.53 – 2.63 (m, 1H, $SCH_2CH_aH_bC(O)$), 2.68 (dddd, *J* = 14.3, 6.1, 4.1, 1.1 Hz, 1H, $SCH_2CH_aH_bC(O)$), 2.76 (ddd, *J* = 13.9, 3.7, 1.1 Hz, 1H, $SCHCH_aH_bC(O)$), 2.84 (td, *J* = 9.6, 5.0 Hz, 1H, SCH_aH_b), 2.93 (ddd, *J* = 13.9, 6.1, 5.0 Hz, 1H, SCH_aH_b), 3.11 – 3.25 (m, 2H, SC*H*, $SO_2CH_aH_b$), 3.29 – 3.38 (m, 1H, $SO_2CH_aH_b$), 7.56 – 7.65 (m, 2H, Ar *H*), 7.66 – 7.77 (m, 1H, Ar *H*), 7.84

– 7.96 (m, 2H, Ar *H*). **^{13}C NMR** (101 MHz, $CDCl_3$) δ 27.4 ($SCH_2CH_2C(O)$), 28.0 ($SO_2CH_2CH_2$), 42.9 (S*C*H), 43.1 (S*C*H_2), 50.1 ($SCHCH_2C(O)$), 53.7 (SO_2CH_2), 128.1 (Ar *C*H), 129.5 (Ar *C*H), 134.0 (Ar *C*H), 138.9 (Ar *C*), 207.1 (*C*=O). **v_{max}** (thin film/cm^{-1}): 3053, 1730, 1422, 1265, 896, 739, 706. **HRMS** $C_{13}H_{17}O_3S_2$ ($M+H^+$) Expected 285.0614, Found 285.0606.

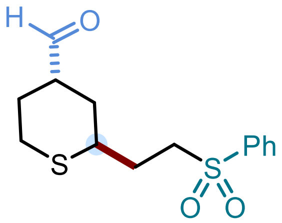

7g

2-(2-(phenylsulfonyl)ethyl)tetrahydro-2*H*-thiopyran-4-carbaldehyde (7g)

7g was prepared according to general method a, using phenyl vinyl sulfone (16.8 mg, 0.1 mmol) and tetrahydrothiopyran-4-carbaldehyde (88.1 mg, 1.0 mmol). The crude product was purified using column chromatography (30% EtOAc: Hexane) to give an off-white oil as an inseparable mixture of diastereomers (16.9 mg, 0.058 mmol, 58%, dr 1:10). **^{1}H NMR** (400 MHz, $CDCl_3$) δ 1.20 – 1.34 (m, 1.1H, $SCHCH_aH_bCH$, D1+D2), 1.36 – 1.47 (m, 1.1H, $SCH_2CH_aH_b$, D1+D2), 1.73 – 1.83 (m, 1.1H, SO_2CH_2CH-$_aH_b$, D1+D2), 1.97 – 2.07 (m, 1.1H, $SO_2CH_2CH_aH_b$, D1+D2), 2.14 – 2.29 (m, 3.3H, $SCHCH_aH_bCH$, $SCH_2CH_aH_b$, CHOC*H,* D1+D2), 2.58 – 2.69 (m, 2.2H, S*C*H_2, D1+D2), 2.75 – 2.83 (m, 1.1H, SC*H*, D1+D2), 3.10 – 3.37 (m, 2.2H, SO_2CH_2, D1+D2), 7.48 – 7.55 (m, 2.2H, Ar C*H*, D1+D2), 7.57 – 7.64 (m, 1.1H, Ar C*H*, D1+D2), 7.81 – 7.88 (m, 2H, Ar C*H*, D1+D2), 9.58 (s, 1H, C*H*O, D2), 9.63 (s, 0.1H, C*H*O, D1). **^{13}C NMR** (101 MHz, $CDCl_3$) δ 26.9 (SCH_2CH_2, D1+D2), 28.4 (S*C*H_2, D1+D2), 28.9 ($SO_2CH_2CH_2$, D1+D2), 34.0 ($SCHCH_2CH$, D1+D2), 40.9 (S*C*H D1+D2), 50.2 (CHO*C*H, D1+D2), 53.4 (SO_2CH_2, D1+D2), 128.1 (Ar *C*H, D1+D2), 129.4 (Ar *C*H, D1+D2), 133.9 (Ar *C*H, D1+D2), 138.9 (Ar *C*, D1+D2), 202.2 (*C*HO, D1+D2). **v_{max}** (thin film/cm^{-1}): 2918, 1723, 1146, 1305, 1149, 1087, 903, 538. **HRMS** $C_{14}H_{19}O_3S_2$ ($M+H^+$) Expected 299.0770, Found 295.0762.

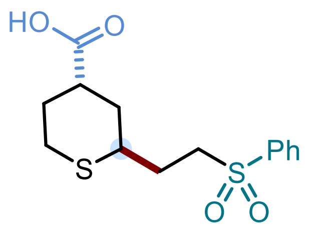

7h

2-(2-(phenylsulfonyl)ethyl)tetrahydro-2*H*-thiopyran-4-carboxylic acid (7h)

7h was prepared according to general method a, using phenyl vinyl sulfone (16.8 mg, 0.1 mmol) and tetrahydro-2*H*-thiopyran-4-carboxylic acid (146 mg, 1 mmol). The crude product was purified using column chromatography (20% EtOAc: Hexane) to give an off-white oil as a mixture of diastereomers (18.8 mg, 0.060 mmol 60%, dr 1:5). **^{1}H NMR** (500 MHz, $CDCl_3$) δ 1.51 (q, *J* = 12.3 Hz, 1H, SCHC$H_a$$H_b$CH, D1), 1.61 – 1.76 (m, 1H, SCH_2C$H_a$$H_b$, D1), 1.77 – 1.90 (m, 6H, SO_2CH_2C$H_a$$H_b$, D1+D2), 1.90 – 2.07 (m, 16H, SO_2CH_2C$H_a$$H_b$, D1 + SCH_2C$H_a$$H_b$, D2 + SCHC$H_2$CH, D2), 2.09 – 2.18 (m, 5H, SCH_2C$H_a$$H_b$, D2), 2.20 – 2.30 (m, 7H, SO_2CH_2C$H_a$$H_b$, D2 + SCH_2C$H_a$$H_b$, D1 + SCHC$H_a$$H_b$CH, D1), 2.30 – 2.41 (m, 1H, C*H*COOH, D1), 2.50 – 2.58 (m, 5H, SC$H_a$$H_b$, D2), 2.60 – 2.70 (m, 7H, SC$H_a$$H_b$, D2 + SC$H_2$, D1), 2.71 – 2.77 (m, 5H, C*H*COOH, D2), 2.78 – 2.84 (m, 1H, SC*H*, D1), 2.85 – 2.94 (m, 5H, SC*H*, D2), 3.09 – 3.20 (m, 6H, SO_2C$H_a$$H_b$, D1+D2), 3.20 – 3.29 (m, 1H, SO_2C$H_a$$H_b$, D1), 3.30 – 3.42 (m, 5H, SO_2C$H_a$$H_b$, D2), 7.58 (t, *J* = 7.6 Hz, 12H, Ar C*H,* D1+D2), 7.67 (t, *J* = 7.4 Hz, 6H, Ar C*H,* D1+D2), 7.87 – 7.96 (m, 12H, Ar C*H,* D1+D2). **^{13}C NMR** (126 MHz, $CDCl_3$) δ 24.2 (S*C*H_2, D2), 27.3 (SCH*C*H_2CH, D2), 28.4 (S*C*H_2, D1), 28.4 (S*C*$H_2$$CH_2$, D2), 28.8 ($SO_2CH_2$*C*$H_2$, D1), 29.3 ($SCH_2$*C*$H_2$, D1), 34.8 ($SO_2CH_2$*C*$H_2$, D2), 36.4 (SCH*C*$H_2$CH, D1), 37.2 (S*C*H, D2), 38.1 (*C*HCOOH, D2), 40.8 (S*C*H, D1), 43.0 (*C*HCOOH, D1), 53.4 (SO_2*C*H_2, D1), 54.2 (SO_2*C*H_2, D2), 128.0 (Ar *C*H, D2), 128.1 (Ar *C*H, D1), 129.4 (Ar *C*H, D1), 129.4 (Ar *C*H, D2), 133.8 (Ar *C*H, D2), 133.9 (Ar *C*H, D1), 138.9 (Ar *C*, D1), 139.0 (Ar *C*, D2), 179.5 (*C*O, D1) 179.6 (*C*O, D2). **ν_{max}** (thin film/cm^{-1}): 3001, 2984, 1737, 1447, 1372, 1233, 1098, 1043, 938, 847. **HRMS** $C_{14}H_{18}O_4NaS$ ($M+Na^+$) Predicted 337.0539, Found 337.0529.

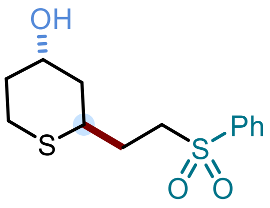

7i

2-(2-(phenylsulfonyl)ethyl)tetrahydro-2H-thiopyran-4-ol (7i)

7i was prepared according to general method a, using phenyl vinyl sulfone (16.8 mg, 0.1 mmol) and tetrahydrothiopyran-4-ol (71 mg, 0.60 mmol). The crude product was purified using preparative TLC (30% EtOAc: Hexane) to give an off-white oil (12.4 mg, 0.043 mmol, 43%, dr > 20:1). **^{1}H NMR** (500 MHz, $CDCl_3$) δ 1.72 (ddd, *J* = 13.3, 10.0, 2.7 Hz, 1H, $SO_2CH_2CH_aH_b$), 1.89 (m, 3H, $SCH_2CH_aH_b$, SCH_2), 1.93 – 2.03 (m, 2H, SCH_2CHaHb, $SO_2CH_2CH_aH_b$), 2.41 (m, 1H, $SCHCH_aH_bCHOH$), 2.93 (td, *J* = 8.3, 3.9 Hz, 1H, $SCHCH_aH_bCHOH$), 3.07 – 3.19 (m, 2H, SC*H*, $SO_2CH_aH_b$), 3.33 (ddd, *J* = 13.9, 11.2, 4.9 Hz, 1H, $SO_2CH_aH_b$), 4.09 (s, 1H, C*H*OH), 7.58 (t, *J* = 7.7 Hz, 2H, Ar *H*), 7.63 – 7.70 (m, 1H, Ar *H*), 7.91 (dd, *J* = 7.8, 1.6 Hz, 2H, Ar *H*). **^{13}C NMR** (126 MHz, $CDCl_3$) δ 22.9 ($SCHCH_2CHOH$), 28.1 (SCH_2CH_2), 34.1 (SCH_2), 35.8 (S*C*H), 41.0 ($SO_2CH_2CH_2$), 54.1 (SO_2CH_2), 65.3 (*C*HOH), 128.0 (Ar *C*H), 129.4 (Ar *C*H), 133.8 (Ar *C*H), 139.4 (Ar *C*). **v_{max}** (thin film/cm^{-1}): 3409 (br), 2917, 1579, 1446, 1303, 1149, 1086, 1039, 927, 798, 745, 690. **HRMS** $C_{13}H_{19}O_3S_2$ ($M+H^+$) Expected 287.0770, Found 287.0759.

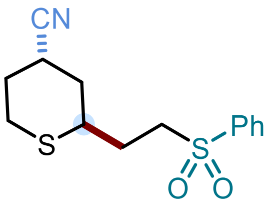

7j

2-(2-(phenylsulfonyl)ethyl)tetrahydro-2*H*-thiopyran-4-carbonitrile (7j)

7j was prepared according to general method a, using phenyl vinyl sulfone (16.8 mg, 0.1 mmol) and tetrahydrothiopyran-4-carbonitrile (127 mg, 1.0 mmol). The crude product was purified using column chromatography (30% EtOAc: Hexane) to give an off-white oil (11.2 mg, 0.038 mmol, 38%, dr: 1:2). **^{1}H NMR** (400 MHz, $CDCl_3$) δ 1.63 – 1.78 (m, 3H, $SCH_2CH_aH_b$, D1+D2), 1.80 – 1.94 (m, 6H, $SO_2CH_2CH_aH_b$, D1+D2 + $SCHCH_aH_bCH$, D1+D2), 1.97 – 2.11 (m, 3H, $SO_2CH_2CH_aH_b$, D1+D2), 2.12 – 2.23 (m, 4H, $SCHCH_aH_bCH$, D2 + $SCH_2CH_aH_b$, D2), 2.25 – 2.38 (m, 2H, $SCHCH_aH_bCH$, D1 + $SCH_2CH_aH_b$, D1), 2.50

– 2.56 (m, 1H, CNC*H*, D1), 2.56 – 2.65 (m, 2H, SC$H_a$$H_b$, D2), 2.65 – 2.71 (m, 2H, SCH_2, D1), 2.75 - 2.86 (m, 1H, SC*H,* D1), 2.92 (dtd, *J* = 14.4, 11.8, 2.7 Hz, 2H, SC$H_a$$H_b$, D2), 2.96 – 3.06 (m, 2H, SC*H*, D2), 3.10 (m, 2H, C*H*CN, D2), 3.12 – 3.20 (m, 3H, SO_2C$H_a$$H_b$, D1+D2), 3.20 – 3.28 (m, 1H, SO_2C$H_a$$H_b$, D1), 3.28 – 3.35 (m, 2H, SO_2C$H_a$$H_b$, D2), 7.56 – 7.63 (m, 6H, Ar C*H*, D1 + D2), 7.65 – 7.71 (m, 3H, Ar C*H*, D1 + D2), 7.85 – 7.96 (m, 6H, Ar C*H*, D1 + D2). **^{13}C NMR** (101 MHz, $CDCl_3$) δ 24.6 (S*C*H_2, D2), 27.2 (*C*HCN, D2), 27.6 (S*C*H_2, D1), 28.0 (SO_2CH_2*C*H_2, D2), 28.5 (SO_2CH_2*C*H_2, D1), 28.7 (*C*HCN, D1), 29.1 (SCH*C*H_2CH, D2), 30.2 (SCH*C*H_2CH, D2), 36.0 (SCH_2*C*H_2, D2), 37.1 (SCH_2*C*H_2, D1), 37.1 (S*C*H, D2), 40.0 (S*C*H, D1), 53.1 (SO_2*C*H_2, D2), 53.6 (SO_2*C*H_2, D1), 120.6 (*C*N, D2), 121.1 (*C*N, D1), 128.0 (Ar *C*H, D2) 128.1 (Ar *C*H, D1), 129.5 (Ar *C*H, D2), 129.8 (Ar *C*H, D1), 134.0 (Ar *C*H, D2), 134.0 (Ar *C*H, D1), 138.9 (Ar *C*, D2), 138.9 (Ar *C*, D1). **v_{max}** (thin film/cm^{-1}): 2922, 1711, 1446, 1306, 1149, 1306, 1149, 1086, 803, 744, 690, 594. **HRMS** $C_{14}H_{17}NO_2S_2$ ($M+H^+$) predicted 296.0773, found 296.0762.

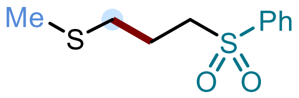

7k

Methyl(3-(phenylsulfonyl)propyl)sulfane (7k)

7k was prepared according to general method a, using phenyl vinyl sulfone (16.8 mg, 0.1 mmol) and dimethyl sulfide (62.1 mg, 73.9 μL, 1 mmol). The crude product was purified using column chromatography (30% EtOAc: Hexane) to give an off-white oil (14.9 mg, 0.065 mmol, 65%). **^{1}H NMR** (500 MHz, $CDCl_3$) δ 1.98 – 2.07 (m, 5H, SO_2CH_2CH_2, CH_3S), 2.57 (t, *J* = 6.9 Hz, 2H, SCH_2), 3.20 – 3.27 (m, 2H, SO_2CH_2), 7.58 (dd, *J* = 8.4, 7.1 Hz, 2H, Ar C*H*), 7.62 – 7.70 (m, 1H, Ar C*H*), 7.87 – 7.95 (m, 2H, Ar C*H*). **^{13}C NMR** (126 MHz, $CDCl_3$) δ 15.1 (S*C*H_3), 21.8 (SCH_2*C*H_2), 32.4 (S*C*H_2), 54.8 (SO_2CH_2), 128.0 (Ar *C*H), 129.7 (Ar *C*H), 133.8 (Ar *C*H), 139.1 (Ar *C*). **v_{max}** (thin film/cm^{-1}): 2919, 1447, 1306, 1148, 1087, 734, 690, 591, 568, 532. **HRMS** $C_{10}H_{15}O_2S_2$ ($M+H^+$) Expected 231.0508, Found 231.0501.

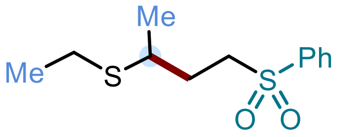

7l

Ethyl(4-(phenylsulfonyl)butan-2-yl)sulfane (7l)

7l was prepared according to general method a, using phenyl vinyl sulfone (16.8 mg, 0.1 mmol) and diethyl sulfide (90.9 mg, 103 μL, 1 mmol). The crude product was purified using column chromatography (30% EtOAc: Hexane) to give an off-white oil (17.8 mg, 0.069 mmol, 69%). **^{1}H NMR** (400 MHz, $CDCl_3$) δ 1.19 (t, J = 7.4 Hz, 3H, CH_3CH_2), 1.25 (d, J = 6.9 Hz, 3H, CH_3CH), 1.81 – 2.07 (m, 2H, $SO_2CH_2CH_2$), 2.45 (q, J = 7.4 Hz, 2H, CH_3CH_2), 2.76 – 2.91 (m, 1H, SC*H*), 3.18 – 3.36 (m, 2H, SO_2CH_2), 7.52 – 7.63 (m, 2H, Ar *H*), 7.63 – 7.73 (m, 1H, Ar *H*), 7.89 – 7.96 (m, 2H, Ar *H*). **^{13}C NMR** (101 MHz, $CDCl_3$) δ 14.8 (CH_3CH_2), 21.4 (CH_3CH), 24.0 ($SO_2CH_2CH_2$), 29.0 (CH_3CH_2), 38.4 (S*C*H), 53.7 (SO_2CH_2), 128.1 (Ar *C*H), 129.3 (Ar *C*H), 133.7 (Ar *C*H), 139.2 (Ar *C*). **v_{max}** (thin film/cm^{-1}): 2923, 1446, 1035, 1086, 800, 746, 689, 558. **HRMS** $C_{12}H_{19}O_2S_2$ (M+H$^+$) Expected 259.0821, Found 259.0815.

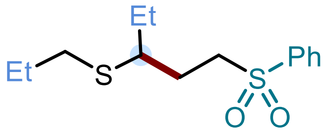

7m

(1-(phenylsulfonyl)pentan-3-yl)(propyl)sulfane (7m)

7m was prepared according to general method a, using phenyl vinyl sulfone (16.8 mg, 0.1 mmol) and dipropylsulfide (118 mg, 141 μL, 1 mmol). The crude product was purified using column chromatography (30% EtOAc: Hexane) to give an off-white oil (17.5 mg, 0.064 mmol, 64%). **^{1}H NMR** (400 MHz, $CDCl_3$) δ 0.90 – 0.98 (m, 6H, 2 × CH_3), 1.44 – 1.60 (m, 4H, 2 × CH_3CH_2), 1.83 (dddd, J = 14.0, 10.5, 8.3, 5.5 Hz, 1H, $SO_2CH_2CH_aH_b$), 2.03 (dddd, J = 14.1, 10.3, 5.5, 4.5 Hz, 1H, $SO_2CH_2CH_aH_b$), 2.29 – 2.38 (m, 2H, SCH_2), 2.56 (dtd, J = 8.3, 6.7, 4.5 Hz, 1H, SC*H*), 3.21 – 3.39 (m, 2H, SO_2CH_2), 7.53 – 7.63 (m, 2H, Ar *H*), 7.63 – 7.73 (m, 1H, Ar *H*), 7.89 – 7.96 (m, 2H, Ar *H*). **^{13}C NMR** (101 MHz, $CDCl_3$) δ 11.4 (CH_3),

13.6 (CH_3), 23.1 (CH_3CH_2), 26.8 ($SO_2CH_2CH_2$), 27.9 (CH_3CH_2), 32.1 (SCH_2), 46.1 (S*C*H), 53.6 (SO_2CH_2), 128.0 (Ar *C*H), 129.3 (Ar *C*H), 133.7 (Ar *C*H), 139.2 (Ar *C*). **v_{max}** (thin film/cm^{-1}): 2962, 2925, 2873, 1447, 1305, 1087, 798, 660, 606. **HRMS** $C_{14}H_{23}O_2S_2$ ($M+H^+$) Expected 287.1134, Found 287.1124.

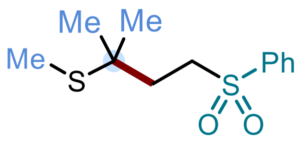

7n (R1)

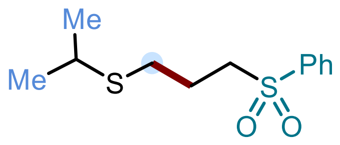

7n (R2)

Methyl(2-methyl-4-(phenylsulfonyl)butan-2-yl)sulfane (R1) and isopropyl(3-(phenylsulfonyl)propyl)sulfane (R2) (7n)

7n was prepared according to general method a, using phenyl vinyl sulfone (16.8 mg, 0.1 mmol) and isopropyl methyl sulfide (111 µL, 1 mmol). The crude product was purified using column chromatography (20% EtOAc: Hexane) to give an off-white oil as a separable regioisomers (Combined yield 7.7 mg, 0.03 mmol, 30%, rr 1:1).

Regioisomer 1: **^{1}H NMR** (500 MHz, $CDCl_3$) δ 1.23 (s, 6H, CH_3), 1.83 (s, 3H, SCH_3), 1.84 – 1.90 (m, 2H, $SO_2CH_2CH_2$), 3.23 – 3.31 (m, 2H, SO_2CH_2), 7.59 (dd, J = 8.4, 7.0 Hz, 2H, Ar C*H*), 7.63 – 7.71 (m, 1H, Ar C*H*), 7.90 – 7.96 (m, 2H, Ar C*H*). **^{13}C NMR** (126 MHz, $CDCl_3$) δ 10.6 (SCH_3), 28.2 ($C(CH_3)_2$), 32.7 ($SO_2CH_2CH_2$), 43.0 ($C(CH_3)_2$), 52.9 (SO_2CH_2), 128.0 (Ar *C*H), 129.3 (Ar *C*H), 133.7 (Ar *C*H), 139.2 (Ar *C*). **v_{max}** (thin film/cm^{-1}): 2922, 2853, 1446, 1304, 1146, 1086, 797, 590, 560. **HRMS** $C_{12}H_{19}O_2S_2$ ($M+H^+$) Expected 259.0821, Found 295.0810.

Regioisomer 2: **^{1}H NMR** (400 MHz, $CDCl_3$) δ 1.15 (d, J = 6.8 Hz, 6H, 2 × CH_3), 1.87 – 1.99 (m, 2H, SCH_2CH_2), 2.53 (t, J = 7.0 Hz, 2H, SCH_2), 2.77 (hept, J = 6.7 Hz, 1H, SC*H*), 3.13 – 3.29 (m, 2H, SO_2CH_2), 7.47 – 7.56 (m, 2H, Ar CH), 7.56 – 7.63 (m, 1H, Ar CH), 7.82 – 7.89 (m, 2H, Ar CH). **^{13}C NMR** (101 MHz, $CDCl_3$) δ 22.7 (SCH_2CH_2), 23.3 ($CH(CH_3)_2$), 28.9 (SCH_2), 34.7 (S*C*H), 55.0 (SO_2CH_2), 128.1 (Ar *C*H), 129.3 (Ar *C*H), 129.5 (Ar *C*H), 133.8 (Ar *C*). **v_{max}** (thin film/cm^{-1}): 2922, 2853, 1446, 1304, 1146, 1086, 797, 590, 560 **HRMS** $C_{12}H_{19}O_2S_2$ ($M+H^+$) Expected 259.0821, Found 295.0810.

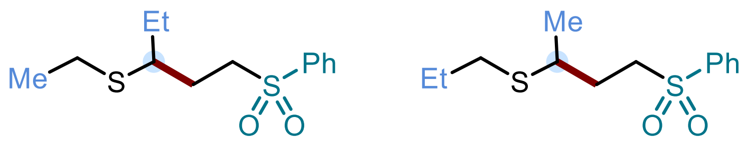

7o (R1) **7o (R2)**

Ethyl(1-(phenylsulfonyl)pentan-3-yl)sulfane (R1) and 4-(phenylsulfonyl)butan-2-yl)(propyl)sulfane (R2) (7o)

7o was prepared according to general method a, using phenyl vinyl sulfone (16.8 mg, 0.1 mmol) and ethyl propyl sulfide (123 μL,1 mmol). The crude product was purified using column chromatography (20% EtOAc: Hexane) to give an off-white oil as an inseparable mixture of regioisomers (18.5 mg, 68%, rr 1:1.6). **^{1}H NMR** (500 MHz, $CDCl_3$) δ 0.95 (q, *J* = 7.2 Hz, 7.8H, CH_3CH_2CH (R1) + $CH_3CH_2CH_2$ (R2)), 1.18 (t, *J* = 7.4 Hz, 3H, CH_3CH_2S (R1)), 1.25 (d, *J* = 6.8 Hz, 4.8H, CH_3CH (R2)), 1.47 – 1.58 (m, 5.2H, CH_3CH_2CH (R1) + $CH_3CH_2CH_2$ (R2)), 1.78 – 1.92 (m, 2.6H, $SO_2CH_2CH_aH_b$ (R1+R2)), 1.92 – 2.09 (m, 2.6H ,$SO_2CH_2CH_aH_b$ (R1+R2)), 2.35 – 2.44 (m, 5.2H, SCH_2 (R1+R2)), 2.55 – 2.63 (m, 1H, SC*H*, R1)), 2.77 – 2.87 (m, 1.6H, SC*H*, R2), 3.20 – 3.40 (m, 5.2H, SO_2CH_2 (R1+R2)), 7.54 – 7.61 (m, 5.2H, Ar C*H* (R1+R2)), 7.64 – 7.69 (m, 2.6H, Ar C*H* (R1+R2)), 7.90 – 7.94 (m, 5.2H, Ar C*H* (R1+R2)). **^{13}C NMR** (126 MHz, $CDCl_3$) δ 11.4 (CH_3CH_2CH (R1)), 13.6 ($CH_3CH_2CH_2$ (R2)), 14.8 (CH_3CH_2S (R1)), 21.5 (CH_3CH (R2), 23.0 (CH_3CH_2CH (R1)), 24.0 (SCH_2 (R1)), 26.7 ($SO_2CH_2CH_2$ (R2)), 27.8 ($CH_3CH_2CH_2$ (R2)), 29.1 ($SO_2CH_2CH_2$ (R1)), 32.1 (SCH_2 (R2)), 38.7 (S*C*H (R2)), 45.8 (S*C*H (R1)), 53.6 (SO_2CH_2 (R1), 53.7 (SO_2CH_2 (R2), 128.0 (Ar *C*H (R1)), 128.0 (Ar *C*H (R2)), 129.3 (Ar *C*H (R1)), 129.3 (Ar *C*H (R2)), 133.7 (Ar *C*H (R1)), 133.7 (Ar *C*H (R2)), 139.2 (Ar *C* (R2)), 139.2 (Ar *C* (R1)). **v_{max}** (thin film/cm^{-1}): 2984, 1447, 1045, 913, 847, 648, 608. **HRMS** $C_{13}H_{21}O_2S_2$ ($M+H^+$) Expected 273.0977, Found 273.0972.

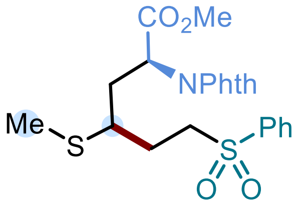

7p (R1+R2)

Methyl-2-(1,3-dioxoisoindolin-2-yl)-4-(methylthio)-6-(phenylsulfonyl)hexanoate (R1) and methyl-2-(1,3-dioxoisoindolin-2-yl)-4-((3-(phenylsulfonyl)propyl)thio)butanoate (R2) (7p)

7p was prepared according to general method a, using phenyl vinyl sulfone (16.8 mg, 0.1 mmol) and phthalimide methionine methyl ester (87.9 mg, 0.3 mmol). The crude product was purified using column chromatography (40% EtOAc: Hexane) to give an off-white oil as an inseparable mixture of diastereomers and regioisomers (18.4 mg, 0.040 mmol, 40%, rr 2 (dr 1:1):1). **^{1}H NMR** (500 MHz, $CDCl_3$) δ 1.85 (s, 3H, SC*H*$_3$), 1.91 (s, 3H, SC*H*$_3$), 1.93 – 2.00 (m, 3H), 2.01 – 2.10 (m, 3H), 2.19 – 2.62 (m, 10H), 2.76 – 2.86 (m, 1H), 3.17 – 3.43 (m, 6H), 3.71 (s, 3H, OC*H*$_3$), 3.72 (s, 3H, OC*H*$_3$), 3.73 (s, 3H, OC*H*$_3$), 5.04 (t, *J* = 7.2 Hz, 1H, C*H*CO_2Me), 5.16 (t, *J* = 7.1 Hz, 1H, C*H*CO_2Me), 5.35 (dd, *J* = 11.4, 3.3 Hz, 1H, C*H*CO_2Me), 7.51 – 7.69 (m, 9H, Ar C*H*), 7.70 – 7.80 (m, 6H, Ar C*H*), 7.83 – 7.95 (m, 12H, Ar C*H*). **^{13}C NMR** (126 MHz, $CDCl_3$) δ 11.0 (S*C*H$_3$), 11.1 (SCH$_3$), 22.3, 26.4, 27.3, 28.5, 28.5, 30.2, 32.7, 33.8, 41.9 (S*C*H), 42.4 (S*C*H), 49.5 (*C*HCO_2Me), 50.3 (*C*HCO_2Me), 50.7 (*C*HCO_2Me), 53.0 (2 × OCH$_3$), 53.0 (O*C*H$_3$), 53.6, 53.6, 54.7, 123.7, 123.7, 128.0, 128.1, 129.4, 129.8, 131.8, 131.78, 133.8, 134.3, 134.4, 134.4, 134.6, 139.0, 139.0, 167.4 (*C*O), 167.7 (*C*O), 167.8 (*C*O), 169.4 (*C*O), 169.5 (*C*O), 169.7 (*C*O). **ν_{max}** (thin film/cm^{-1}): 2922, 1776, 1744, 1715, 1447, 1388, 1306, 1149, 1087, 722, 690. **HRMS** $C_{22}H_{24}O_6NS_2$ (M+H$^+$) Predicted 462.1040, Found 462.1039.

Alkene Scope

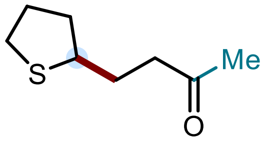

7q

4-(tetrahydrothiophen-2-yl)butan-2-one (7q)

7q was prepared according to general method a, using methyl vinyl ketone (8.3 µL, 0.1 mmol) and tetrahydrothiophene (88.1 µL, 1.0 mmol). The crude product was purified using column chromatography (20% EtOAc: Hexane) to give an off-white oil (12.1 mg, 0.076 mmol, 76%). **^{1}H NMR** (400 MHz, $CDCl_3$) δ 1.46 – 1.57 (m, 1H, $SCH_2CH_2C\mathit{H}_aH_b$), 1.58 – 1.69 (m, 1H, $COCH_2C\mathit{H}_aH_b$), 1.78 – 2.03 (m, 4H, $COCH_2CH_a\mathit{H}_b$, $SCH_2C\mathit{H}_2$, $SCH_2CH_2CH_a\mathit{H}_b$), 2.08 (s, 3H, $C\mathit{H}_3$), 2.37 – 2.55 (m, 2H, $COC\mathit{H}_2$), 2.73 – 2.85 (m, 2H, $SC\mathit{H}_2$), 3.22 – 3.31 (m, 1H, $SC\mathit{H}$). **^{13}C NMR** (126 MHz, $CDCl_3$) δ 30.0 ($\mathit{C}H_3$), 30.2 ($SCH_2\mathit{C}H_2$), 31.4 ($COCH_2\mathit{C}H_2$), 32.2 ($S\mathit{C}H_2$), 37.4 ($SCH_2CH_2\mathit{C}H_2$), 42.8 ($CO\mathit{C}H_2$), 48.5 ($S\mathit{C}H$), 208.3 ($\mathit{C}O$). **ν_{max}** (thin film/cm^{-1}): 2923, 2852, 1714, 1463, 545, 514, 457, 437, 472. **HRMS** $C_8H_{15}SO$ ($M+H^+$) Predicted 159.0838 , Found 159.0839.

The data are in accordance with the literature. [2]

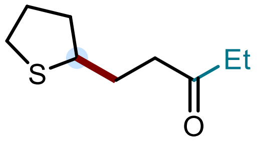

7r

1-(tetrahydrothiophen-2-yl)pentan-3-one (7r)

7r was prepared according to general method a, using ethyl vinyl ketone (9.9 µL, 0.1 mmol) and tetrahydrothiophene (88.1 µL, 1.0 mmol). The crude product was purified using column chromatography (20% EtOAc: Hexane) to give an off-white oil (12.9 mg, 0.075 mmol, 75%). **^{1}H NMR** (500 MHz, $CDCl_3$) δ 1.05 (t, J = 7.4 Hz, 3H, $COCH_2C\mathit{H}_3$), 1.53 – 1.63 (m, 1H, $SCH_2CH_2C\mathit{H}_aH_b$), 1.69 (dtd, J = 13.7, 9.2, 5.6 Hz, 1H, $COCH_2C\mathit{H}_aH_b$), 1.85 – 2.12 (m, 4H, $COCH_2CH_a\mathit{H}_b$, $SCH_2C\mathit{H}_2$, $SCH_2CH_2CH_a\mathit{H}_b$), 2.38 – 2.58 (m, 4H, $COC\mathit{H}_2CH_3$, $COC\mathit{H}_2CH_2$), 2.79 – 2.90 (m, 2H, SCH_2), 3.32 (ddt, J = 9.3, 7.0, 5.6 Hz, 1H, $SC\mathit{H}$). **^{13}C NMR** (101 MHz, $CDCl_3$) δ 7.9 ($COCH_2\mathit{C}H_3$), 30.2 ($SCH_2\mathit{C}H_2$), 31.4 ($COCH_2\mathit{C}H_2$), 32.3 ($S\mathit{C}H_2$), 36.0

($COCH_2CH_3$), 37.4 ($SCH_2CH_2CH_2$), 41.4 ($COCH_2$), 48.6 (SCH), 211.1 (CO). **ν_{max}** (thin film/cm^{-1}): 2921, 2851, 1715, 1444, 1414, 1375, 1264, 1185, 1112, 1024, 83, 735, 702. **HRMS** $C_9H_{17}OS$ ($M+H^+$) Predicted 173.0995, Found 173.0995.

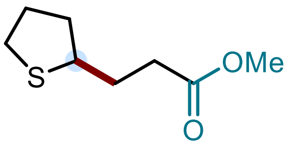

7s

Methyl-3-(tetrahydrothiophen-2-yl)propanoate (7s)

7s was prepared according to general method a, using methyl acrylate (9.0 µL, 0.1 mmol) and tetrahydrothiophene (88.1 µL, 1.0 mmol). The crude product was purified using column chromatography (20% EtOAc: Hexane) to give an off-white oil (9.5 mg, 0.055 mmol, 55%). **^{1}H NMR** (400 MHz, $CDCl_3$) δ 1.59 (m, 1H, $SCH_2CH_2CH_aH_b$), 1.74 – 1.85 (m, 1H, $COCH_2CH_aH_b$), 1.86 – 2.14 (m, 4H, SCH_2CH_2, $COCH_2CH_aH_b$, $SCH_2CH_2CH_aH_b$), 2.30 – 2.49 (m, 2H, $COCH_2$), 2.76 – 2.97 (m, 2H, SCH_2), 3.27 – 3.40 (m, 1H, SCH), 3.67 (s, 3H, CH_3O). **^{13}C NMR** (101 MHz, $CDCl_3$) δ 30.2 (SCH_2CH_2), 32.3 (SCH_2), 32.7 ($COCH_2CH_2$), 33.2 ($COCH_2$), 37.2 ($SCH_2CH_2CH_2$), 48.3 (SCH), 51.6 ($COCH_3$), 173.7 (CO). **ν_{max}** (thin film/cm^{-1}): 2923, 2863, 2035, 1737, 1440, 1260, 1176, 1019, 795. **HRMS** $C_8H_{14}O_2NaS$ ($M+Na^+$) Predicted 197.0608, Found 197.0605.

The data are in accordance with the literature. [2]

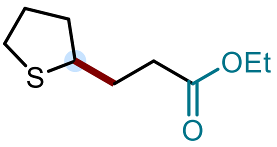

7t

Ethyl-3-(tetrahydrothiophen-2-yl)propanoate (7t)

7t was prepared according to general method a, using methyl acrylate (9.0 µL, 0.1 mmol) and tetrahydrothiophene (88.1 µL, 1.0 mmol). The crude product was purified using column chromatography

(20% EtOAc: Hexane) to give an off-white oil (12.2 mg, 0.065 mmol, 65%). **^{1}H NMR** (500 MHz, $CDCl_3$) δ 1.25 (t, *J* = 7.1 Hz, 3H, OCH_2CH_3), 1.53 – 1.62 (m, 1H, $SCH_2CH_2CH_aH_b$), 1.74 – 1.85 (m, 1H, $COCH_2CH_aH_b$), 1.85 – 2.13 (m, 4H, SCH_2CH_2, $SCH_2CH_2CH_aH_b$, $COCH_2CH_aH_b$), 2.30 – 2.47 (m, 2H, $COCH_2$), 2.80 – 2.92 (m, 2H, SCH_2), 3.27 – 3.41 (m, 1H, SC*H*), 4.12 (q, *J* = 7.1 Hz, 2H, OCH_2). **^{13}C NMR** (101 MHz, $CDCl_3$) δ 14.2 (OCH_2CH_3), 30.2 (SCH_2CH_2), 32.3 (SCH_2), 32.7 ($COCH_2CH_2$), 33.5 ($COCH_2$), 37.1 ($SCH_2CH_2CH_2$), 48.3 (S*C*H), 60.4 (OCH_2), 173.2 (*C*O). **v_{max}** (thin film/cm^{-1}): 2919, 1851, 1729, 1443, 1376, 1265, 1186, 1023, 738, 703. **HRMS** $C_9H_{16}O_2NaS$ (M+Na$^+$) Predicted 211.0763, Found 211.0763.

The data are in accordance with the literature. [2]

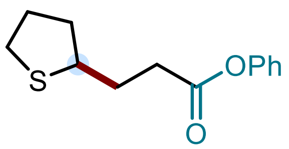

7u

Phenyl-3-(tetrahydrothiophen-2-yl)propanoate (7u)

7u was prepared according to general method a, using phenyl acrylate (13.7 μL, 0.1 mmol) and tetrahydrothiophene (88.1 μL, 1.0 mmol). The crude product was purified using column chromatography (20% EtOAc: Hexane) to give an off-white oil (11.7 mg, 0.050 mmol, 50%). **^{1}H NMR** (400 MHz, $CDCl_3$) δ 1.62 – 1.72 (m, 1H, $SCH_2CH_2CH_aH_b$), 1.88 – 2.01 (m, 2H, $CO_2CH_2CH_aH_b$, $SCH_2CH_aH_b$), 2.02 – 2.21 (m, 3H, $CO_2CH_2CH_aH_b$, $SCH_2CH_2CH_aH_b$, $SCH_2CH_aH_b$), 2.56 – 2.76 (m, 2H, CO_2CH_2), 2.81 – 2.99 (m, 2H, SCH_2), 3.38 – 3.53 (m, 1H, SC*H*), 7.05 – 7.11 (m, 2H, Ar C*H*), 7.19 – 7.24 (m, 1H, Ar C*H*), 7.33 – 7.42 (m, 2H, Ar C*H*). **^{13}C NMR** (101 MHz, $CDCl_3$) δ 30.2 (SCH_2CH_2), 32.3 (SCH_2), 32.6 ($CO_2CH_2CH_2$), 33.5 (CO_2CH_2), 37.2 ($SCH_2CH_2CH_2$), 48.3 (S*C*H), 121.6 (Ar *C*H), 125.8 (Ar *C*H), 129.4 (Ar *C*H), 150.7 (Ar *C*), 171.7 (*C*O). **HRMS** $C_{13}H_{16}O_2NaS$ (M + Na$^+$) predicted 259.0763, Found 259.0759

The data are in accordance with the literature.[2]

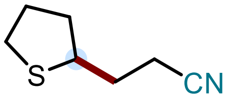

7v

3-(tetrahydrothiophen-2-yl)propanenitrile (7v)

7v was prepared according to general method a, using acrylonitrile (6.55 µL, 0.1 mmol) and tetrahydrothiophene (88.1 µL, 1.0 mmol). The crude product was purified using column chromatography (20% EtOAc: Hexane) to give an off-white oil (8.5 mg, 0.060 mmol, 60%). **^{1}H NMR** (500 MHz, $CDCl_3$) δ 1.60 – 1.68 (m, 1H, $SCH_2CH_2C\textit{H}_aH_b$), 1.77 – 1.86 (m, 1H, $NCCH_2C\textit{H}_aH_b$), 1.93 – 2.10 (m, 3H, $SCH_2C\textit{H}_2$ + $NCCH_2CH_a\textit{H}_b$), 2.11 – 2.18 (m, 1H, $SCH_2CH_2CH_a\textit{H}_b$), 2.33 – 2.43 (m, 1H, $NCCH_a\textit{H}_b$), 2.44 – 2.55 (m, 1H, $NCC\textit{H}_aH_b$), 2.83 – 2.95 (m, 2H, $SC\textit{H}_2$), 3.40 – 3.49 (m, 1H, $SC\textit{H}$). **^{13}C NMR** (126 MHz, $CDCl_3$) δ 16.6 ($NC\textit{C}H_2$), 30.3 ($SCH_2\textit{C}H_2$), 32.5 ($S\textit{C}H_2$), 33.3 ($NCCH_2\textit{C}H_2$), 37.0 ($SCH_2CH_2\textit{C}H_2$), 47.6 ($S\textit{C}H$), 119.5 ($\textit{C}N$). **HRMS**: $C_7H_{11}NSNa$ ($M+Na^+$), predicted 164.0504, found 164.0504.

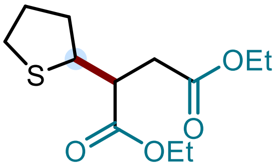

7w

Diethyl-2-(tetrahydrothiophen-2-yl)succinate (7w)

7w was prepared according to general method a, using diethyl maleate (16.1 µL, 0.1 mmol) and tetrahydrothiophene (88.1 µL, 1.0 mmol). The crude product was purified using column chromatography (20% EtOAc: Hexane) to give an off-white oil as a mixture of inseparable diastereomers (16.6 mg, 64%, 0.064 mmol, dr 1:1.3). **^{1}H NMR** (400 MHz, $CDCl_3$) δ 1.24 – 1.27 (m, 6H, 2 × $C\textit{H}_3$, D1 + 7.8H 2 × $C\textit{H}_3$, D2), 1.54 – 1.70 (m, 1.3H, $SCH_2CH_2C\textit{H}_aH_b$ (D2)), 1.71 – 1.81 (m, 1H, $SCH_2CH_2C\textit{H}_aH_b$ (D1)) 1.82 – 1.97 (m, 1H, $SCH_2CH_a\textit{H}_b$ D1 + 1.3H, $SCH_2CH_a\textit{H}_b$, D2)), 1.99 – 2.15 (m, 1H, $SCH_2C\textit{H}_aH_b$, D1 + 1.3H, $SCH_2C\textit{H}_aH_b$, D2 + 1.3H, $SCH_2CH_2CH_a\textit{H}_b$, D2 + 1H, $SCH_2CH_2CH_a\textit{H}_b$, D1), 2.63 – 2.78 (m, 2H, $COC\textit{H}_2$, D1 + 2.6H, $COC\textit{H}_2$, D2), 2.79 – 2.90 (m, 1H, $COC\textit{H}$ (D1) + 2H, SCH_2, D1 + 2.6H, SCH_2, D2), 2.98 – 3.07 (m, 1.3H, $COC\textit{H}$, D2), 3.44 – 3.54 (m, 1H, SCH, D1), 3.67 – 3.77 (m, 1.3 H, $SC\textit{H}$, D2), 4.01 – 4.24 (m, 4H, 2 × $OC\textit{H}_2$, D1 + 5.2H, 2 × $OC\textit{H}_2$, D2). **^{13}C NMR** (101 MHz, $CDCl_3$) δ 13.1 ($COCH_2\textit{C}H_3$, D1),

13.1, (COCH$_2$*C*H$_3$, D2) 13.2 (COCH$_2$*C*H$_3$, D1), 13.2 (COCH$_2$*C*H$_3$, D2), 29.2 (SCH$_2$*C*H$_2$, D1), 29.8 (SCH$_2$*C*H$_2$, D2), 31.3 (S*C*H$_2$, D1), 31.6 (S*C*H$_2$, D2), 32.6 (CO*C*H$_2$, D1), 33.0 (CO*C*H$_2$, D2), 34.3 (SCH$_2$CH$_2$*C*H$_2$, D1), 35.5 (SCH$_2$CH$_2$*C*H$_2$, D2), 45.5 (CO*C*H, D2), 47.1 (CO*C*H, D1), 48.2 (S*C*H, D2), 49.3 (S*C*H, D1), 59.7 (O*C*H$_2$, D1), 59.7 (O*C*H$_2$, D2), 59.8 (O*C*H$_2$, D1), 59.9 (O*C*H$_2$, D2), 170.7 (*C*O, D1), 170.8 (*C*O, D2), 172.1 (*C*O, D1), 172.4 (*C*O, D2). **ν_{max}** (thin film/cm^{-1}): 2932, 2861, 1731, 1444, 1371, 1336, 1258, 1162, 1097, 1030, 857. **HRMS** $C_{12}H_{21}O_4S$ (M+H$^+$) Predicted 261.1155, Found 261.1151.

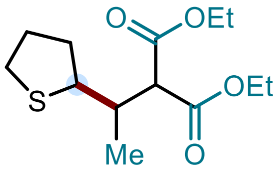

7x

Diethyl 2-(1-(tetrahydrothiophen-2-yl)ethyl)malonate (7x)

7x was prepared according to general method a, using diethyl 2-ethylidenemalonate (18.3 μL, 0.1 mmol) and tetrahydrothiophene (88.1 μL, 1.0 mmol). The crude product was purified using column chromatography (20% EtOAc: Hexane) to give an off-white oil (11.6 mg, 43%, 0.043 mmol, dr 1:1). **1H NMR** (400 MHz, CDCl$_3$) δ 1.09 (d, *J* = 6.8 Hz, 3H, CHC*H*$_3$, D1), 1.13 (d, *J* = 6.8 Hz, 3H, CHC*H*$_3$, D2), 1.25 – 1.27 (m, 6H, 2 × C*H*$_3$CH$_2$O + 6H, 2 × C*H*$_3$CH$_2$O, D2), 1.51 – 1.63 (m, 2H, SCH$_2$CH$_2$C*H*$_a$H$_b$, D1+D2), 1.77 – 1.97 (m, 2H, SCH$_2$C*H*$_a$H$_b$, D1+D2), 2.07 – 2.20 (m, 2H, SCH$_2$CH$_2$CH$_a$*H*$_b$, D1+D2 + 2H, SCH$_2$C*H*$_a$H$_b$, D1+D2), 2.23 – 2.35 (m, 1H, C*H*CH$_3$, D1), 2.42 – 2.52 (m, 1H, C*H*CH$_3$, D2), 2.77 – 2.87 (m, 2H, SC*H*$_2$, D1 + 2H, SCH$_2$, D2), 3.34 – 3.44 (m, 1H, COC*H*CO, D2 + 1H, SC*H*, D2), 3.46 – 3.52 (m, 1H, SC*H*, D1), 3.66 (d, *J* = 5.2 Hz, 1H, COC*H*CO, D1), 4.07 – 4.19 (m, 4H, 2 × OC*H*$_2$, D1 + 4H, 2 × OC*H*$_2$, D2). **13C NMR** (101 MHz, CDCl$_3$) δ 14.0 (OCH$_2$*C*H$_3$, D1), 14.1 (OCH$_2$*C*H$_3$, D1), 14.1 (OCH$_2$*C*H$_3$, D2), 14.2 (OCH$_2$*C*H$_3$, D2), 14.7 (*C*H$_3$CH, D1), 14.9 (*C*H$_3$CH, D1), 31.0 (SCH$_2$*C*H$_2$, D1), 31.2 (SCH$_2$*C*H$_2$, D1), 32.3 (S*C*H$_2$, D1), 32.3 (S*C*H$_2$, D2), 34.5 (SCH$_2$CH$_2$*C*H$_2$, D1), 35.1 (SCH$_2$CH$_2$*C*H$_2$, D2), 38.9 (*C*HCH$_3$, D1), 39.9 (*C*HCH$_3$, D2), 52.5 (S*C*H, D1), 52.8 (S*C*H, D2), 56.1 (CO*C*HCO, D1), 56.4 (CO*C*HCO, D2), 61.1 (CO*C*H$_2$, D1), 61.3 (CO*C*H$_2$, D1), 61.3 (CO*C*H$_2$, D2), 61.42 (CO*C*H$_2$, D2), 168.4

(2 × *C*O), 168.8 (*C*O), 169.2 (*C*O). **v_{max}** (thin film/cm^{-1}): 2918, 2849, 1731, 1463, 1370, 1176, 1030, 445, 427. **HRMS** $C_{13}H_{22}O_4NaS$ (M+Na$^+$) predicted 297.1128, Found 297.1131.

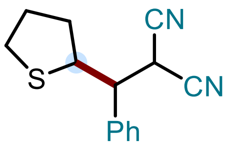

7y

2-(phenyl(tetrahydrothiophen-2-yl)methyl)malononitrile (7y)

7y was prepared according to general method a, using diethyl 2-ethylidenemalonate (18.3 µL, 0.1 mmol) and tetrahydrothiophene (88.1 µL, 1.0 mmol). The crude product was purified using column chromatography (20% EtOAc: Hexane) to give two separable diastereomers as off-white oils (16.9 mg, 0.070 mmol, 70%, dr 1:1).

D1 - ^{1}H NMR (400 MHz, $CDCl_3$) δ 1.56 – 1.67 (m, 1H, $SCH_2CH_2CH_aH_b$), 1.85 – 2.07 (m, 3H, SCH_2CH_2 + $SCH_2CH_2CH_aH_b$), 2.94 – 3.07 (m, 3H, SCHC*H* + SCH_2), 3.91 – 4.01 (m, 1H, SC*H*), 4.60 (d, *J* = 4.2 Hz, 1H, C*H*$(CN)_2$), 7.35 – 7.48 (m, 5H, Ar C*H*). **^{13}C NMR** (101 MHz, $CDCl_3$) δ 29.7 (*C*H$(CN)_2$), 29.7 (SCH_2CH_2), 33.2 (SCH_2), 35.7 ($SCH_2CH_2CH_2$), 50.0 (S*C*H), 54.5 (SCH*C*H), 111.4 (*C*N), 112.0 (*C*N), 128.3 (Ar *C*H), 129.3 (Ar *C*H), 129.3 (Ar *C*H), 135.6 (Ar *C*). **v_{max}** (thin film/cm^{-1}): 2923, 2855, 1494, 1454, 1050, 969, 790, 705, 544. **HRMS** $C_{14}H_{13}N_2S$ (M+H$^+$) predicted 241.0805, Found 241.0792.

D2 - ^{1}H NMR (400 MHz, $CDCl_3$) δ 1.63 – 1.74 (m, 1H, $SCHCH_aH_b$), 1.89 – 2.11 (m, 2H, SCH_2CH_2), 2.31 – 2.43 (m, 1H, $SCHCH_aH_b$), 2.70 – 2.90 (m, 2H, SCH_2), 3.27 – 3.37 (m, 1H, SCHCH), 4.08 – 4.15 (m, 1H, SC*H*), 4.17 (d, *J* = 6.1 Hz, 1H, C*H*$(CN)_2$), 7.40 (s, 5H, Ar C*H*). **^{13}C NMR** (101 MHz, $CDCl_3$) δ 28.7 (*C*H$(CN)_2$), 31.2 (SCH_2CH_2), 32.5 (SCH_2), 35.5 (SCHCH_2), 50.3 (S*C*H), 52.0 (SCH*C*H), 111.5 (*C*N), 111.6 (*C*N), 128.3 (Ar *C*H), 129.2 (Ar *C*H), 129.3 (Ar *C*H), 136.2 (Ar *C*). **v_{max}** (thin film/cm^{-1}): 2924, 2858, 1496, 1455, 1048, 757, 701, 575. **HRMS** $C_{14}H_{13}N_2S$ (M+H$^+$) predicted 241.0805, Found 241.0792

The data are in accordance with the literature. [2]

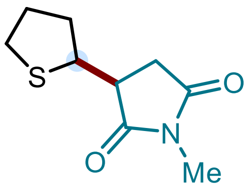

7z

1-methyl-3-(tetrahydrothiophen-2-yl)pyrrolidine-2,5-dione (7z)

7z was prepared according to general method a, using 1-methyl-1*H*-pyrrole-2,5-dione (8.5 µL, 0.1 mmol) and tetrahydrothiophene (88.1 µL, 1.0 mmol). The crude product was purified using column chromatography (20% EtOAc: Hexane) to give an off-white oil as a mixture of inseparable diastereomers (12.1 mg, 0.061 mmol, 61%, dr 1:2.3). **^{1}H NMR** (400 MHz, $CDCl_3$) δ 1.57 – 1.75 (m, 3.3H, SCHCH_aH$_b$, D1+D2), 1.75 – 1.97 (m, 3.3H, SCH_2CH_aH$_b$, D1+D2), 2.00 – 2.24 (m, 4.3H, $SCH_2CH_a$$H_b$, D1+D2 + SCHCH$_a$$H_b$, D1), 2.29 (m, 2.3H, SCHCH$_a$$H_b$, D2), 2.54 (dd, *J* = 18.6, 4.5 Hz, 1H, COCH_aH$_b$, D1), 2.72 (d, *J* = 2.4 Hz, 2.3H, COCH_aH$_b$, D2), 2.74 (s, 2.3H, COCH$_a$$H_b$, D2), 2.79 – 2.92 (m, 7.6H, SCH_2, D1+D2, COCH$_a$$H_b$, D1), 2.97 (s, 6.9H, NC$H_3$, D2), 2.98 (s, 3H, NC$H_3$, D1), 3.03 – 3.09 (m, 2.3H, SCHC*H*, D2), 3.13 (m, 1H, SCHC*H*, D1), 3.81 (m, 1H, SC*H*, D1), 3.98 (m, 2.3H, SC*H*, D2). **^{13}C NMR** (101 MHz, $CDCl_3$) δ 23.7 (NCH_3, D1), 23.8 (NCH_3, D2), 29.9 ($SCH_2$$CH_2$, D1), 29.9 ($SCH_2$$CH_2$, D2), 30.5 (CO$CH_2$, D2), 31.9 (SCH$CH_2$, D1), 32.0 (CO$CH_2$, D1), 32.0 (S$CH_2$, D2), 32.0 (S$CH_2$, D1), 34.6 (SCH$CH_2$, D2), 42.8 (SCH*C*H, D1), 43.7 (SCH*C*H, D2), 47.8 (S*C*H, D2), 47.9 (S*C*H, D1), 175.2 (*C*O, D1), 175.4 (*C*O, D2), 176.9 (*C*O, D1), 177.1 (*C*O, D2). **v_{max}** (thin film/cm^{-1}): 2932, 2863, 1774, 1692, 1434, 1382, 1280, 1122, 1016, 977, 949, 786, 702, 649, 603. **HRMS** $C_9H_{13}NO_2SNa$ ($M+Na^+$), predicted 222.0558, found 222.0559

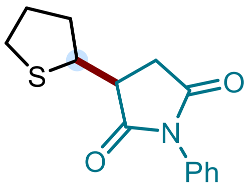

7aa

1-phenyl-3-(tetrahydrothiophen-2-yl)pyrrolidine-2,5-dione (7aa)

7aa was prepared according to general method a, using 1-phenyl-1*H*-pyrrole-2,5-dione (17.3 µL, 0.1 mmol) and tetrahydrothiophene (88.1 µL, 1.0 mmol). The crude product was purified using column

chromatography (20% EtOAc: Hexane) to give off-white oils as separable diastereomers (15.9 mg, 0.060 mmol, 62%, dr 2:1).

D1 ^{1}H NMR (400 MHz, $CDCl_3$) δ 1.51 – 1.65 (m, 1H, SCHCH_aH_b), 1.81 – 1.94 (m, 1H, SCH_2CH_aH_b), 2.09 – 2.18 (m, 1H, $SCH_2CH_a$$H_b$), 2.22 – 2.33 (m, 1H, SCH$CH_a$$H_b$), 2.76 – 2.93 (m, 4H, COCH_2 + SCH_2), 3.20 (dt, *J* = 8.4, 5.0 Hz, 1H, COC*H*), 4.00 – 4.11 (m, 1H, SC*H*), 7.15 – 7.24 (m, 2H, Ar C*H*), 7.28 – 7.36 (m, 1H, Ar C*H*), 7.36 – 7.45 (m, 2H, Ar C*H*). **^{13}C NMR** (101 MHz, $CDCl_3$) δ 31.0 (SCH_2CH_2), 31.5 (SCH_2), 33.2 (COCH_2), 35.6 (SCHCH_2), 44.6 (CO*C*H), 49.1 (S*C*H), 126.6 (Ar *C*H), 128.7 (Ar *C*H), 129.2 (Ar *C*H), 131.8 (Ar *C*), 175.4 (*C*O), 177.2 (*C*O). **v_{max}** (thin film/cm^{-1}): 2930, 2860, 1774, 1697, 1499, 1436, 1383, 1281, 1184, 1123, 949, 786, 759, 699, 648. **HRMS** $C_{14}H_{15}O_2NSNa$ (M+Na$^+$) predicted 284.0716, found 284.0709.

D2 ^{1}H NMR (400 MHz, $CDCl_3$) δ 1.82 – 1.92 (m, 2H, SCH_2CH_aH_b + SCHCH_aH_b), 2.17 – 2.32 (m, 2H, $SCH_2CH_a$$H_b$ + SCH$CH_a$$H_b$), 2.74 (dd, *J* = 18.6, 4.4 Hz, 1H, COCH_aH_b), 2.86 – 2.96 (m, 2H, SCH_2), 3.04 (dd, *J* = 18.7, 9.3 Hz, 1H, CO$CH_a$$H_b$), 3.28 – 3.37 (m, 1H, COC*H*), 3.89 – 3.99 (m, 1H, SC*H*), 7.26 – 7.32 (m, 2H. Ar C*H*), 7.37 – 7.42 (m, 1H, Ar C*H*), 7.44 – 7.52 (m, 2H, Ar C*H*). **^{13}C NMR** (101 MHz, $CDCl_3$) δ 31.1 (SCHCH_2), 32.8 (SCH_2CH_2), 33.1 (SCH_2), 33.5 (COCH_2), 43.7 (CO*C*H), 49.5 (S*C*H), 126.6 (Ar *C*H), 128.7 (Ar *C*H), 129.2 (Ar *C*H), 131.8 (Ar C), 175.2 (*C*O), 176.9 (*C*O). **v_{max}** (thin film/cm^{-1}): 2930, 2850, 1768, 1700, 1499, 1440, 1288, 1127, 787, 751, 650. **HRMS** $C_{14}H_{15}O_2NSNa$ (M+Na$^+$) predicted 284.0716, found 284.0710.

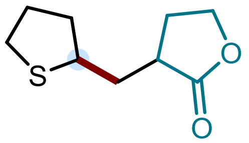

7ab

3-((tetrahydrothiophen-2-yl)methyl)dihydrofuran-2(3*H*)-one (7ab)

7ab was prepared according to general method a, using α-methylene-γ-butyrolactone (8.8 μL, 0.1 mmol) and tetrahydrothiophene (88.1 μL, 1.0 mmol). The crude product was purified using column chromatography (20% EtOAc: Hexane) to give an off-white oil as a mixture of inseparable diastereomers (14.1 mg, 76%, 0.076 mmol, dr 1:1). **^{1}H NMR** (400 MHz, $CDCl_3$) δ 1.53 – 1.81 (m, 4H,

SCHCH$_a$*H*$_b$CH, D1 + SCH$_2$CH$_2$C*H*$_2$, D2 + SCH$_2$CH$_2$CH$_a$*H*$_b$, D1), 1.86 – 2.20 (m, 9H, OCH$_2$CH$_a$*H*$_b$, D1 + SCH$_2$C*H*$_2$, D1+D2 + SCH$_2$CH$_2$C*H*$_a$H$_b$, D1 + OCH$_2$C*H*$_2$, D2 + SCHC*H*$_a$H$_b$CH, D2), 2.23 – 2.33 (m, 1H, SCHC*H*$_a$H$_b$CH, D1), 2.38 – 2.52 (m, 2H, OCH$_2$C*H*$_a$H$_b$, D1 + SCHCH$_a$*H*$_b$CH, D2), 2.52 – 2.62 (m, 1H, COC*H*, D1), 2.65 – 2.78 (m, 1H, COC*H*, D2), 2.81 – 2.97 (m, 4H, SCH$_2$, D1 + D2), 3.32 – 3.45 (m, 1H, SC*H*, D1), 3.53 – 3.63 (m, 1H, SC*H*, D2), 4.13 – 4.23 (m, 2H, OC*H*$_2$, D2), 4.29 – 4.40 (m, 2H, OC*H*$_2$, D1). **^{13}C NMR** (101 MHz, CDCl$_3$) δ 28.8 (OCH$_2$CH$_2$, D2), 29.7 (OCH$_2$CH$_2$, D1), 30.0 (SCH$_2$*C*H$_2$, D2), 30.00 (SCH$_2$*C*H$_2$, D1), 32.2 (SCH$_2$, D2), 32.5 (SCH$_2$, D1), 37.5 (SCH$_2$CH$_2$*C*H$_2$, D2), 37.6 (SCH$_2$CH$_2$*C*H$_2$, D1), 38.1 (SCH*C*H$_2$CH, D2), 38.5 (SCH*C*H$_2$CH, D1), 38.9 (CO*C*H, D2), 39.1 (CO*C*H, D1), 46.8 (S*C*H, D2), 47.3 (S*C*H, D1), 66.5 (O*C*H$_2$, D2), 66.5 (O*C*H$_2$, D1) 179.0 (*C*O, D2), 179.1 (*C*O, D1). **v_{max}** (thin film/cm^{-1}): 2924, 2856, 1765, 1441, 1373, 1253, 1150, 2011, 708, 436. **HRMS** $C_9H_{15}O_2S$ (M+H$^+$) predicted 187.0787, found 187.0788.

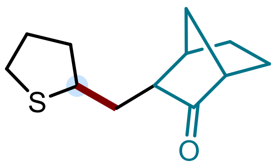

7ac

3-((tetrahydrothiophen-2-yl)methyl)bicyclo[2.2.1]heptan-2-one (7ac)

7ac was prepared according to general method a, using 3-methylenebicyclo[2.2.1]heptan-2-one (12.2 μL, 0.1 mmol) and tetrahydrothiophene (88.1 μL, 1.0 mmol). The crude product was purified using column chromatography (20% EtOAc: Hexane) to give an off-white oil as a mixture of inseparable diastereomers (15.5 mg, 0.074 mmol, 74%, dr 1:1). **^{1}H NMR** (400 MHz, CDCl$_3$) δ 1.32 – 1.42 (m, 2H, SCHCH$_2$CHCHCH$_a$*H*$_b$CH$_2$, D1+D2), 1.43 – 1.63 (m, 10H, SHCH$_2$CHCOCHCH$_a$*H*$_b$CH$_2$, D1+D2 + SCHC*H*$_2$CH, D1+D2 + SCHCH$_2$CHCHCH$_a$*H*$_b$CH, D1+D2, SCH$_2$CH$_2$CH$_a$*H*$_b$, D1+D2), 1.65 – 1.72 (m, 2H, SHCH$_2$CHCOCHC*H*$_a$H$_b$CH$_2$, D1+D2), 1.73 – 2.14 (m, 11H, SCH$_2$C*H*$_2$, D1+D2 + SCH$_2$CH$_2$C*H*$_a$H$_b$, D1+D2 + SCHCH$_2$CHC*H*, D2 + SCHCH$_2$CHCHC*H*$_a$H$_b$CH$_2$, D1+D2 + SCHCH$_2$CHCHC*H*$_a$H$_b$CH, D1+D2), 2.20 (dt, *J* = 9.9, 4.5 Hz, 1H, SCHCH$_2$CHC*H*, D1), 2.56 – 2.64 (m, 3H, SCHCH$_2$C*H*, D1 + SCHCH$_2$CHCOC*H*, D1+D2), 2.64 – 2.70 (m, 1H, SCHCH$_2$C*H*, D2), 2.77 – 2.94 (m, 4H, SC*H*$_2$, D1+D2),

3.31 – 3.42 (m, 1H, SC*H*, D2), 3.43 – 3.54 (m, 1H, SC*H*, D1). **^{13}C NMR** (101 MHz, $CDCl_3$) δ 21.4 (SCH*C*H_2CH, D1), 21.6 (SCH*C*H_2CH, D2), 25.3 (SCHCH_2CHCH*C*$H_2$$CH_2$, D1), 25.4 (SCH$CH_2$CHCH*C*$H_2$$CH_2$, D2), 30.1 ($SCH_2$*C*$H_2$, D1+D2), 32.1 (S*C*$H_2$, D1), 32.3 (S*C*$H_2$, D2), 34.0 ($SCH_2CH_2$*C*$H_2$, D1), 34.4 ($SCH_2CH_2$*C*$H_2$, D2), 37.1 (SCH$CH_2$CHCOCH*C*$H_2$$CH_2$, D1), 37.1 (SCH$CH_2$CHCOCH*C*$H_2$$CH_2$, D2), 37.3 (SCH$CH_2$CHCH*C*$H_2$CH, D1), 37.6 (SCH$CH_2$CHCH*C*$H_2$CH, D2), 38.1 (SCH$CH_2$*C*H, D1), 39.2 (SCH$CH_2$*C*H, D2), 47.2 (S*C*H, D1), 48.1 (S*C*H, D2), 50.4 (SCHCH_2CHCO*C*H, D1), 50.5 (SCHCH_2CHCO*C*H, D2), 53.2 (SCHCH_2CH*C*H, D1), 53.4 (SCHCH_2CH*C*H, D2), 219.5 (*C*O, D1), 219.5 (*C*O, D2). **v_{max}** (thin film/cm^{-1}): 1921, 2852, 1740, 1702, 1458, 1440, 1378, 1264, 1179, 739, 704. **HRMS** $C_{12}H_{19}OS$ (M+H$^+$) predicted 211.1151, found 211.1148.

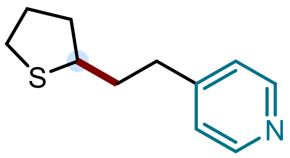

7ad

4-(2-(tetrahydrothiophen-2-yl)ethyl)pyridine (7ad)

7ad was prepared according to general method a, using 4-vinyl pyridine (10.8 µL, 0.1 mmol) and tetrahydrothiophene (88.1 µL, 1.0 mmol). The crude product was purified using column chromatography (20% EtOAc: Hexane) to afford an off-white oil (6.7 mg, 0.035 mmol, 35%). **^{1}H NMR** (400 MHz, $CDCl_3$) δ 1.55 – 1.62 (m, 1H, SCH_2CH_2C*H_a*H_b), 1.74 – 2.00 (m, 3H, SCH_2CH_a*H_b* + SCHC*H_2*CH_2Ar), 2.04 – 2.19 (m, 2H, SCH_2C*H_a*H_b + $SCH_2CH_2CH_a$*H_b*), 2.58 – 2.68 (m, 1H, CH_a*H_b*Ar), 2.69 – 2.80 (m, 1H, C*H_a*H_bAr), 2.82 – 2.96 (m, 2H, SC*H_2*), 3.25 – 3.36 (m, 1H, SC*H*), 7.09 – 7.14 (m, 2H, Ar C*H*), 8.49 (d, *J* = 5.0 Hz, 2H, Ar C*H*). **^{13}C NMR** (101 MHz, $CDCl_3$) δ 30.3 (SCH_2*C*H_2)), 32.3 (S*C*H_2), 34.6 (*C*H_2Ar), 37.4 (SCH_2CH_2*C*H_2), 38.3 (*C*$H_2$$CH_2$Ar), 48.4 (S*C*H), 123.9 (Ar *C*H), 149.8 (Ar *C*H), 150.7 (Ar *C*). **v_{max}** (thin film/cm^{-1}): 2917, 2849, 2034, 1602, 1461, 1414, 1376, 1301, 1050, 1013, 806, 720. **HRMS** $C_{11}H_{16}NS$ (M+H$^+$), predicted 194.0998, found 194.0998.

Possible origin of diastereocontrol in couplings of substituted tetrahydrothiopyran substrates

We propose that the diastereoselectivity of the radical couplings is roughly in line with the A value of the 4-substituent on the thiopyran ring. We believe that the coupling of these substrates proceeds via the formation and reaction of an anomerically-stabilized, nucleophilic axial radical alpha to sulfur. The preference of the substituent X to sit equatorial therefore results in different degrees of selectivity for the *anti* products. The exception to this trend is the 'OH' bearing substrate that gives **7i** with very high diastereocontrol. In this case, the hydroxyl may interact with the partially positively charged sulfur thus giving rise to even greater levels of *anti*-diastereoselectivity.

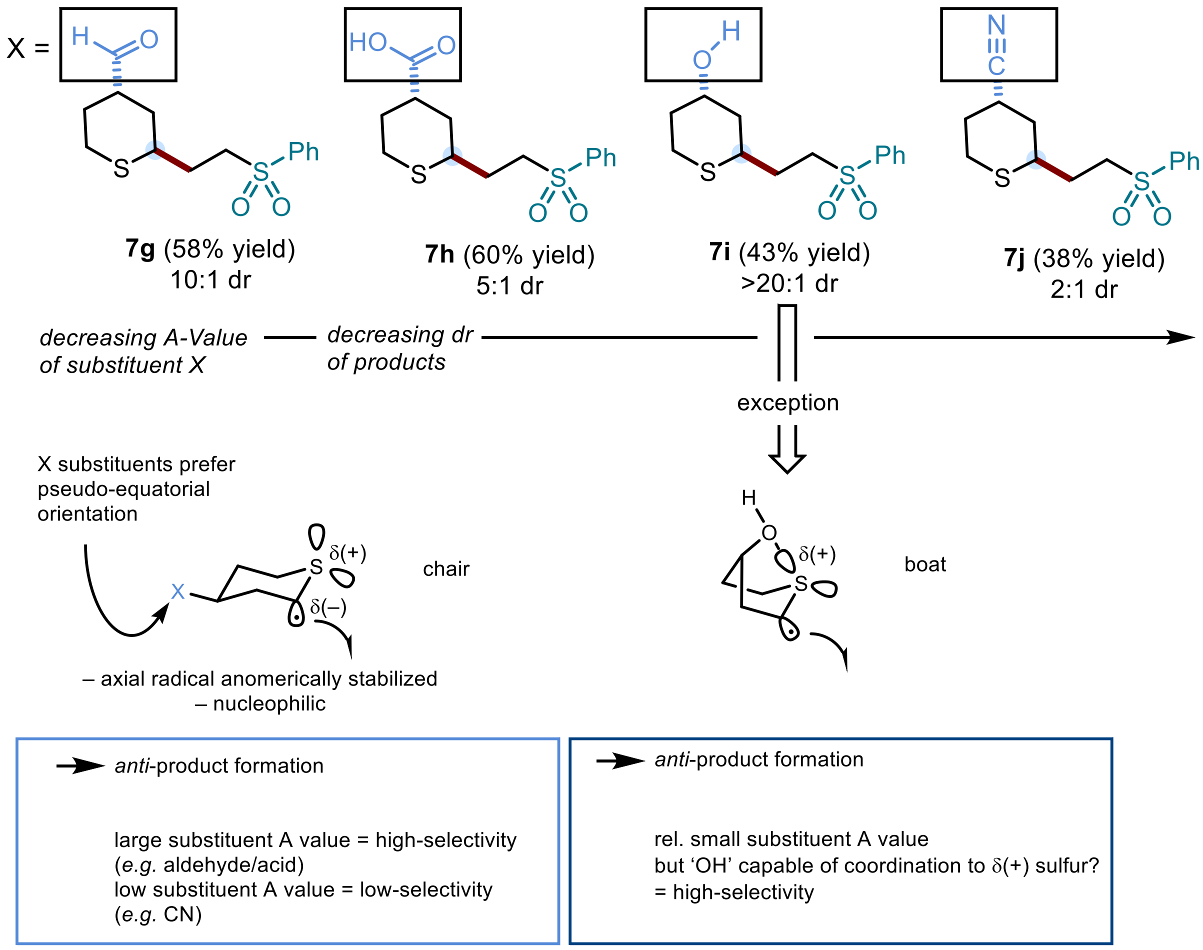

Methionine Analogue Scope

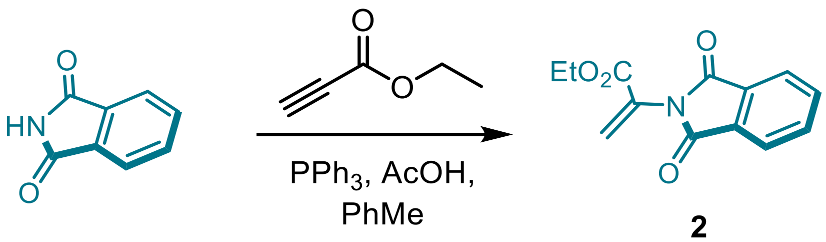

2

Ethyl 2-(1,3-dioxoisoindolin-2-yl)acrylate (2)

To a solution of phthalimide (7.36 g, 50 mmol), triphenylphosphine (1.31 g, 5 mmol), and sodium acetate (2.05 g, 25 mmol) in toluene (500 mL) was added glacial acetic acid (1.43 mL, 20 mmol), followed by ethyl propiolate (6.08 mL, 60 mmol), and the reaction mixture was stirred for 24 hours until completion as shown by TLC. The reaction mixture was then partitioned between water and ethyl acetate, extracted with ethyl acetate (3 × 300 mL), washed with brine (3 × 100 mL), dried over magnesium sulfate and evaporated under reduced pressure to yield a white powder. The crude product was recrystallized from petroleum ether: ethyl acetate (1:1) to yield colourless crystals (6.65 g, 27.0 mmol, 54%). **^{1}H NMR** (500 MHz, $CDCl_3$) δ 1.30 (t, *J* = 7.1 Hz, 3H, C*H*$_3$), 4.28 (q, *J* = 7.1 Hz, 2H, C*H*$_2$CH$_3$), 5.98 (s, 1H, CC*H*$_a$H$_b$), 6.68 (s, 1H, CCH$_a$*H*$_b$), 7.78 (dd, *J* = 5.5, 3.0 Hz, 2H, Ar C*H*), 7.92 (dd, *J* = 5.4, 3.1 Hz, 2H, Ar C*H*). **^{13}C NMR** (126 MHz, $CDCl_3$) δ 14.1 (*C*H$_3$), 62.1 (*C*H$_2$CH$_3$), 123.9 (C*C*H$_2$), 127.8 (Ar *C*H), 129.4 (*C*CH$_2$), 131.9 (Ar *C*), 134.5 (Ar *C*H), 162.2 (*C*O), 166.5 (*C*O). **HRMS** $C_{13}H_{11}O_4NNa$ (M+Na$^+$) predicted 268.0580, found 268.0587.

The data are in accordance with the literature.[3]

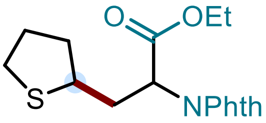

8a

Ethyl 2-(1,3-dioxoisoindolin-2-yl)-3-(tetrahydrothiophen-2-yl)propanoate (8a)

8a was prepared according to general method a, using ethyl 2-(1,3-dioxoisoindolin-2-yl)acrylate (24.5 mg, 0.1 mmol) and tetrahydrothiophene (88.1 μL, 1.0 mmol). The crude product was purified using column chromatography (20% EtOAc: Hexane) to give an off-white oil (25.6 mg, 0.077 mmol, 77%, dr 1.6:1). **^{1}H NMR** (400 MHz, $CDCl_3$) δ 1.22 (t, *J* = 7.1 Hz, 7.8H, CH_3, D1 + D2), 1.55 – 1.65 (m, 2.6H, $SCH_2CH_2CH_aH_b$, D1+D2), 1.74 – 1.85 (m, 1.6H, $SCH_2CH_aH_b$, D1), 1.85 – 1.96 (m, 1H, $SCH_2CH_aH_b$, D2), 1.98 – 2.13 (m, 3.6H, $SCH_2CH_2CH_aH_b$, D2 + $SCH_2CH_aH_b$, D1 + $SCH_2CH_aH_b$, D2), 2.21 (ddd, *J* = 11.4, 6.6, 5.5 Hz, 1.6H, $SCH_2CH_2CH_aH_b$, D1), 2.25 – 2.35 (m, 1H, $NCHCH_aH_b$, D2), 2.40 – 2.53 (m, 1H, $NCHCH_aH_b$, D2), 2.62 (m, 1.6H, $NCHCH_aH_b$, D1), 2.68 – 2.75 (m, 1.6H, $NCHCH_aH_b$, D1) 2.75 – 2.96 (m, 5.2H, SCH_2, D1+D2), 3.20 (p, *J* = 5.9 Hz, 1H, SC*H*, D2), 3.33 (p, *J* = 7.1 Hz, 1.6H, SC*H*, D1), 4.15 – 4.24 (m, 5.2H, OCH_2, D1 + D2), 4.90 (dd, *J* = 10.6, 4.6 Hz, 1.6H, NC*H*, D1), 5.03 (dd, *J* = 11.7, 4.0 Hz, 1H, NC*H*, D2), 7.71 – 7.77 (m, 5.2H, Ar C*H*, D1 + D2), 7.85 – 7.91 (m, 5.2H, Ar C*H*, D1 + D2). **^{13}C NMR** (101 MHz, $CDCl_3$) δ 13.1 (CH_2CH_3, D1), 13.1 (CH_2CH_3, D1), 28.7 (SCH_2CH_2, D1), 28.9 (SCH_2CH_2, D2), 31.3 (SCH_2, D1), 31.5 (SCH_2, D2), 35.2 ($NCHCH_2$, D1), 35.3 ($NCHCH_2$, D2), 36.3 ($SCH_2CH_2CH_2$, D2), 36.3 ($SCH_2CH_2CH_2$, D1), 44.5 (S*C*H, D2), 45.0 (S*C*H, D1), 50.6 (NC*H*, D1), 51.1 (N*C*H, D2), 60.9 (OCH_2, D1), 61.0 (OCH_2, D2), 122.6 (Ar *C*H, D1), 122.6 (Ar *C*H, D2) 130.8 (Ar C, D2), 130.9, (Ar *C*, D1), 133.1 (Ar *C*H, D1), 133.2 (Ar *C*H, D2), 166.6 (N*C*O, D1), 166.7 (N*C*O, D2), 167.84 (*C*O, D1), 168.14 (*C*O, D2). **v_{max}** (thin film/cm^{-1}): 2923, 2853, 1776, 1715, 1466, 1385, 1251, 1098, 1022, 875, 721, 530. **HRMS** $C_{17}H_{20}O_4NS$ ($M+H^+$) predicted 334.1108, found 334.1106.

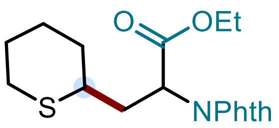

8b

Ethyl 2-(1,3-dioxoisoindolin-2-yl)-3-(tetrahydro-2*H*-thiopyran-2-yl)propanoate (8b)

8b was prepared according to general method a, using ethyl 2-(1,3-dioxoisoindolin-2-yl)acrylate (24.5 mg, 0.1 mmol) and tetrahydrothiopyran (102.2 mg, 101.1 µL, 1 mmol). The crude product was purified using column chromatography (20% EtOAc: Hexane) to give an off-white oil (27.8 mg, 0.080 mmol, 80%, dr 1:1.6). **^{1}H NMR** (500 MHz, $CDCl_3$) δ 1.19 – 1.24 (m, 7.8H, OCH_2CH_3, D1+D2), 1.28 – 1.39 (m, 2.6H, $SCH_2CH_2CH_aH_b$, D1+D2), 1.42 – 1.51 (m, 1.6H, $SCH_2CH_2CH_2CH_aH_b$, D2), 1.51 – 1.69 (m, 3H, SCH_2CH_2, D1 + $SCH_2CH_2CH_2CH_aH_b$, D1), 1.75 – 1.89 (m, 4.6H, SCH_2, D1 + $SCH_2CH_2CH_aH_b$, D1+D2), 1.89 – 1.96 (m, 1H, $SCH_2CH_2CH_2CH_aH_b$, D1), 2.10 (ddt, *J* = 13.3, 5.7, 3.0 Hz, 1.6H, $SCH_2CH_2CH_2$-CH_aH_b, D2), 2.33 (ddd, *J* = 14.6, 9.6, 7.2 Hz, 1.6H, $NCHCH_aH_b$, D2), 2.38 – 2.65 (m, 11H, SC*H*, D1 + $NCHCH_2$, D1 + SCH_2, D2 +SCH_2CH_2, D2 + $NCHCH_aH_b$, D2), 2.70 – 2.80 (m, 1.6H, SC*H*, D2), 4.12 – 4.26 (m, 5.2H, OCH_2, D1+D2), 5.03 (dd, *J* = 9.6, 5.1 Hz, 1.6H, NC*H*, D2), 5.16 – 5.22 (m, 1H, NC*H*, D1), 7.75 (dt, *J* = 5.5, 3.2 Hz, 5.2H, Ar C*H*, D1+D2), 7.88 (dt, *J* = 5.5, 3.2 Hz, 5.2H, Ar C*H*, D1+D2). **^{13}C NMR** (126 MHz, $CDCl_3$) δ 14.0 (OCH_2CH_3, D2), 14.0 (OCH_2CH_3, D1), 25.1 ($SCH_2CH_2CH_2$, D1), 25.4 ($SCH_2CH_2CH_2$, D2), 27.0 (SCH_2CH_2, D1), 27.0 (SCH_2, D1), 28.0 (SCH_2CH_2, D2), 28.7 (SCH_2, D2), 33.7 ($SCH_2CH_2CH_2CH_2$, D2), 34.20 ($NCHCH_2$, D1), 34.3 ($SCH_2CH_2CH_2CH_2$, D1), 34.9 ($NCHCH_2$, D2), 38.6 (S*C*H, D1), 39.4 (S*C*H, D2), 49.8 (N*C*H, D2), 50.2 (N*C*H, D1), 61.9 (OCH_2, D1+D2), 123.5 (Ar *C*H, D1+D2), 131.8 (Ar *C*, D1), 131.8 (Ar *C*, D2), 134.0 (Ar *C*H, D2), 134.1 (Ar *C*H, D1), 167.5 (*C*O, D2), 167.7 (*C*O, D1), 169.0 (*C*O, D2), 169.3 (*C*O, D1). **v_{max}** (thin film/cm^{-1}): 2927, 2846, 1776, 1740, 1714, 1468, 1440, 1386, 1233, 1106, 1024, 874, 795, 721, 531. **HRMS** $C_{18}H_{21}O_4NSNa$ ($M+Na^+$) predicted 370.1084, found 370.1093.

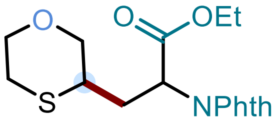

8c

Ethyl 2-(1,3-dioxoisoindolin-2-yl)-3-(1,4-oxathian-3-yl)propanoate (8c)

8c was prepared according to general method a, using ethyl 2-(1,3-dioxoisoindolin-2-yl)acrylate (24.5 mg, 0.1 mmol) and 1,4-oxathiane (104.1 mg, 93.5.1 µL, 1 mmol). The crude product was purified using column chromatography (40% EtOAc: Hexane) to give an off-white oil (22.6 mg, 65%, dr 1:1.5). **^{1}H NMR** (500 MHz, $CDCl_3$) δ 1.22 (q, *J* = 7.0 Hz, 7.5H, OCH_2CH_3, D1+D2), 2.34 (dt, *J* = 14.8, 8.7 Hz, 1.5H, $NCHCH_aH_b$, D2), 2.53 – 2.63 (m, 8.5H, SCH_aH_b, D1 + SC*H*, D1 + $NCHCH_2$, D1 + SCH_2, D2 + $NCHCH_aH_b$, D2), 2.71 (ddd, *J* = 13.9, 6.4, 2.7 Hz, 1H, SCH_aH_b, D1), 2.89 (dtd, *J* = 8.9, 6.4, 2.8 Hz, 1.5H, SC*H*, D2), 3.59 – 3.66 (m, 2.5H, $SCHCH_aH_bO$, D1+D2), 3.76 (ddd, *J* = 11.8, 7.3, 3.3 Hz, 1.5H, $SCH_2CH_aH_b$, D2), 3.83 (ddd, *J* = 11.7, 7.4, 2.7 Hz, 1H, $SCH_2CH_aH_b$, D1), 3.94 (dtd, *J* = 12.0, 6.5, 6.0, 3.2 Hz, 2.5H, $SCH_2CH_aH_b$, D1+D2), 3.98 (dd, *J* = 11.7, 2.3 Hz, 1H, $SCHCH_aH_bO$, D1), 4.09 (dd, *J* = 11.7, 2.8 Hz, 1.5H, $SCHCH_aH_bO$, D2), 4.15 – 4.26 (m, 5H, OCH_2, D1+D2), 5.04 (dd, *J* = 9.0, 5.9 Hz, 1.5H, NC*H*, D2), 5.20 (dd, *J* = 11.2, 3.8 Hz, 1H, NC*H*, D1), 7.71 – 7.78 (m, 5H, Ar C*H*, D1+D2), 7.84 – 7.92 (m, 5H, Ar C*H*, D1+D2). **^{13}C NMR** (126 MHz, $CDCl_3$) δ 14.1 (OCH_2CH_3, D2), 14.1 (OCH_2CH_3, D1), 25.4 (SCH_2, D1), 25.9 (SCH_2, D2), 30.2 ($NCHCH_2$, D1), 31.0 ($NCHCH_2$, D2), 36.0 (S*C*H, D1), 36.5 (S*C*H, D2), 50.0 (N*C*H, D2), 50.3 (N*C*H, D1), 62.1 (OCH_2, D2), 62.1 (OCH_2, D1), 68.6 (SCH_2CH_2, D2), 68.6 (SCH_2CH_2, D1), 73.0 ($SCHCH_2O$, D2), 73.5 ($SCHCH_2O$, D1), 123.7 (Ar *C*H, D1+D2), 131.8 (Ar *C*, D1), 131.9 (Ar *C*, D2), 134.2 (Ar *C*H, D2), 134.3 (Ar *C*H, D1), 167.5 (CO, D2), 167.8 (CO, D1), 168.8 (CO, D1), 169.2 (CO, D2). **v_{max}** (thin film/cm^{-1}): **HRMS:** $C_{17}H_{19}O_5NNaS$ ($M+Na^+$) predicted 372.0676, found 392.0877.

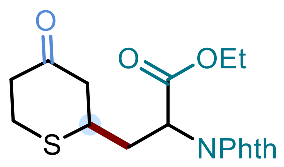

8d

Ethyl 2-(1,3-dioxoisoindolin-2-yl)-3-(4-oxotetrahydro-2*H*-thiopyran-2-yl)propanoate (8d)

8d was prepared according to general method a, using ethyl 2-(1,3-dioxoisoindolin-2-yl)acrylate (245 mg, 0.1 mmol) and tetrahydrothiopyran-4-one (116.8 mg, 1 mmol). The crude product was purified using column chromatography (40% EtOAc: Hexane) to give an off-white oil (18.1 mg, 0.050 mmol, 50%, dr 1:1.2). **^{1}H NMR** (500 MHz, $CDCl_3$) δ 1.19 – 1.23 (m, 6.6H, OCH_2CH_3, D1+D2), 2.33 (dt, *J* = 14.7, 8.6 Hz, 1.2H, $NCHCH_aH_b$, D2), 2.44 – 2.54 (m, 3.2H, $SCHCH_aH_bCO$, D1+D2 + $NCHCH_aH_b$, D1), 2.55 – 2.72 (m, 6.6H, SCH_2, D1+D2 + $NCHCH_aH_b$, D1+D2), 2.76 (dd, *J* = 13.8, 3.9 Hz, 1H, $SCHCH_aH_bCO$, D1), 2.80 – 2.96 (m, 4.6H, SCH_2CH_2, D2 + $SCHCH_aH_bCO$, D2 + $SCH_2CH_aH_b$, D1), 2.98 – 3.05 (m, 2H, $SCH_2CH_aH_b$, D1 + SC*H*, D1), 3.29 (tdd, *J* = 9.3, 5.9, 3.7 Hz, 1.2H, SC*H*, D2), 4.13 – 4.27 (m, 4.4H, OCH_2, D1+D2), 5.05 (dd, *J* = 8.6, 6.0 Hz, 1.2H, NC*H*, D2), 5.19 (dd, *J* = 11.5, 3.7 Hz, 1H, NC*H*, D1), 7.76 (dt, *J* = 5.7, 2.9 Hz, 4.4H, Ar C*H*, D1+D2), 7.84 – 7.99 (m, 4.4H, Ar C*H*, D1+D2). **^{13}C NMR** (126 MHz, $CDCl_3$) δ 14.1 ($OCHCH_2CH_3$, D2), 14.1 ($OCHCH_2CH_3$, D1), 27.4 (SCH_2CH_2, D1), 27.7 (SCH_2CH_2, D2), 33.8 ($NCHCH_2$, D1), 34.5 ($NCHCH_2$, D2), 41.2 (S*C*H, D1), 41.9 (S*C*H, D2), 43.3 (SCH_2, D2), 43.3 (SCH_2, D1), 49.9 (N*C*H, D2), 49.9 ($SCHCH_2CO$, D2), 50.1 ($SCHCH_2CO$, D1), 50.2 (N*C*H, D1), 62.2 (OCH_2, D2), 62.2 (OCH_2, D1), 123.7 (Ar *C*H, D2), 123.7 (Ar *C*H, D1), 131.8 (Ar *C*, D2), 131.8 (Ar *C*, D2), 134.3 (Ar *C*H, D2), 134.4 (Ar *C*H, D1), 167.5 (*C*O, D2), 167.7 (*C*O, D1), 168.6 (*C*O, D2), 168.8 (*C*O, D1), 207.6 (*C*O, D2), 207.7 (*C*O, D1). **v_{max}** (thin film/cm^{-1}): 2983, 2924, 1776, 1740, 1714, 1486, 1387, 1252, 1105, 1024, 875, 722, 530. **HRMS** $C_{18}H_{19}O_5NSNa$ ($M+Na^+$) predicted 384.0876, found 384.0887.

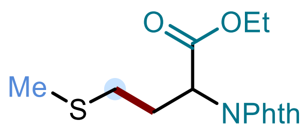

8e

Ethyl 2-(1,3-dioxoisoindolin-2-yl)-4-(methylthio)butanoate (8e)

8e was prepared according to general method a, using ethyl 2-(1,3-dioxoisoindolin-2-yl)acrylate (24.5 mg, 0.1 mmol) and dimethyl sulfide (62.1 µL, 1.0 mmol). The crude product was purified using column chromatography (20% EtOAc: Hexane) to give an off-white oil (15.3 mg, 0.050 mmol, 50%). **^{1}H NMR** (500 MHz, $CDCl_3$) δ 1.23 (t, *J* = 7.1 Hz, 3H, OCH_2CH_3), 2.08 (s, 3H, SCH_3), 2.43 – 2.61 (m, 4H, SCH_2CH_2), 4.21 (m, 2H, OCH_2), 5.07 (dd, *J* = 8.6, 5.7 Hz, 1H, COC*H*), 7.75 (m, 2H, Ar C*H*), 7.87 (m, 2H, Ar C*H*). **^{13}C NMR** (126 MHz, $CDCl_3$) δ 14.1 (OCH_2CH_3), 15.4 (SCH_3), 28.1 (SCH_2CH_2), 30.9 (SCH_2), 51.1 (COCH), 62.0 (OCH_2), 123.6 (Ar C*H*), 131.8 (Ar C), 134.6 (Ar C*H*), 167.7 (*C*O), 169.0 (*C*O). **HRMS** $C_{15}H_{17}O_4NSNa$ ($M+Na^+$) predicted 330.0770, found 330.0761.

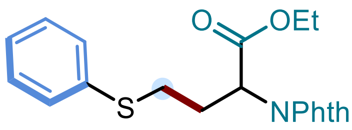

8f

Ethyl 2-(1,3-dioxoisoindolin-2-yl)-4-(phenylthio)butanoate (8f)

8f was prepared according to general method a, using ethyl 2-(1,3-dioxoisoindolin-2-yl)acrylate (24.5 mg, 0.1 mmol) and thioanisole (117 µL, 1.0 mmol). The crude product was purified using column chromatography (20% EtOAc: Hexane) to give an off-white oil (15.1 mg, 0.041 mmol, 41%). **^{1}H NMR** (400 MHz, $CDCl_3$) δ 1.21 (t, *J* = 7.1 Hz, 3H, CH_3CH_2O), 2.47 – 2.62 (m, 2H, $NCHCH_2$), 2.85 – 3.07 (m, 2H, SCH_2), 4.19 (m, 2H, OCH_2) , 5.10 (dd, *J* = 9.4, 5.6 Hz, 1H, COC*H*), 7.13 – 7.19 (m, 1H, Ar C*H*), 7.22 – 7.29 (m, 2H, Ar C*H*), 7.34 (dd, *J* = 7.5, 1.7 Hz, 2H, Ar C*H*), 7.73 – 7.78 (m, 2H, Ar C*H*), 7.87 (dd, *J* = 5.4, 3.1 Hz, 2H). **^{13}C NMR** (101 MHz, $CDCl_3$) δ 14.1 (OCH_2CH_3), 28.6 ($NCHCH_2$), 31.0 (SCH_2), 51.0 (COCH), 62.0 (OCH_2), 123.6 (Ar *C*H), 126.4 (Ar *C*H), 129.0 (Ar *C*H), 130.0 (Ar *C*H), 131.8 (Ar *C*), 134.3 (Ar *C*H), 135.4 (Ar *C*), 167.6 (*C*O), 168.9 (*C*O). **v_{max}** (thin film/cm^{-1}): 2923, 2853, 1776, 1716, 1468,

1440, 1386, 1238, 1101, 1025, 875, 722, 692, 530. **HRMS** $C_{20}H_{19}O_4NSNa$ (M+Na$^+$) predicted 392.0927, found 397.0916.

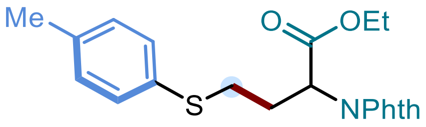

8g

Ethyl 2-(1,3-dioxoisoindolin-2-yl)-4-(*p*-tolylthio)butanoate (8g)

8g was prepared according to general method a, using ethyl 2-(1,3-dioxoisoindolin-2-yl)acrylate (24.5 mg, 0.1 mmol) and 4-methylthioanisole (134 µL, 1.0 mmol). The crude product was purified using column chromatography (20% EtOAc: Hexane) to give an off-white oil (17.2 mg, 0.045 mmol, 45%).^{1}**H NMR** (400 MHz, $CDCl_3$) δ 1.20 (t, *J* = 7.1 Hz, 3H, OCH_2CH_3), 2.30 (s, 3H, CH_3Ar), 2.43 – 2.59 (m, 2H, $NCHCH_2$), 2.83 – 2.98 (m, 2H, SCH_2), 4.18 (m, 2H, OCH_2), 5.10 (dd, *J* = 9.4, 5.5 Hz, 1H, COC*H*), 7.07 (d, *J* = 8.0 Hz, 2H, Ar C*H*), 7.25 (d, *J* = 7.4 Hz, 2H, Ar C*H*), 7.75 (dd, *J* = 5.5, 3.0 Hz, 2H, Ar C*H*), 7.87 (m, 2H, Ar C*H*). ^{13}**C NMR** (101 MHz, $CDCl_3$) δ 14.1 (OCH_2CH_3), 21.0 (CH_3Ar), 28.5 ($NCHCH_2$), 31.8 (SCH_2), 51.0 (CO*C*H), 62.0 (OCH_2), 123.6 (Ar *C*H), 129.8 (Ar *C*H), 130.9 (Ar *C*H), 131.5 (Ar *C*), 131.8 (Ar *C*), 134.3 (Ar *C*H), 136.7 (Ar *C*), 167.6 (*C*O), 168.9 (*C*O). $\mathbf{v_{max}}$ (thin film/cm^{-1}): 2980, 2920, 1777, 1742, 1716, 1493, 1468, 1386, 1238, 1090, 1026, 875, 805, 719. **HRMS** $C_{21}H_{21}O_4NS$ (M+H$^+$) predicted 383.1186, found 383.1171.

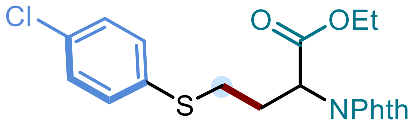

8h

Ethyl 4-((4-chlorophenyl)thio)-2-(1,3-dioxoisoindolin-2-yl)butanoate (8h)

8h was prepared according to general method a, using ethyl 2-(1,3-dioxoisoindolin-2-yl)acrylate (24.5 mg, 0.1 mmol) and 4-chlorothioanisole (160 µL, 1.0 mmol). The crude product was purified using column chromatography (20% EtOAc: Hexane) to give an off-white oil (12.9 mg, 0.032 mmol, 32%). ^{1}**H NMR** (400 MHz, $CDCl_3$) δ 1.21 (t, *J* = 7.1 Hz, 3H, OCH_2CH_3), 2.41 – 2.62 (m, 2H, $NCHCH_2$), 2.83 – 3.04 (m, 2H, SCH_2), 4.19 (m, 2H, OCH_2), 5.07 (dd, *J* = 9.4, 5.5 Hz, 1H, COC*H*), 7.20 – 7.27 (m, 4H, Ar C*H*), 7.71

– 7.78 (m, 2H, Ar C*H*), 7.87 (m, 2H, Ar C*H*). **^{13}C NMR** (101 MHz, $CDCl_3$) δ 14.1 (OCH_2CH_3), 28.5 (NCHCH_2), 31.3 (SCH_2), 50.9 (CO*C*H), 62.1 (OCH_2), 123.7 (Ar *C*H), 129.1 (Ar *C*H), 131.4 (Ar *C*H), 131.8 (Ar *C*), 132.5 (Ar *C*), 133.9 (Ar *C*), 134.3 (Ar *C*H), 167.6 (*C*O), 168.8 (*C*O). **v_{max}** (thin film/cm^{-1}): 2920, 1777, 1717, 1477, 1387, 1239, 1096, 1012, 816, 720. **HRMS** $C_{20}H_{19}O_4NSCl$ (M+H$^+$) predicted 404.0714, found 404.0718.

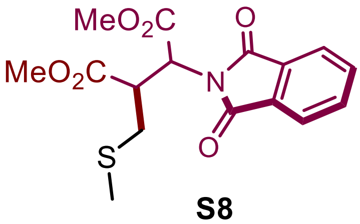

S8

Dimethyl 2-(1,3-dioxoisoindolin-2-yl)-3-((methylthio)methyl)succinate (S8)

S8 was prepared according to general method a, using S3 (28.9 mg, 0.1 mmol) and dimethyl sulfide (62.1 µL, 1.0 mmol). The crude product was purified using column chromatography (30% EtOAc: Hexane) to give an off-white oil as a mixture of inseparable diastereomers (14.7 mg, 42%, 0.042 mmol, dr 1:1.5). **^{1}H NMR** (400 MHz, $CDCl_3$) δ 2.04 (s, 3H, SCH_3, D1), 2.15 (s, 4.5H, SCH_3, D2), 2.62 – 2.67 (m, 2H, SCH_2, D1), 2.95 – 3.00 (m, 3H, SCH_2, D2), 3.62 (s, 4.5H, OCH_3, D2), 3.68 – 3.73 (m, 4H, OCH_3 + SCH_2C*H*), D1 3.73 – 3.79 (m, 6H, OCH_3 + SCH_2C*H*, D2), 3.81 (s, 3H, OCH_3, D1), 5.25 (d, *J* = 7.5 Hz, 1.5H, NC*H*, D2), 5.36 (d, *J* = 9.7 Hz, 1H, NC*H*, D1), 7.71 – 7.82 (m, 5H, Ar C*H*, D1+D2), 7.82 – 7.94 (m, 5H, Ar C*H*, D1+D2). **^{13}C NMR** (101 MHz, $CDCl_3$) δ 15.8 (SCH_3, D1+D2), 33.5 (SCH_2, D1), 33.6 (SCH_2, D2), 44.3 (SCH_2CH, D1), 45.5 (SCH_2CH, D2), 51.9 (N*C*H, D2), 52.3 (N*C*H, D1), 52.4 (OCH_3, D1+D2), 53.0 (OCH_3, D2), 53.1 (OCH_3, D1), 123.8 (Ar *C*H, D2), 123.9 (Ar *C*H, D1), 131.6 (Ar *C*, D2), 131.6 (Ar *C*, D1), 134.4 (Ar *C*H, D2), 134.5 (Ar *C*H, D1), 167.1 (*C*O), 167.4 (*C*O), 168.1 (*C*O), 168.4 (*C*O), 171.3 (*C*O), 172.5 (*C*O). **HRMS** $C_{16}H_{17}O_2N_5SNa$ (M+Na$^+$) predicted 374.0669, found 374.0681.

Product Derivatisation

General Method C: Deprotection to give unnatural amino acids

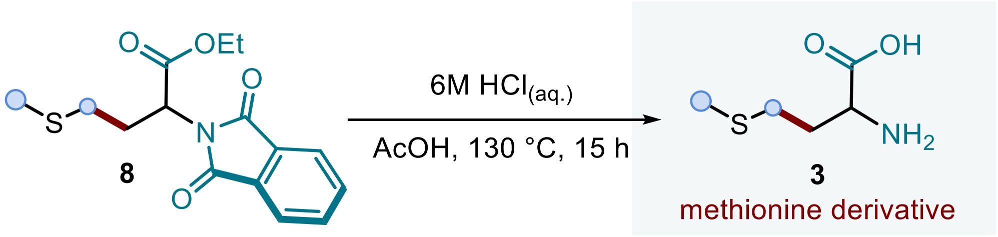

The protected amino acid (0.045 – 0.08 mmol) was placed in an oven dried microwave vial, dissolved in 6M aqueous HCl solution (5 mL) and glacial acetic acid (0.5 mL) and stirred at 130 °C for 16 h. Volatiles were then removed under reduced pressure, and the crude residue purified using reverse phase silica gel chromatography.

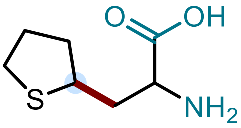

3a

2-amino-3-(tetrahydrothiophen-2-yl)propanoic acid (3a)

3a was prepared according to general method C, using **8a** (25.6 mg, 0.077 mmol), 6M HCl (5 mL) and AcOH (0.5 mL). The product was purified using reverse phase silica gel chromatography (H_2O) to give an off-white solid (12.4 mg, 92%, dr 1:1.6). ^{1}**H NMR** (400 MHz, D_2O) δ 1.63 – 1.76 (m, 2.6H, $SCH_2CH_2C\textit{H}_aH_b$, D1+D2), 1.89 – 2.25 (m, 11.4H, $NH_2CHC\textit{H}_aH_b$, D2 + $SCH_2CH_2CH_a\textit{H}_b$, D1+D2 + $NH_2CHC\textit{H}_2$, D1 +$SCH_2C\textit{H}_2$, D1+D2), 2.27 – 2.39 (m, 1.6H, $NH_2CHCH_a\textit{H}_b$, D2), 2.83 – 3.00 (m, 5.2H, $SC\textit{H}_2$, D1+D2), 3.47 – 3.62 (m, 2.6H, SC*H*, D1+D2), 3.88 – 4.07 (m, 2.6H, $NH_2C\textit{H}$, D1+D2). ^{13}**C NMR** (101 MHz, D_2O) δ 29.4 ($SCH_2CH_2\textit{C}H_2$, D2), 29.6 ($SCH_2CH_2\textit{C}H_2$, D1), 32.0 ($S\textit{C}H_2$, D1), 32.1 ($S\textit{C}H_2$, D2), 36.7 ($SCH_2\textit{C}H_2$, D1+D2), 37.7 ($NH_2CH\textit{C}H_2$, D1), 38.0 ($NH_2CH\textit{C}H_2$, D2), 44.4 (S*C*H, D1), 44.5 (S*C*H, D2), 53.1 ($NH_2\textit{C}H$, D1+D2), 172.4 (*C*O, D1+D2). **HRMS** $C_7H_{13}O_2SNNa$ ($M+Na^+$) predicted 198.0559, found 198.0559.

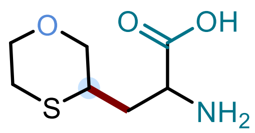

3c

2-amino-3-(1,4-oxathian-3-yl)propanoic acid (3c)

3c was prepared according to general method C, using **8c** (22.6 mg, 0.065 mmol), 6M HCl (5 mL) and AcOH (0.5 mL). The product was purified using reverse phase silica gel chromatography (H_2O) to give an off-white solid (11.2 mg, 0.058 mmol, 90%, dr 1:1.3). **^{1}H NMR** (400 MHz, D_2O) δ 2.04 (ddd, *J* = 15.3, 9.5, 4.4 Hz, 1H, $NH_2CHCH_aH_b$, D1), 2.11 – 2.16 (m, 2.6H, NH_2CHCH_2, D2), 2.31 (ddd, *J* = 15.2, 11.1, 3.9 Hz, 1H, $NH_2CHCH_aH_b$, D1), 2.58 – 2.71 (m, 2.3H, SCH_aH_b, D1+D2), 2.78 – 2.93 (m, 3.3H, SCH_aH_b, D1+D2 + SC*H*, D1), 3.00 (qd, *J* = 6.5, 2.7 Hz, 1.3H, SC*H*, D2), 3.68 – 3.76 (m, 2.3H, $SCHCH_aH_bO$, D1+D2), 3.84 – 4.02 (m, 6.9H, NH_2CH, D1+D2 + SCH_2CH_2, D1+D2), 4.08 (dt, *J* = 12.0, 2.9 Hz, 2.3H, $SCHCH_aH_bO$, D1+D2). **^{13}C NMR** (101 MHz, D_2O) δ 24.3 (SCH_2, D1), 24.4 (SCH_2, D2), 31.8 (NH_2CHCH_2, D1), 32.3 (NH_2CHCH_2, D2), 34.7 (SCH, D1), 35.1 (SCH, D2), 52.5 (NH_2CH, D1), 52.9 (NH_2CH, D2), 68.2 (SCH_2CH_2, D1), 68.2 (SCH_2CH_2, D2), 72.6 ($SCHCH_2O$, D1), 72.7 ($SCHCH_2O$, D2), 174.0 (*C*O, D2), 174.2 (*C*O, D1). **HRMS** $C_7H_{13}O_3SNNa$ ($M+Na^+$) predicted 214.0508, found 214.0509.

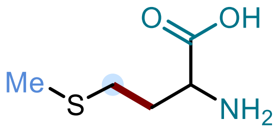

3e

DL-Methionine (3e)

3e was prepared according to general method C, using **8e** (15.3 mg, 0.050 mmol), 6M HCl (5 mL) and AcOH (6.4 mg, 0.5 mL). The product was purified using reverse-phase silica gel chromatography (H_2O) to give a white solid (6.0 mg, 0.044 mmol, 87%). **^{1}H NMR** (400 MHz, D_2O) δ 2.08 – 2.24 (m, 5H, SCH_3 + SCH_2CH_2), 2.64 (t, *J* = 7.5 Hz, 2H, SCH_2), 3.86 (dd, *J* = 7.1, 5.3 Hz, 1H, NH_2CH). **^{13}C NMR** (126 MHz, D_2O) δ 13.8 (SCH_3), 28.7 (SCH_2), 29.6 (SCH_2CH_2), 53.8 (NH_2CH), 174.1 (*C*O). **HRMS** $C_5H_{11}O_2SNNa$ ($M+Na^+$) predicted 172.0403, found 172.0401.

Data in agreement with reported values.[4]

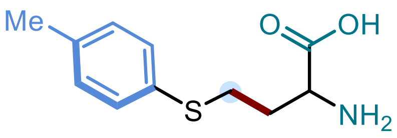

3g

(*p*-tolyl)homocysteine (3g)

3g was prepared according to general method C, using **8g** (17.2 mg, 0.045 mmol), 6M HCl (5 mL) and AcOH (0.5 mL). The product was purified using reverse phase silica gel chromatography (H_2O:MeOH 1:1) to give a white solid (8.5 mg, 0.038 mmol, 84%). **^{1}H NMR** (400 MHz, D_2O) δ 1.99 – 2.18 (m, 2H, SCH_2CH_2), 2.33 (s, 3H, Ar CH_3), 3.04 (t, *J* = 7.6 Hz, 2H, SCH_2), 3.79 – 3.87 (m, 1H, NH_2CH), 7.26 (d, *J* = 8.0 Hz, 2H, ArC*H*), 7.35 – 7.43 (m, 2H, Ar C*H*). **^{13}C NMR** (126 MHz, D_2O) δ 20.0 (Ar CH_3), 29.0 (SCH_2 + SCH_2CH_2), 51.4 (NH_2CH), 129.6 (Ar*C*), 129.9 (Ar*C*H), 130.7 (Ar*C*H), 137.8 (Ar*C*), 171.3 (*C*O). **HRMS** $C_{11}H_{15}O_2SNNa$ (M+Na$^+$) predicted 248.0716, found 248.0712.

Diastereoselective Synthesis

Synthesis of Karady-Beckwith Alkene

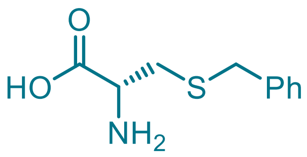

S9

***S*-benzyl-*L*-cysteine (S9)**

To a round bottom flask was added L-cysteine (12 g, 76.0 mmol), aqueous NaOH (2 M, 125 mL) and EtOH (96 mL). The reaction was stirred quickly at room temperature until clear, and cooled to 0 °C. Benzyl bromide (9.0 mL, 76 mL) was then added slowly and the reaction mixture stirred at room temperature for 1 hour. Concentrated HCl was then added carefully to adjust the pH to 7, as a white solid precipitate formed. The solid was filtered and washed sequentially with water, EtOH and Et_2O to obtain the product as a while solid (14.3 g, 67.7 mmol, 89%). **^{1}H NMR** (400 MHz, D_2O) δ 2.82 (dd, *J* =

15.0, 7.7 Hz, 1H, CHCH_aH$_b$S), 2.93 (dd, J = 15.1, 4.5 Hz, 1H, CHCH$_a$$H_b$S), 3.67 (d, J = 0.9 Hz, 2H, PhCH_2S), 4.00 (dd, J = 7.7, 4.4 Hz, 1H, NHCH), 7.14 – 7.21 (m, 1H, Ar CH), 7.22 – 7.27 (m, 4H, Ar CH). **^{13}C NMR** (101 MHz, D_2O) δ 35.2 (SCH$_2$CH), 36.5 (PhCH$_2$S), 51.6 (NH$_2$$C$H), 127.5 (Ar C), 128.8 (Ar CH), 128.9 (Ar CH), 137.4 (Ar CH), 170.2 (CO). **HRMS** $C_{10}H_{14}O_2NS$ (M+H$^+$) predicted 212.0740, found 212.0741.

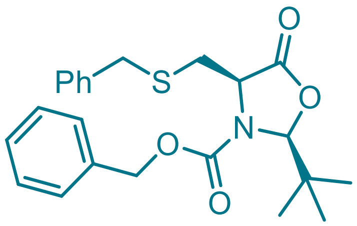

S10

Benzyl (2*S*,4*R*)-4-((benzylthio)methyl)-2-(tert-butyl)-5-oxooxazolidine-3-carboxylate (S10)

To a round bottom flask was added S-benzyl-Lcysteine (7.00 g, 32.9 mmol, 1 equiv.), NaOH (1.26 g, 31.5 mmol, 0.95 equiv), and dry MeOH (350 mL). The reaction was stirred at room temperature for 30 minutes. Trimethylacetaldehyde (4.30 mL, 32.9 mmol, 1.2 equiv) and activated 3 Å molecular sieves (35 g) were then added to the reaction flask. The reaction was placed under nitrogen atmosphere and stirred at room temperature until the starting material had been consumed (^{1}H NMR). The reaction was filtered through celite and concentrated before drying under high vacuum for 5 hours to afford the imine as a white solid. The imine then was dissolved in anhydrous DCM (175 mL) and cooled to -30 °C. Benzyl chloroformate (3.51 mL, 49.4 mmol, 1.5 equiv) was added to the reaction dropwise via syringe. The reaction was allowed to reach 0 °C and stirred for 18 hours then warmed to room temperature and stirred for an additional 6 hours. The mixture was washed with 1 M aqueous NaOH (1x 150 mL). The organic layer was dried over $MgSO_4$, filtered, and concentrated under reduced pressure. The residue was purified by column chromatography (10% EtOAc/hexane) to afford the product (1.50 g, 3.62 mmol, 11% yield) as a colourless oil. **^{1}H NMR** (400 MHz, $CDCl_3$) δ 0.89 (s, 9H, C(CH_3)$_3$), 2.75 (dd, J = 13.9, 6.2 Hz, 1H, COCHCH_aH$_b$), 2.90 (dd, J = 13.9, 8.0 Hz, 1H, COCHCH$_a$$H_b$), 3.75 (q, J = 13.5 Hz, 2H, PhCH_2S), 4.51 (t, J = 7.1 Hz, 1H, COCH), 5.11 – 5.24 (m, 2H, PhCH_2O), 5.52 (s, 1H, OCHN), 7.19 – 7.29 (m, 5H, Ar CH), 7.35 (s, 5H). **^{13}C NMR** (101 MHz, $CDCl_3$) δ 24.78 (CH_3), 33.3 (SCH$_2$CH), 36.5 (PhCH$_2$S), 36.8 (C(CH$_3$)$_3$), 57.5 (NCHCO), 68.5 (PHCH$_2$O), 96.3 (OCHN), 127.1 (Ar CH), 128.5 (Ar

CH), 128.5 (Ar CH), 128.7 (2 × Ar CH) , 129.0 (Ar CH), 135.0 (Ar *C*), 137.7 (Ar *C*), 155.8 (*C*O), 171.2 (*C*O). **HRMS** $C_{23}H_{28}O_6NS$ (M+H$^+$) predicted 414.1734, found 414.1736

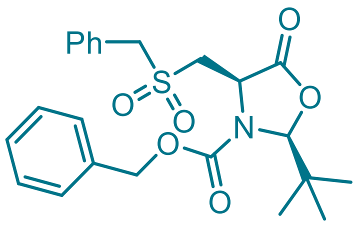

S11

Benzyl (2*S*,4*R*)-4-((benzylsulfonyl)methyl)-2-(tert-butyl)-5-oxooxazolidine-3-carboxylate (S11)

To a round bottom flask was added benzyl (2S,4R)- 4-((benzylthio)methyl)-2-(tert-butyl)-5-oxooxazolidine-3-carboxylate (1.50 g, 3.63 mmol, 1 equiv), *meta*-chloroperoxybenzoic acid (1.57 g, 9.08 mmol, 2.5 equiv), and CH_2Cl_2 (120 mL). The reaction was stirred at room temperature for 18 hours. The reaction mixture was then washed with 1 M aqueous sodium hydroxide (3 × 40 mL). The organic layer was dried over $MgSO_4$, filtered, and concentrated under reduced pressure. The crude residue was purified by column chromatography (30% EtOAc/hexane) to afford the product (1.49 g, 3.34 mmol, 92% yield) as a colourless oil. **^{1}H NMR** (400 MHz, $CDCl_3$) δ 0.89 (s, 9H, C(C*H*$_3$)$_3$), 3.15 (dd, *J* = 15.4, 4.0 Hz, 1H, COCHC*H*$_a$H$_b$), 3.44 (dd, *J* = 15.3, 8.1 Hz, 1H, COCHCH$_a$*H*$_b$), 4.42 (d, *J* = 14.0 Hz, 1H, PhC*H*$_a$H$_b$-SO$_2$), 4.67 (d, *J* = 14.1 Hz, 1H, PhCH$_a$*H*$_b$SO$_2$), 5.08 (dd, *J* = 8.1, 4.1 Hz, 1H, NC*H*CO), 5.18 – 5.29 (m, 2H, PhC*H*$_2$O), 5.62 (s, 1H, OC*H*N), 7.30 – 7.45 (m, 10H, Ar C*H*). **^{13}C NMR** (101 MHz, $CDCl_3$) δ 24.5 (C*H*$_3$), 37.1 ((*C*(CH$_3$)$_3$), 52.7 (SO$_2$CH$_2$CH), 53.6 (SO$_2$CH$_2$CH), 60.4 (PhCH$_2$SO$_2$), 69.0 (Ph*C*H$_2$O), 97.0 (O*C*HN), 127.9 (Ar *C*), 128.8 (Ar *C*H), 128.8 (Ar *C*H), 128.9 (Ar *C*H), 129.1 (Ar *C*H), 129.2 (Ar *C*H), 130.9 (Ar *C*H), 134.9 (Ar *C*), 155.3 (*C*O), 170.7 (CO). **HRMS** $C_{23}H_{28}O_4NS$ (M+H$^+$) predicted 446.1632, found 446.1626.

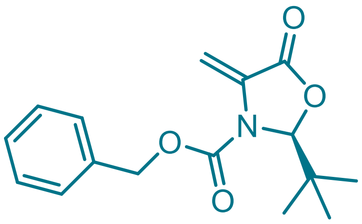

(+)-**9**

Benzyl (*S*)-2-(tert-butyl)-4-methylene-5-oxooxazolidine-3-carboxylate ((+)-9)

To a round bottom flask was added (benzyl (2S,4R)-4-((benzylsulfonyl)methyl)-2-(tert-butyl)-5-oxooxazolidine-3-carboxylate) (1.35 g, 3.03 mmol, 1 equiv), and CH_2Cl_2 (40 mL). The flask was cooled to 0 °C in an ice bath, and DBU (497 µL, 3.33 mmol, 1.1 equiv) was added dropwise. The reaction was stirred at 0 °C until the starting material had been consumed (determined by TLC, around 15 minutes). While still at 0 °C, the reaction mixture was quenched with saturated aqueous ammonium chloride (10 mL), the layers were separated, and the organic phase was washed with saturated aqueous ammonium chloride (3 × 20 mL). The organic layer was then dried over $MgSO_4$, filtered, and concentrated under reduced pressure. The crude residue was purified by column chromatography (10% EtOAc/hexane) to afford the product (801 mg, 2.76 mmol, 91% yield) as a colourless oil. **^{1}H NMR** (400 MHz, $CDCl_3$) δ 0.93 (s, 9H, C(C*H*$_3$)$_3$), 5.26 (d, *J* = 1.6 Hz, 2H, PHC*H*$_2$O), 5.58 – 5.77 (m, 3H, CC*H*$_2$ + OC*H*N), 7.33 – 7.45 (m, 5H, Ar C*H*). **^{13}C NMR** (101 MHz, $CDCl_3$) δ 24.3 (C*H*$_3$), 38.6 (C(CH$_3$)$_3$), 68.7 (Ph*C*H$_2$O), 93.9 (*C*C*H*$_2$), 104.3 (C*C*H$_2$), 128.6 (Ar *C*H), 128.7 (2 × Ar *C*H), 128.8 (Ar *C*H), 130.1 (Ar *C*H), 134.6 (Ar *C*), 158.9f (*C*O), 164.5 (*C*O). **HRMS** $C_{16}H_{20}O_4N$ (M+H$^+$) predicted 290.1387, found 290.1384. $[\alpha]_D^{28}$ -17.6. (*c* 1.0 mg/mL, $CHCl_3$).

Karady-Beckwith Example

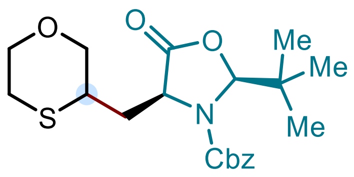

10

Benzyl (2*S*,4*S*)-4-((1,4-oxathian-3-yl)methyl)-2-(tert-butyl)-5-oxooxazolidine-3-carboxylate (11)

11 was prepared according to general method a, using the Karady Beckwith alkene (28.9 mg, 0.1 mmol) and 1,4-oxathiane (104 µL, 1.0 mmol). The crude product was purified using column chromatography (20% EtOAc: Hexane) to give an off-white oil as a mixture of inseparable diastereomers (13.7 mg, 0.035 mmol, 35%, dr 1:1.3). **^{1}H NMR** (400 MHz, $CDCl_3$) δ 0.96 (d, *J* = 6.7 Hz, 20.7H, $(CH_3)_3$, D1+D2), 1.95 – 2.08 (m, 2.3H, $NCHCH_aH_b$, D1+D2), 2.11 – 2.27 (m, 1H, SCH_aH_b, D1), 2.28 – 2.39 (m, 2.3H, $NCHCH_aH_b$, D1+D2), 2.39 – 2.49 (m, 1.3H, SCH_aH_b, D2), 2.50 – 2.68 (m, 1H, SCH_aH_b, D1), 2.74 – 2.85 (m, 1.3H, SCH_aH_b, D2), 2.96 – 3.04 (m, 1H, SC*H*, D2), 3.06 – 3.16 (m, 1H, SC*H*, D1), 3.61 – 3.74 (m, 2.3H, OCH_aH_bCH, D1+D2), 3.80 – 3.91 (m, 4.6H, OCH_2CH_2, D1+D2), 4.03 (dd, *J* = 11.8, 2.7 Hz, 2.3H, OCH_aH_bCH, D1+D2), 4.60 (dd, *J* = 7.4, 5.3 Hz, 1.3H, NC*H*CO, D2), 4.71 (dd, *J* = 10.0, 4.6 Hz, 1H, NC*H*CO, D1), 5.09 – 5.26 (m, 4.6H, $PhCH_2O$, D1+D2), 5.58 (d, *J* = 2.1 Hz, 2H, NC*H*O, D1+D2), 7.34 – 7.41 (m, 11.5H, Ar C*H*). **^{13}C NMR** (101 MHz, $CDCl_3$) δ 24.8 ($(CH_3)_3$, D1), 24.6 ($(CH_3)_3$, D2), 26.0 ($C(CH_3)_3$, D1), 34.5 ($C(CH_3)_3$, D2), 34.9 ($NCHCH_2$, D1), 35.6 (S*C*H, D1), 35.7 ($NCHCH_2$, D1), 36.9 (SCH_2, D1), 37.1 (SCH_2, D2), 55.0 (N*C*HCO, D1), 55.3 (N*C*HCO, D2), 68.5 ($PhCH_2O$, D1), 68.6 ($PhCH_2O$, D2 + OCH_2CH, D2), 68.7 (OCH_2CH, D1), 73.0 (OCH_2CH_2, D2), 73.2 (OCH_2CH_2, D1), 96.2 (N*C*HO, D1), 96.5 (N*C*HO, D2), 128.5 – 128.9 (6 x Ar *C*H, D1+D2), 135.0 (Ar *C*, D2), 135.1 (Ar *C*, D1), 155.8 (O*C*ON, D1), 156.0 (O*C*ON, D2), 172.4 (O*C*O, D2), 172.6 (O*C*O, D2). **HRMS** $C_{20}H_{28}O_5NS$ ($M+H^+$) predicted 384.1683, found 384.1674.

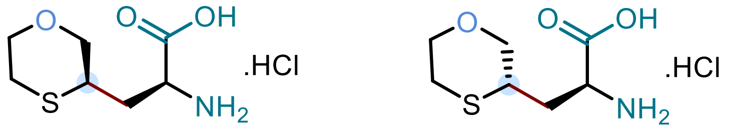

(2S, 3R)-**3c** and (2S, 3S)-**3c**

(2S)-2-Amino-3-((3R)-1,4-oxathian-3-yl)propanoic acid . HCl ((2S, 3R)**-3c) and (2S)-2-amino-3-((3S)-1,4-oxathian-3-yl)propanoic acid . HCl (**(2S, 3S)**-3c)**

To a round bottomed flask was added benzyl (2*S*,4*S*)-4-((1,4-oxathian-3-yl)methyl)-2-(tert-butyl)-5-oxooxazolidine-3-carboxylate (0.035 mmol, 13.7 mg) and concentrated HCl (3 mL). The reaction was stirred at 80 °C for 1 hour. Volatiles were then removed under reduced pressure to yield the product as an off-white amorphous solid (7.5 mg, 0.033 mmol, 95%, dr 1:1.3). **^{1}H NMR** (400 MHz, D_2O) δ 2.07 – 2.16 (m, 1.3H, $NH_2CHCH_aH_b$ with H_a italic, D2), 2.20 – 2.34 (m, 2H, $NH_2CHC\mathit{H}_2$, D1), 2.37 – 2.47 (m, 1.3H, $NH_2CHCH_a\mathit{H}_b$, D2), 2.55 – 2.66 (m, 2.6H, $SC\mathit{H}_2$, D2), 2.83 – 2.96 (m, 3H, $SC\mathit{H}_2$, D1 + SC*H*, D1), 2.98 – 3.06 (m, 1.3H, SC*H* ,D2), 3.72 – 3.80 (m, 2.3H, $OC\mathit{H}_aH_bCHS$, D1+D2), 3.93 – 3.97 (m, 4.6H, $OC\mathit{H}_2CH_2$, D1+D2), 4.03 – 4.12 (m, 2.3H, $OCH_a\mathit{H}_bCHS$, D1+D2), 4.18 – 4.28 (m, 2H, $NH_2C\mathit{H}$, D1+D2). **^{13}C NMR** (101 MHz, D_2O) δ 24.0 ($\mathit{S}CH_2$, D1+D2), 31.3 ($NH_2CH\mathit{C}H_2$, D1), 31.4 ($NH_2CH\mathit{C}H_2$, D1), 34.3 (S*C*H, D2), 34.5 (S*C*H, D1), 51.3 ($NH_2\mathit{C}H$, D1+D2), 68.3 ($OCH_2\mathit{C}H_2$, D1+D2), 72.4 ($O\mathit{C}H_2CHS$, D1), 72.5 ($O\mathit{C}H_2CHS$, D2), 172.2 (*C*O, D1+D2). **HRMS** $C_7H_{13}O_3SNNa$ ($M+Na^+$) predicted 214.0508, found 214.0514.

The enantiomeric purity of ((2S, 3S)-**3c**) and ((2S, 3R)-**3c**) was determined by HPLC analysis in comparison with authentic racemic material (92:8 er_M, 92:8 er_m shown; Astec CHIROBIOTIC T column, 1:1 MeOH:H_2O, 1.0 mL/min, 25 °C, 205 nm).

Instead of the four possible peaks (**D1-E2**, **D1-E1, D2-E2, D2-E1**), we instead observed three peaks in the chiral HPLC trace. The observed diastereomeric ratio in the racemic standard observed by ^{1}H NMR was 1:1.5 (D1:D2). This can further be observed in the corresponding chiral HPLC trace with the peaks at 6.818 min (arbitrary 1, **D1-E1**) and 7.830 min (relative 1.5, **D2-E1**), and the coincident diastereomers observed at 5.665 min (**D1-E2** + **D2-E2**). Therefore, we can assume that the relative ratio of the coincident diastereoisomers is 1+1.5, relative to the peak at 6.818, thus giving an overall isomeric ratio of **(1:1.5):1:1.5**.

Whilst for the enantioenriched sample, the diastereomeric ratio observed by ^{1}H NMR was 1.3:1 (D1:D2). The coincident diastereoisomers are at 4.83 min in the enantioenriched chiral HPLC trace, with the overall observed isomeric ratio being (**17:12):1.5:1**.

Racemic

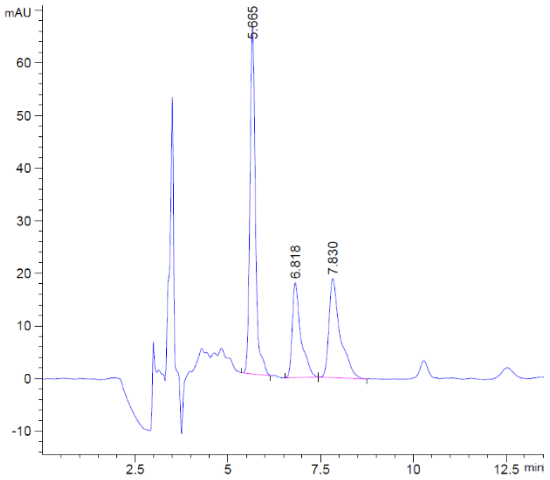

Peak #	RetTime [min]	Type	Width [min]	Area [mAU*s]	Height [mAU]	Area %
1	5.665	BB	0.1581	681.44745	66.20562	49.8012
2	6.818	BB	0.2344	291.76294	18.06565	21.3225
3	7.830	BB	0.3010	395.12598	18.81867	28.8764

Enantioenriched

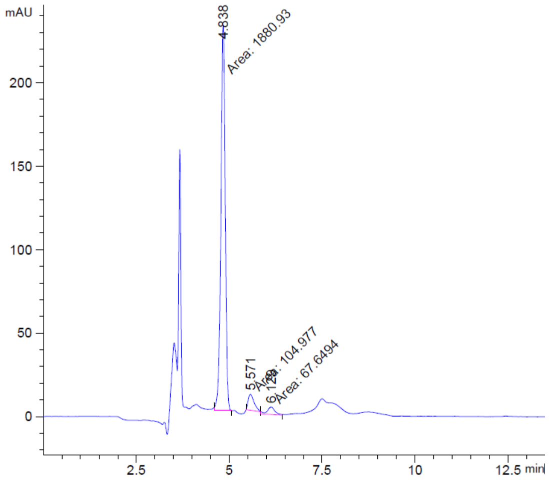

Peak #	RetTime [min]	Type	Width [min]	Area [mAU*s]	Height [mAU]	Area %
1	4.838	MM	0.1357	1880.92773	231.00006	91.5938
2	5.571	MM	0.1837	104.97672	9.52331	5.1120
3	6.129	MM	0.2470	67.64936	4.56469	3.2943

Note: a switch of major and minor diastereomers from racemic to enantiopure material was observed and confirmed by ^{1}H NMR.

Residue-Exchange Strategy

Peptide Starting Materials Synthesis

General Method B: Preparation of dehydro di-peptides

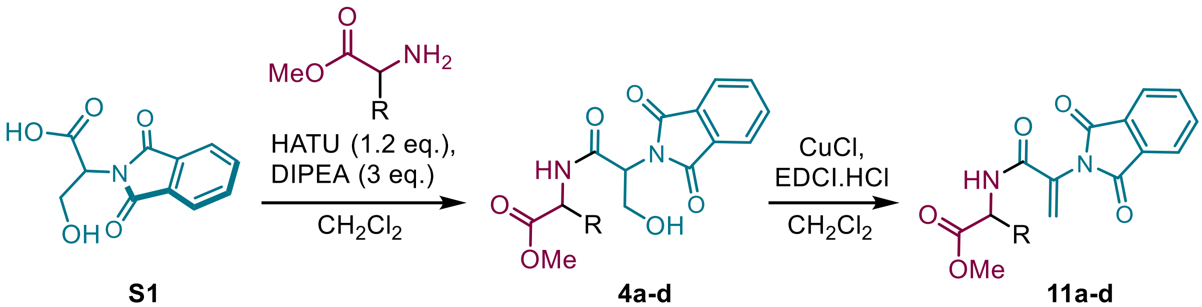

S1 **4a-d** **11a-d**

S1 (470 mg, 2.0 mmol) was dissolved in dry CH_2Cl_2 (15 mL) under an atmosphere of nitrogen and the solution cooled to 0 °C. The amino acid methyl ester hydrochloride (2.0 mmol) was then added followed by HATU (836 mg, 2.20 mmol) and slow addition of DIPEA (0.497 mL, 6.0 mmol). The reaction mixture was slowly warmed to room temperature and stirred overnight. The reaction mixture was then washed with water (3 × 10 mL), brine (10 mL), dried over $MgSO_4$ and evaporated under reduced pressure. The crude product was then used in the next reaction forward without further purification. The crude residue was re-dissolved in CH_2Cl_2 (15 mL) and placed under an atmosphere of nitrogen. To this was added EDCI.HCl (1.1 eq.) and CuCl (0.33 eq.), and the reaction stirred at room temperature for 18 hours. The solution was then washed with two portions of water (2 × 10 mL), dried over $MgSO_4$ and evaporated under reduced pressure. The crude product was purified using column chromatography (EtOAc / Hexane).

The following starting materials were prepared according to literature procedures: 2-(1,3-dioxoisoindolin-2-yl)-3-hydroxypropanoic acid (S1), ethyl 2-(1,3-dioxoisoindolin-2-yl)acrylate (2).

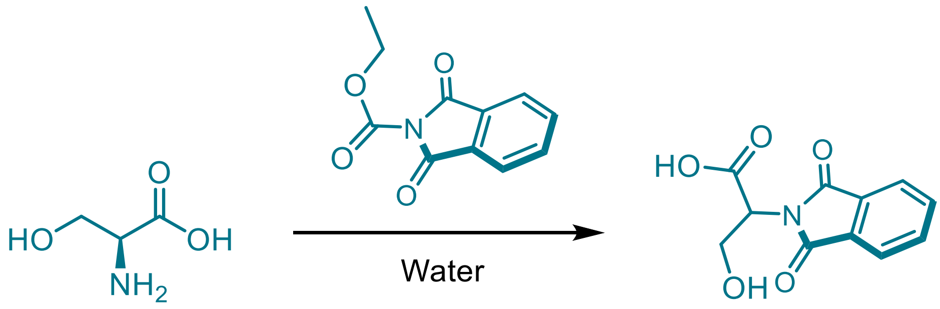

S1

2-(1,3-dioxoisoindolin-2-yl)-3-hydroxypropanoic acid (S1)

L-serine (6.0 g, 57.1 mmol) and N-carboxy ethyl phthalimide (12.5 g, 57.1 mmol) were dissolved in water (45 mL) and the solution stirred for 30 minutes. The reaction mixture was then filtered, and the filtrate acidified to pH 3 with 6 M HCl. The precipitated product was dissolved by heating, giving long needles upon cooling (7.65 g, 32.5 mmol, 57%) **^{1}H NMR** (500 MHz, $CDCl_3$) δ 4.21 – 4.33 (m, 2H, OC*H*$_2$), 5.08 (dd, *J* = 5.7, 4.1 Hz, 1H, NC*H*), 7.72 – 7.80 (m, 2H, Ar C*H*), 7.85 – 7.92 (m, 2H, Ar C*H*). **^{13}C NMR** (126 MHz, $CDCl_3$) δ 54.7 (N*C*H), 61.0 (HO*C*H$_2$), 123.9 (d, *J* = 8.5 Hz, Ar *C*H), 131.6 (Ar *C*), 134.6 (Ar *C*H), 168.2 (*C*O), 171.6 (*C*O). **HRMS** $C_{13}H_{11}O_4NNa$ (M+Na$^+$) predicted 268.0580, found 268.0587.

The data are in accordance with the literature.[5]

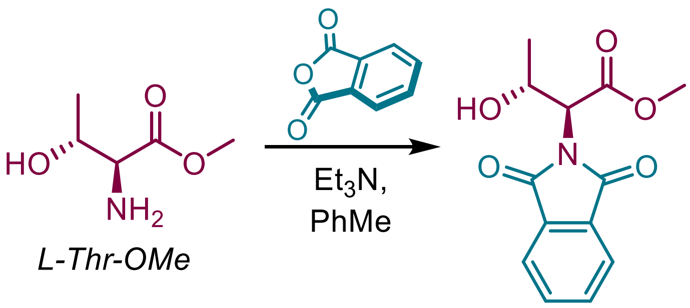

S2

Methyl (2*S*)-2-(1,3-dioxoisoindolin-2-yl)-3-hydroxybutanoate (S2)

To a solution of L-threonine methyl ester (2.00 g, 11.8 mmol) and phthalic anhydride (1.75 g, 11.9 mmol) in toluene (35 mL) was added triethylamine (1.64 mL, 11.78 mmol), and the reaction was stirred for 2 hours under reflux in a Dean Stark apparatus. The reaction mixture was then concentrated under reduced pressure, re-dissolved in EtOAc (50 mL) and washed with 10% citric acid (25 mL) and

saturated Na_2CO_3 (25 mL). The solution was dried over $MgSO_4$ and evaporated under reduced pressure to yield an off-white solid (1.51 g, 5.75 mmol, 49%) which was used in the next step without further purification. **^{1}H NMR** (500 MHz, $CDCl_3$) δ 1.22 (d, J = 6.5 Hz, 3H, CHC*H*$_3$), 3.80 (s, 3H, OC*H*$_3$), 4.07 (d, J = 9.7 Hz, 1H, O*H*), 4.62 – 4.70 (m, 1H, C*H*CH$_3$), 4.99 (d, J = 4.1 Hz, 1H, NC*H*), 7.74 – 7.83 (m, 2H, Ar C*H*), 7.88 – 7.95 (m, 2H, Ar C*H*). **^{13}C NMR** (101 MHz, $CDCl_3$) δ 20.20 (*C*H$_3$CH), 52.96 (O*C*H$_3$), 59.16 (HO*C*H), 66.72 (N*C*H), 123.94 (Ar CH), 131.66 (Ar *C*), 134.63 (Ar *C*H), 168.33 (*C*O), 168.74 (*C*O). **HRMS** $C_{13}H_{13}O_5NNa$ (M+Na$^+$) predicted 286.0686, found 286.0684.

The data are in accordance with the literature.[6]

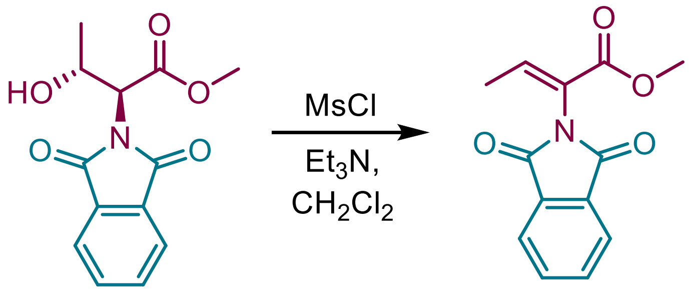

S2 **S3**

Methyl (Z)-2-(1,3-dioxoisoindolin-2-yl)but-2-enoate (S3)

S2 (1.50 g, 5.70 mmol), triethylamine (7.98 mmol, 1.11 mL) and MsCl (7.98 mmol, 617 µL) were dissolved in CH_2Cl_2 at 0 °C. After stirring at room temperature for 3 hours, another portion of triethylamine (1.59 mL, 11.4 mmol) was added and the solution heated to reflux for a further 2.5 hours. The reaction mixture was then acidified with 1M HCl, extracted with CH_2Cl_2 (2 × 15 mL), washed with brine (1 × 15 mL), dried over $MgSO_4$, and evaporated under reduced pressure. The crude product was purified using column chromatography (30% EtOAc : Hexane) to yield a yellow solid (885 mg, 3.61 mmol, 63%). **^{1}H NMR** (500 MHz, $CDCl_3$) δ 1.83 (d, J = 7.2 Hz, 3H, CHC*H*$_3$), 3.77 (s, 3H, OC*H*$_3$), 7.40 (q, J = 7.2 Hz, 1H, C*H*CH$_3$), 7.73 – 7.82 (m, 2H, Ar C*H*), 7.88 – 7.96 (m, 2H, Ar C*H*). **^{13}C NMR** (101 MHz, $CDCl_3$) δ 14.5 (*C*H$_3$CH), 52.6 (O*C*H$_3$), 123.3 (N*C*CH), 123.9 (Ar *C*H), 132.1 (Ar *C*), 134.4 (Ar *C*H), 143.3 (*C*HCH$_3$), 163.1 (*C*O), 166.6 (*C*O). **HRMS** $C_{13}H_{11}O_4NNa$ (M+Na$^+$) predicted 268.0580, found 268.0579.

The data are in accordance with the literature.[7]

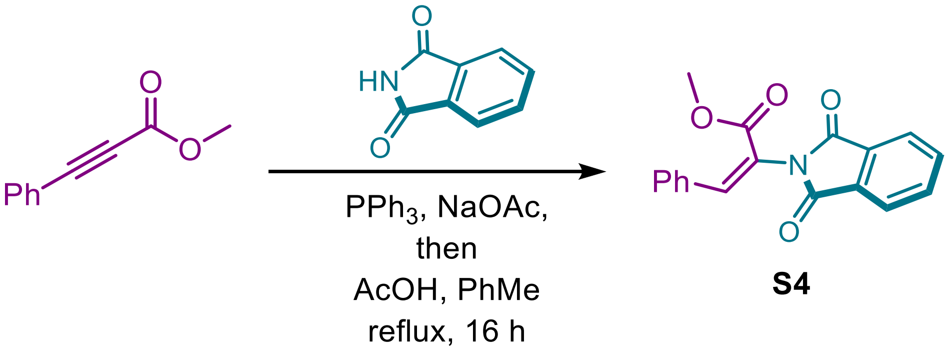

Methyl-2-(1,3-dioxoisoindolin-2-yl)-3-phenylacrylate (S4)

Phthalimide (1.77 g, 12 mmol), sodium acetate (492 mg, 6 mmol) and PPh_3 (315 mg, 1.2 mmol) were dissolved in toluene (12 mL) and stirred under reflux for 10 minutes. The solution was then cooled to room temperature, followed by addition of glacial acetic acid (0.69 mL, 12 mmol) and methyl 3-phenylpropiolate (1.92 g, 12 mmol). The reaction mixture was then heated at reflux for 16 hours. The mixture was then cooled, filtered, and the filtrate diluted with CH_2Cl_2 (30 mL) and washed with $NaHCO_3$ (15 mL) and brine (15 mL), dried over $MgSO_4$ and concentrated under vacuum to yield a white solid product, which was used without further purification (2.17 g, 7.08 mmol, 59%). **^{1}H NMR** (500 MHz, $CDCl_3$) δ 3.83 (s, 3H, OC*H*$_3$), 7.27 – 7.35 (m, 3H, Ar C*H*), 7.40 (d, *J* = 7.3 Hz, 2H, Ar C*H*), 7.76 – 7.83 (m, 2H, Ar C*H*), 7.89 – 7.96 (m, 2H, Ar C*H*), 8.12 (s, 1H, C=C*H*). **^{13}C NMR** (126 MHz, $CDCl_3$) δ 53.0 (O*C*H$_3$), 120.0 (*C*=CH), 124.1 (Ar CH), 129.0 (Ar *C*), 129.4 (Ar *C*), 130.6 (Ar *C*H), 132.2 (Ar *C*H), 132.3 (Ar *C*H), 134.5 (Ar *C*H), 143.2 (C=*C*H), 164.0 (*C*O), 167.0 (*C*O). **HRMS** $C_{18}H_{13}O_4NNa$ (M+Na$^+$) predicted 330.0737, found 330.0729.

The data are in accordance with the literature.[8]

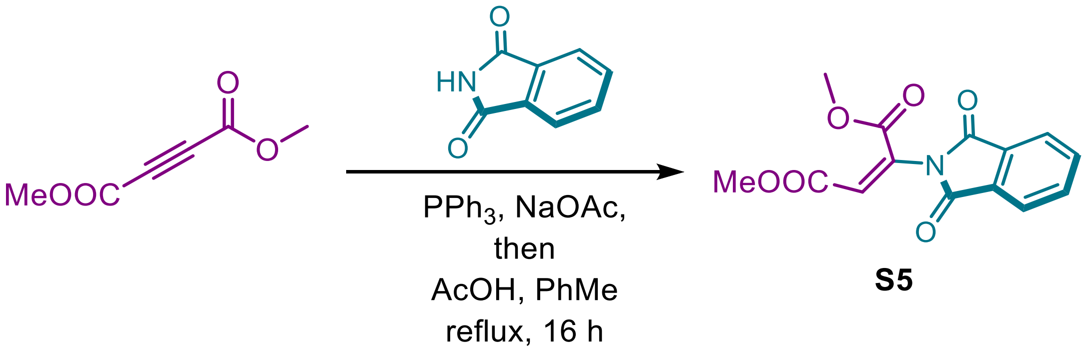

S5

Dimethyl 2-(1,3-dioxoisoindolin-2-yl)maleate (S5)

Phthalimide (1.77 g, 12 mmol), sodium acetate (492 mg, 6 mmol) and PPh_3 (315 mg, 1.2 mmol) were dissolved in toluene (12 mL) and stirred under reflux for 10 minutes. The solution was then cooled to room temperature, followed by addition of glacial acetic acid (0.69 mL, 12 mmol) and dimethyl but-2-ynedioate (1.70 g, 12 mmol). The reaction mixture was then heated at reflux for 16 hours. The mixture was then cooled, filtered, and the filtrate diluted with CH_2Cl_2 (30 mL) and washed with $NaHCO_3$ (15 mL) and brine (15 mL), dried over $MgSO_4$ and concentrated under vacuum to yield a white solid product, which was used without further purification (1.77 g, 6.12 mmol, 51%). **^{1}H NMR** (500 MHz, $CDCl_3$) δ 3.72 (s, 3H, OC*H*$_3$), 3.86 (s, 3H, OC*H*$_3$), 7.17 (s, 1H, C=C*H*), 7.75 – 7.83 (m, 2H, Ar C*H*), 7.89 – 7.97 (m, 2H, Ar C*H*). **^{13}C NMR** (126 MHz, $CDCl_3$) δ 52.5 (O*C*H$_3$), 53.5 (O*C*H$_3$), 124.2 (Ar *C*H), 128.7 (Ar *C*) , 131.7 (*C*=CH), 132.1 (C=*C*H), 134.6 (Ar *C*H), 162.4 (*C*O), 163.2 (*C*O), 165.6 (*C*O). **HRMS** $C_{14}H_{11}O_6NNa$ (M+Na$^+$) predicted 312.0479, found 312.0469.

The data are in accordance with the literature. [8]

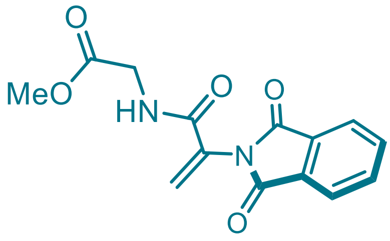

11a

Methyl (2-(1,3-dioxoisoindolin-2-yl)acryloyl)glycinate (11a)

11a was prepared according to general method b, using glycine methyl ester hydrochloride (2.0 mmol, 251 mg), followed by EDCI.HCl (344 mg, 1.64 mmol) and CuCl (52.8 mg, 0.54 mmol). The crude product

was purified using column chromatography (EtOAc) to give a white solid (179 mg, 0.620 mmol, 31%). **1H NMR** (400 MHz, $CDCl_3$) δ 3.79 (s, 3H, OC*H*$_3$), 4.16 (d, *J* = 5.0 Hz, 2H, NHC*H*$_2$), 5.86 (d, *J* = 1.4 Hz, 1H, CC*H*$_a$H$_b$), 6.24 (d, *J* = 1.4 Hz, 1H, CC*H*$_a$H$_b$), 6.56 (s, 1H, N*H*) 7.71 – 7.82 (m, 2H, Ar C*H*), 7.86 – 7.96 (m, 2H, Ar C*H*). **13C NMR** (101 MHz, $CDCl_3$) δ 41.7 (NH*C*H$_2$), 52.6 (O*C*H$_3$), 120.8 (C*C*H$_2$), 124.0 (Ar *C*H), 131.7 (Ar *C*), 132.5 (*C*CH$_2$), 134.6 (Ar *C*H), 163.1 (*C*O), 166.4 (*C*O), 170.0 (*C*O). **ν_{max}** (thin film/cm^{-1}): 3340, 2980, 1721, 1677, 1632, 1537, 1383, 1298, 1209, 1115, 887, 845, 719, 531, 467. **HRMS** $C_{14}H_{12}O_5N_2Na$ (M+Na$^+$) predicted 311.0638, found 311.0628.

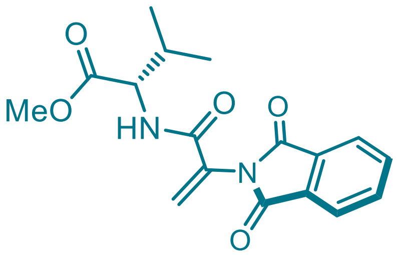

11b

Methyl (2-(1,3-dioxoisoindolin-2-yl)acryloyl)-*L*-valinate (11b)

11b was prepared according to general method b, using valine methyl ester hydrochloride (2.0 mmol, 335 mg), followed by EDCI.HCl (402 mg, 2.1 mmol) and CuCl (62.3 mg, 0.63 mmol). The crude product was purified using column chromatography (EtOAc) to give a white solid (257 mg, 0.780 mmol, 39%). **^{1}H NMR** (500 MHz, $CDCl_3$) δ 0.96 (d, *J* = 6.9 Hz, 3H, C*H*$_3$CH), 1.01 (d, *J* = 6.9 Hz, 3H, C*H*$_3$CH), 2.24 (hd, *J* = 6.9, 4.7 Hz, 1H, (CH$_3$)$_2$C*H*), 3.77 (s, 3H, OC*H*$_3$), 4.66 (dd, *J* = 8.7, 4.7 Hz, 1H, COC*H*), 5.85 (d, *J* = 1.4 Hz, 1H, CC*H*$_a$H$_b$), 6.19 (d, *J* = 1.5 Hz, 1H, CCH$_a$*H*$_b$), 6.52 (d, *J* = 8.8 Hz, 1H, N*H*), 7.72 – 7.79 (m, 2H, Ar C*H*), 7.86 – 7.95 (m, 2H, Ar C*H*). **^{13}C NMR** (126 MHz, $CDCl_3$) δ 17.8 (*C*H$_3$CH), 18.9 (*C*H$_3$CH), 31.8 (CH$_3$)$_2$*C*H), 52.4 (O*C*H$_3$), 57.5 (CO*C*H), 119.9 (C*C*H$_2$), 124.0 (Ar *C*H), 131.8 (Ar *C*), 132.9 (*C*CH$_2$), 134.6 (Ar *C*H), 162.9 (CO), 166.3 (CO), 172.2 (CO). **ν_{max}** (thin film/cm^{-1}): 3285, 2970, 2937, 1786, 1723, 1655, 1620, 1536, 1469, 1440, 1374, 1305, 1254, 1205, 1152, 1129, 1101, 992, 882, 707. **HRMS** $C_{17}H_{19}O_5N_2$ (M+H$^+$) predicted 331.1288, found 331.1277. $[\alpha]_D^{28}$ 2.8. (*c* 1.0 mg/mL, MeOH).

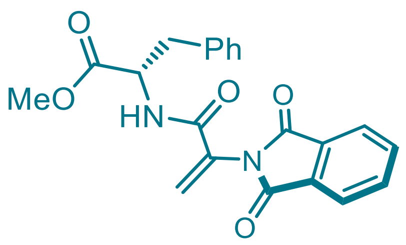

11c

Methyl (2-(1,3-dioxoisoindolin-2-yl)acryloyl)-*L*-phenylalaninate (11c)

11c was prepared according to general method b, using phenylalanine methyl ester hydrochloride (2.0 mmol, 430 mg), followed by EDCI.HCl (397 mg, 1.98 mmol) and CuCl (58.3 mg, 0.60 mmol). The crude product was purified using column chromatography (EtOAc) to give a white solid (190 mg, 0.500 mmol, 25%). **^{1}H NMR** (500 MHz, $CDCl_3$) δ 3.15 – 3.28 (m, 2H, PhCH_2), 3.76 (s, 3H, OCH_3), 4.96 (ddd, *J* = 7.6, 5.6, 4.9 Hz, 1H, COC*H*), 5.81 (d, *J* = 1.5 Hz, 1H, CC$H_a$$H_b$), 6.07 (d, *J* = 1.5 Hz, 1H, CC$H_a$$H_b$), 6.48 (d, *J* = 7.6 Hz, 1H, N*H*), 7.13 – 7.17 (m, 2H, Ar C*H*), 7.21 – 7.25 (m, 1H, Ar C*H*), 7.26 – 7.30 (m, 2H, Ar C*H*), 7.73 – 7.81 (m, 2H, Ar C*H*), 7.87 – 7.95 (m, 2H, Ar C*H*). **^{13}C NMR** (126 MHz, $CDCl_3$) δ 37.6 (Ph*C*H_2), 52.5 (O*C*H_3), 53.4 (CO*C*H), 120.2 (C*C*H_2), 124.0 (Ar CH), 127.2 (Ar *C*H), 128.6 (Ar *C*H), 129.5 (Ar *C*H), 131.8 (Ar *C*), 132.7 (*C*CH_2), 134.6 (Ar *C*H), 135.5 (Ar *C*), 162.4 (CO), 166.3 (CO), 171.5 (CO). **ν_{max}** (thin film/cm^{-1}): 3293, 2980, 1788, 1723, 1656, 1534, 1378, 1310, 1206, 1066, 885. **HRMS** $C_{21}H_{19}O_5N_2$ (M+H$^+$) predicted 379.1288, found 379.1279. $[\alpha]_D^{28}$ 10.0. (*c* 1.0 mg/mL, MeOH).

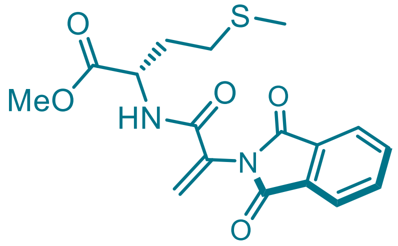

11d

Methyl (2-(1,3-dioxoisoindolin-2-yl)acryloyl)-*L*-methioninate (11d)

11d was prepared according to general method b, using methionine methyl ester hydrochloride (2.0 mmol, 394 mg), followed by EDCI.HCl (393 mg, 1.98 mmol) and CuCl (58.3 mg, 0.60 mmol). The crude product was purified using column chromatography (EtOAc) to give a white solid (210 mg, 0.58 mmol,

29%). **^{1}H NMR** (400 MHz, $CDCl_3$) δ 2.04 – 2.15 (m, 4H, SCH_3 + $SCH_2CH_aH_b$), 2.28 – 2.31 (m, 1H, $SCH_2CH_aH_b$), 2.61 (t, *J* = 7.3 Hz, 2H, SCH_2), 3.80 (s, 3H, OCH_3), 4.82 – 4.92 (m, 1H, NHC*H*), 5.86 (d, *J* = 1.4 Hz, 1H, CCH_aH_b), 6.26 (d, *J* = 1.4 Hz, 1H, CCH_aH_b), 6.90 (d, *J* = 7.7 Hz, 1H, N*H*), 7.74 – 7.83 (m, 2H, ArC*H*), 7.88 – 7.97 (m, 2H, ArC*H*). **^{13}C NMR** (101 MHz, $CDCl_3$) δ 15.5 (SCH_3), 29.9 (SCH_2), 31.3 (SCH_2CH_2), 52.1 (NH*C*H), 52.7 (OCH_3), 120.6 (CCH_2), 124.0 (Ar *C*H), 131.7 (CCH_2), 132.6 (Ar *C*), 134.6 (Ar *C*H), 162.7 (*C*O), 166.3 (*C*O), 172.1 (*C*O). **HRMS** $C_{27}H_{18}O_5N_2Na$ (M+Na$^+$) predicted 385.0829, found 385.0822.

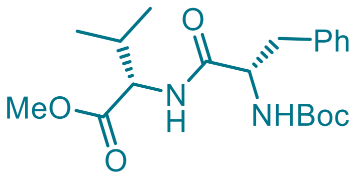

S6

Methyl (*tert*-butoxycarbonyl)-*L*-phenylalanyl-*L*-valinate (S6)

S6 was prepared according to a literature procedure[9] from valine methyl ester (1.01 g, 6.0 mmol), *N*-Boc phenylalanine (1.59 g, 6 mmol), DIPEA (2.08 mL, 12 mmol) and HATU (2.74 g, 7.2 mmol) to yield the product (1.24 g, 3.28 mmol, 54%). The product was used in the next step without further purification. **^{1}H NMR** (500 MHz, $CDCl_3$) δ 0.84 (d, *J* = 6.9 Hz, 3H, $(CH_3)_2CH$)), 0.87 (d, *J* = 6.8 Hz, 3H, $(CH_3)_2CH$)), 1.42 (s, 9H, $(CH_3)_3C$), 2.10 (heptd, *J* = 6.9, 5.0 Hz, 1H, $(CH_3)_2CH$)), 3.07 (d, *J* = 6.9 Hz, 2H, $PhCH_2$), 3.69 (s, 3H, OCH_3), 4.32 – 4.37 (m, 1H, NHC*H*), 4.46 (dd, *J* = 8.6, 5.1 Hz, 1H, NHC*H*), 5.01 (s, 1H, N*H*), 6.35 (d, *J* = 8.6 Hz, 1H, N*H*), 7.19 – 7.25 (m, 3H, Ar C*H*), 7.27 – 7.33 (m, 2H, Ar C*H*). **^{13}C NMR** (126 MHz, $CDCl_3$) 17.9 (CH_3), 19.0 (CH_3), 28.4 (3 × CH_3), 31.4 (CH_3), 38.1 ($PhCH_2$), 52.2 (NH*C*H), 56.0 (NH*C*H), 57.4 (CH), 80.4 ($(CH_3)_3C$), 127.1 (Ar *C*H), 128.8 (Ar *C*H), 129.5 (Ar *C*H) 136.7 (Ar *C*), 155.5 (*C*O), 171.3 (*C*O), 171.9 (*C*O). **HRMS** $C_{20}H_{31}N_2O_5$ (M+H$^+$) predicted 379.2155, found 379.2149.

The data are in accordance with the literature.[10]

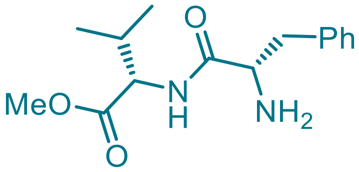

S7

Methyl *L*-phenylalanyl-*L*-valinate (S7)

S6 (1.24 g, 3.28 mmol) was dissolved in CH_2Cl_2 (30 mL) and cooled to -20 °C. To this solution was added TFA (15 mL) portion-wise and the solution stirred at room temperature for 24 hours. Solvents were then evaporated under reduced pressure, and the crude mixture was re-dissolved in ethyl acetate (30 mL), and washed with a saturated solution of $NaCO_3$ (15 mL), followed by brine (15 mL), dried over $MgSO_4$ and evaporated under reduced pressure. The crude product was purified using column chromatography (5% MeOH-CH_2Cl_2) to give a colourless oil (865 mg, 3.11 mmol, 94%). **^{1}H NMR** (500 MHz, $CDCl_3$) δ 0.82 (d, J = 6.9 Hz, 3H, $(CH_3)_2CH$)), 0.86 (d, J = 6.9 Hz, 3H, $(CH_3)_2CH$)) 2.06 – 2.17 (m, 1H, $(CH_3)_2CH$), 3.14 – 3.26 (m, 2H, PhCH_2), 3.73 (s, 3H, OCH_3), 4.37 (dd, J = 8.2, 5.0 Hz, 1H, NHC*H*), 4.60 (t, J = 7.3 Hz, 1H, NH_2C*H*), 6.96 (d, J = 8.2 Hz, 1H, N*H*), 7.19 – 7.24 (m, 2H, Ar C*H*), 7.28 – 7.36 (m, 3H, Ar C*H*). **^{13}C NMR** (126 MHz, $CDCl_3$) δ 17.3 (CH_3), 18.4 (CH_3), 30.8 ($(CH_3)_2CH$), 37.3 (PhCH_2), 52.7 (OCH_3), 55.1 (NH_2CH), 58.5 (NH*C*H), 128.5 (Ar *C*H), 129.3 (Ar *C*H), 129.5 (Ar *C*H), 132.6 (Ar *C*), 168.6 (*C*O), 171.4 (*C*O). **HRMS** $C_{15}H_{22}O_3N_2Na$ (M+Na$^+$) predicted 301.1523, found 301.1529. $[\alpha]_D^{28}$ 2.7. (*c* 1 mg/mL, MeOH).

The data are in accordance with the literature.[11]

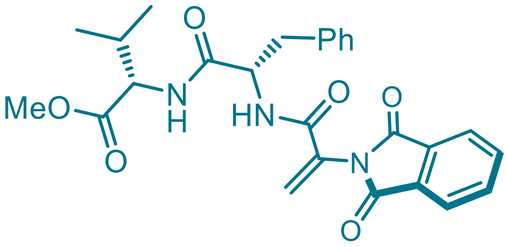

11e

Methyl (2-(1,3-dioxoisoindolin-2-yl)acryloyl)-*L*-phenylalanyl-*L*-valinate (11e)

11e was prepared according to general method b, using **S7** (3.11 mmol, 865 mg), **S1** (730 mg, 3.11 mmol), HATU (1.30 g, 3.42 mmol) and DIPEA (1.62 mL, 9.33 mmol), followed by EDCI.HCl (653 mg, 3.00 mmol) and CuCl (99.0 mg, 1.0 mmol). The crude product was purified using column chromatography (EtOAc) to give a white solid (250 mg, 0.529 mmol, 17%). **^{1}H NMR** (400 MHz, $CDCl_3$) δ 0.85 (d, *J* = 6.9 Hz, 3H, CH_3CH), 0.89 (d, *J* = 6.9 Hz, 3H, CH_3CH), 2.13 (pd, *J* = 6.9, 5.3 Hz, 1H, $(CH_3)_2CH$), 3.11 (dd, *J* = 13.8, 7.6 Hz, 1H, $PhCH_aH_b$), 3.28 (dd, *J* = 13.9, 5.7 Hz, 1H, $PhCH_aH_b$), 3.72 (s, 3H, OCH_3), 4.42 (dd, *J* = 8.3, 5.2 Hz, 1H, COC*H*CH), 4.77 (td, *J* = 7.6, 5.7 Hz, 1H, $COCHCH_2$), 5.80 (d, *J* = 1.4 Hz, 1H, CCH_aH_b), 6.10 (d, *J* = 1.4 Hz, 1H, CCH_aH_b), 6.36 (d, *J* = 8.3 Hz, 1H, N*H*), 6.72 (d, *J* = 7.5 Hz, 1H, N*H*), 7.21 (ddd, *J* = 8.5, 5.3, 3.3 Hz, 1H, Ar C*H*), 7.26 – 7.30 (m, 4H, Ar C*H*), 7.79 (dd, *J* = 5.5, 3.1 Hz, 2H, Ar C*H*), 7.87 – 7.96 (m, 2H, Ar C*H*). **^{13}C NMR** (101 MHz, $CDCl_3$) δ 17.9 (CH_3CH),18.8 (CH_3CH), 30.9 ($(CH_3)_2CH$), 37.8 ($PhCH_2$), 52.1 (OCH_3), 54.7 ($COCHCH_2$), 57.8 (CO*C*HCH), 120.6 (CCH_2), 124.0 (Ar *C*H), 127.1 (Ar *C*H), 128.7 (Ar *C*H), 129.4 (Ar *C*H), 131.7 (Ar *C*), 132.4 (CCH_2), 134.6 (Ar *C*H), 136.3 (Ar *C*), 162.8 (CO), 166.3 (CO), 170.2 (CO), 171.5 (CO). **v_{max}** (thin film/cm^{-1}): 3293, 2965, 1789, 1725, 1650, 1626, 1546, 1468, 1375, 1303, 1209, 1152, 1114, 950, 886, 714. **HRMS** $C_{26}H_{28}O_6N_3$ ($M+H^+$) predicted 478.1973, found 478.1953. $[\alpha]_D^{28}$ 10.5. (*c* 1.0 mg/mL, MeOH).

Residue Exchange Scope

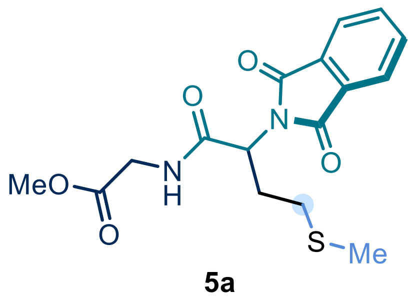

5a

Methyl (2-(1,3-dioxoisoindolin-2-yl)-4-(methylthio)butanoyl)glycinate (5a)

5a was prepared according to general method a, using **11a** (28.8 mg, 0.1 mmol) and dimethyl sulfide (62.1 μL, 1.0 mmol). The crude product was purified using column chromatography (60% EtOAc: Hexane) to give an off-white oil (12.2 mg, 0.035 mmol, 35%). **^{1}H NMR** (400 MHz, $CDCl_3$) δ 2.08 (s, 3H, $SC\textit{H}_3$), 2.48 – 2.64 (m, 4H, SCH_2 + $SCH_2C\textit{H}_2$), 3.74 (s, 3H, $OC\textit{H}_3$), 4.06 (d, *J* = 5.1 Hz, 2H, $NHC\textit{H}_2$), 5.03 – 5.10 (m, 1H, COC*H*), 6.65 (s, 1H, N*H*), 7.72 – 7.81 (m, 2H, Ar C*H*), 7.84 – 7.93 (m, 2H, Ar C*H*). **^{13}C NMR** (101 MHz, $CDCl_3$) δ 15.5 ($S\textit{C}H_3$), 28.0 ($S\textit{C}H_2$), 31.0 ($SCH_2\textit{C}H_2$), 41.5 ($NH\textit{C}H_2$), 52.5 ($O\textit{C}H_3$), 53.4 (COCH), 123.8 (Ar *C*H), 131.6 (Ar *C*), 134.5 (Ar *C*H), 168.0 (*C*O), 168.7 (*C*O), 170.0 (*C*O). **ν_{max}** (thin film/cm^{-1}): 3354, 2922, 2846, 1741, 1717, 1691, 1532, 1439, 1373, 1237, 1044, 916, 846, 789, 722, 530. **HRMS** $C_{16}H_{19}O_5N_2S$ ($M+H^+$) predicted 351.1009, found 351.0999.

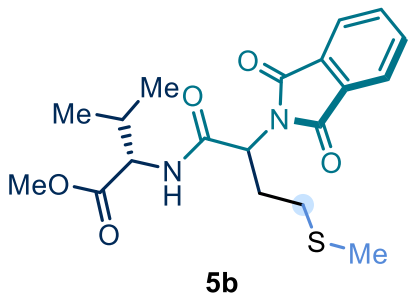

5b

Methyl (2-(1,3-dioxoisoindolin-2-yl)-4-(methylthio)butanoyl)-*L*-valinate (5b)

5b was prepared according to general method a, using **11b** (33.0 mg, 0.1 mmol) and dimethyl sulfide (62.1 μL, 1.0 mmol). The crude product was purified using column chromatography (60% EtOAc: Hexane) to give an off-white oil as a mixture of inseparable diastereomers (13.3 mg, 34%, 0.034 mmol, dr 1:1). **^{1}H NMR** (400 MHz, $CDCl_3$) δ 0.86 – 0.94 (m, 12H, $(C\textit{H}_3)_2CH$, D1+D2), 2.08 (s, 3H, $SC\textit{H}_3$, D1), 2.09 (s, 3H, $SC\textit{H}_3$, D2), 2.11 – 2.23 (m, 2H, $(CH_3)_2C\textit{H}$, D1+D2), 2.44 – 2.67 (m, 8H, $SC\textit{H}_2$ + $SCH_2C\textit{H}_2$,

D1+D2), 3.70 (s, 3H, OC*H*$_3$, D1), 3.71 (s, 3H, OC*H*$_3$, D2), 4.49 – 4.62 (m, 2H, NHC*H*, D1+D2), 5.00 – 5.09 (m, 2H, NC*H*, D1+D2), 6.65 (t, *J* = 8.1 Hz, 2H, N*H*, D1+D2), 7.71 – 7.81 (m, 4H, Ar C*H*), 7.84 – 7.94 (m, 4H, Ar C*H*). **^{13}C NMR** (126 MHz, CDCl$_3$) δ 15.4 (S*C*H$_3$, D1), 15.5 (S*C*H$_3$ D2), 17.7 (*C*H$_3$CH, D1), 17.8, (*C*H$_3$CH, D2), 18.9 (*C*H$_3$CH, D1), 19.0 (*C*H$_3$CH, D2), 28.1 (SCH$_2$*C*H$_2$, D1), 28.1 (SCH$_2$*C*H$_2$, D2), 30.9 (S*C*H$_2$, D1), 31.0 (S*C*H$_2$, D2), 31.2 ((CH$_3$)$_2$*C*H, D1), 31.3 ((CH$_3$)$_2$*C*H, D2), 52.3 (OCH$_3$, D1+D2), 53.6 (N*C*H, D1), 53.7 (N*C*H, D2), 57.4 (NH*C*H, D2), 57.5 (NH*C*H, D1), 123.8 (Ar *C*H, D1), 123.8 (Ar *C*H, D2), 131.6 (Ar *C*, D1+D2), 134.5 (Ar *C*H, D2), 134.5 (Ar *C*H, D1), 168.1 (*C*O, D1+D2), 168.5 (*C*O, D1+D2), 172.1 (*C*O, D2), 172.2 (*C*O, D1). **v_{max}** (thin film/cm^{-1}): 3361, 2960, 2922, 2856, 1775, 1718, 1681, 1532, 1468, 1384, 1305, 1089, 795. **HRMS** $C_{16}H_{19}O_5N_2S$ (M+H$^+$) predicted 351.1009, found 351.0999.

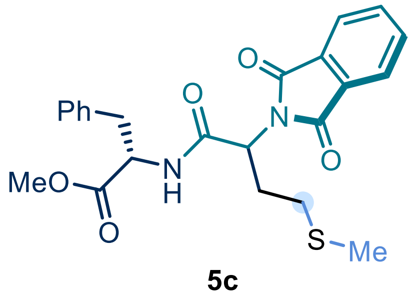

5c

Methyl (2-(1,3-dioxoisoindolin-2-yl)-4-(methylthio)butanoyl)-*L*-phenylalaninate (5c)

5c was prepared according to general method a, using **11c** (37.8 mg, 0.1 mmol) and dimethyl sulfide (62.1 μL, 1.0 mmol). The crude product was purified using column chromatography (60% EtOAc: Hexane) to give an off-white oil as a mixture of inseparable diastereomers (15.4 mg, 35%, 0.035 mmol, dr 1:1). **^{1}H NMR** (400 MHz, CDCl$_3$) δ 2.04 (s, 3H, SC*H*$_3$, D1), 2.04 (s, 3H, SC*H*$_3$, D2), 2.30 – 2.66 (m, 8H, SC*H$_2$* + SCH$_2$C*H*$_2$, D1+D2), 3.02 – 3.29 (m, 4H, PhC*H*$_2$, D1+D2), 3.71 (s, 3H, OC*H*$_3$, D1), 3.72 (s, 3H, OC*H*$_3$, D2), 4.81 – 4.90 (m, 2H, NHC*H,* D1+D2), 4.93 – 5.00 (m, 2H, NC*H,* D1+D2), 6.43 (d, *J* = 7.7 Hz, 1H, N*H*, D1), 6.59 (d, *J* = 7.5 Hz, 1H, N*H*, D2), 6.98 – 7.06 (m, 3H, Ar C*H*), 7.07 – 7.18 (m, 7H, Ar C*H*), 7.73 – 7.80 (m, 4H, Ar C*H*), 7.83 – 7.90 (m, 4H, Ar C*H*). **^{13}C NMR** (101 MHz, CDCl$_3$) δ 14.4 (S*C*H$_3$, D1+D2) , 26.6 (SCH$_2$*C*H$_2$, D1), 26.9 (SCH$_2$*C*H$_2$, D1), 29.9 (S*C*H$_2$, D1+D2), 36.5 (Ph*C*H$_2$, D2), 36.7 (Ph*C*H$_2$, D1), 51.4 (O*C*H$_3$, D1+D2), 52.2 (NH*C*H, D1+D2), 52.3 (N*C*H, D1), 52.6 (N*C*H, D2), 122.7 (Ar *C*H, D1+D2), 126.1 (Ar *C*H, D1+D2), 127.5 (Ar *C*H, D1+D2), 128.1 (Ar *C*H, D1), 128.2 (Ar *C*H, D2), 130.6 (Ar *C*, D1+D2), 133.4 (Ar *C*H, D1+D2), 134.5 (Ar *C*, D1+D2), 166.8 (*C*O, D1+D2), 166.9 (*C*O,

D1+D2), 170.6 (*C*O, D1+D2). **ν_{max}** (thin film/cm^{-1}): 3340, 2922, 2852, 1774, 1717, 1525, 1255, 1382, 1177, 1087, 720, 530. **HRMS** $C_{23}H_{25}O_5N_2S$ (M+H$^+$) predicted 441.1479, found 441.1469.

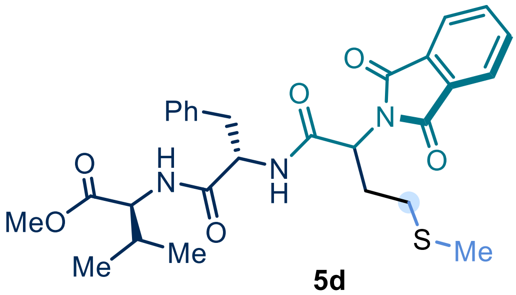

5d

Methyl (2-(1,3-dioxoisoindolin-2-yl)-4-(methylthio)butanoyl)-*L*-phenylalanyl-*L*-valinate (5d)

5d was prepared according to general method a, using **11e** (47.8 mg, 0.1 mmol) and dimethyl sulfide (62.1 μL, 1.0 mmol). The crude product was purified using column chromatography (70% EtOAc: Hexane) to give an off-white oil as a mixture of inseparable diastereomers (20.3 mg, 36%, 0.036 mmol, dr 1:1.3). **ν_{max}** (thin film/cm^1): **^{1}H NMR** (400 MHz, $CDCl_3$) δ 0.76 – 0.89 (m, 13.8H, CH(C*H*$_3$)$_2$, D1+D2), 2.04 (s, 6.9H, SC*H*$_3$, D1+D2), 2.05 – 2.15 (m, 2.3H, C*H*(CH$_3$)$_2$, D1+D2), 2.39 – 2.51 (m, 4.6H, PhC*H*$_2$, D1+D2), 2.95 – 3.33 (m, 9.2H, SC*H*$_2$C*H*$_2$, D1+D2), 3.67 (s, 3H, OC*H*$_3$, D1), 3.70 (s, 3.9H, OC*H*$_3$, D2), 4.35 – 4.46 (m, 2.3H, C*H*CH(CH$_3$)$_2$, D1+D2), 4.55 – 4.72 (m, 2.3H, COC*H*NPhth, D1+D2), 4.89 – 5.02 (m, 2.3H, PhCH$_2$C*H*, D1+D2), 6.11 (d, *J* = 8.6 Hz, 1.3H, N*H*, D2), 6.26 (d, *J* = 8.4 Hz, 1H, N*H,* D1), 6.33 (d, *J* = 8.5 Hz, 1.3H, N*H,* D2), 6.60 (d, *J* = 7.6 Hz, 1.3H, N*H,* D2), 6.70 (d, *J* = 7.4 Hz, 1H, N*H,* D1), 7.00 – 7.20 (m, 12H, Ar C*H*, D1+D2), 7.20 – 7.33 (m, 8H, Ar C*H*, D1+D2), 7.50 (d, *J* = 8.2 Hz, 1H), 7.73 – 7.79 (m, 4.6H, Ar C*H*, D1+D2), 7.85 (td, *J* = 5.1, 3.0 Hz, 4.6H, Ar C*H*, D1+D2). **^{13}C NMR** (101 MHz, $CDCl_3$) δ 15.4 (2 × SCH$_3$), 17.6 – 17.9 (m, CH(*C*H$_3$)$_2$, D1), 18.8 – 18.9 (m, CH(*C*H$_3$)$_2$, D2), 27.8 (Ph*C*H$_2$, D1), 27.9 (Ph*C*H$_2$, D2), 30.8 (*C*H(CH$_3$)$_2$, D1), 30.9 (*C*H(CH$_3$)$_2$, D2), 37.4 (SCH$_2$*C*H$_2$, D1), 37.6 (SCH$_2$*C*H$_2$, D2), 38.2 (S*C*H$_2$, D2+D2), 52.1 (O*C*H$_3$, D2), 52.2 (O*C*H$_3$, D1), 53.1 (PhCH$_2$*C*H, D1), 53.4 (PhCH$_2$*C*H, D2), 54.3 (*C*HNPhth, D1), 54.8 (*C*HNPhth, D2), 57.4 (CO*C*HCH, D1), 57.6 (CO*C*HCH, D2), 123.6 (Ar *C*H, D1+D2), 127.0 (Ar *C*H, D2), 127.3 (Ar *C*H, D1), 128.6 (Ar *C*H, D2), 128.8 (Ar *C*H, D1), 129.3 (Ar *C*H, D1+D2), 131.6 (Ar *C*, D1+D2), 134.3 (Ar *C*H, D1), 134.4 (Ar *C*H, D2), 135.9 (Ar *C*, D1), 136.0 (Ar *C*, D2), 167.8 (*C*O, D1+D2), 168.5 (*C*O, D1+D2), 170.3 (*C*O, D1+D2), 171.6 (*C*O, D1+D2). **HRMS** $C_{28}H_{33}O_6N_3SNa$ (M+H$^+$) predicted 562.1982, found 562.2003.

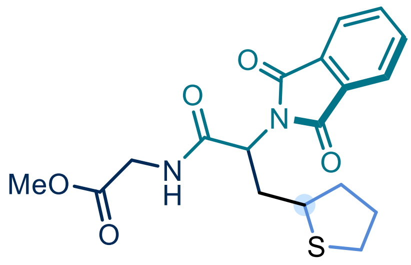

5e

Methyl (2-(1,3-dioxoisoindolin-2-yl)-3-(tetrahydrothiophen-2-yl)propanoyl)glycinate (5e)

5e was prepared according to general method a, using **11a** (28.8 mg, 0.1 mmol) and tetrahydrothiophene (88.1 µL, 1.0 mmol). The crude product was purified using column chromatography (60% EtOAc: Hexane) to give an off-white oil as a mixture of inseparable diastereomers (14.3 mg, 0.038 mmol, 38%, dr 1:2.5). **^{1}H NMR** (500 MHz, $CDCl_3$) δ 1.59 – 1.64 (m, 3.5H, $SCH_2CH_2CH_aH_b$, D2 + $SCH_2CH_aH_b$, D1), 1.64 – 1.73 (m, 1H, $SCH_2CH_2CH_aH_b$, D1), 1.75 – 1.86 (m, 2.5H, $SCH_2CH_aH_b$, D2), 1.87 – 1.96 (m, 1H, $SCH_2CH_aH_b$, D1), 2.00 – 2.11 (m, 3.5H, $SCH_2CH_2CH_aH_b$, D1 + $SCH_2CH_aH_b$, D2), 2.13 – 2.24 (m, 3.5H, $NCHCH_aH_b$, D1 + $SCH_2CH_2CH_aH_b$, D2), 2.43 – 2.55 (m, 2.5H, $NCHCH_aH_b$, D2), 2.64 (dt, *J* = 14.3, 5.5 Hz, 2.5H, $NCHCH_aH_b$, D2), 2.73 – 2.86 (m, 6H, SCH_2, D2 + SCH_aH_b, D1), 2.86 – 2.93 (m, 2H, SCH_aH_b, D1 + $NCHCH_aH_b$, D1), 3.19 – 3.26 (m, 1H, SC*H*, D1), 3.27 – 3.36 (m, 2.5H, SC*H*, D2), 3.73 (s, 3H, OCH_3, D1), 3.74 (s, 7.5H, OCH_3, D2), 4.05 (dd, *J* = 5.0, 2.7 Hz, 7H, NCH_2, D1+D2), 4.93 (dd, *J* = 10.1, 5.1 Hz, 2.5H, NC*H*, D2), 5.02 (dd, *J* = 11.3, 4.5 Hz, 1H, NC*H*, D1), 6.54 (s, 1H, N*H*, D1), 6.73 (s, 2.5H, N*H*, D2), 7.74 – 7.79 (m, 7H, Ar C*H*, D1+D2), 7.85 – 7.92 (m, 7H, Ar C*H*, D1+D2). **^{13}C NMR** (126 MHz, $CDCl_3$) δ 29.7 (SCH_2CH_2, D2), 29.8 (SCH_2CH_2, D1), 32.4 (SCH_2, D1), 32.7 (SCH_2, D2), 36.1 ($NCHCH_2$, D2), 36.5 ($NCHCH_2$, D1), 37.2 ($SCH_2CH_2CH_2$, D1), 37.5 ($SCH_2CH_2CH_2$, D2), 41.5 (NCH_2, D1+D2), 45.6 (S*C*H, D1), 46.2 (S*C*H, D2), 52.5 (OCH_3, D1+D2), 54.0 (N*C*H, D1), 54.2 (N*C*H, D2), 123.8 (Ar *C*H, D1+D2), 131.7 (Ar *C*, D1), 131.8 (Ar *C*, D2), 134.4 (Ar *C*H, D1), 134.4 (Ar *C*H, D2), 168.1 (*C*O, D1+D2), 168.7 (*C*O, D1+D2), 170.0 (*C*O, D1+D2). **v_{max}** (thin film/cm^{-1}): 3340, 2924, 2853, 1714, 1533, 1439, 1838, 1208, 1086, 720, 530, 457. **HRMS**: $C_{18}H_{21}O_5N_2S$ (M+H$^+$) predicted 377.1166, found 377.1148.

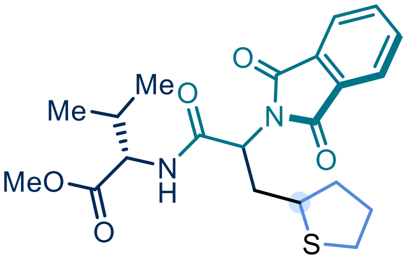

5f

Methyl (2-(1,3-dioxoisoindolin-2-yl)-3-(tetrahydrothiophen-2-yl)propanoyl)-*L*-valinate (5f)

5f was prepared according to general method a, using **11b** (33.0 mg, 0.1 mmol) and tetrahydrothiophene (88.1 µL, 1.0 mmol). The crude product was purified using column chromatography (60% EtOAc: Hexane) to give an off-white oil as a mixture of four inseparable diastereomers (12.9 mg, 0.031 mmol, 31%, dr 2:2:1:1). Note: Only one major and one minor diastereoisomers reported for simplicity. **^{1}H NMR** (400 MHz, $CDCl_3$) δ 0.88 – 0.96 (m, 18H, CH(C*H*$_3$)$_2$, D1+D2), 1.58 – 1.74 (m, 4H, SCH$_2$C*H*$_a$H$_b$, D1 + SCH$_2$CH$_2$C*H*$_a$H$_b$, D1+D2), 1.74 – 1.87 (m, 2H, SCH$_2$C*H*$_a$H$_b$, D2), 1.87 – 1.98 (m, 1H, SCH$_2$C*H*$_a$H$_b$, D1), 2.01 – 2.09 (m, 3H, SCH$_2$CH$_a$*H*$_b$, D2 + SCH$_2$CH$_2$CH$_a$*H*$_b$, D1), 2.11 – 2.25 (m, 6H, C*H*(CH$_3$)$_2$, D1+D2, NCHC*H*$_a$H$_b$, D1 + SCH$_2$CH$_2$CH$_a$*H*$_b$, D2), 2.43 – 2.67 (m, 4H, NCHC*H*$_2$, D2), 2.73 – 2.95 (m, 7H, SC*H*$_2$, D1+D2 + NCHCH$_a$*H*$_b$, D1), 3.18 – 3.39 (m, 3H, SC*H*, D1+D2), 3.65 – 3.76 (m, 9H, OC*H*$_3$, D1+D2), 4.48 – 4.60 (m, 3H, N*H*CH, D1+D2), 4.92 (dd, *J* = 10.0, 5.2 Hz, 2H, NC*H*, D2), 5.01 (dt, *J* = 11.1, 4.2 Hz, 1H, NC*H*, D1), 6.54 (d, *J* = 8.6 Hz, 1H, N*H*, D1), 6.73 (t, *J* = 9.1 Hz, 2H, N*H*, D2), 7.70 – 7.79 (m, 6H, Ar C*H*, D1+D2), 7.82 – 7.94 (m, 6H, Ar C*H*, D1+D2). **^{13}C NMR** (101 MHz, $CDCl_3$) δ 17.7 ((*C*H$_3$)$_2$CH, D2), 18.9 ((*C*H$_3$)$_2$CH, D1), 29.6 (SCH$_2$*C*H$_2$, D1), 29.8 (SCH$_2$*C*H$_2$, D1), 31.1 ((CH$_3$)$_2$*C*H, D2), 31.2 ((CH$_3$)$_2$*C*H, D1), 32.6 (S*C*H$_2$, D1+D2), 36.0 (SCH*C*H$_2$CHN, D1+D2), 37.4 (SCH$_2$CH$_2$*C*H$_2$, D1+D2), 46.0 (S*C*H, D1), 46.2 (S*C*H, D1), 52.2 (O*C*H$_3$, D1+D2), 54.2 (CO*C*HNPhth, D1+D2), 57.3 (CO*C*HCH, D1), 57.5 (CO*C*HCH, D2), 123.7 (Ar *C*H, D2), 123.7 (Ar *C*H, D1), 131.6 (Ar *C*, D1), 131.7 (Ar *C*, D2), 134.3 (Ar *C*H, D2), 134.4 (Ar *C*H, D1), 168.0 (2 × *C*O, D1+D2), 172.0 (*C*O, D1+D2). **ν_{max}** (thin film/cm^{-1}): 3368, 2969, 2926, 1716, 1513, 1438, 1382, 1264, 1209, 1087, 895, 736, 705. **HRMS** $C_{21}H_{27}O_5N_2S$ ($M+H^+$) predicted 419.1635, found 419.1622.

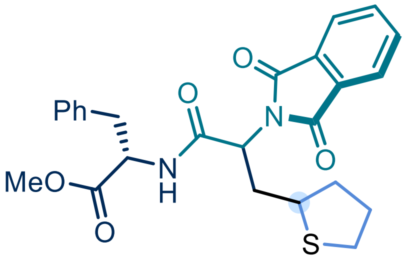

5g

Methyl (2-(1,3-dioxoisoindolin-2-yl)-3-tetrahydrothiophen-2-yl)propanoyl)-*L*-phenylalaninate (5g)

5g was prepared according to general method a, using **11c** (37.8 mg, 0.1 mmol) and tetrahydrothiophene (88.1 µL, 1.0 mmol). The crude product was purified using column chromatography (60% EtOAc: Hexane) to give an off-white oil as a mixture of inseparable diastereomers (17.2 mg, 37%, 0.037 mmol, dr approx. 1:1:1:1). **^{1}H NMR** (500 MHz, $CDCl_3$) δ 1.61 – 2.20 (m, 16H, $SCH_2CH_2CH_2$ + SCH_2CH_2), 2.29 – 2.63 (m, 8H, $SCHCH_2CHN$), 2.72 – 2.92 (m, 8H, SCH_2), 3.02 – 3.29 (m, 12H, SCH + CH_2Ph), 3.64 – 3.80 (m, 12H, OCH_3), 4.79 – 4.98 (m, 8H, CO*CH*NH + CO*CH*NPhth), 6.35 (d, *J* = 7.8 Hz, 1H, N*H*), 6.48 (d, *J* = 7.7 Hz, 2H, N*H*), 6.69 (d, *J* = 7.6 Hz, 1H, N*H*), 6.96 – 7.20 (m, 20H, Ar C*H*), 7.71 – 7.81 (m, 8H, Ar C*H*), 7.81 – 7.88 (m, 8H, Ar C*H*). **^{13}C NMR** (126 MHz, $CDCl_3$) δ 29.8 ($SCH_2CH_2CH_2$), 30.0 ($SCH_2CH_2CH_2$), 32.4 (SCH_2), 32.6 (SCH_2), 35.8 ($SCHCH_2CHN$), 37.2 (SCH_2CH_2), 37.4 ($PhCH_2$), 37.5 ($PhCH_2$), 37.7, 46.2 (S*C*H), 52.4 (OCH_3), 53.3 (N*C*H), 53.4 (N*C*H), 53.9 (N*C*H), 54.4 (N*C*H), 123.7 (Ar *C*H), 127.1 (Ar *C*), 128.5 (Ar *C*H), 129.2 (2 × Ar *C*H), 131.8 (Ar *C*), 134.3 (Ar *C*H), 167.9 (*C*O), 168.0 (*C*O), 171.6 (*C*O). **ν_{max}** (thin film/cm^{-1}): 3358, 2958, 1776, 1715, 1687, 1516, 1438, 1382, 1264, 1209, 1086, 1016, 874, 801, 733, 703, 530. **HRMS** $C_{25}H_{27}O_5N_2S$ ($M+H^+$) predicted 467.1635, found 467.1617.

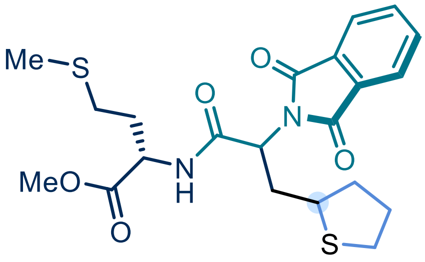

5h

Methyl (2-(1,3-dioxoisoindolin-2-yl)-3-(tetrahydrothiophen-2-yl)propanoyl)-*L*-methioninate (5h)

5h was prepared according to general method a, using **11d** (36.1 mg, 0.1 mmol) and tetrahydrothiophene (88.1 µL, 1.0 mmol). The crude product was purified using column chromatography (60% EtOAc: Hexane) to give an off-white oil as a mixture of inseparable diastereomers (14.4 mg, 32%, 0.032 mmol, dr 2:2:1:1. **^{1}H NMR** (400 MHz, $CDCl_3$) δ 1.52 – 1.73 (m, 2H), 1.73 – 1.88 (m, 4H), 1.90 – 2.08 (m, 12H), 2.00 – 2.05 (4 x s, 18H, SCH_3), 2.10 – 2.25 (m, 20H), 2.41 – 2.53 (m, 18H), 2.56 – 2.72 (m, 4H), 2.73 – 2.95 (m, 12H), 3.16 – 3.25 (m, 2H, SC*H*), 3.26 – 3.36 (m, 4H, SC*H*), 3.71 – 3.75 (m, 18H, OCH_3), 4.64 – 4.75 (m, 6H, NHC*H*), 4.91 (dd, *J* = 10.3, 4.9 Hz, 4H, NC*H*), 4.97 – 5.05 (m, 2H, NC*H*), 6.76 (d, *J* = 7.6 Hz, 1H, N*H*), 6.81 (d, *J* = 7.6 Hz, 1H, N*H*), 6.90 (d, *J* = 7.6 Hz, 2H, N*H*), 6.97 (d, *J* = 7.6 Hz, 2H, N*H*), 769 – 7.80 (m, 12H, Ar C*H*), 7.80 – 7.93 (m, 12H, Ar C*H*). **^{13}C NMR** (101 MHz, $CDCl_3$) δ 15.4, 29.7, 29.8, 30.0, 31.0, 31.1, 32.4, 32.6, 35.9, 37.2, 37.5, 45.6, 46.1 (S*C*H), 46.2 (S*C*H), 52.3 (NH*C*H), 52.6 (O*C*H_3), 53.9 (N*C*H), 123.7 (Ar *C*H), 131.8 (Ar *C*), 134.3 (Ar *C*H), 167.9 (*C*O), 168.3 (*C*O), 171.9 (*C*O). **HRMS** $C_{21}H_{26}O_5N_2S_2Na$ ($M+Na^+$) predicted 473.1175, found 473.1166.

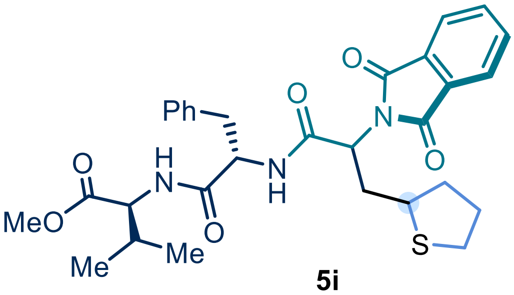

5i

Methyl (2-(1,3-dioxoisoindolin-2-yl)-3-(tetrahydrothiophen-2-yl)propanoyl)-*L*-phenylalanyl-*L*-valinate (5i)

5i was prepared according to general method a, using **11e** (47.8 mg, 0.1 mmol) and tetrahydrothiophene (88 µL, 1.0 mmol). The crude product was purified using column chromatography (70% EtOAc: Hexane) to give an off-white oil as a mixture of four inseparable diastereomers (18.9 mg,

35%, 0.035 mmol, approx. dr 3:1:1:1). **^{1}H NMR** (400 MHz, $CDCl_3$) δ 0.84 – 1.00 (m, 36H, $CH(CH_3)_2$), 1.50 – 2.73 (m, 48H, $CH(CH_3)_2$ + SCH_2CH_2 + $SCH_2CH_2CH_2$ + $PhthNCHCH_2$), 2.78 – 2.99 (m, 12H, SCH_2), 3.03 – 3.41 (m, 18H, SC*H* + $PhCH_2$), 3.72 – 3.80 (m, 18H, OCH_3), 4.42 – 5.10 (m, 18H, 2 × NHC*H* + C*H*NPhth), 6.22 (d, *J* = 8.5 Hz, 2H), 6.39 (dd, *J* = 9.8, 6.0 Hz, 3H, N*H*), 6.45 (d, *J* = 8.5 Hz, 1H, N*H*), 6.63 (d, *J* = 7.5 Hz, 1H, N*H*), 6.71 (d, *J* = 7.7 Hz, 1H, N*H*), 6.76 (d, *J* = 7.5 Hz, 1H, N*H*), 6.86 (d, *J* = 7.4 Hz, 1H, N*H*), 7.06 – 7.41 (m, 30H, Ar C*H*), 7.59 (d, *J* = 8.2 Hz, 2H, N*H*), 7.79 – 7.88 (m, 12H, Ar C*H*), 7.89 – 7.96 (m, 12H, Ar C*H*). **^{13}C NMR** (101 MHz, $CDCl_3$) δ 17.8 (m), 18.8 (m), 27.7, 29.6 – 29.9 (m), 30.0, 31.0, 31.2, 32.4, 32.6, 36.0, 36.2, 36.4, 37.2, 37.4, 37.5, 37.8, 38.2, 40.6, 45.5, 46.0, 46.2, 52.1, 52.0, 53.8, 54.0, 54.2, 54.3, 54.8, 57.4, 57.5, 57.6, 123.7, 124.0, 127.0, 127.3, 128.6, 128.6, 128.8, 128.8, 129.6, 131.6, 131.6, 131.7, 134.4, 134.6, 135.9, 136.0, 136.3, 159.9, 167.9, 168.4, 168.6. **HRMS** $C_{30}H_{35}O_6N_3SNa$ ($M+Na^+$) predicted 588.2139, found 588.2161.

N-Protecting group compatibility in dehydroamino acid substrates

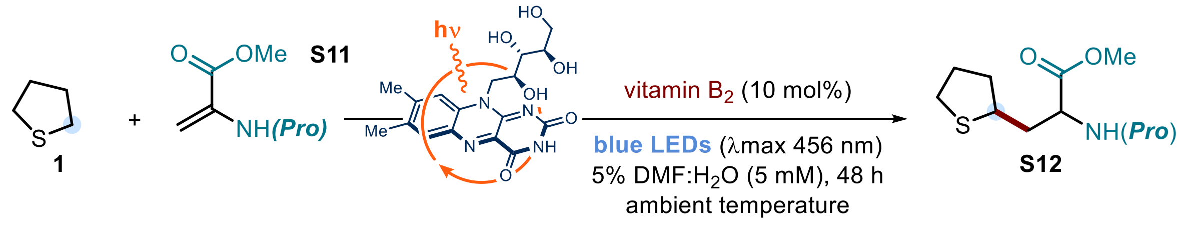

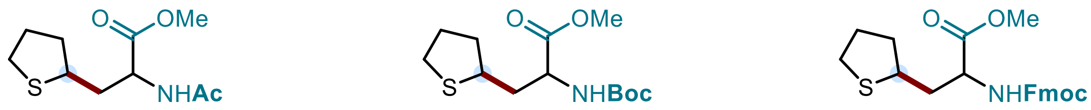

S12a (63% yield)
1.3:1 dr

S12b (19% yield)
1:1 dr

S12c (<5% yield*)

Figure S1: N-Protecting group compatibility in dehydroamino acid substrates. Reaction conditions: tetrahydrothiophene (3.0 equiv), Alkene (1.0 equiv.), riboflavin (10 mol%) in water (19 mL) and DMF (1.0 mL) with blue Kessel LED lamps. Reactions performed on a 0.1 mmol scale. *NMR yield determined by ^{1}H-NMR spectroscopy using $MeNO_2$ as an internal standard.

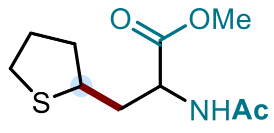

S12a

Ethyl 2-acetamido-3-tetrahydrothiophen-2-yl)propanoate (S12a)

S12a was prepared according to general method a, using ethyl 2-acetamidoacrylate (15.7 mg, 0.1 mmol) and tetrahydrothiophene (88.1 μL, 1.0 mmol). The crude product was purified using column chromatography (80% EtOAc: Hexane) to give an off-white amorphous solid (14.5 mg, 0.063 mmol, 63%, dr: 1:1.5). **^{1}H NMR** (400 MHz, $CDCl_3$) δ 1.53 – 1.64 (m, 2.5H, + $SCH_2CH_2CH_aH_b$, D1+D2), 1.81 – 1.94 (m, 2.5H, $SCH_2CH_aH_b$, D1+D2), 2.02 (s, 3H, $COCH_3$, D1), 2.04 (s, 4.5H, $COCH_3$, D2), 2.04 – 2.27 (m, 10H, $SCH_2CH_aH_b$, D1+D2 + $SCH_2CH_2CH_aH_b$, D1+D2 + $NHCH_2CH_2$, D1+D2), 2.80 – 2.93 (m, 5H, SCH_2, D1+D2), 3.28 – 3.40 (m, 2.5H, SC*H*, D1+D2), 3.75 (s, 4.5H, OCH_3, D2), 3.75 (s, 3H, OCH_3, D1), 4.62 – 4.71 (m, 2.5H, NC*H*, D1+D2), 6.06 (d, J = 7.2 Hz, 1.5H, N*H*, D2), 6.12 (d, J = 8.1 Hz, 1H, N*H*, D1). **^{13}C NMR** (101 MHz, $CDCl_3$) δ 23.2 ($COCH_3$, D1), 23.2 ($COCH_3$, D2), 29.9 (SCH_2CH_2, D1), 30.0

(SCH_2CH_2, D2), 32.4 (SCH_2, D2), 32.6 (SCH_2, D1), 37.4 (2 × $SCH_2CH_2CH_2$, D1+D2), 40.0 ($NHCH_2CH_2$, D1), 40.3 ($NHCH_2CH_2$, D2), 44.8 (S*C*H, D2), 44.9 (S*C*H, D1), 51.7 (NH*C*H, D2), 51.9 (NH*C*H, D1), 52.5 (2 × OCH_3, D1+D2), 169.7 (*C*O, D1), 169.8 (*C*O, D2), 172.6 (*C*O, D2), 172.7 (*C*O, D1). **HRMS** $C_{10}H_{17}O_3NSNa$ ($M+Na^+$) predicted 254.0821, found 254.0814.

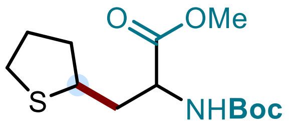

S12a

Methyl 2-((*tert*-butoxycarbonyl)amino)-3-tetrahydrothiophen-2-yl)propanoate (S12b)

S12b was prepared according to general method a, using methyl 2-((*tert*-butoxycarbonyl)amino)acrylate (20.1 mg, 0.1 mmol) and tetrahydrothiophene (88.1 μL, 1.0 mmol). The crude product was purified using column chromatography (60% EtOAc: Hexane) to give an off-white amorphous solid (5.4 mg, 0.019 mmol, 19%, dr: 1:1). **^{1}H NMR** (400 MHz, $CDCl_3$) δ 1.44 (s, 18H, $C(CH_3)_3$, D1+D2), 1.57 – 1.67 (m, 2H, $SCH_2CH_2CH_aH_b$, D1+D2), 1.81 – 2.26 (m, 10H, $SCH_2CH_2CH_aH_b$, D1+D2 + $NHCH_2CH_2$, D1+D2 + SCH_2CH_2, D1+D2), 2.79 – 2.93 (m, 4H, SCH_2, D1+D2), 3.34 – 3.47 (m, 2H, SC*H*, D1+D2), 3.74 (2 × s, 6H, OCH_3, D1+D2), 4.30 – 4.43 (m, 2H, N*H*CH, D1+D2), 4.35 (s, 1H, N*H*, D2), 5.06 (s, 1H, N*H*, D1). **^{13}C NMR** (101 MHz, $CDCl_3$) δ 27.3 ($C(CH_3)_3$, D1+D2), 28.7 ($C(CH_3)_3$, D1+D2), 29.0 (SCH_2CH_2, D1), 29.1 (SCH_2CH_2, D2), 31.3 (SCH_2, D1), 31.5 (SCH_2, D2), 36.2 (SCH_2CH-$_2CH_2$, D1), 36.3 ($SCH_2CH_2CH_2$, D2), 39.7 ($NHCH_2CH_2$, D1+D2), 43.7 (S*C*H, D2), 44.0 (S*C*H, D1), 51.3 (OCH_3, D1), 51.4 (OCH_3, D2), 52.0 (NH*C*H, D1+D2), 154.3 (2 × *C*O, D1+D2), 171.7 (2 × *C*O, D1+D2). **HRMS** $C_{13}H_{23}O_4NSNa$ ($M+Na^+$) predicted 312.1240, found 312.1238.

Unsuccessful Examples

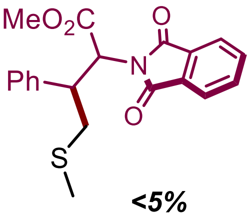

<5%

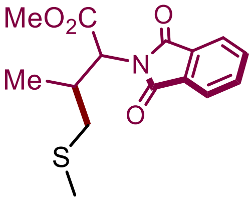

<5%

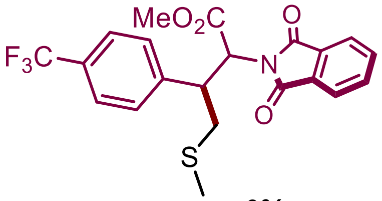

0%

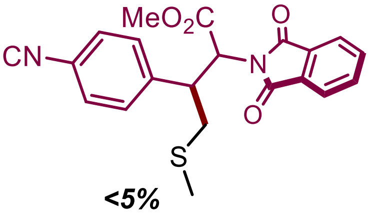

<5%

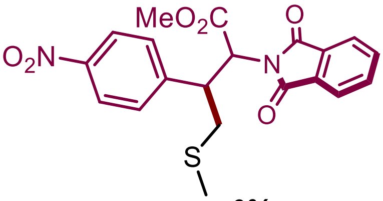

0%

Mechanistic Studies

Deuterium labelling studies

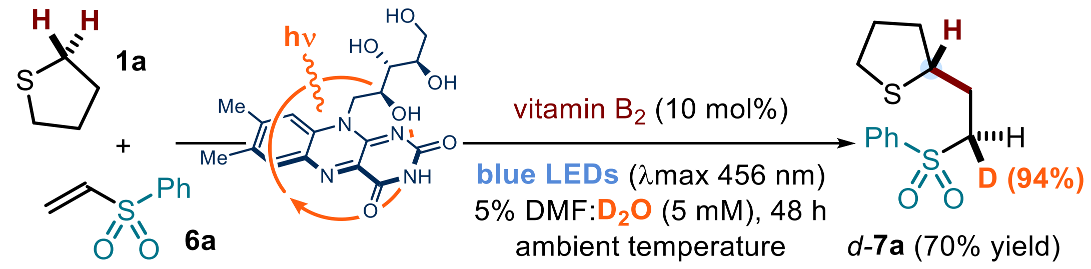

A 40 mL oven-dried Schlenk tube with a magnetic stirrer bar was charged with riboflavin (3.7 mg, 0.01 mmol, 0.1 equiv.), phenyl vinyl sulfone (0.1 mmol, 1 equiv.) and tetrahydrothiophene (1 mmol, 10 equiv.) and subjected to a nitrogen atmosphere. The reagents were dissolved in degassed D_2O (19 mL) and dry DMF (1 mL), and irradiated with blue LEDs for 48 h. After completion, the reaction mixture was extracted in to CH_2Cl_2 (3 × 50 mL), washed with 10% LiCl solution (3 × 50 mL), dried over $MgSO_4$ and evaporated under reduced pressure. The crude residue was purified using column chromatography (20% Ethyl Acetate / Hexane), to give a colourless oil (17.9 mg, 0.07 mmol, 70%), with a deuterium incorporation of 94%, determined by ^{1}H NMR.

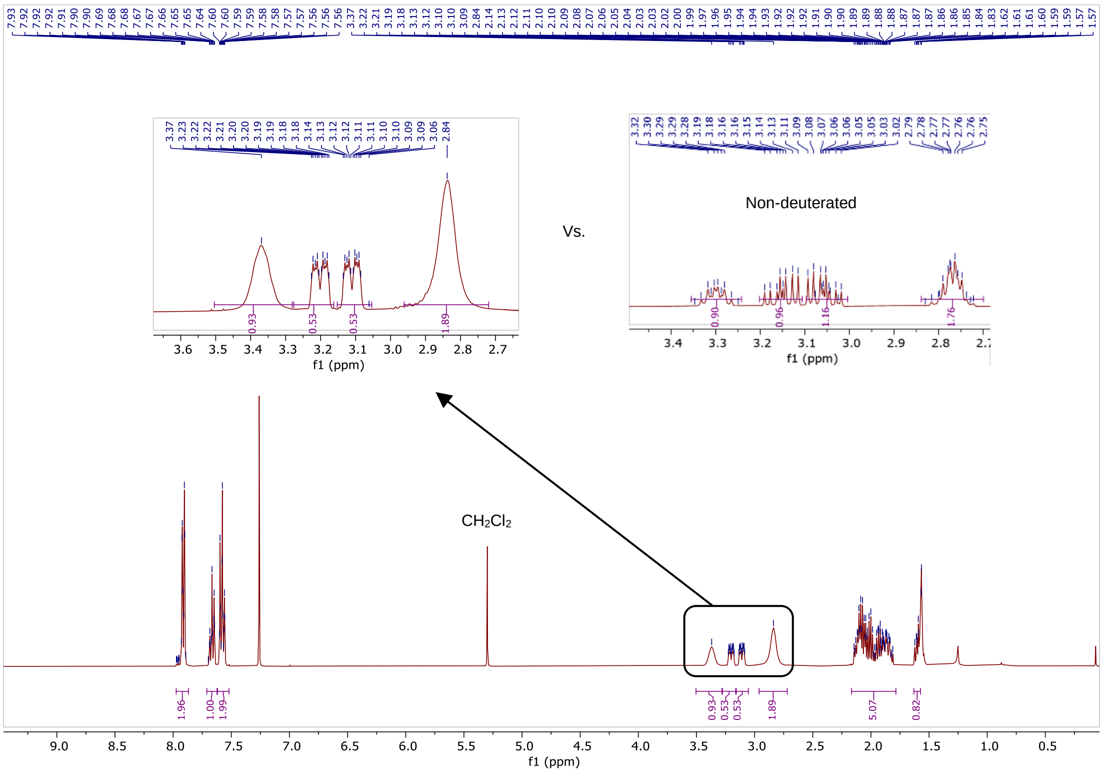

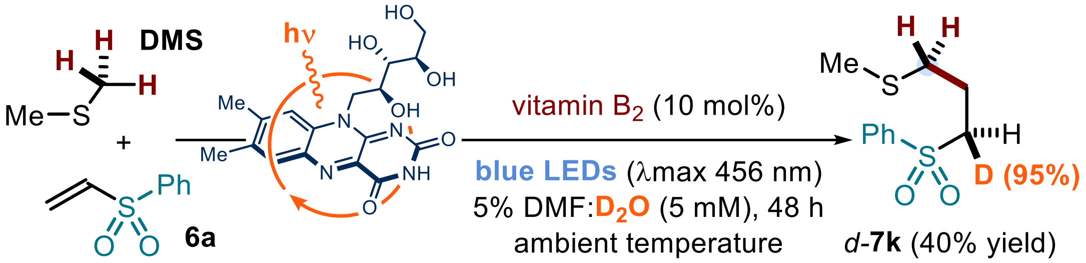

A 40 mL oven-dried Schlenk tube with a magnetic stirrer bar was charged with riboflavin (3.7 mg, 0.01 mmol, 0.1 equiv.), phenyl vinyl sulfone (0.1 mmol, 1 equiv.) and dimethyl sulfide (1 mmol, 10 equiv.) and subjected to a nitrogen atmosphere. The reagents were dissolved in degassed D_2O (19 mL) and dry DMF (1 mL), and irradiated with blue LEDs for 48 h. After completion, the reaction mixture was extracted in to CH_2Cl_2 (3 × 50 mL), washed with 10% LiCl solution (3 × 50 mL), dried over $MgSO_4$ and evaporated under reduced pressure. The crude residue was purified using column chromatography (20% Ethyl Acetate / Hexane), to give a colourless oil (9.2 mg, 0.04 mmol, 40**%**), with a deuterium incorporation of 95%, determined by ^1H NMR.

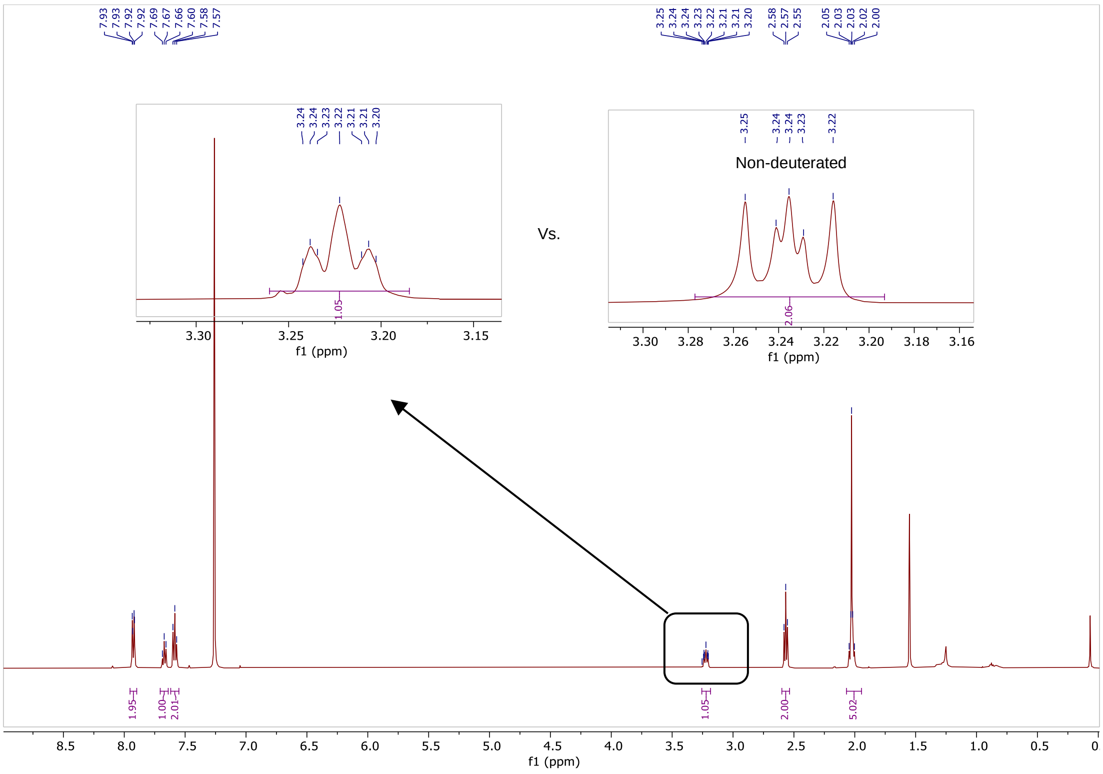

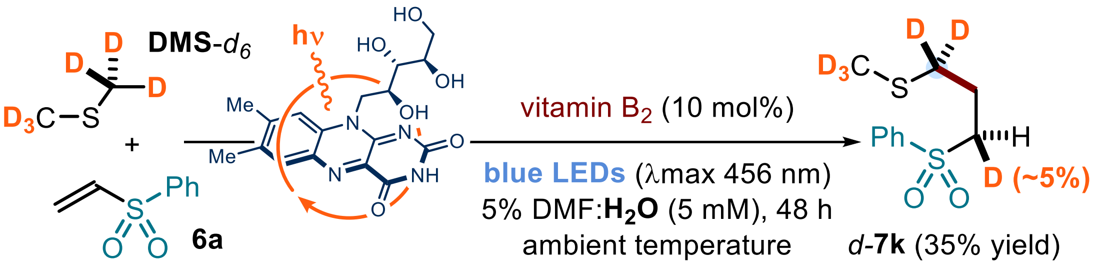

A 40 mL oven-dried Schlenk tube with a magnetic stirrer bar was charged with riboflavin (3.7 mg, 0.01 mmol, 0.1 equiv.), phenyl vinyl sulfone (0.1 mmol, 1 equiv.) and D6-dimethyl sulfide (1 mmol, 10 equiv.) and subjected to a nitrogen atmosphere. The reagents were dissolved in degassed H_2O (19 mL) and dry DMF (1 mL), and irradiated with blue LEDs for 48 h. After completion of the reaction, the reaction mixture was extracted in to CH_2Cl_2 (3 × 50 mL), washed with 10% LiCl solution (3 × 50 mL), dried over $MgSO_4$ and evaporated under reduced pressure. The crude residue was purified using column chromatography (20% Ethyl Acetate / Hexane), to give a colourless oil (8.2 mg, 0.035 mmol, 35%), with a deuterium incorporation of 5%, determined by ^{1}H NMR.

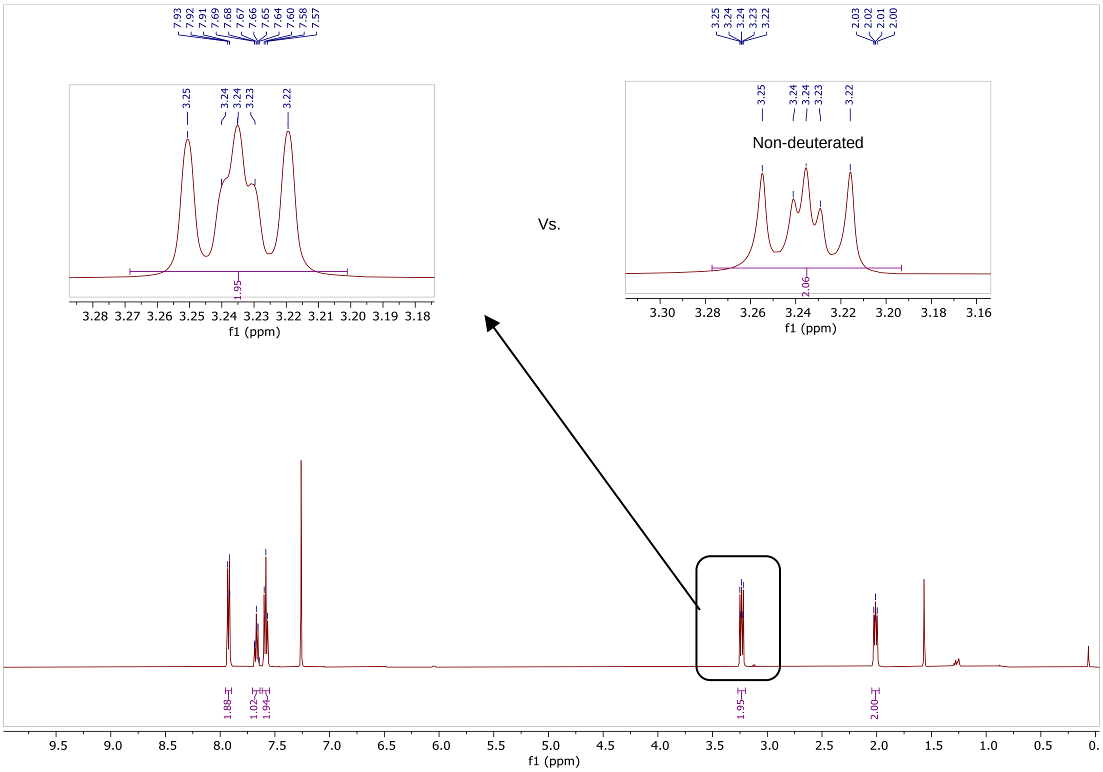

Deuterium Control Experiment

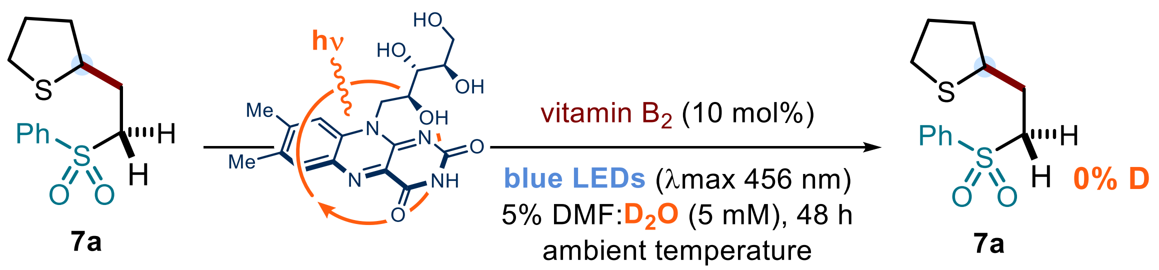

UV/Vis Spectroscopy

UV/Vis analyses were conducted on a Mettler Toledo UV5Bio Spectrophotometer using a 1 cm path length quartz cuvette.

Absorption spectra of each of the individual components were run at a concentration of 0.05 mM (5% DMF:H_2O), followed by mixtures of different components in the reaction mixture in an attempt to show any shift in absorbance caused by pre-association, or the formation of an EDA complex between the photocatalyst and reaction components prior to photoexcitation.

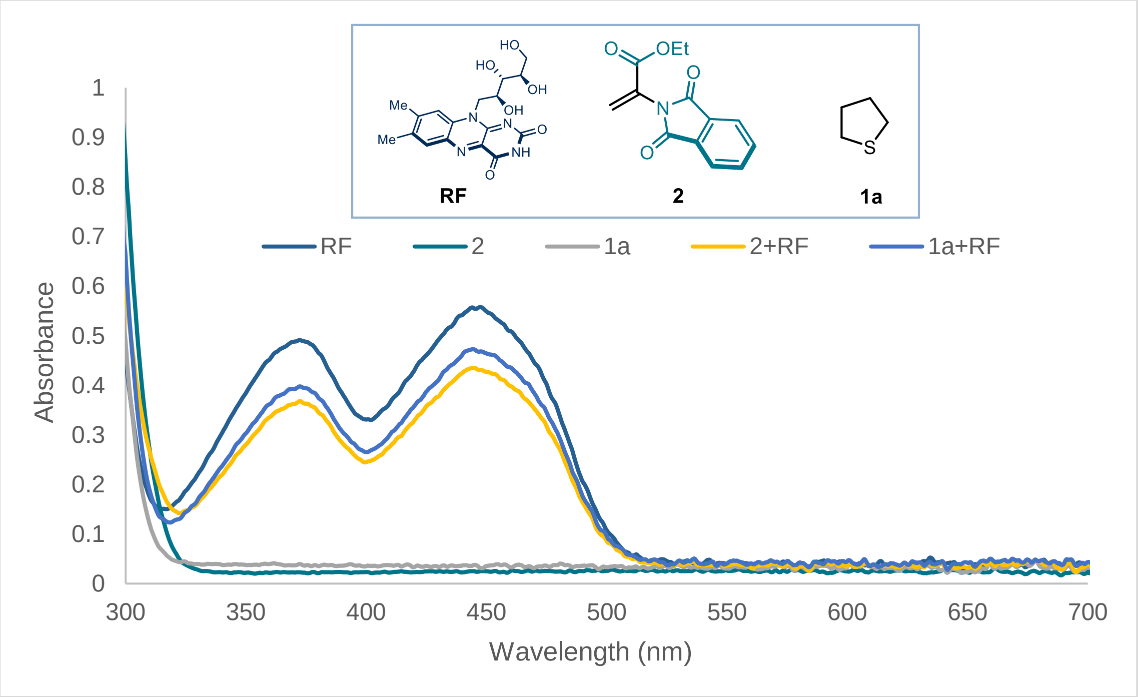

Figure S2: UV/Vis spectra of components and mixtures of components of the reaction.

From the spectra obtained, no shift in the absorbance can be observed in the mixtures of photocatalyst and reactants relative to the photocatalyst alone, suggesting that pre-association does not occur in the ground-state between the flavin and reactants.

Cyclic Voltammetry (CV) Studies

Cyclic voltammetry measurements were conducted on an EmStat4s (PalmSens) potentiostat using a three electrode cell configuration. A glassy carbon working electrode was employed alongside a platinum wire counter electrode and a Ag/AgCl reference electrode. 5 mM solutions of the compounds were freshly prepared in degassed H_2O along with 0.1 M of $NaClO_4$ as supporting electrolyte and were examined at a scan rate of 0.1 V s^{-1}.

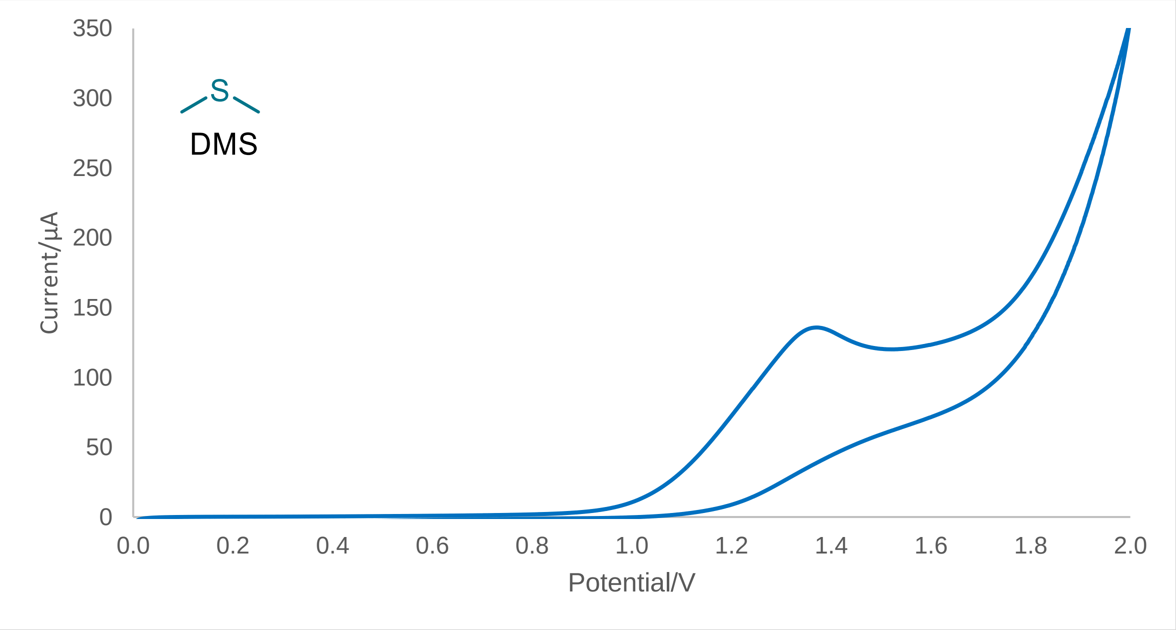

Figure S3: Cyclic voltammogram of dimethyl sulfide (DMS). $E_{p/2}$ (DMS/DMS$^{\bullet+}$) = 1.19 V vs. Ag/AgCl in H_2O.

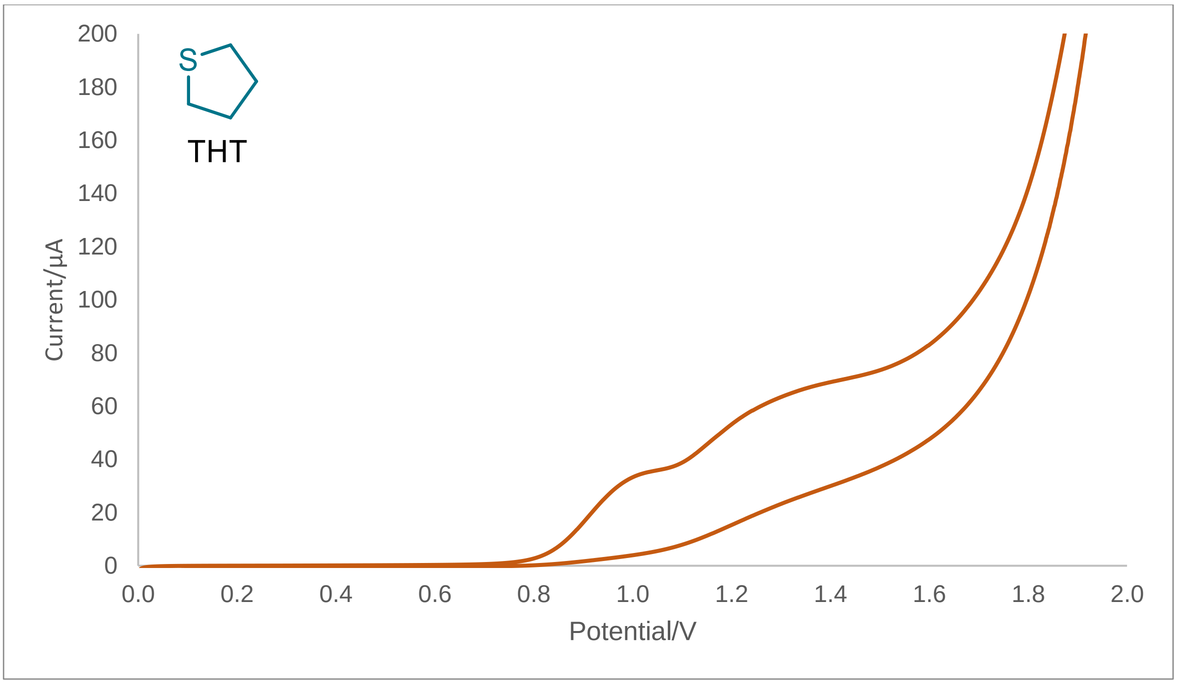

Figure S4: Cyclic voltammogram of tetrahydrothiophene (THT). $E_{p/2}$ (THT/THT$^{\bullet+}$) = 0.92 V vs. Ag/AgCl in H_2O.

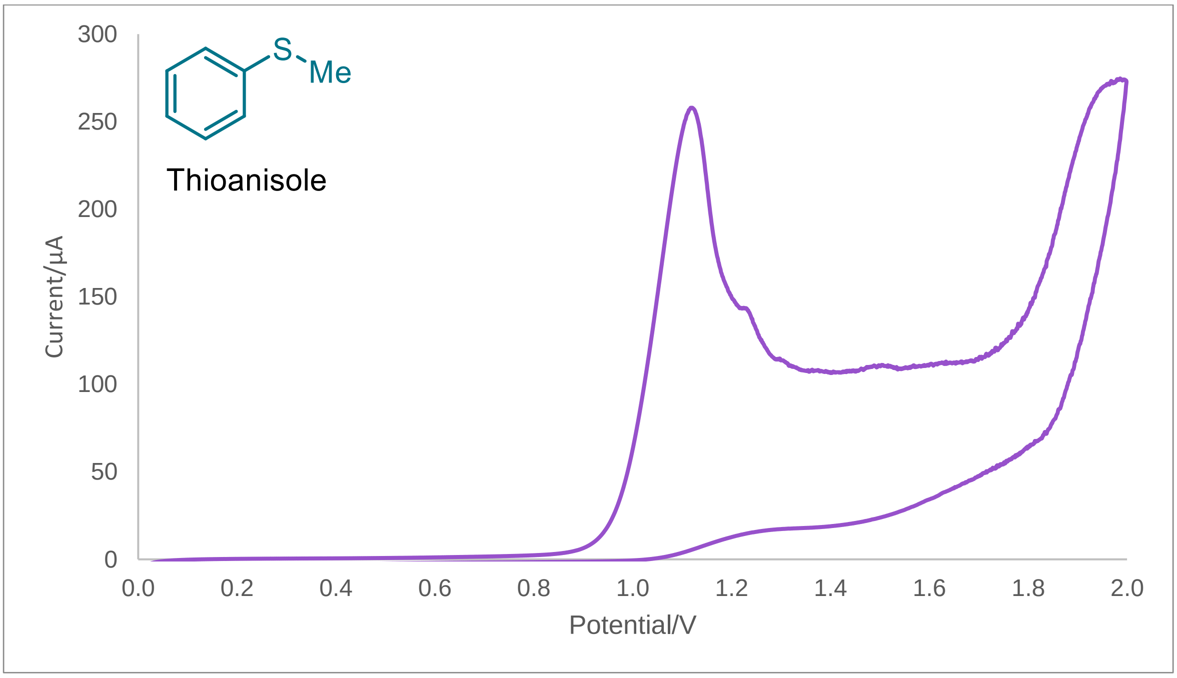

Figure S5: Cyclic voltammogram of thioanisole. $E_{p/2}$ (Thioanisole/Thioanisole$^{\bullet+}$) = 1.04 V vs. Ag/AgCl in H_2O.

Stern-Volmer Quenching Studies

Experiments were performed on a Edinburgh Instruments FLS920 fluorescence spectrometer. Stern-Volmer experiments were conducted to track the quenching of the fluorescence of Riboflavin using both tetrahydrothiophene (THT, **1a**) and ethyl 2-(1,3-dioxoisoindolin-2-yl)acrylate **2**. Degassed stock solutions of each component were prepared prior to each set of experiments. In a typical experiment, 1 mL of a 0.05 mM solution of Riboflavin in 5% DMF:H_2O was added to 1 mL of the substrate in a screw-top 1.0 cm quartz cuvette, giving a final Riboflavin concentration of 0.025 mM. Solutions were excited at a fixed wavelength of 450 nm (incident light slit set to 5 mm) and the emission light was acquired from 460 nm to 750 nm (emission intensity at 530 nm) over three scans. Each measurement was then repeated three times and averaged.

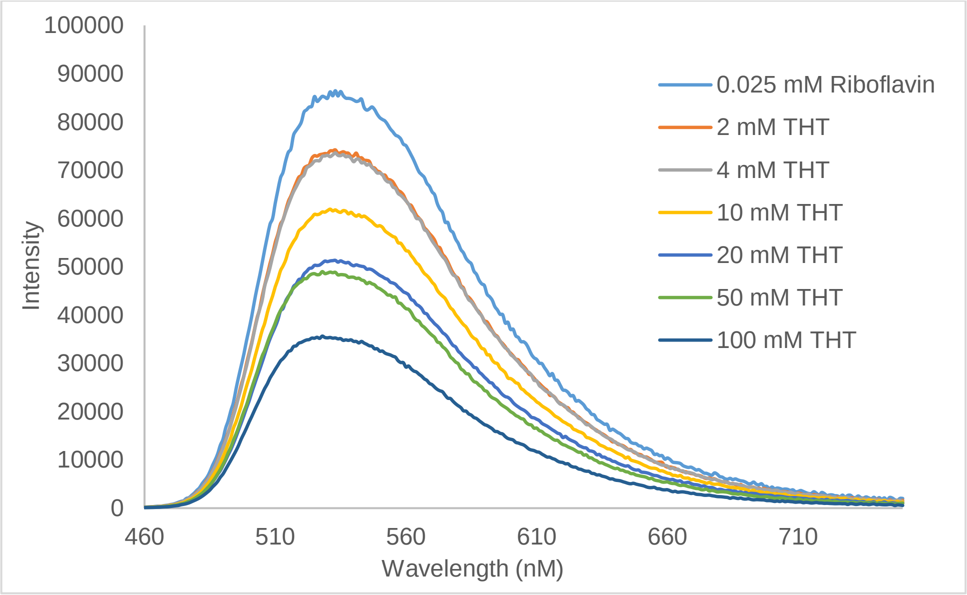

Figure S6: Quenching of Riboflavin emission (0.025 mM in 5% DMF:H_2O) in the presence of increasing amounts of tetrahydrothiophene (THT).

The results shown in Figure S6 suggest that the sulfide quenches the excited state of Riboflavin and its emission.

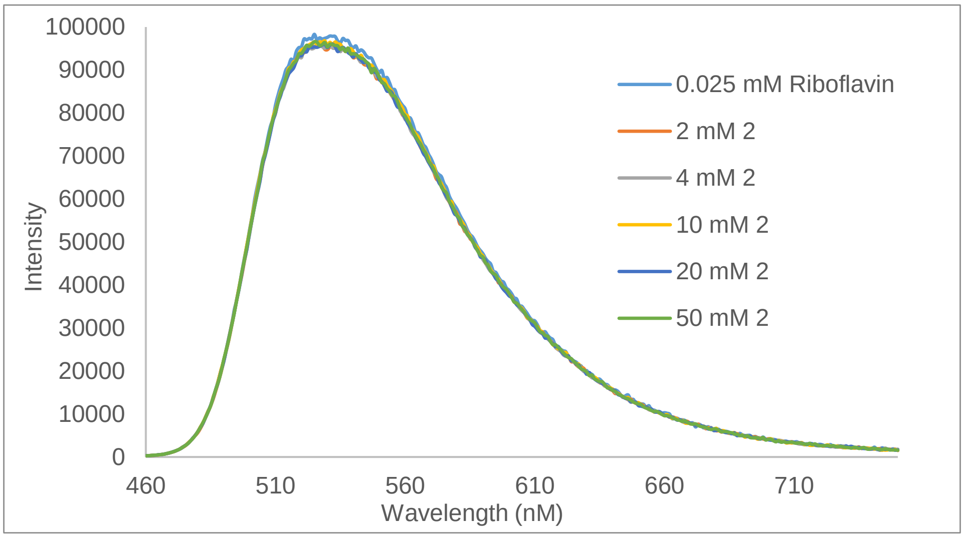

Figure S7: Quenching of Riboflavin emission (0.025 mM in 5% DMF:H_2O) in the presence of increasing amounts of ethyl 2-(1,3-dioxoisoindolin-2-yl)acrylate **2.**

The Stern-Volmer plot (Figure S8) shows a linear correlation between the increasing amounts of THT and the ratio I_0/I. Based on the Stern-Volmer relationship (Eq. 1), it is possible to calculate the Stern-Volmer constant (K_{SV}).[12]

$$I_0/I = 1 + K_{SV}[Q] \quad \textit{[Eq. 1]}$$

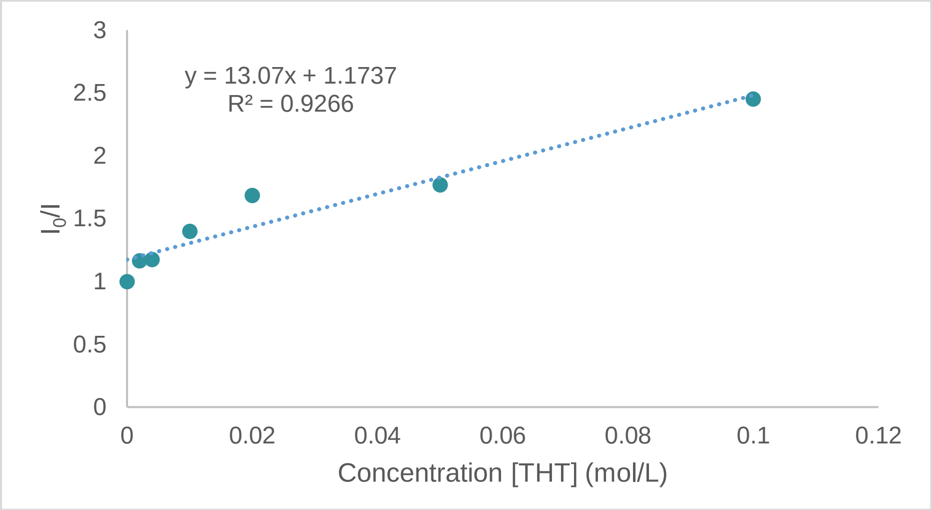

Figure S8: Stern-Volmer quenching plot of THT

A Stern-Volmer quenching constant of 13.1 M^{-1} can be extracted from Figure S8.

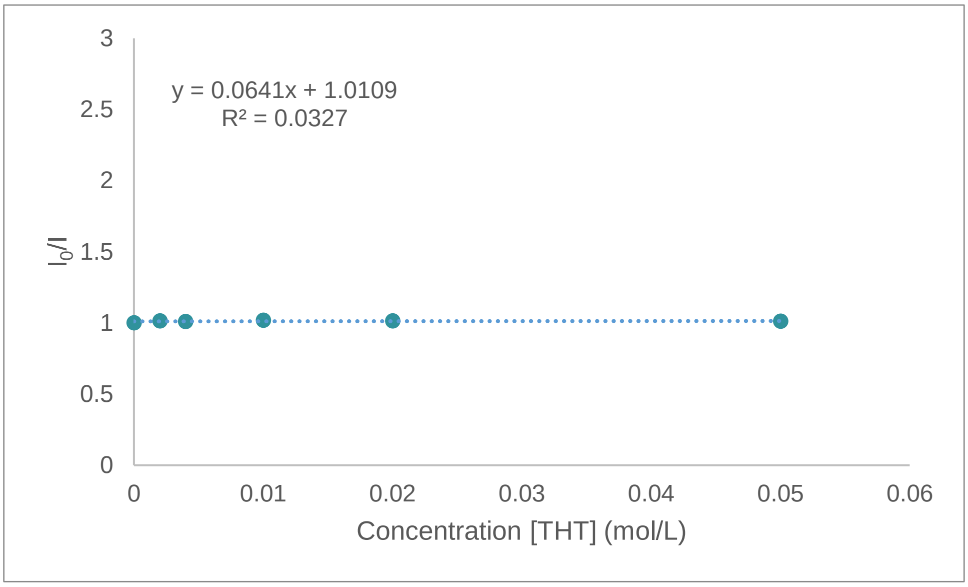

Figure S9: Stern-Volmer quenching plot of ethyl 2-(1,3-dioxoisoindolin-2-yl)acrylate **2**

The results shown in Figure S7 and S9 suggest that alkene **2** does not quench the excited state of Riboflavin and its emission.

¹H- and ¹³C-NMR spectra

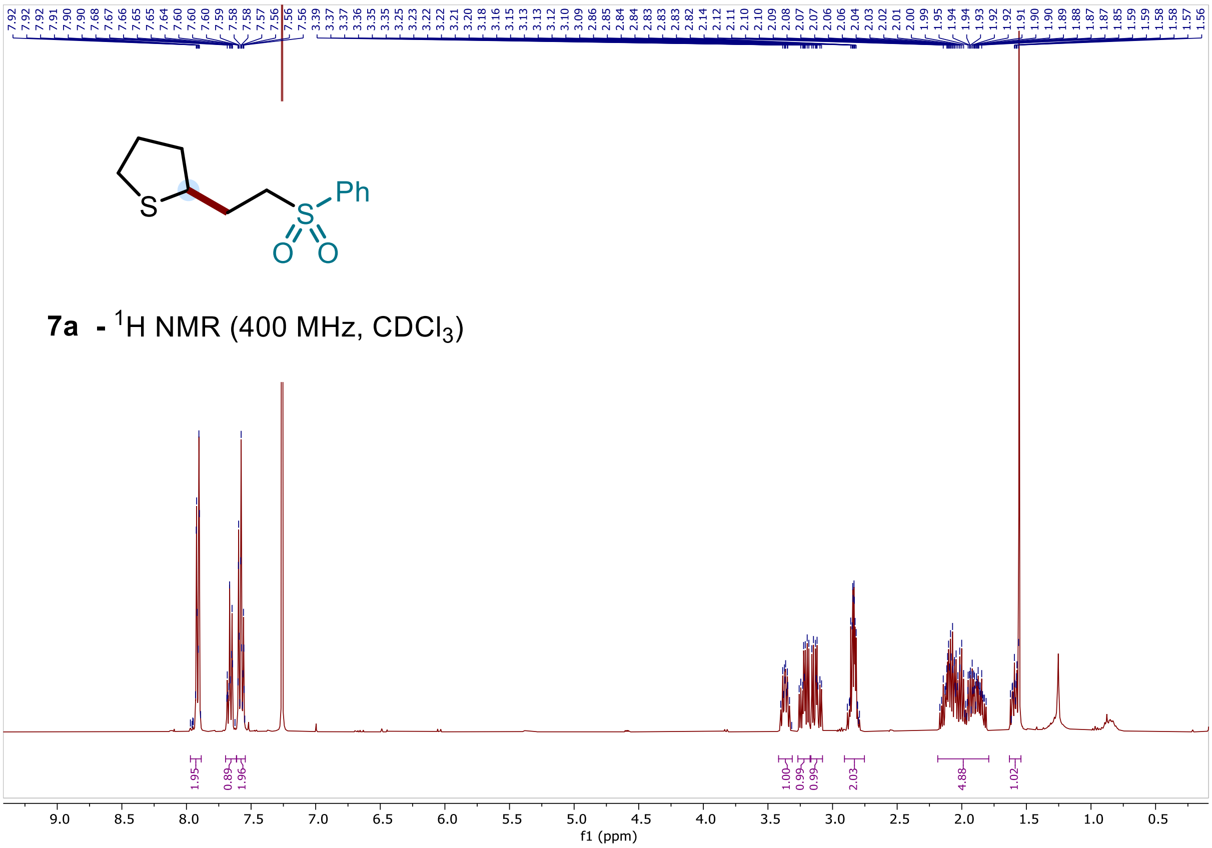

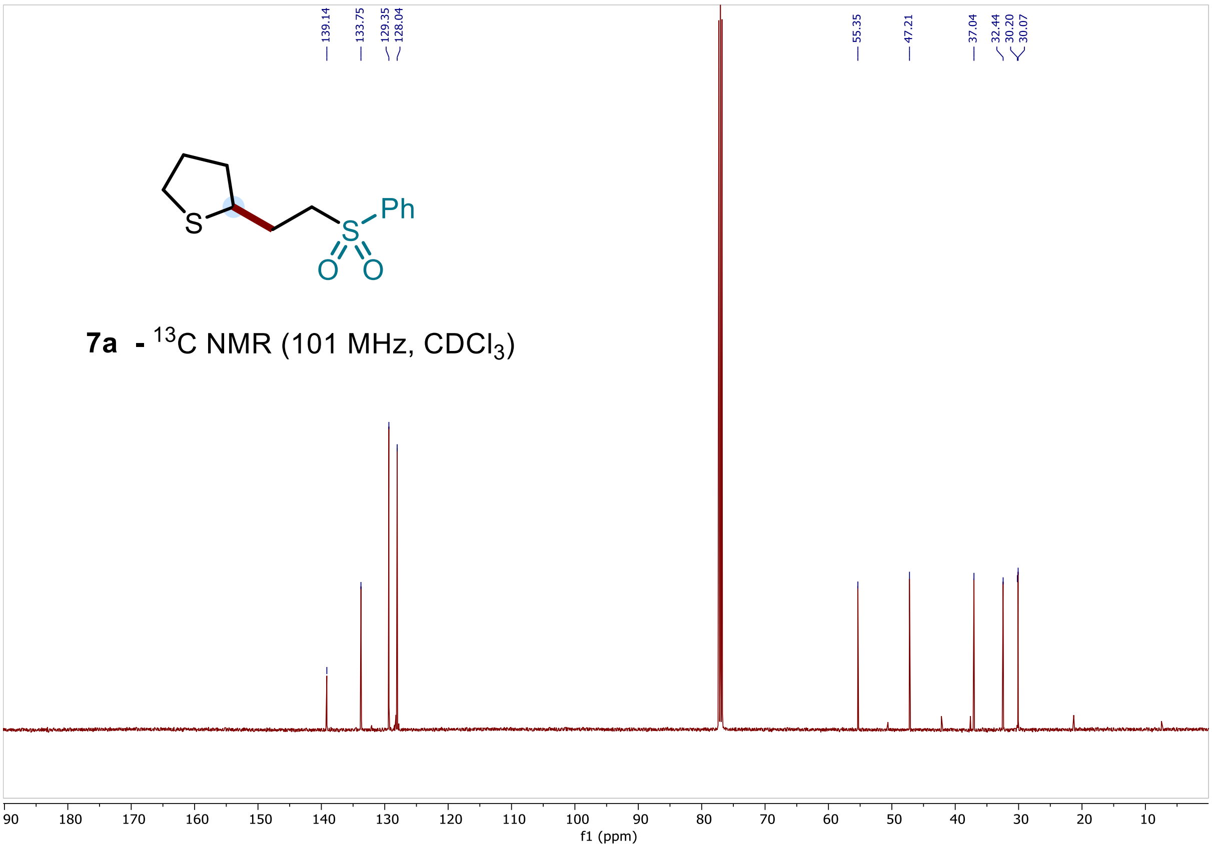

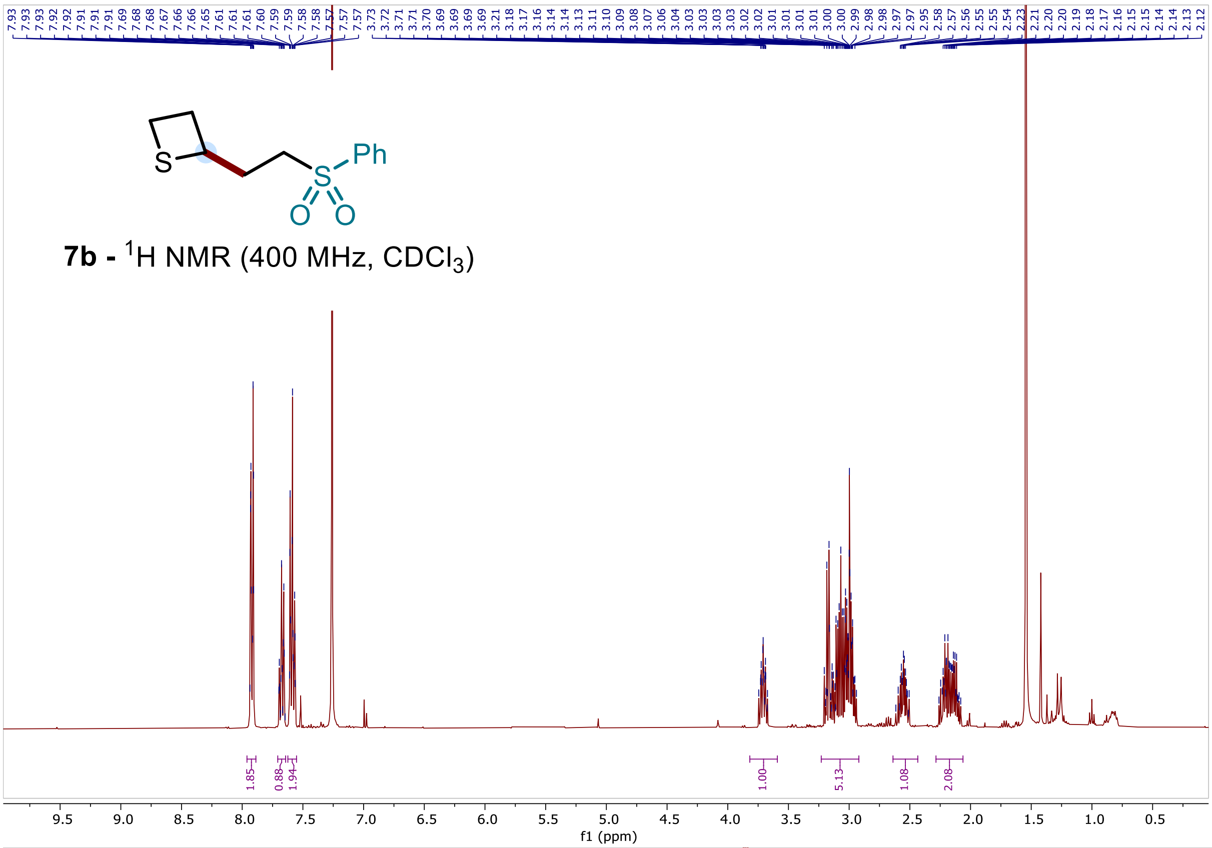
7.93
7.93
7.92
7.92
7.91
7.91
7.69
7.68
7.68
7.67
7.66
7.65
7.61
7.61
7.60
7.59
7.59
7.58
7.58
7.57
7.57
7.57
3.73
3.72
3.71
3.70
3.69
3.69
3.69
3.69
3.21
3.18
3.17
3.16
3.14
3.14
3.13
3.11
3.10
3.09
3.08
3.07
3.06
3.04
3.03
3.03
3.03
3.03
3.02
3.02
3.01
3.01
3.01
3.01
3.00
3.00
2.99
2.98
2.98
2.97
2.97
2.95
2.58
2.57
2.56
2.55
2.55
2.54
2.23
2.21
2.20
2.19
2.18
2.17
2.16
2.15
2.15
2.14
2.14
2.13
2.12
S
Ph
S
O O
7b - ^{1}H NMR (400 MHz, CDCl$_3$)
1.85
0.88
1.94
1.00
5.13
1.08
2.08
9.5 9.0 8.5 8.0 7.5 7.0 6.5 6.0 5.5 5.0 4.5 4.0 3.5 3.0 2.5 2.0 1.5 1.0 0.5
f1 (ppm)

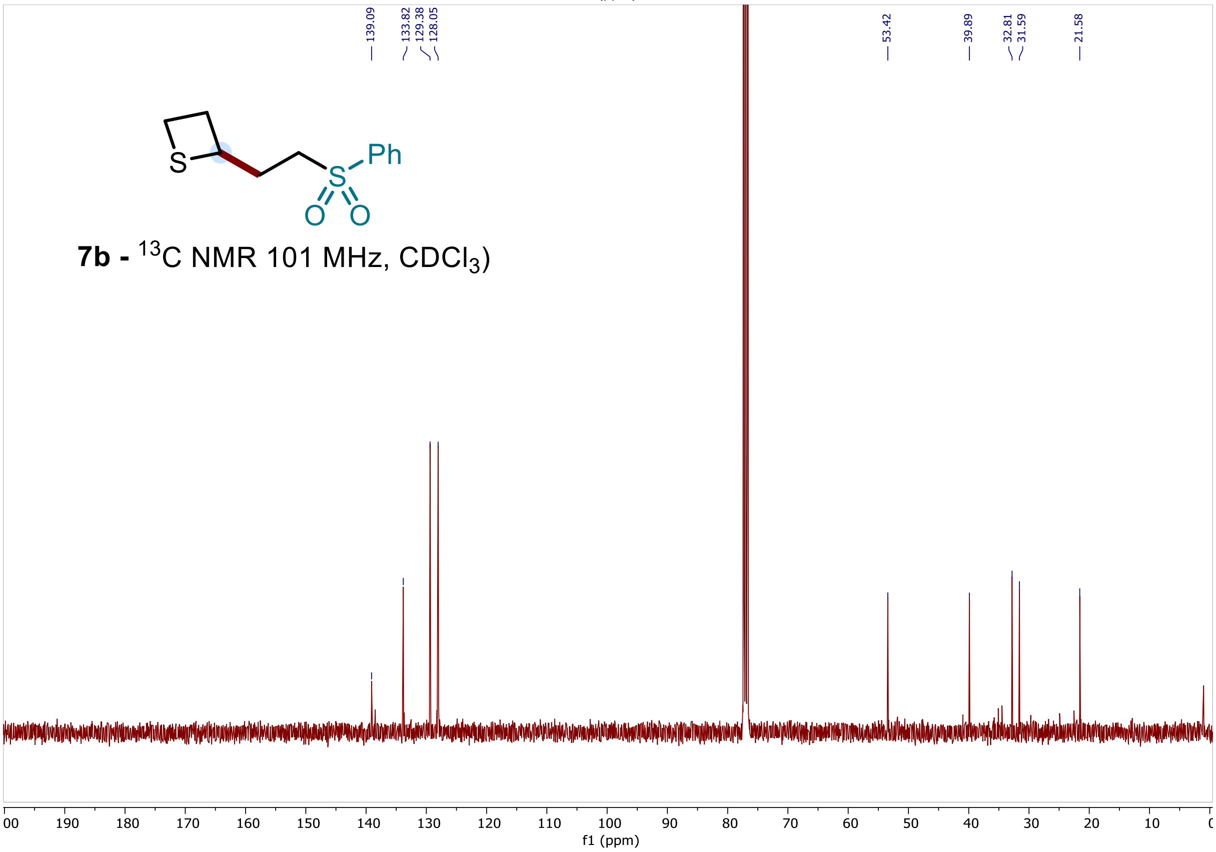
139.09
133.82
129.38
128.05
53.42
39.89
32.81
31.59
21.58
S
Ph
S
O O
7b - ^{13}C NMR 101 MHz, CDCl$_3$)
00 190 180 170 160 150 140 130 120 110 100 90 80 70 60 50 40 30 20 10
f1 (ppm)

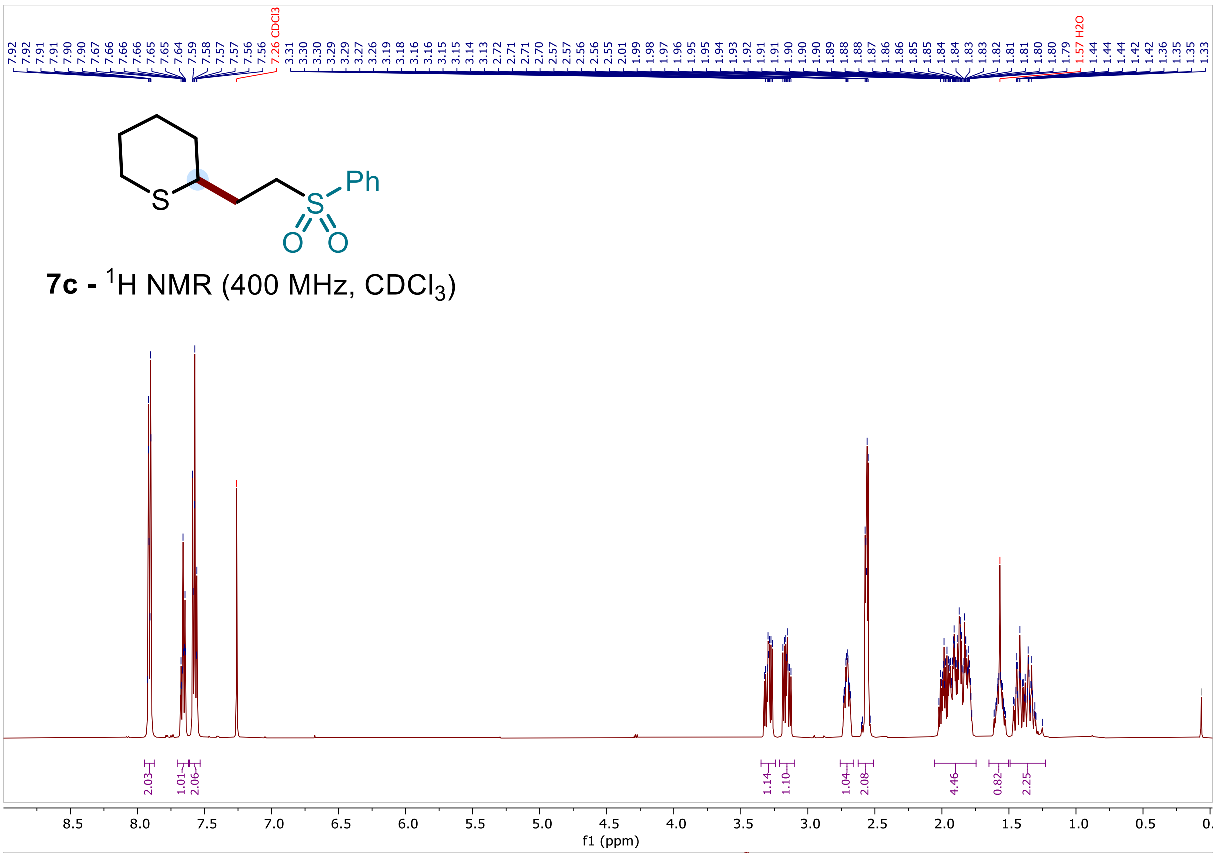
7c - 1H NMR (400 MHz, CDCl3)
f1 (ppm)
Ph
S
O
7.26 CDCl3
1.57 H2O

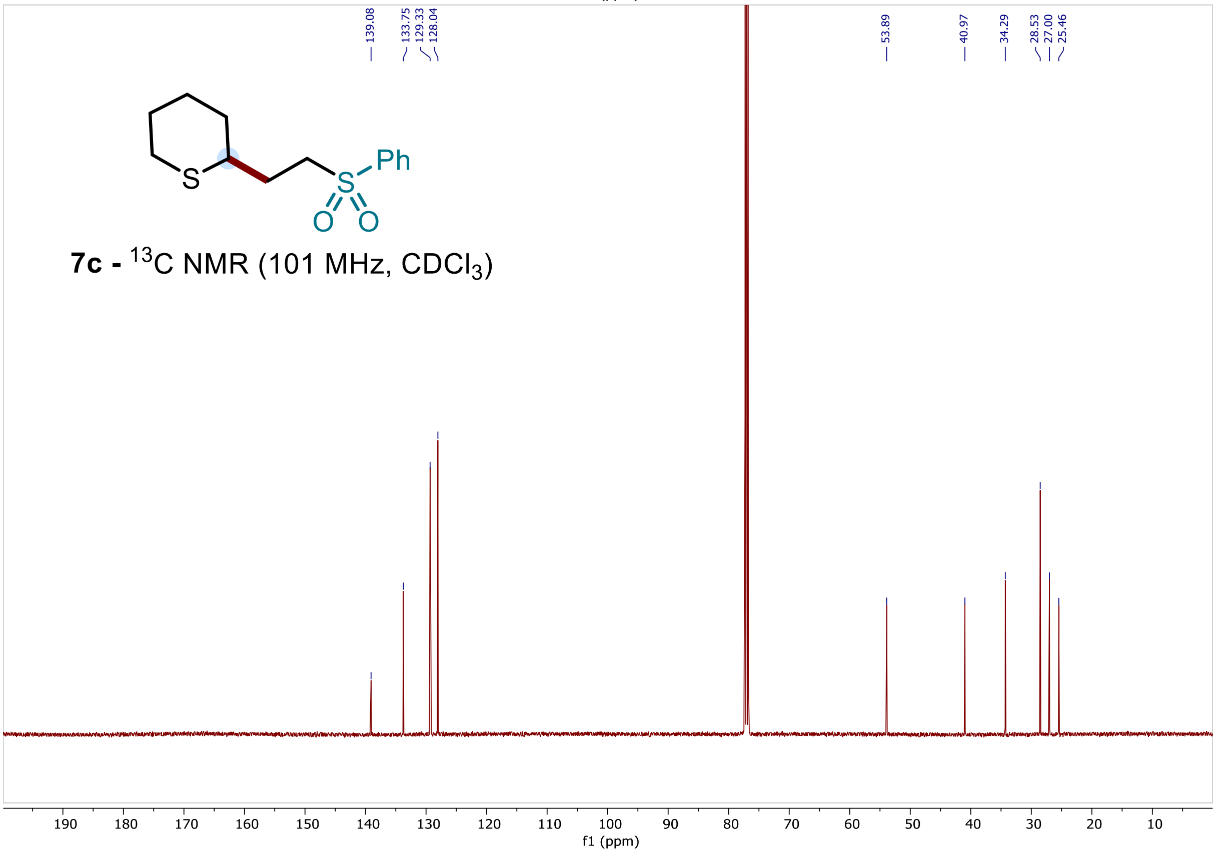
7c - 13C NMR (101 MHz, CDCl3)
f1 (ppm)
139.08
133.75
129.33
128.04
53.89
40.97
34.29
28.53
27.00
25.46

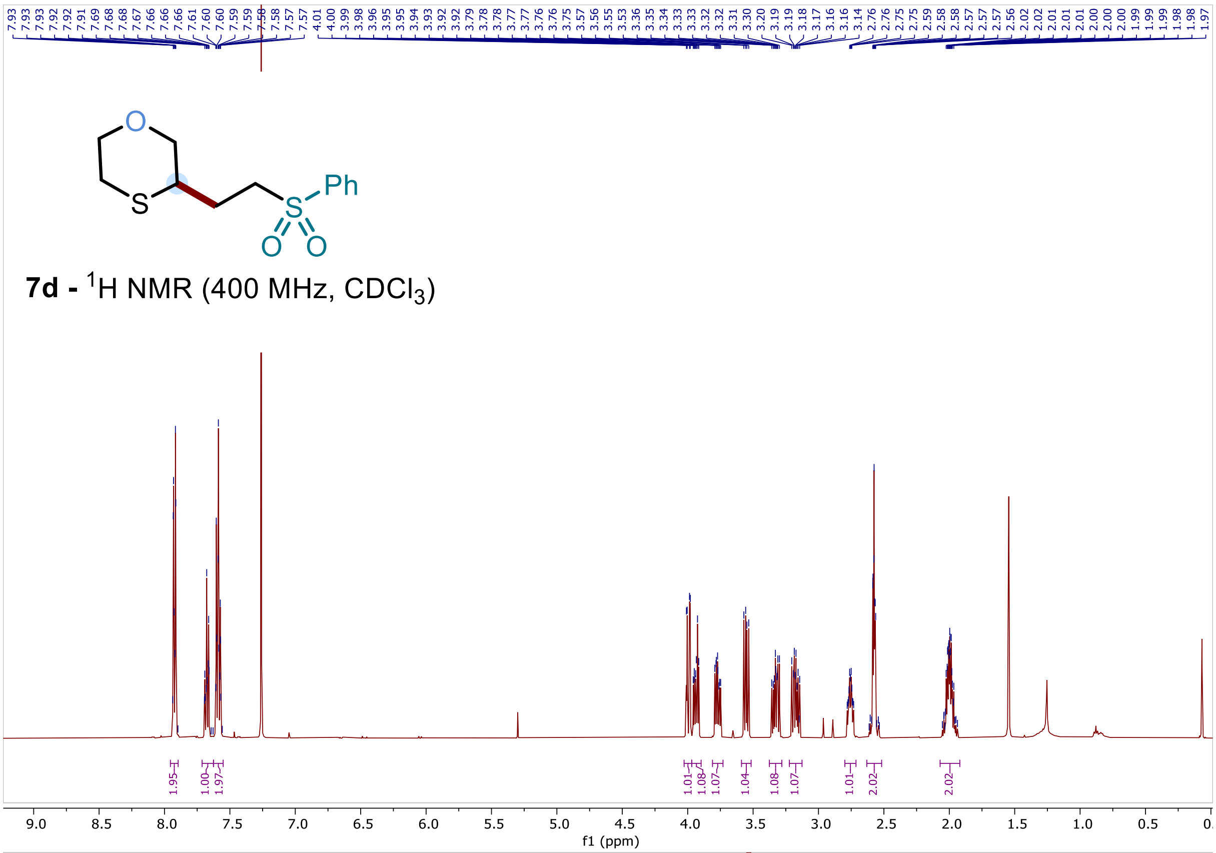
O
S
Ph
S
O O
7d - 1H NMR (400 MHz, CDCl3)
f1 (ppm)

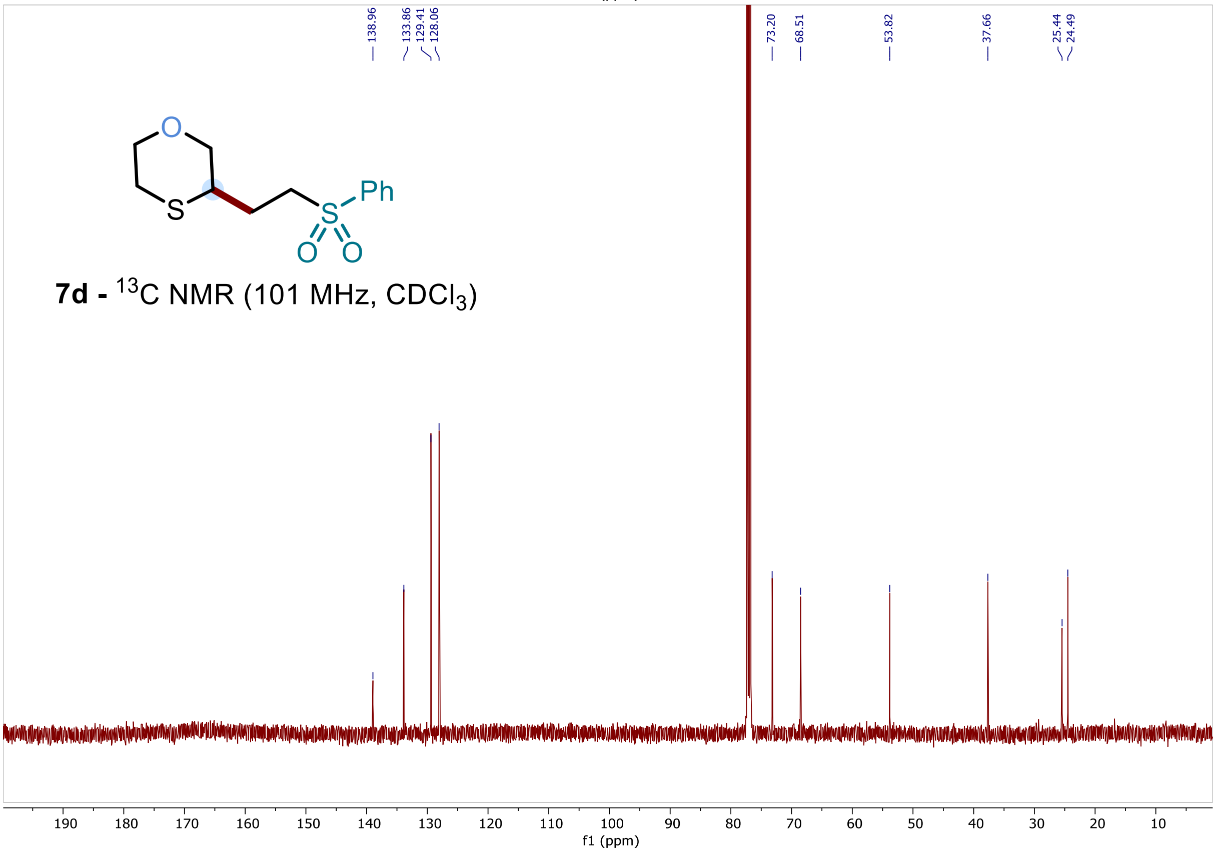
138.96
133.86
129.41
128.06
73.20
68.51
53.82
37.66
25.44
24.49
O
S
Ph
S
O O
7d - 13C NMR (101 MHz, CDCl3)
f1 (ppm)

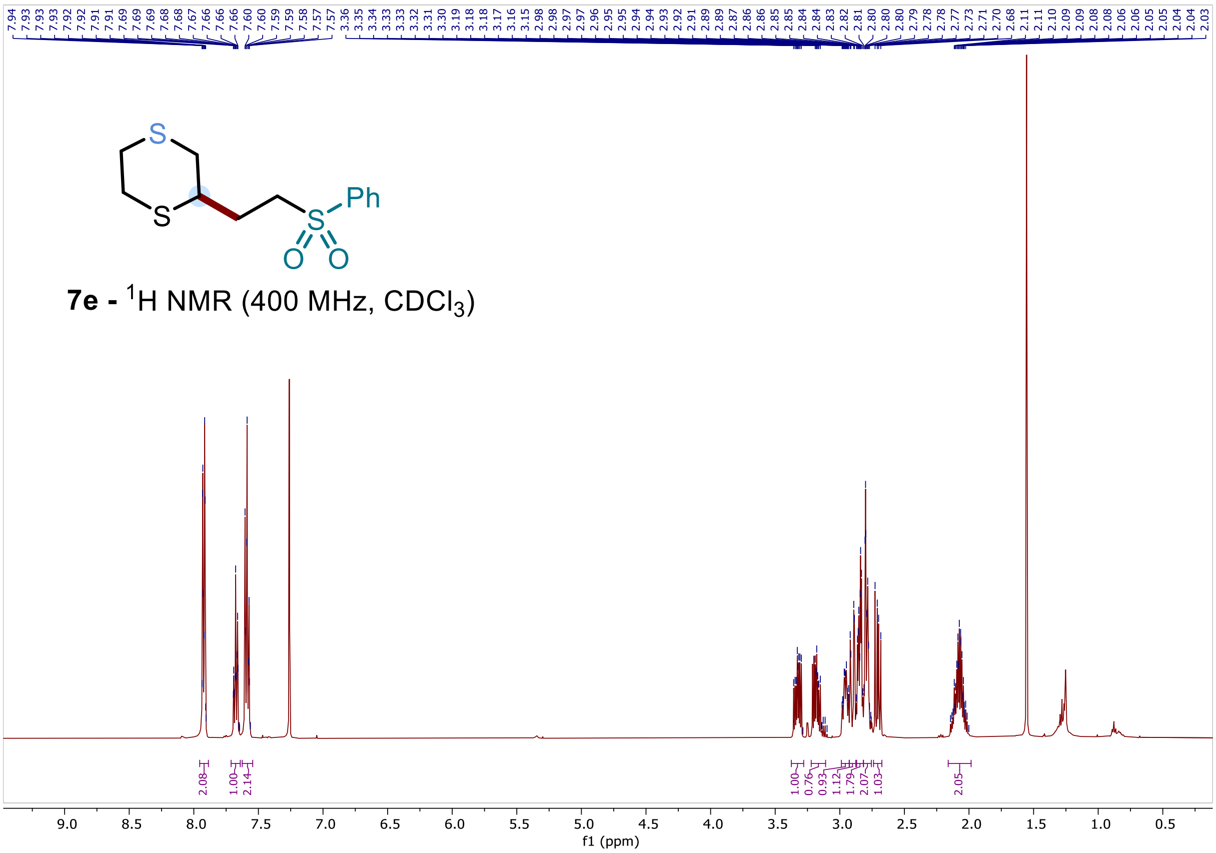
7.94 7.93 7.93 7.92 7.92 7.91 7.91 7.69 7.69 7.69 7.68 7.68 7.67 7.66 7.66 7.60 7.60 7.59 7.59 7.58 7.57 7.57 3.36 3.35 3.34 3.33 3.33 3.32 3.31 3.30 3.19 3.18 3.18 3.17 3.16 3.15 2.98 2.98 2.97 2.97 2.96 2.95 2.95 2.94 2.94 2.93 2.92 2.91 2.89 2.89 2.87 2.86 2.86 2.85 2.85 2.84 2.84 2.83 2.82 2.81 2.80 2.80 2.79 2.78 2.77 2.73 2.71 2.70 2.68 2.11 2.11 2.10 2.09 2.09 2.08 2.08 2.06 2.06 2.05 2.05 2.04 2.04 2.03
S
S
Ph
S
O O
7e - ^{1}H NMR (400 MHz, $CDCl_3$)
2.08
1.00
2.14
1.00
0.76
0.93
1.12
1.79
2.07
1.03
2.05
f1 (ppm)

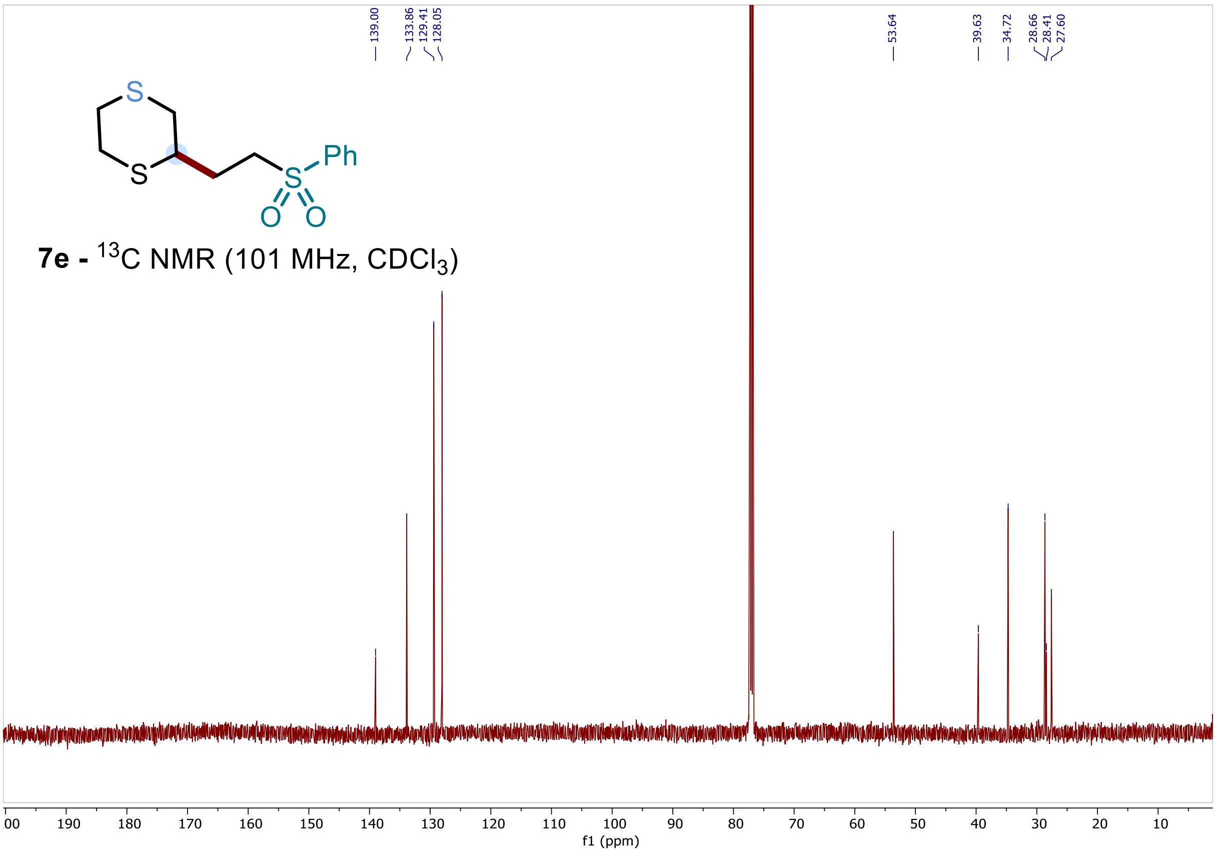
139.00
133.86
129.41
128.05
53.64
39.63
34.72
28.66
28.41
27.60
S
S
Ph
S
O O
7e - ^{13}C NMR (101 MHz, $CDCl_3$)
f1 (ppm)

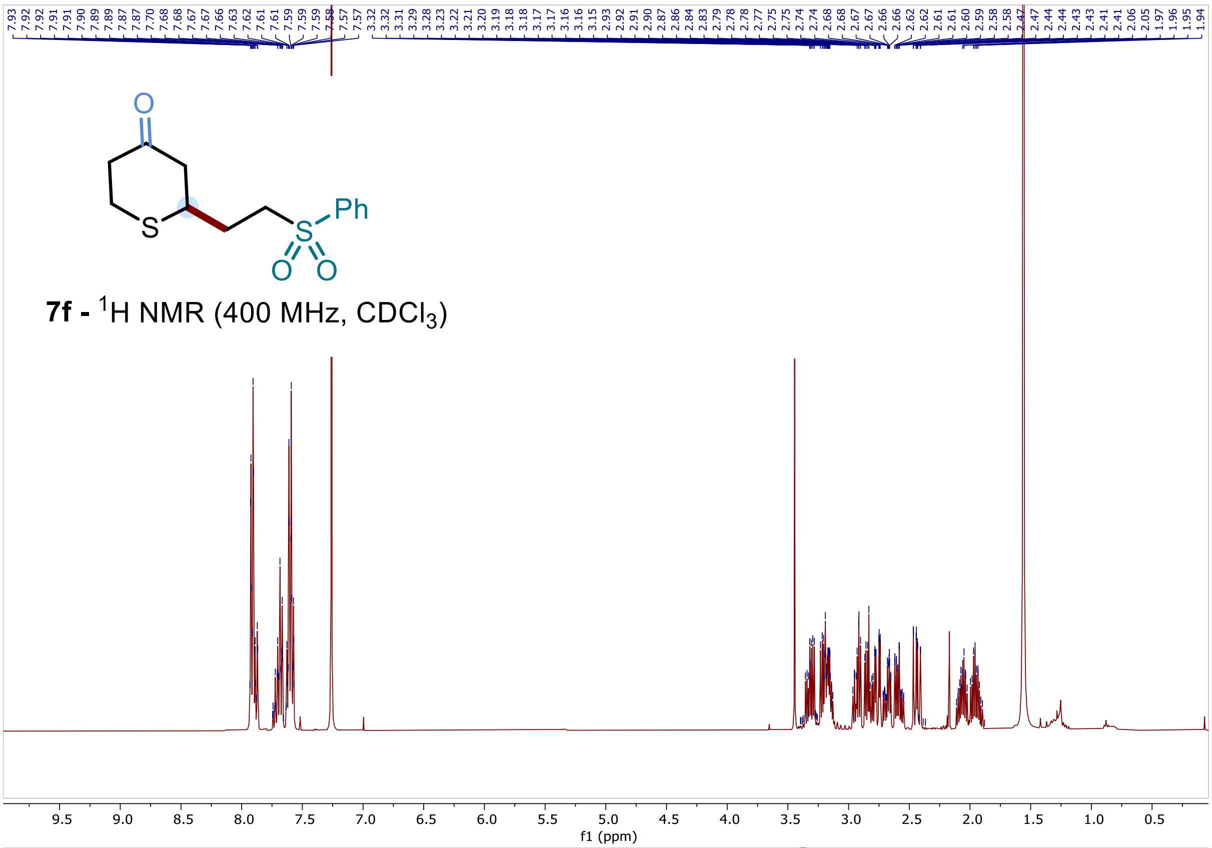
7f - ^{1}H NMR (400 MHz, CDCl$_3$)
Ph
S
O O
7.93 7.92 7.92 7.91 7.90 7.89 7.89 7.87 7.87 7.70 7.68 7.68 7.67 7.67 7.66 7.63 7.62 7.61 7.61 7.59 7.59 7.59 7.58 7.57 7.57 3.32 3.32 3.31 3.29 3.28 3.23 3.22 3.21 3.20 3.19 3.18 3.18 3.17 3.17 3.16 3.16 3.15 2.93 2.92 2.91 2.90 2.87 2.86 2.84 2.83 2.79 2.78 2.78 2.77 2.75 2.75 2.74 2.74 2.68 2.68 2.67 2.67 2.66 2.66 2.62 2.62 2.61 2.61 2.60 2.59 2.58 2.58 2.47 2.47 2.44 2.44 2.43 2.43 2.41 2.41 2.06 2.05 1.97 1.96 1.95 1.94
f1 (ppm)

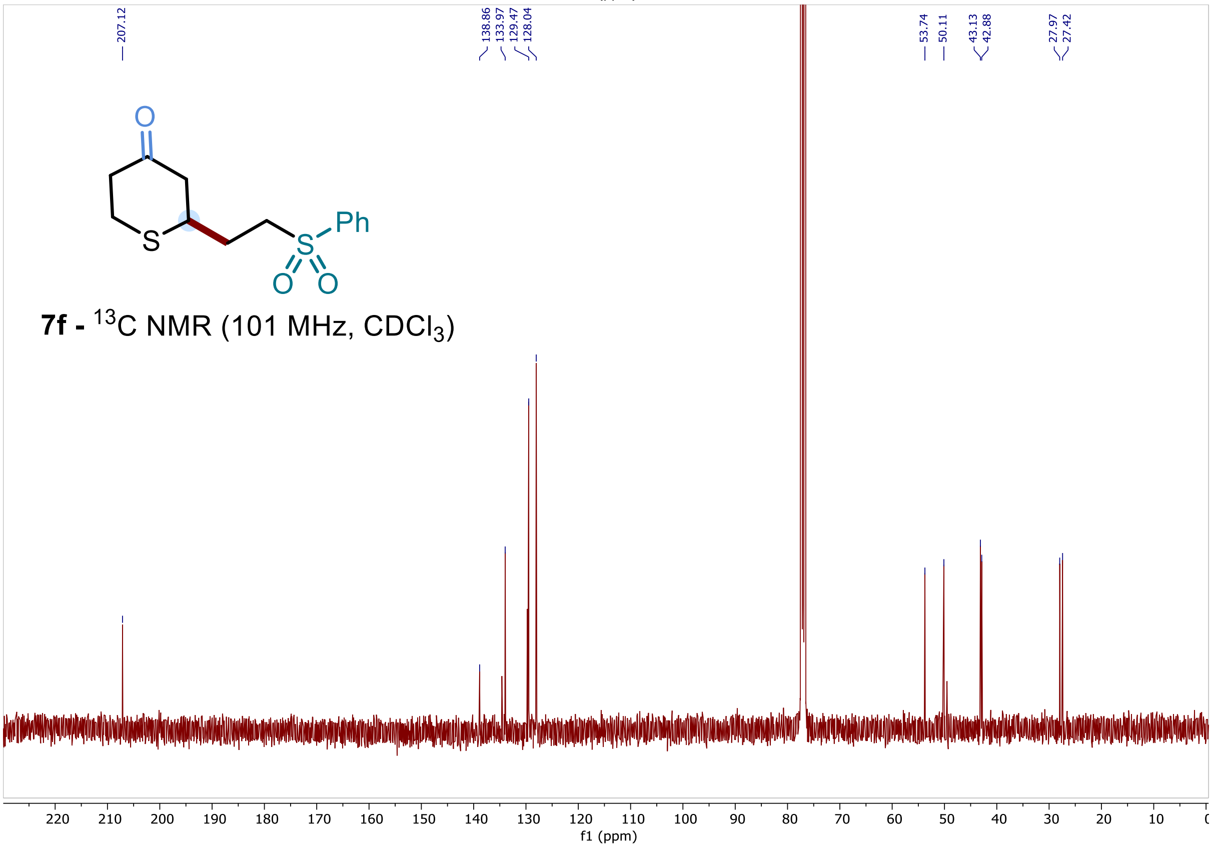
7f - ^{13}C NMR (101 MHz, CDCl$_3$)
Ph
S
O O
207.12
138.86 133.97 129.47 128.04
53.74 50.11 43.13 42.88 27.97 27.42
f1 (ppm)

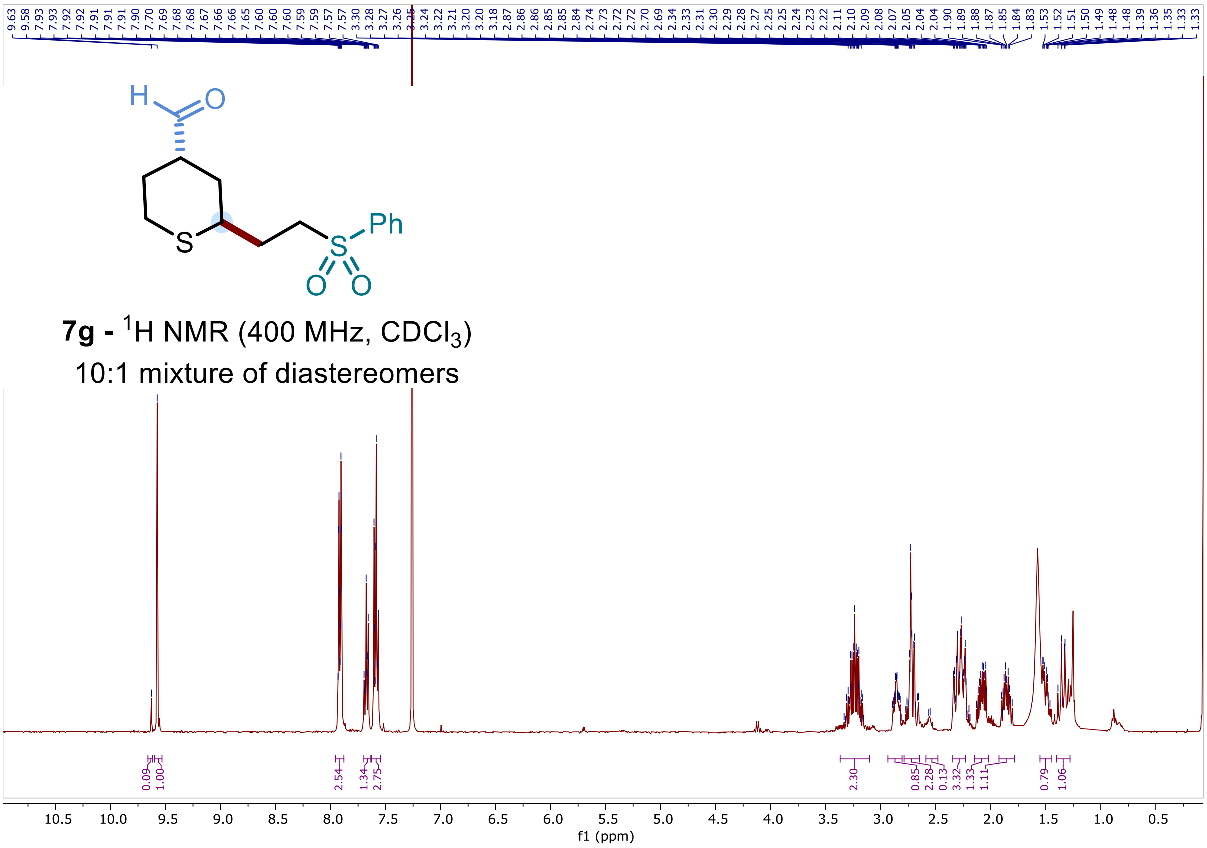
9.63 9.58 7.93 7.93 7.92 7.92 7.91 7.91 7.91 7.90 7.70 7.69 7.68 7.68 7.67 7.66 7.65 7.60 7.60 7.60 7.59 7.59 7.57 7.57 3.30 3.28 3.27 3.26 3.25 3.24 3.22 3.21 3.20 3.18 2.87 2.86 2.86 2.85 2.85 2.84 2.74 2.73 2.72 2.72 2.70 2.69 2.34 2.33 2.31 2.30 2.29 2.28 2.27 2.25 2.25 2.24 2.23 2.22 2.11 2.10 2.09 2.08 2.07 2.05 2.04 2.04 1.90 1.89 1.88 1.87 1.85 1.84 1.83 1.53 1.52 1.51 1.50 1.49 1.48 1.48 1.39 1.36 1.35 1.33 1.33
H
O
S
S
Ph
O O
7g - 1H NMR (400 MHz, CDCl3)
10:1 mixture of diastereomers
0.09 1.00 2.54 1.34 2.75 2.30 0.85 2.28 0.13 3.32 1.33 1.11 0.79 1.06
10.5 10.0 9.5 9.0 8.5 8.0 7.5 7.0 6.5 6.0 5.5 5.0 4.5 4.0 3.5 3.0 2.5 2.0 1.5 1.0 0.5
f1 (ppm)

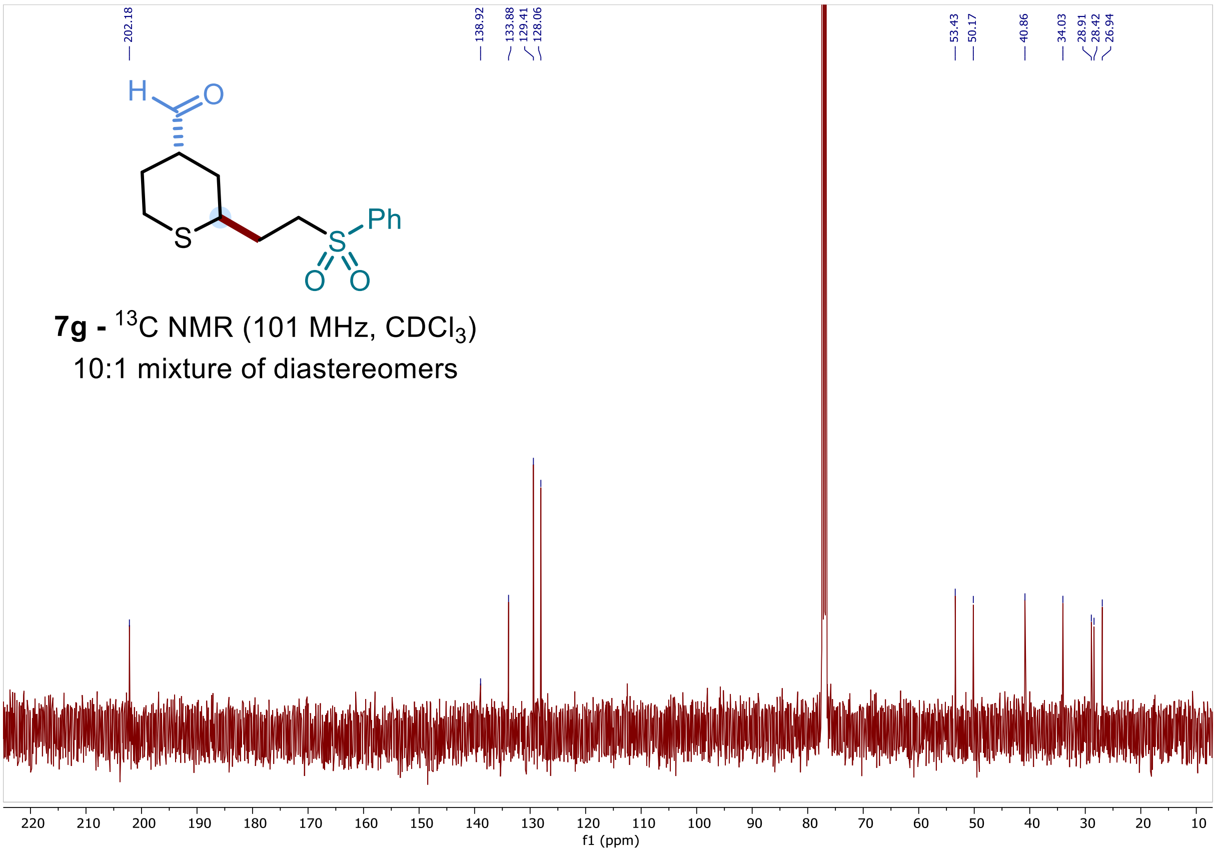
202.18
138.92 133.88 129.41 128.06
53.43 50.17 40.86 34.03 28.91 28.42 26.94
H
O
S
S
Ph
O O
7g - 13C NMR (101 MHz, CDCl3)
10:1 mixture of diastereomers
220 210 200 190 180 170 160 150 140 130 120 110 100 90 80 70 60 50 40 30 20 10
f1 (ppm)

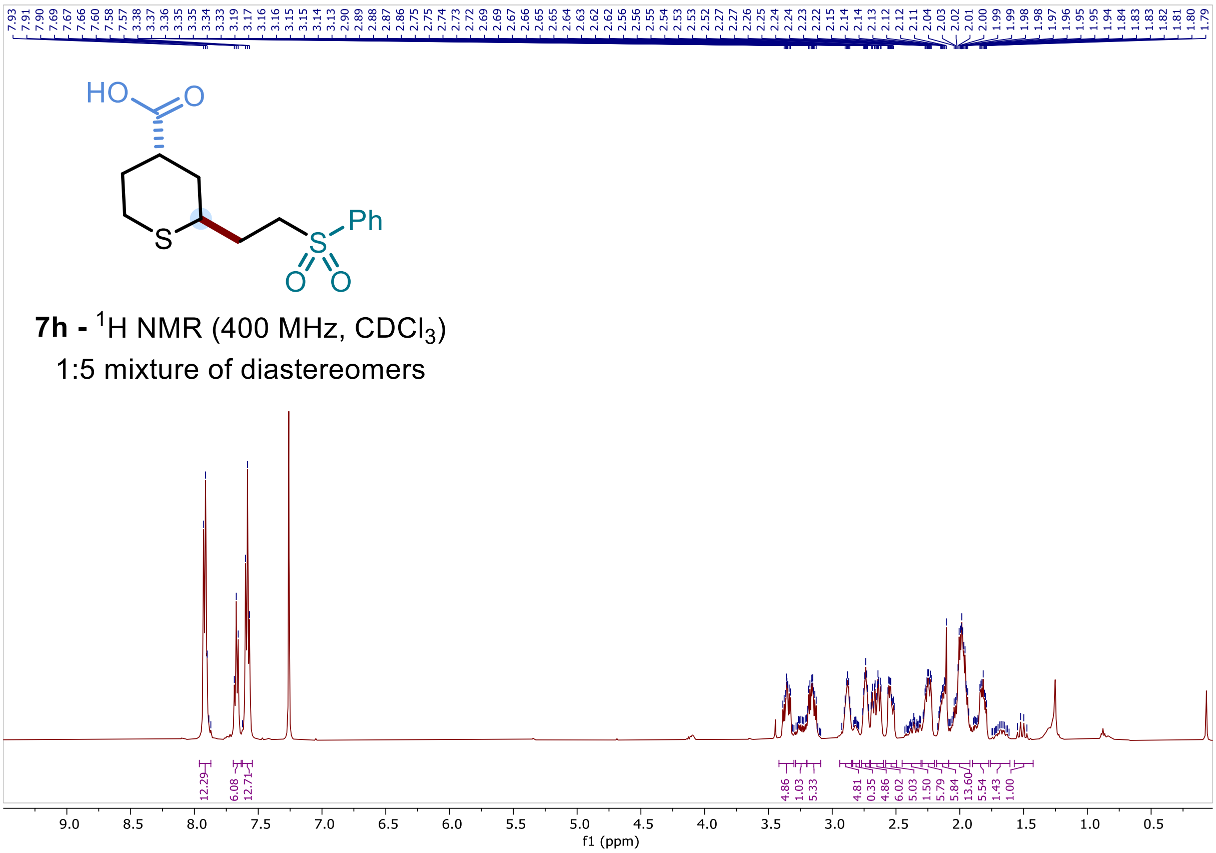

HO
O
S
Ph
S
O
O
7h - ^{1}H NMR (400 MHz, $CDCl_3$)
1:5 mixture of diastereomers
f1 (ppm)

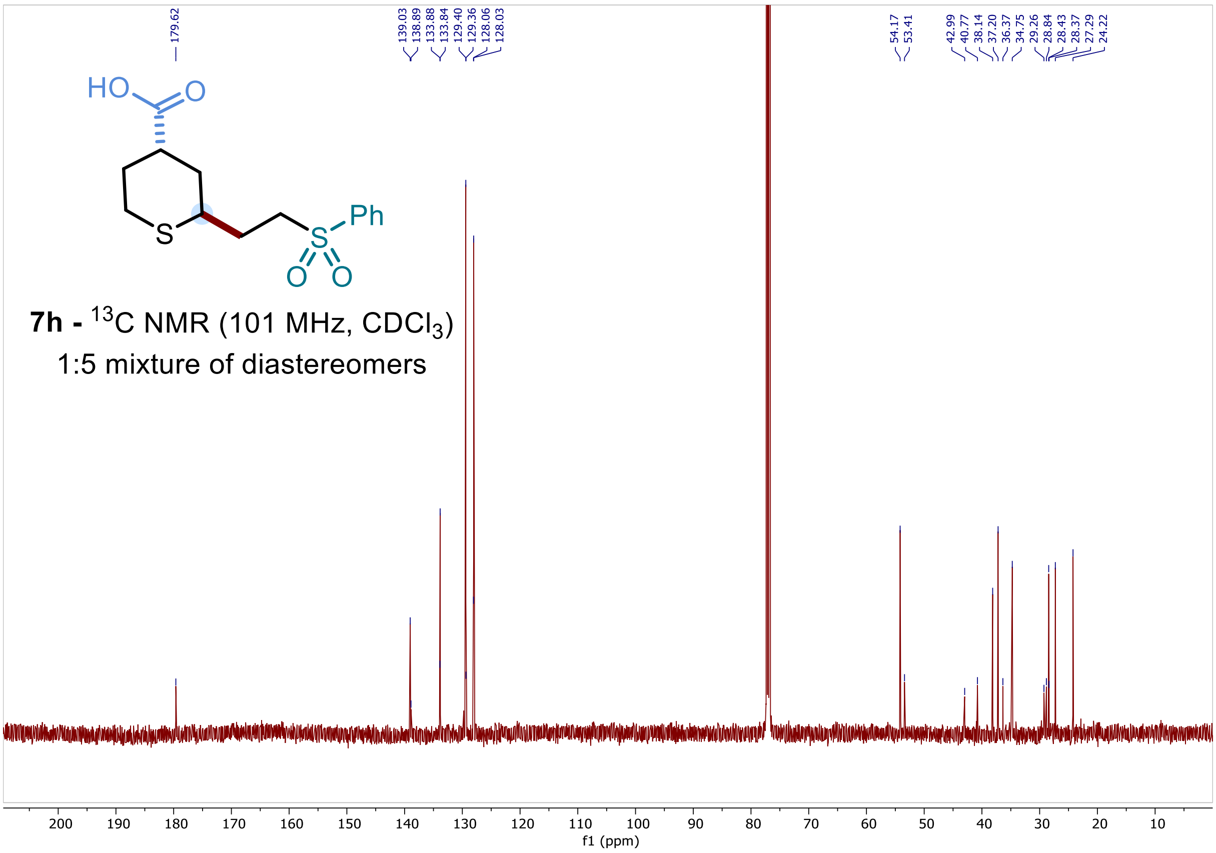

HO
O
S
Ph
S
O
O
7h - ^{13}C NMR (101 MHz, $CDCl_3$)
1:5 mixture of diastereomers
179.62
139.03
138.89
133.88
133.84
129.40
129.36
128.06
128.03
54.17
53.41
42.99
40.77
38.14
37.20
36.37
34.75
29.26
28.84
28.43
28.37
27.29
24.22
f1 (ppm)

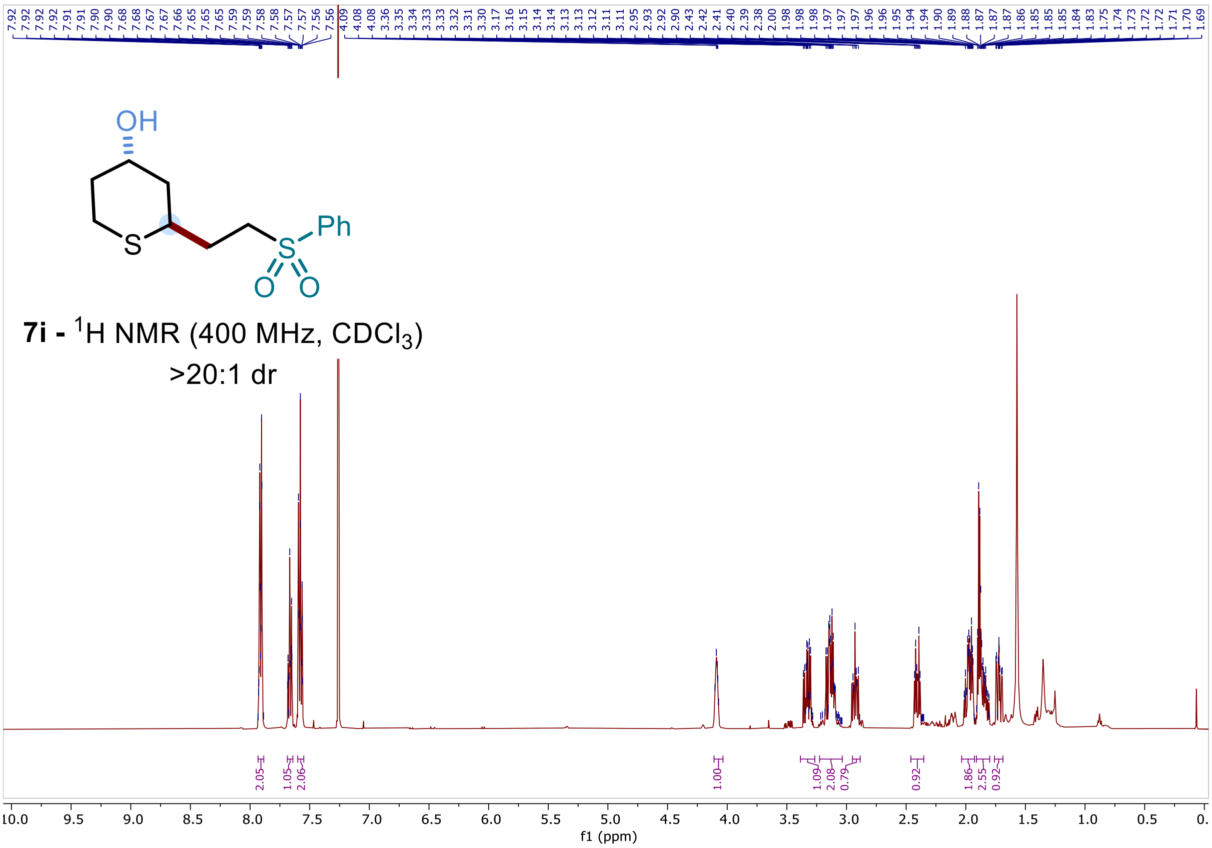
OH
S
S
Ph
O
O
7i - 1H NMR (400 MHz, CDCl3)
>20:1 dr
f1 (ppm)

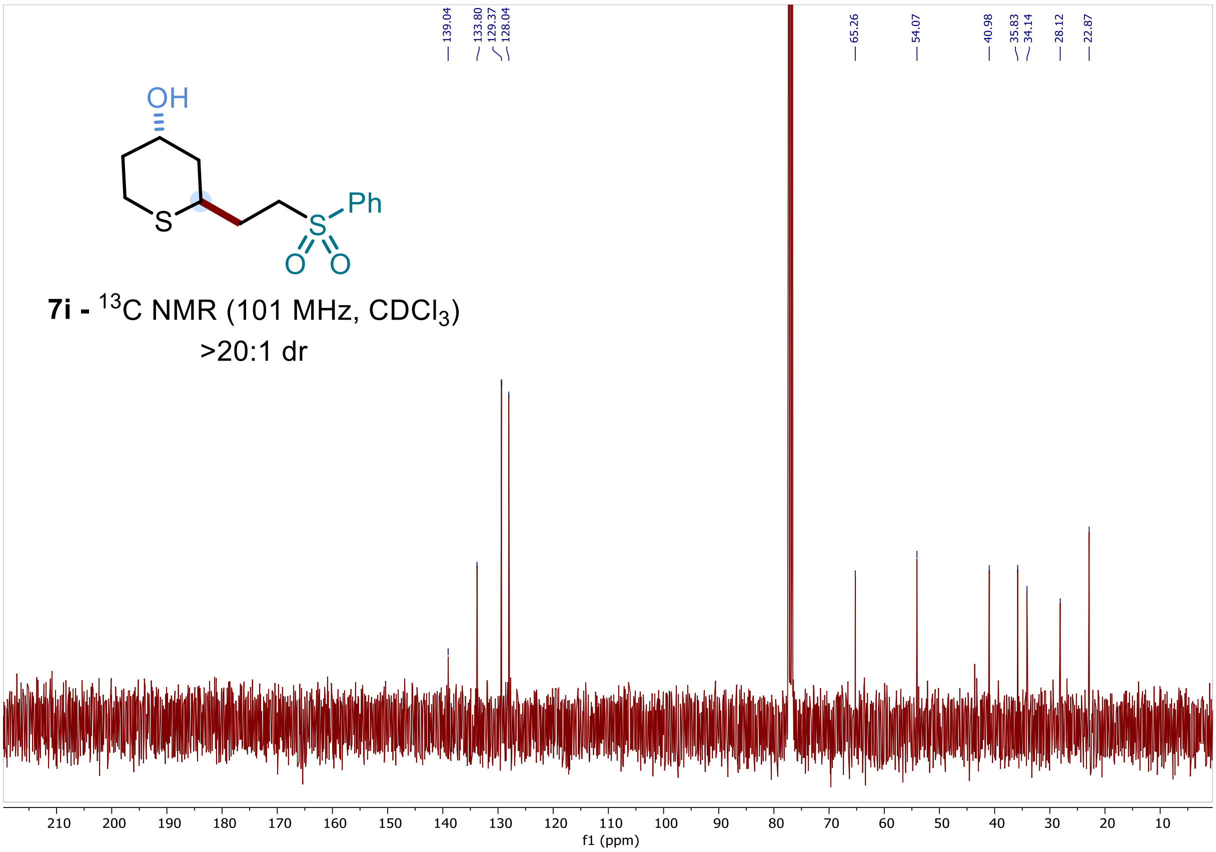
139.04
133.80
129.37
128.04
65.26
54.07
40.98
35.83
34.14
28.12
22.87
OH
S
S
Ph
O
O
7i - 13C NMR (101 MHz, CDCl3)
>20:1 dr
f1 (ppm)

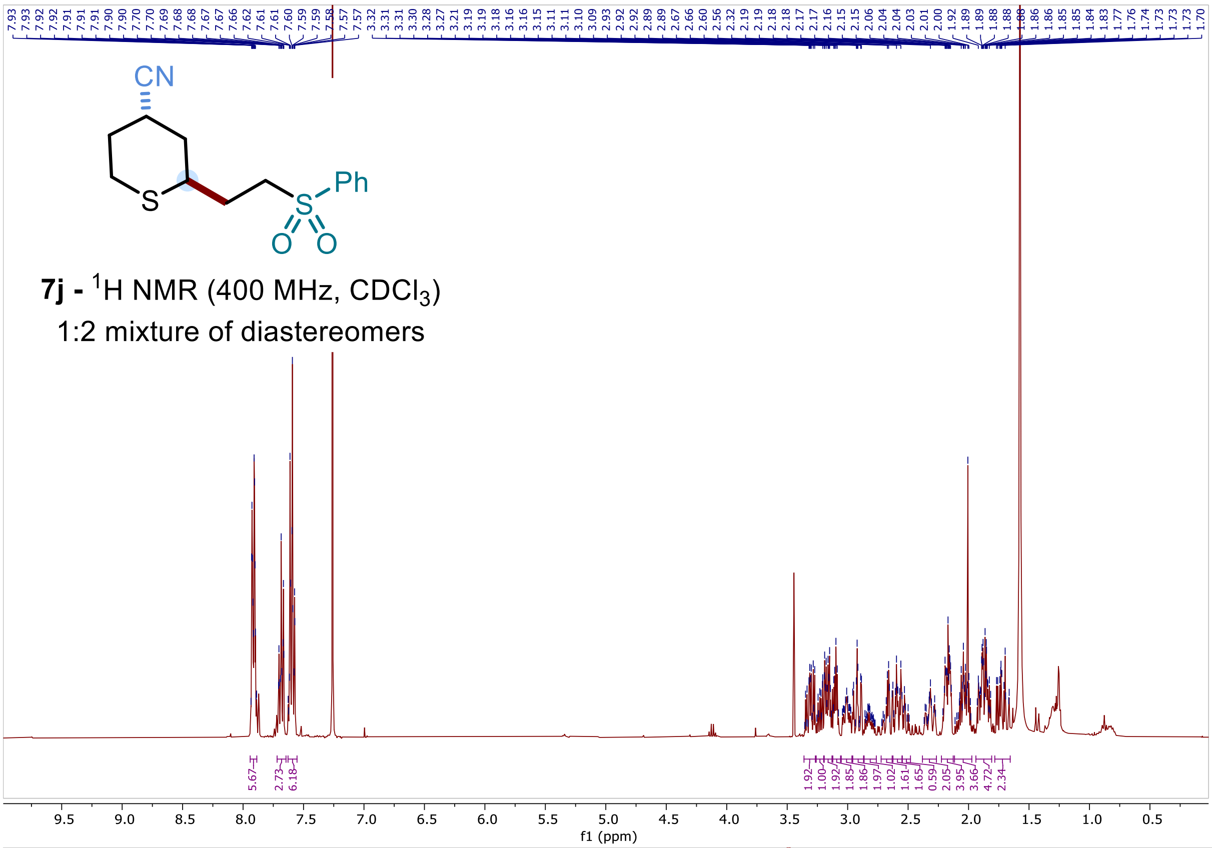
CN
Ph
S
O
O
7j - 1H NMR (400 MHz, CDCl3)
1:2 mixture of diastereomers
f1 (ppm)

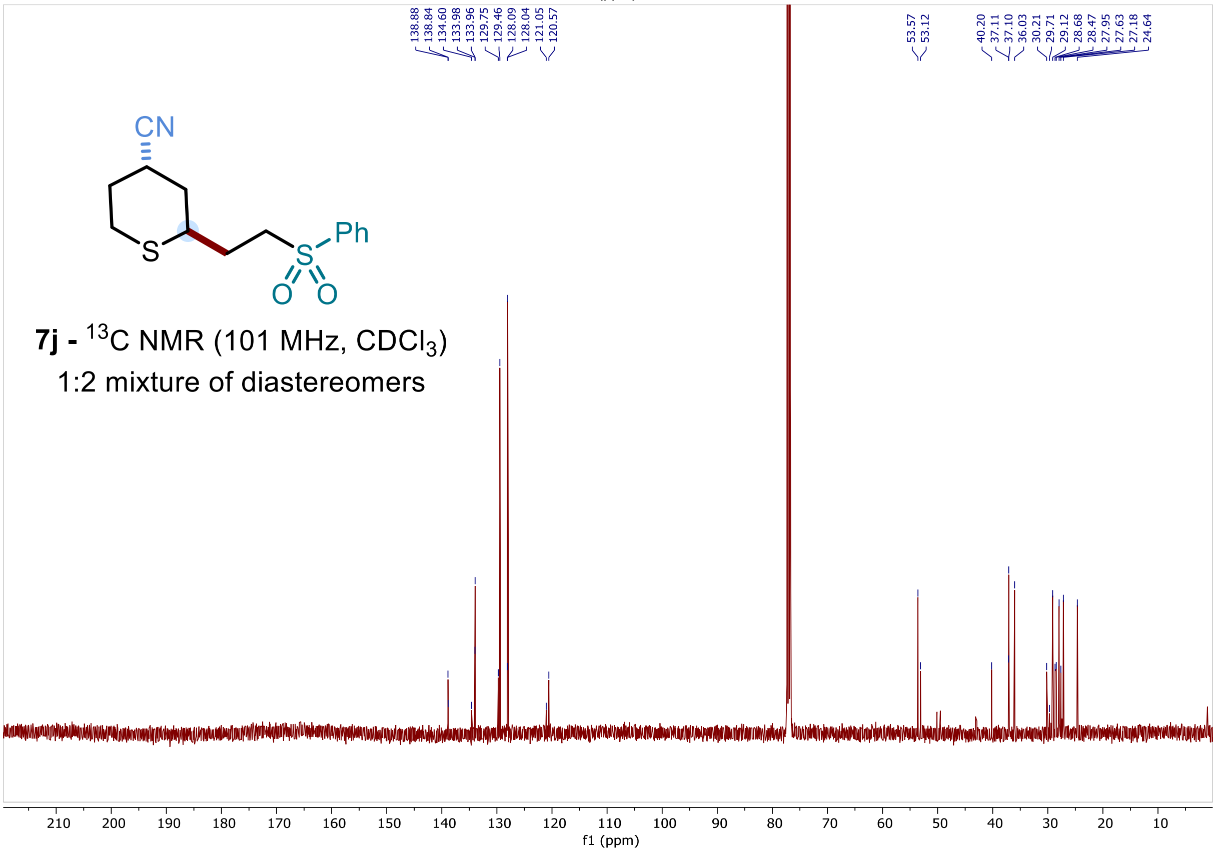
CN
Ph
S
O
O
7j - 13C NMR (101 MHz, CDCl3)
1:2 mixture of diastereomers
f1 (ppm)

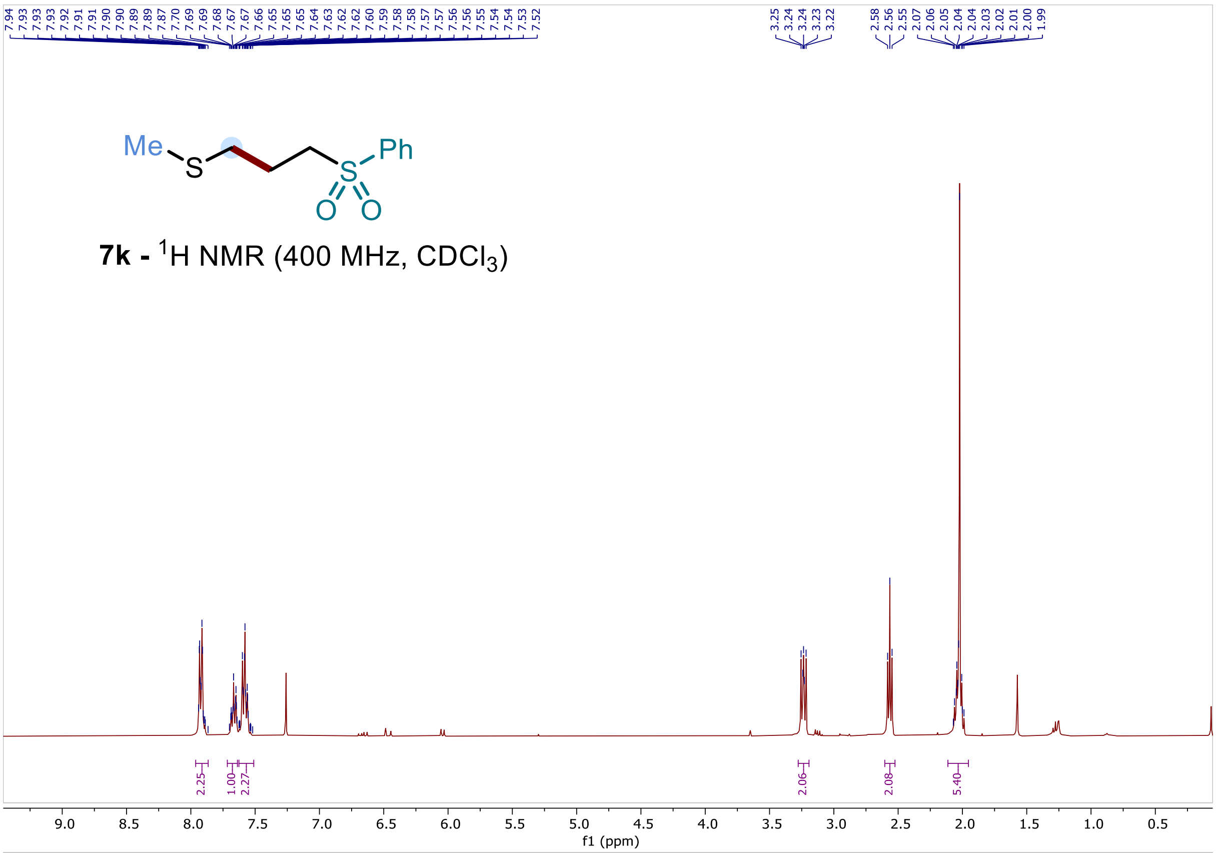
Me
S
S
Ph
O O
7k - ^{1}H NMR (400 MHz, CDCl$_3$)
7.94 7.93 7.93 7.93 7.92 7.91 7.91 7.90 7.90 7.89 7.89 7.87 7.70 7.69 7.69 7.68 7.67 7.67 7.66 7.65 7.65 7.65 7.64 7.63 7.62 7.62 7.60 7.59 7.58 7.58 7.57 7.57 7.56 7.56 7.55 7.54 7.54 7.53 7.52
3.25 3.24 3.24 3.23 3.22
2.58 2.56 2.55 2.07 2.06 2.05 2.04 2.04 2.03 2.02 2.01 2.00 1.99
2.25 1.00 2.27 2.06 2.08 5.40
9.0 8.5 8.0 7.5 7.0 6.5 6.0 5.5 5.0 4.5 4.0 3.5 3.0 2.5 2.0 1.5 1.0 0.5
f1 (ppm)

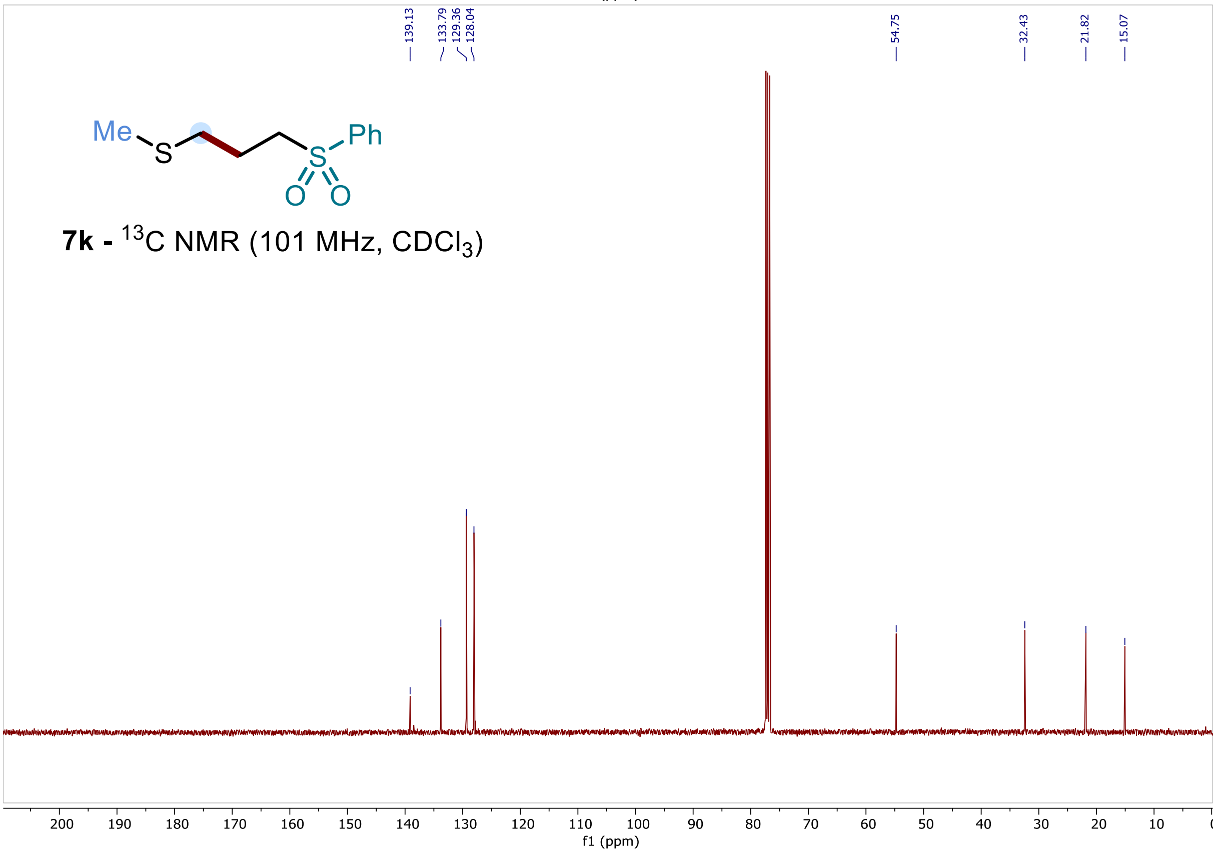
Me
S
S
Ph
O O
7k - ^{13}C NMR (101 MHz, CDCl$_3$)
139.13
133.79 129.36 128.04
54.75
32.43
21.82
15.07
200 190 180 170 160 150 140 130 120 110 100 90 80 70 60 50 40 30 20 10
f1 (ppm)

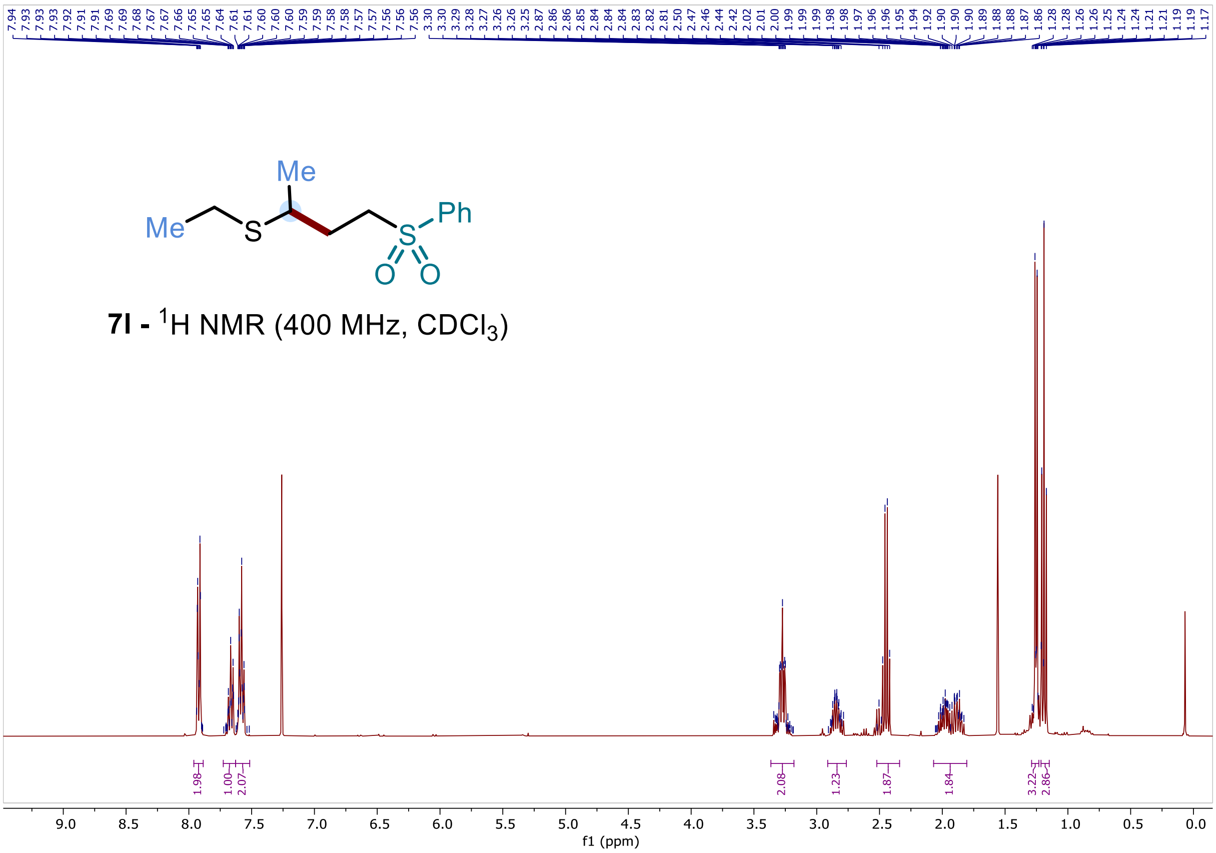
Me
Me
S
S
Ph
O O
7l - ^{1}H NMR (400 MHz, $CDCl_3$)
1.98
1.00
2.07
2.08
1.23
1.87
1.84
3.22
2.86
f1 (ppm)

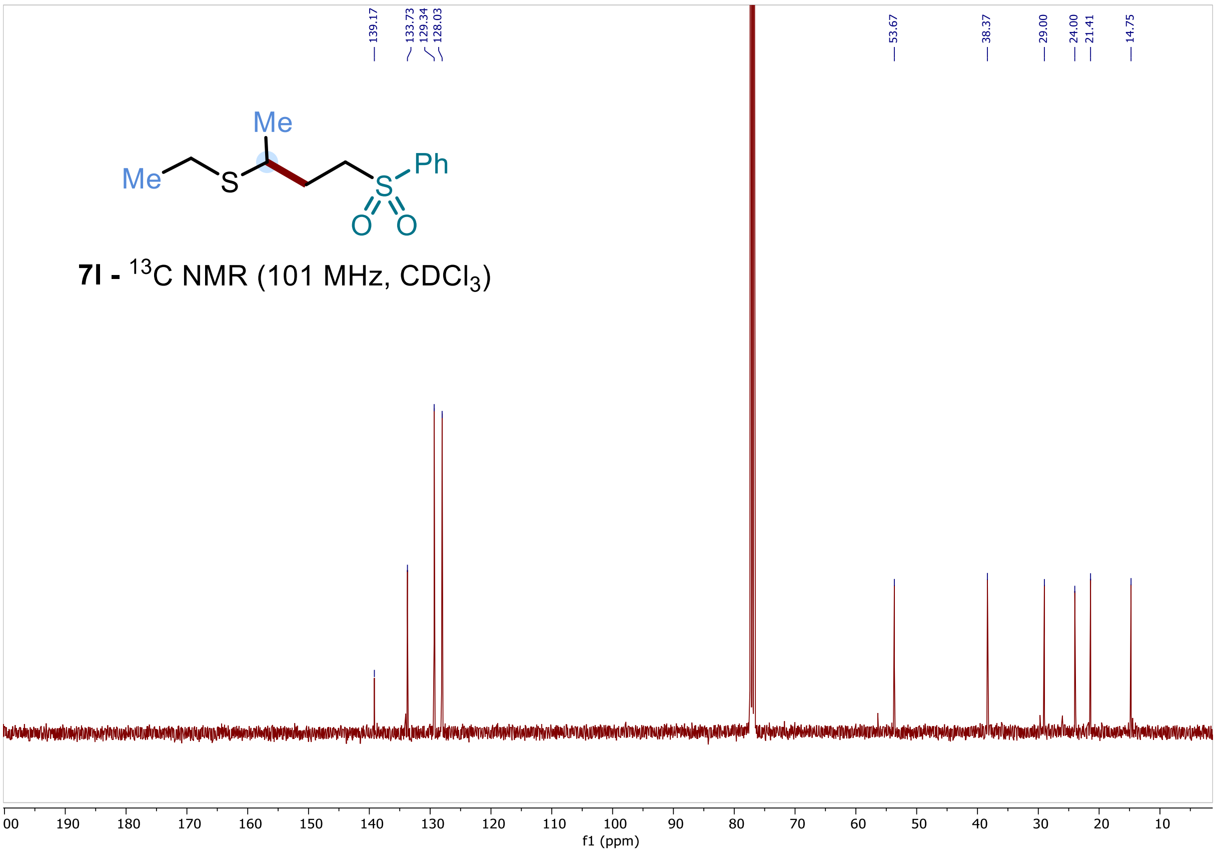
139.17
133.73
129.34
128.03
53.67
38.37
29.00
24.00
21.41
14.75
Me
Me
S
S
Ph
O O
7l - ^{13}C NMR (101 MHz, $CDCl_3$)
f1 (ppm)

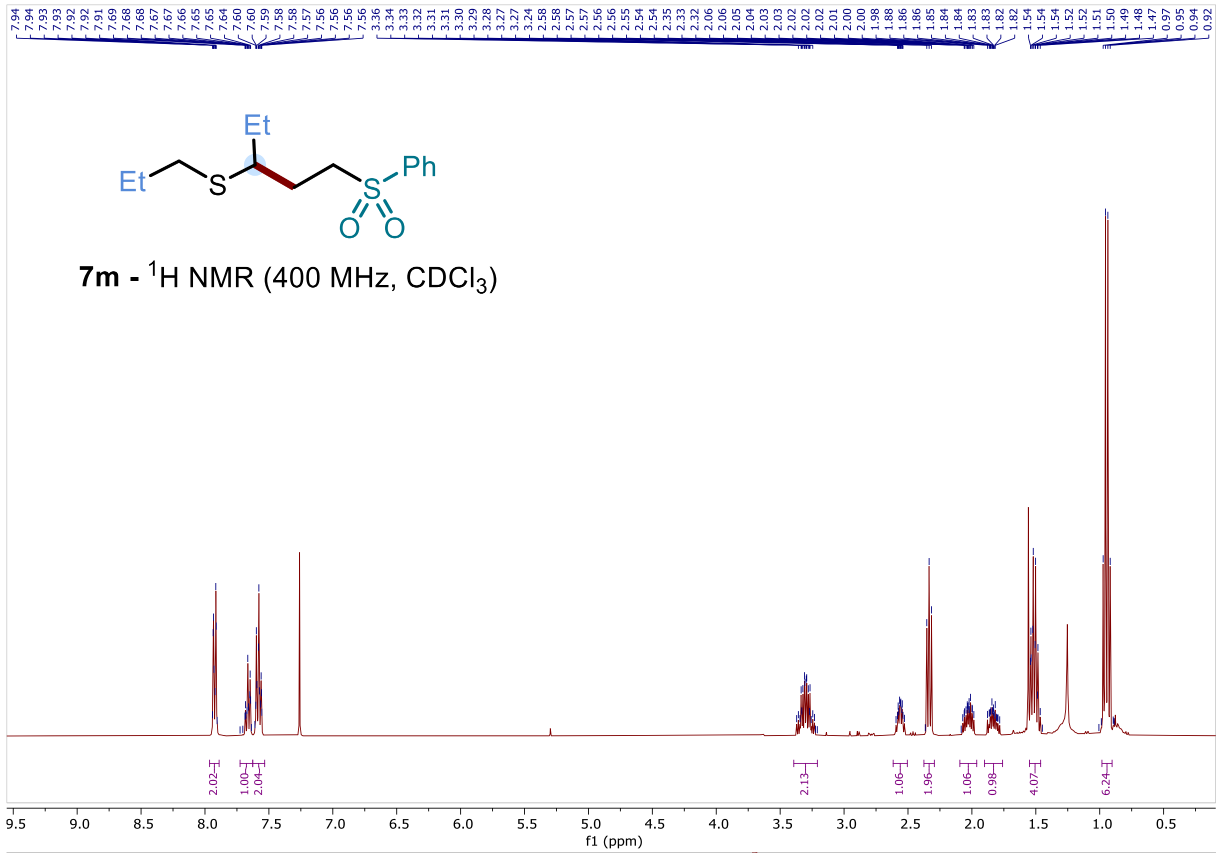

Et
Et
S
S
Ph
O
O
7m - 1H NMR (400 MHz, CDCl3)
f1 (ppm)

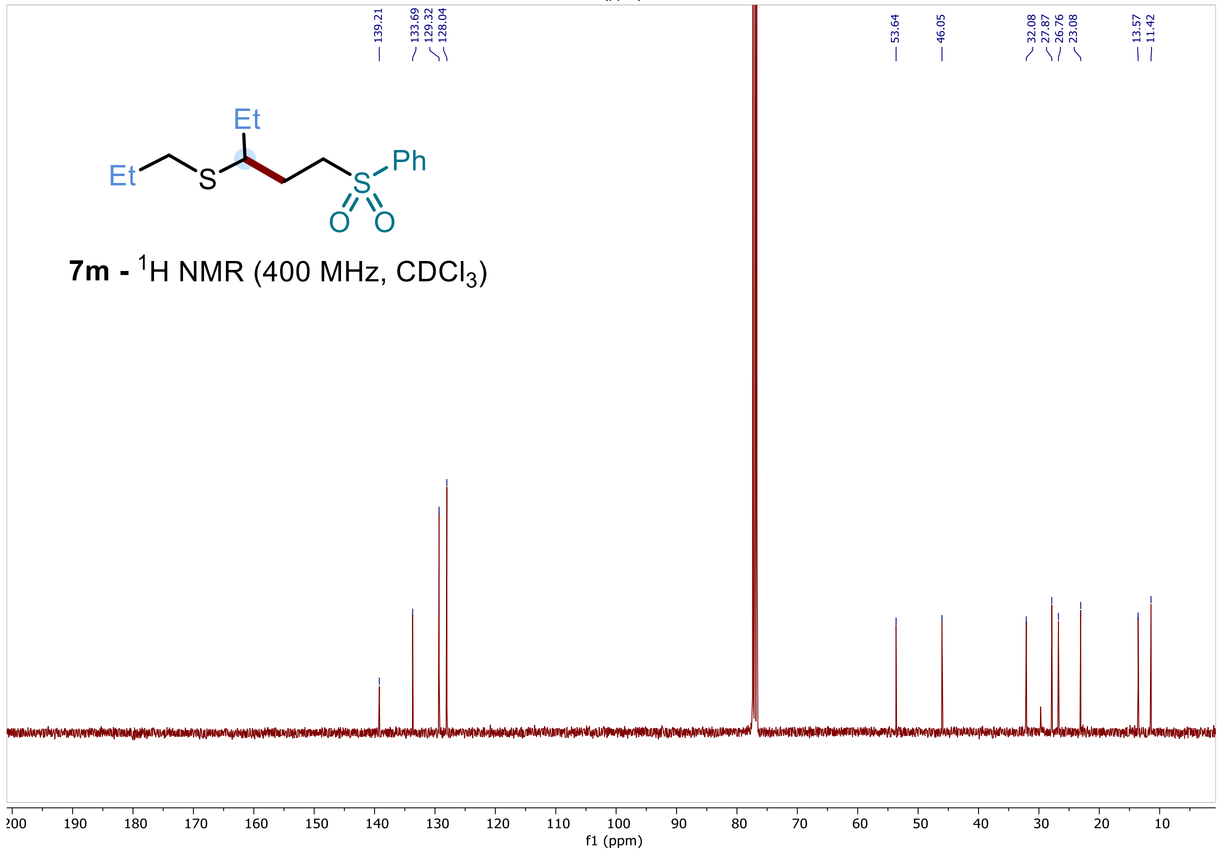

139.21
133.69
129.32
128.04
53.64
46.05
32.08
27.87
26.76
23.08
13.57
11.42
Et
Et
S
S
Ph
O
O
7m - 1H NMR (400 MHz, CDCl3)
f1 (ppm)

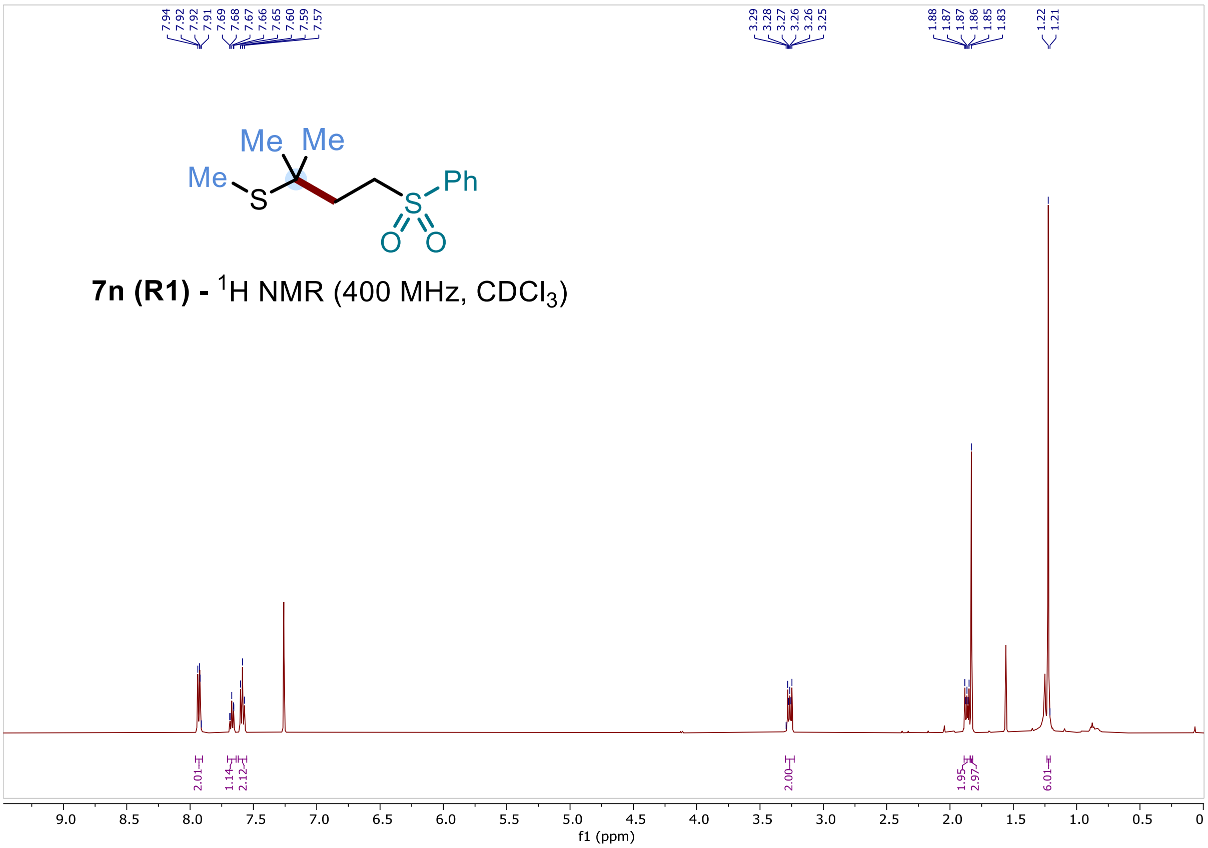
7n (R1) - 1H NMR (400 MHz, CDCl3)
Me Me
Me S Ph S O O
7.94 7.92 7.92 7.91 7.69 7.68 7.67 7.66 7.65 7.60 7.59 7.57
3.29 3.28 3.27 3.26 3.26 3.25
1.88 1.87 1.87 1.86 1.85 1.83
1.22 1.21
2.01 1.14 2.12 2.00 1.95 2.97 6.01
f1 (ppm)

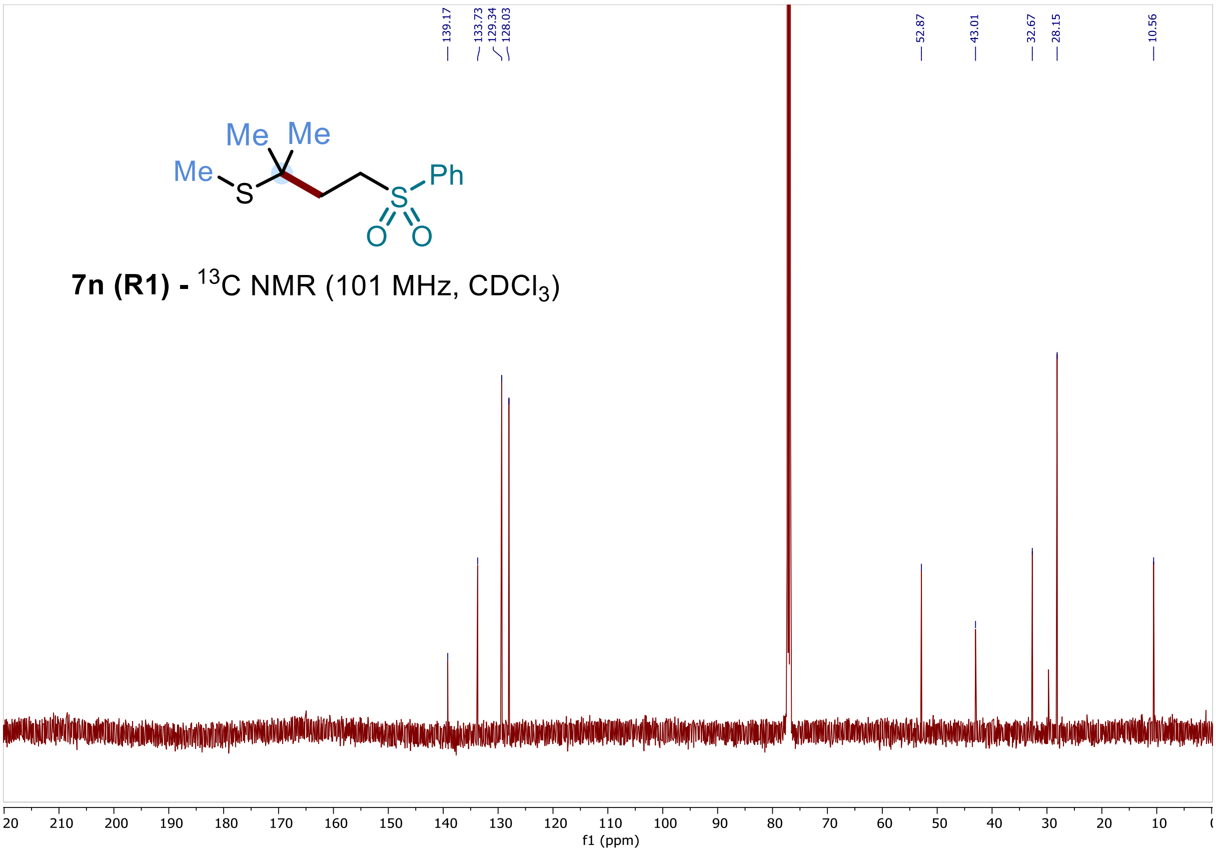
7n (R1) - 13C NMR (101 MHz, CDCl3)
Me Me
Me S Ph S O O
139.17 133.73 129.34 128.03
52.87 43.01 32.67 28.15 10.56
f1 (ppm)

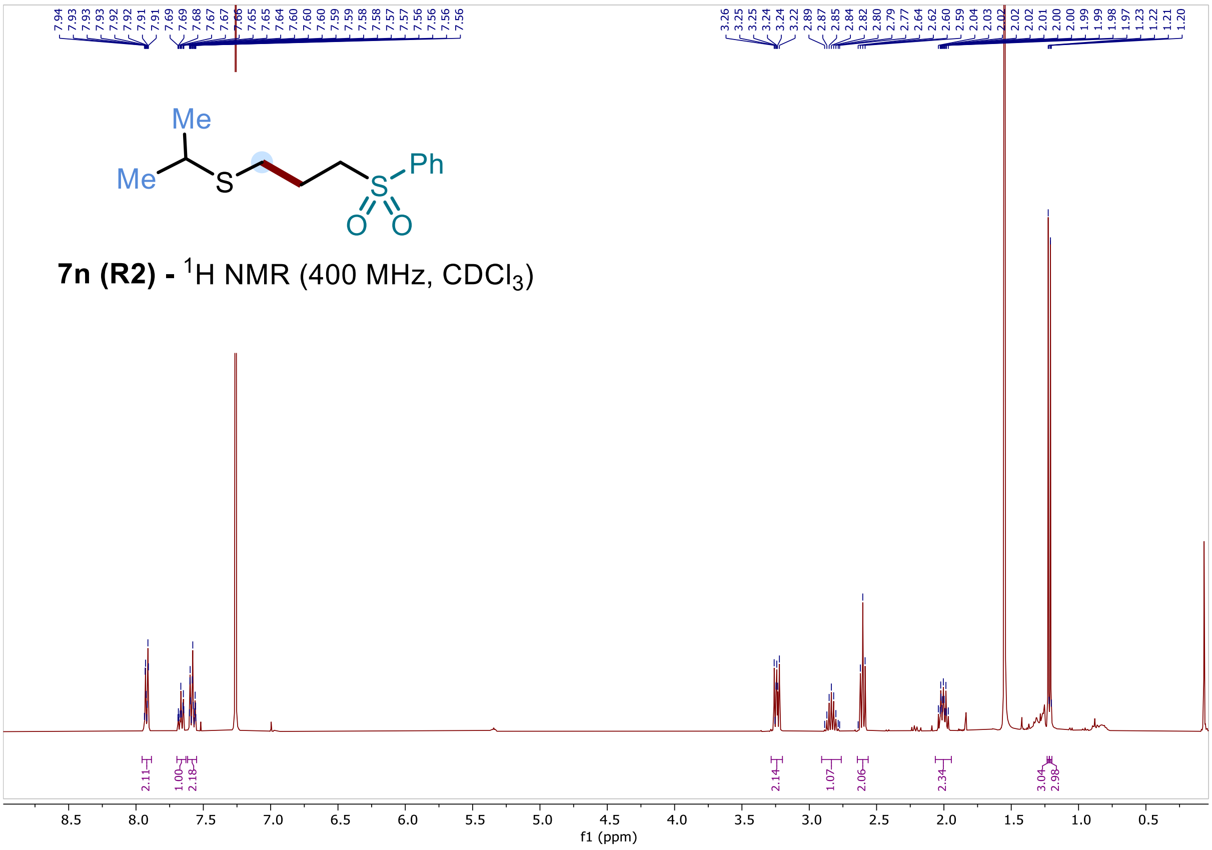
7n (R2) - ^{1}H NMR (400 MHz, CDCl$_3$)
f1 (ppm)

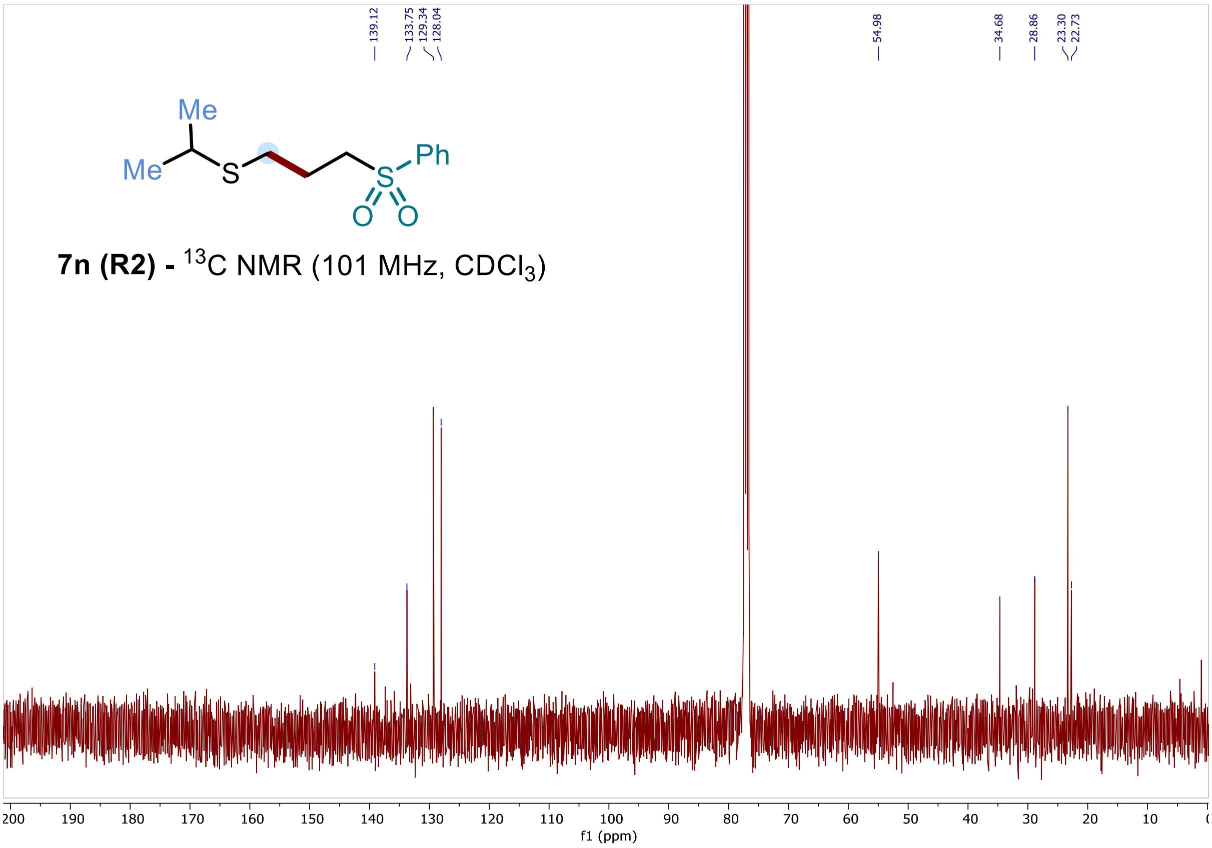
7n (R2) - ^{13}C NMR (101 MHz, CDCl$_3$)
139.12
133.75
129.34
128.04
54.98
34.68
28.86
23.30
22.73
f1 (ppm)

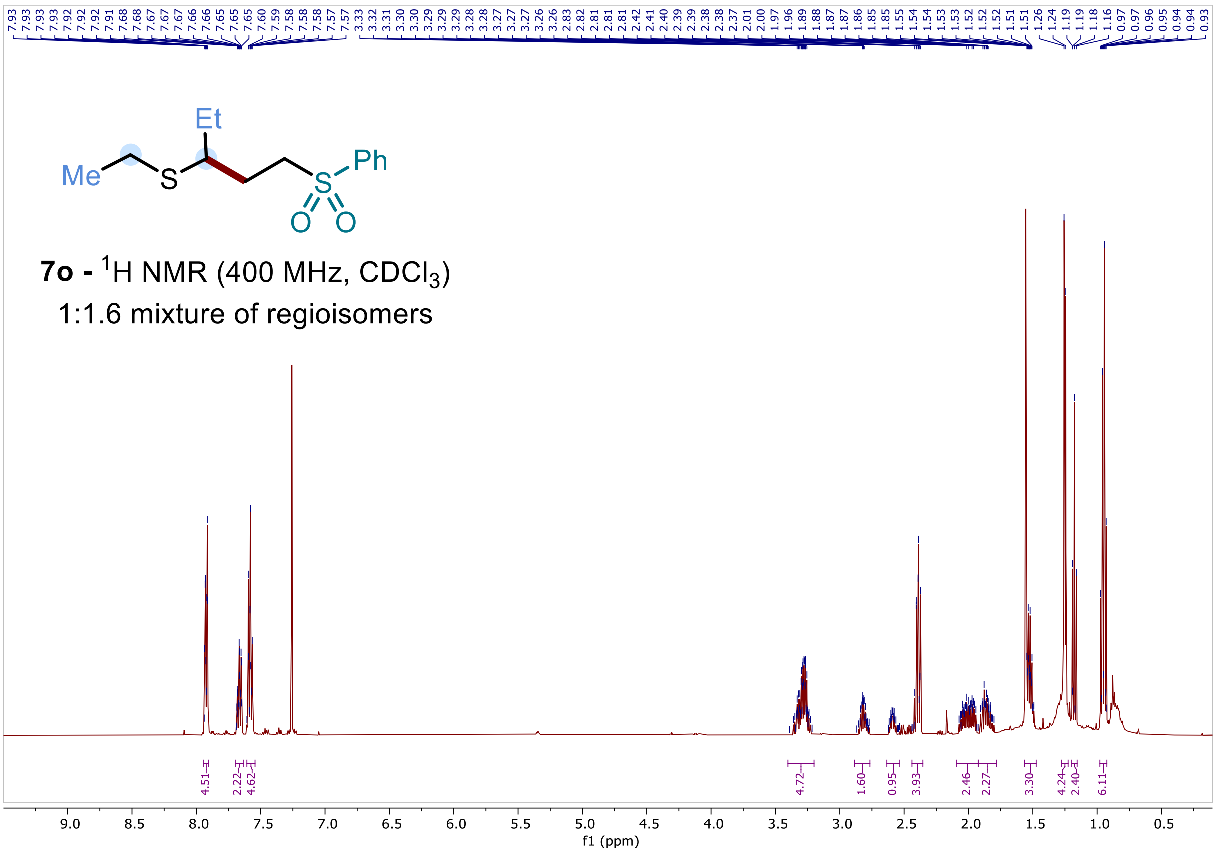
Et
Me
S
Ph
S
O O
7o - 1H NMR (400 MHz, CDCl3)
1:1.6 mixture of regioisomers
4.51 2.22 4.62 4.72 1.60 0.95 3.93 2.46 2.27 3.30 4.24 2.40 6.11
9.0 8.5 8.0 7.5 7.0 6.5 6.0 5.5 5.0 4.5 4.0 3.5 3.0 2.5 2.0 1.5 1.0 0.5
f1 (ppm)

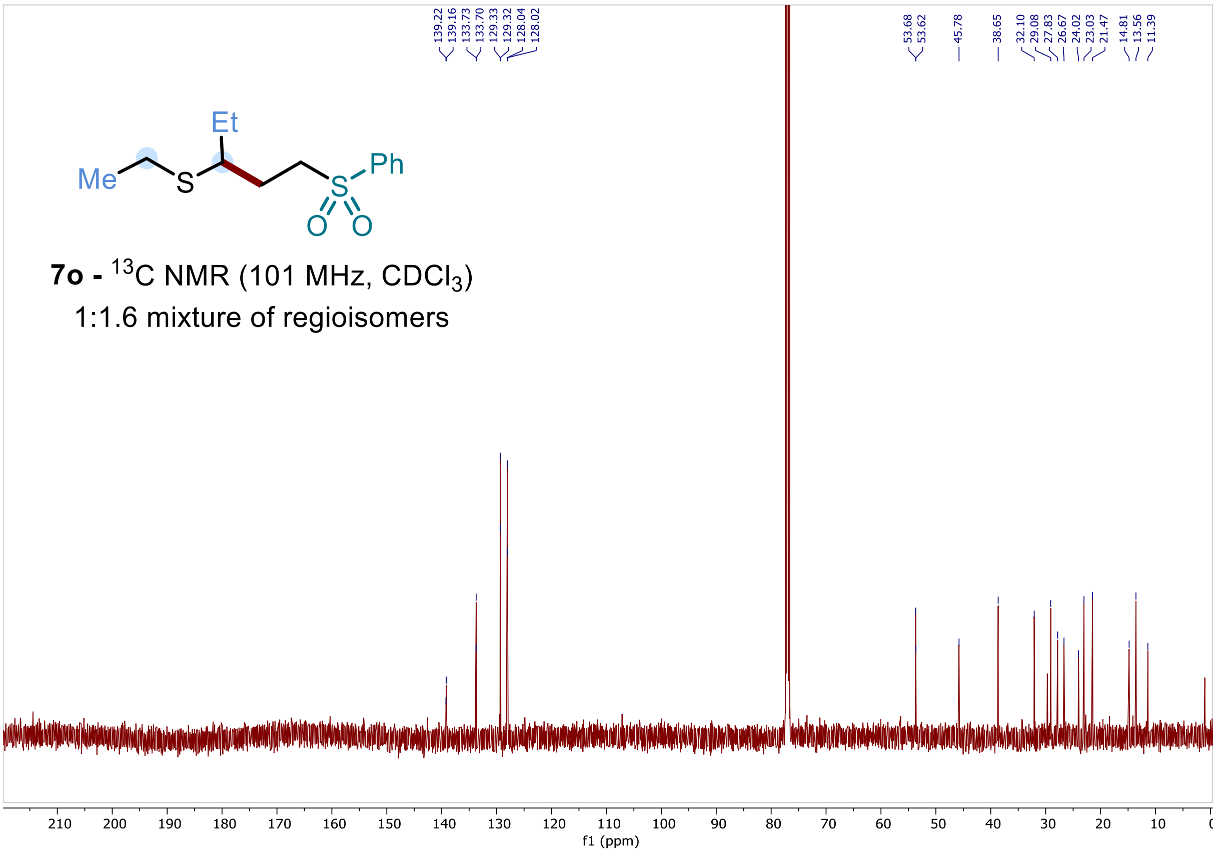
139.22 139.16 133.73 133.70 129.33 129.32 128.04 128.02
53.68 53.62 45.78 38.65 32.10 29.08 27.83 26.67 24.02 23.03 21.47 14.81 13.56 11.39
Et
Me
S
Ph
S
O O
7o - 13C NMR (101 MHz, CDCl3)
1:1.6 mixture of regioisomers
210 200 190 180 170 160 150 140 130 120 110 100 90 80 70 60 50 40 30 20 10
f1 (ppm)

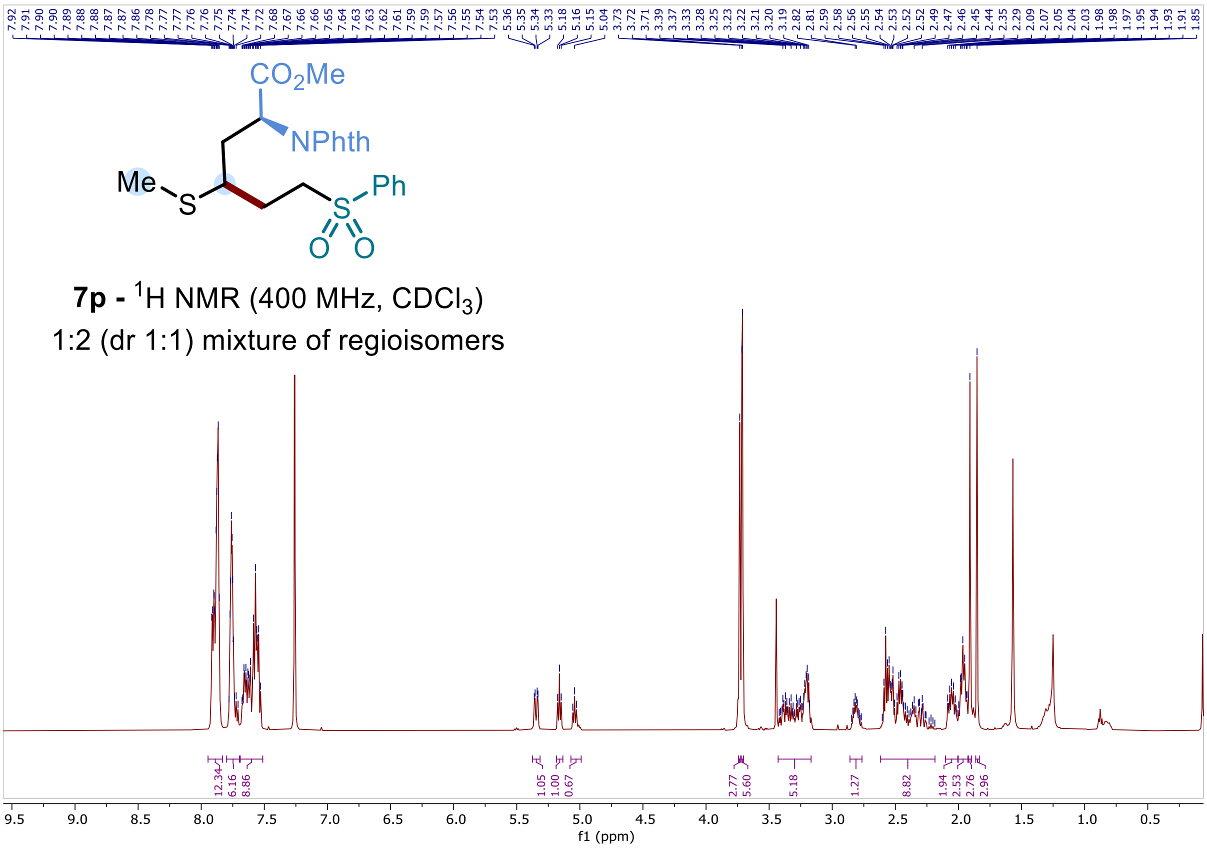
CO2Me
NPhth
Me
S
Ph
S
O O
7p - 1H NMR (400 MHz, CDCl3)
1:2 (dr 1:1) mixture of regioisomers
f1 (ppm)

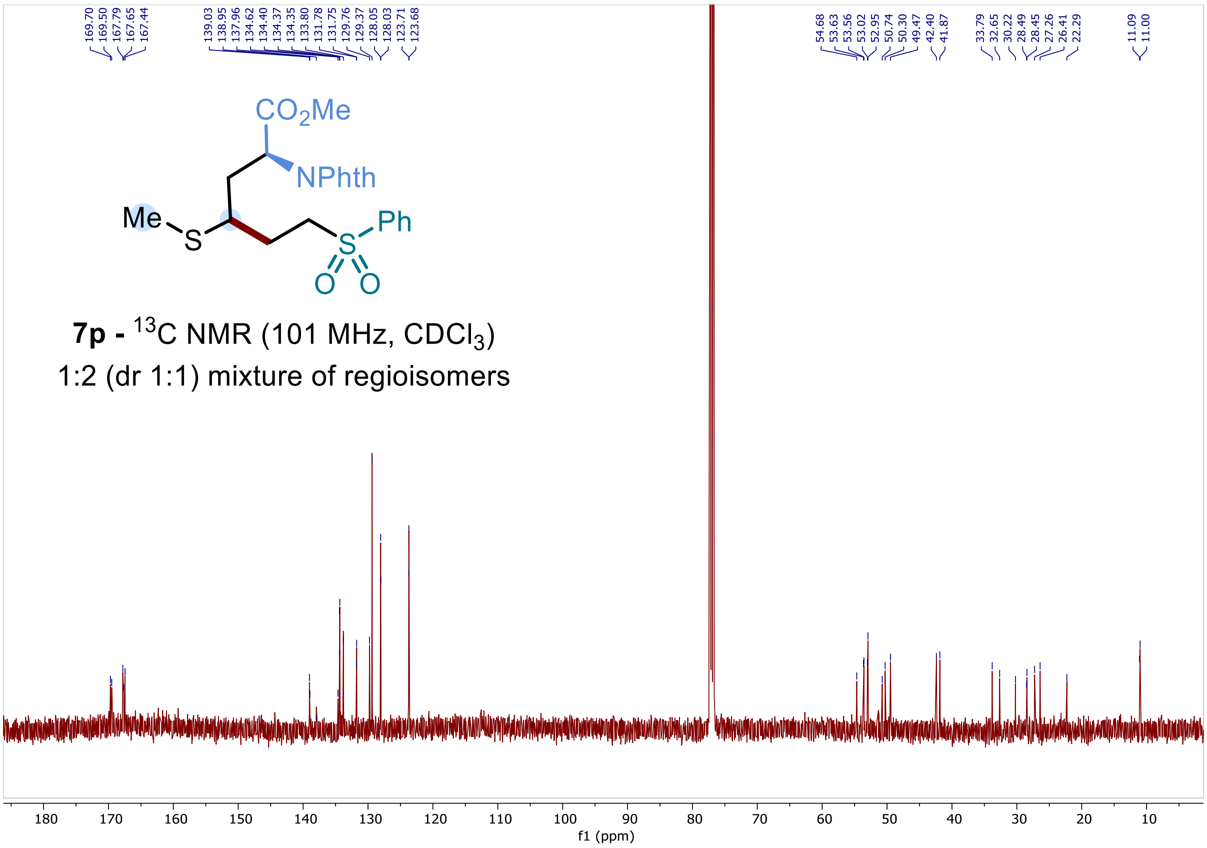
CO2Me
NPhth
Me
S
Ph
S
O O
7p - 13C NMR (101 MHz, CDCl3)
1:2 (dr 1:1) mixture of regioisomers
f1 (ppm)

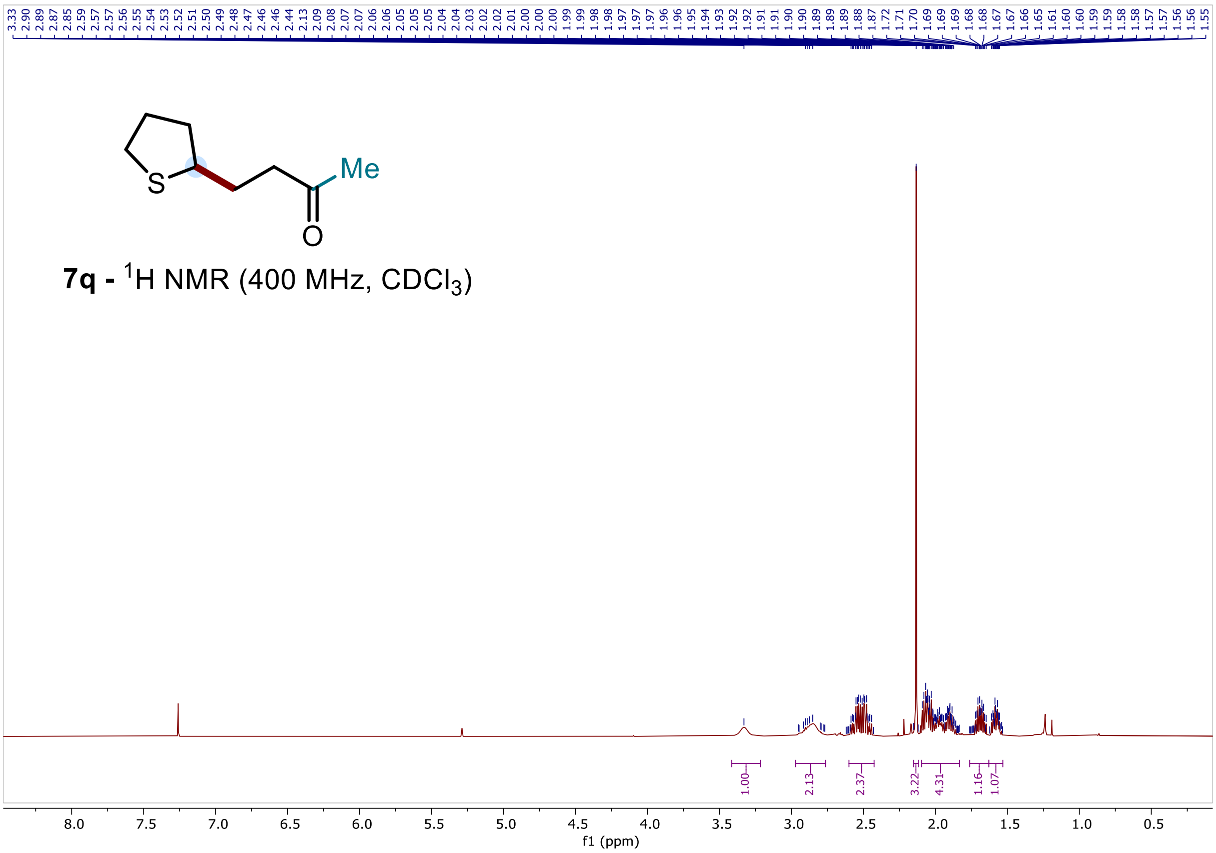
Me
S
O
7q - ¹H NMR (400 MHz, CDCl₃)
f1 (ppm)

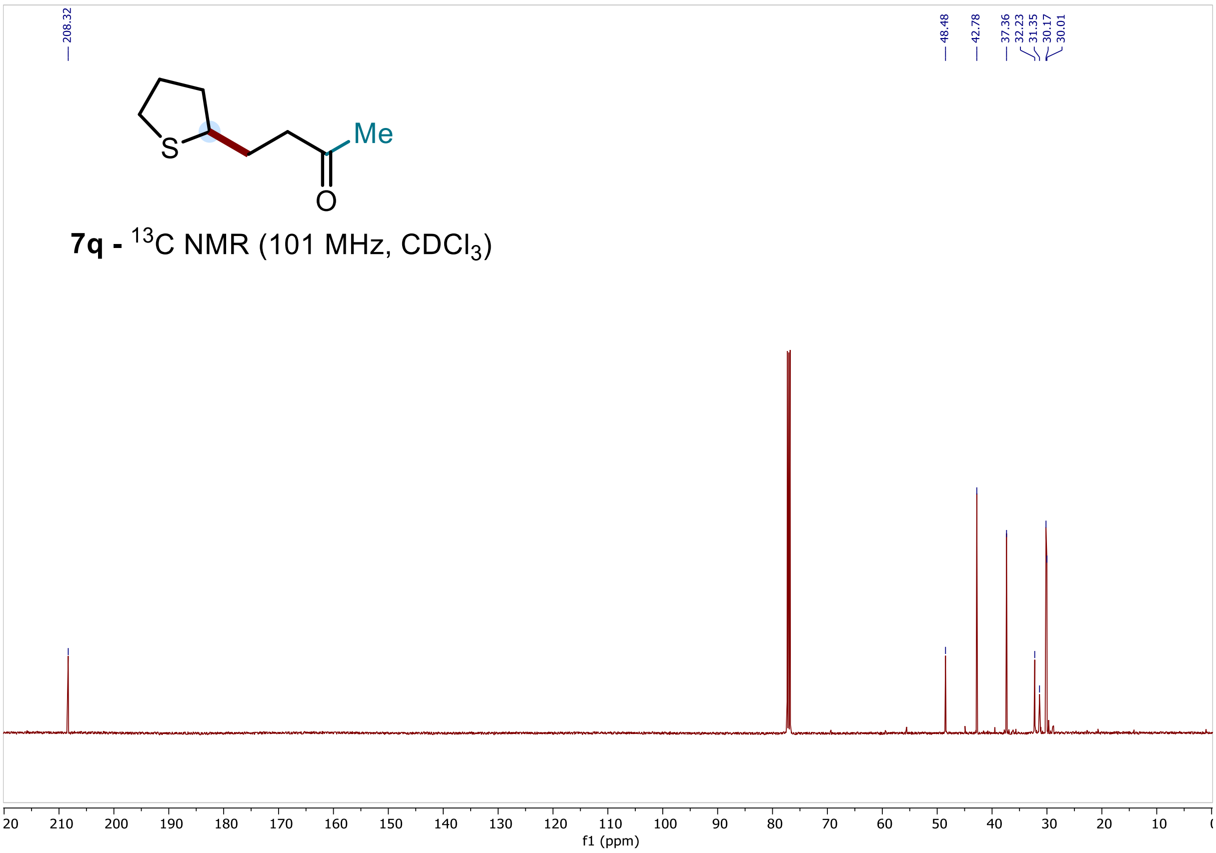
208.32
48.48
42.78
37.36
32.23
31.35
30.17
30.01
Me
S
O
7q - ¹³C NMR (101 MHz, CDCl₃)
f1 (ppm)

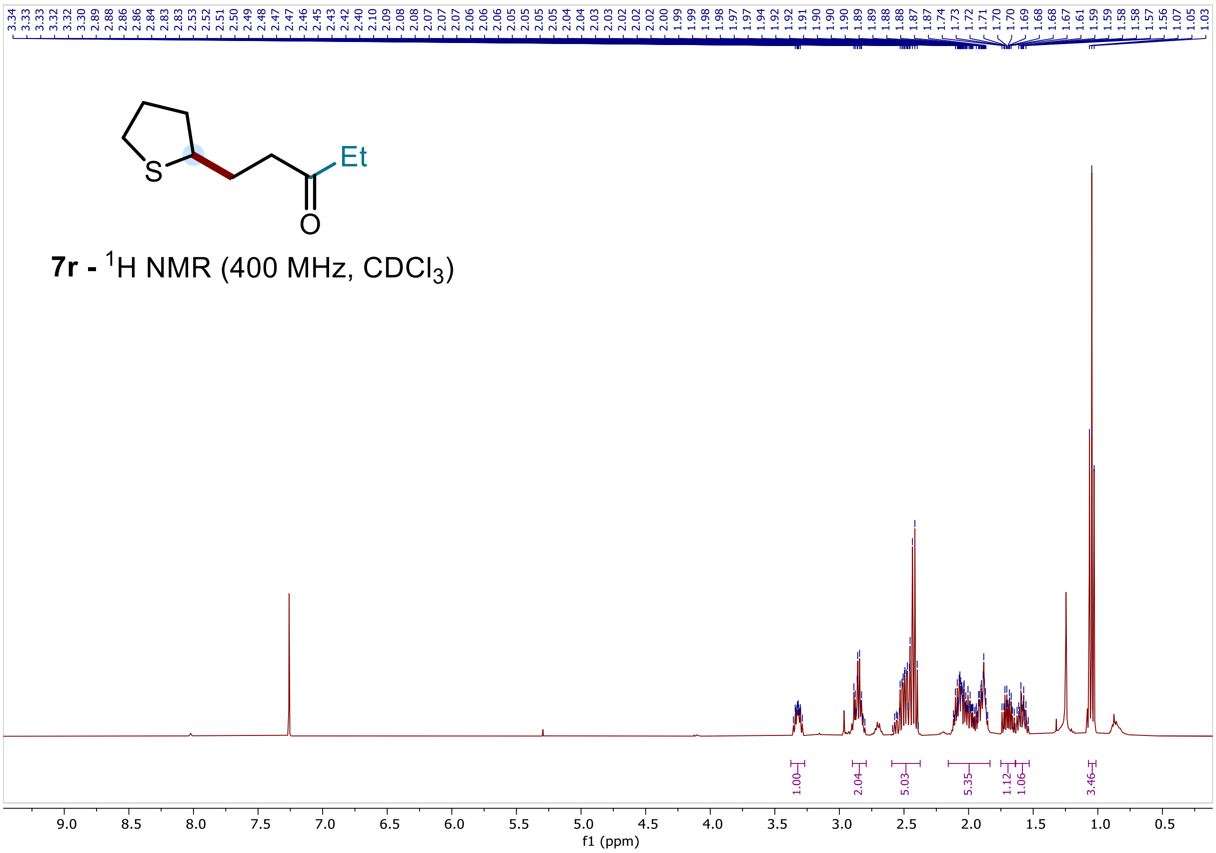

S
Et
O
7r - ¹H NMR (400 MHz, CDCl₃)
1.00
2.04
5.03
5.35
1.12
1.06
3.46
f1 (ppm)

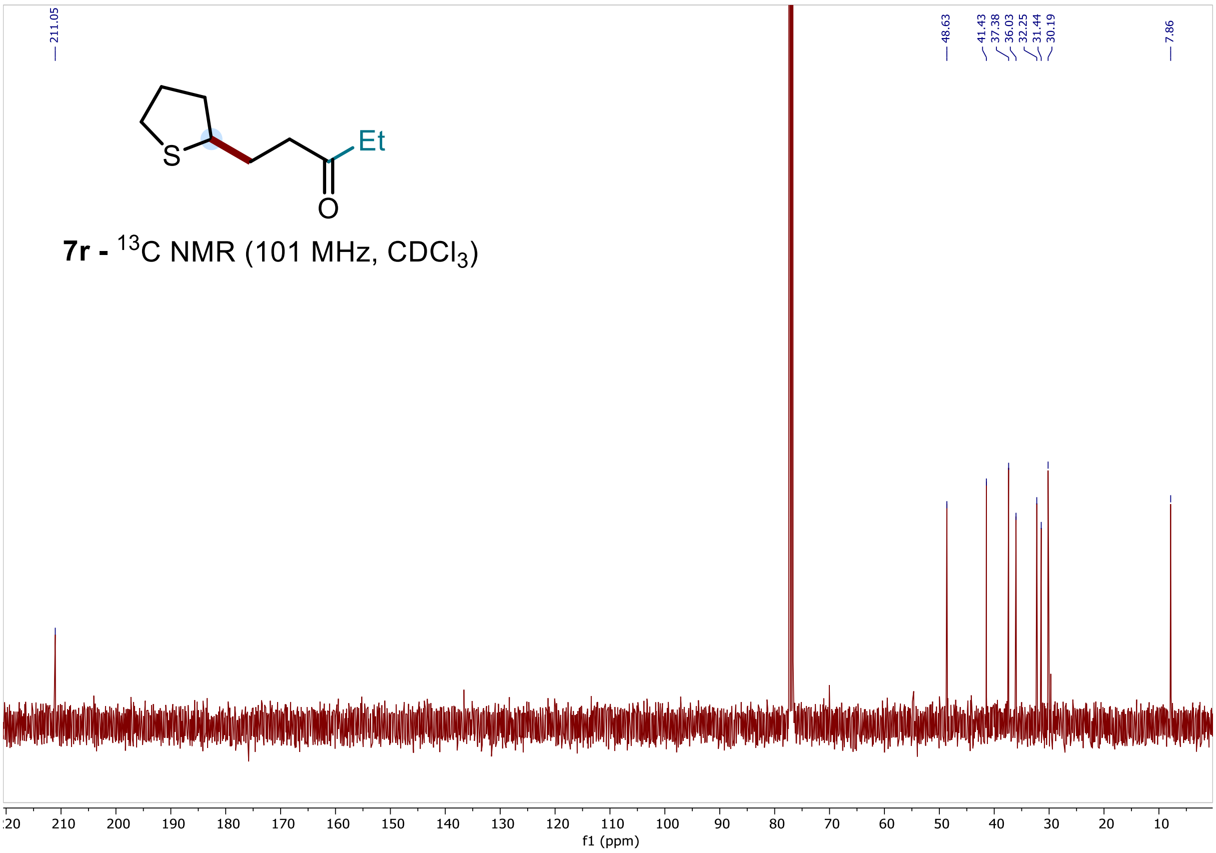

211.05
48.63
41.43
37.38
36.03
32.25
31.44
30.19
7.86
S
Et
O
7r - ¹³C NMR (101 MHz, CDCl₃)
f1 (ppm)

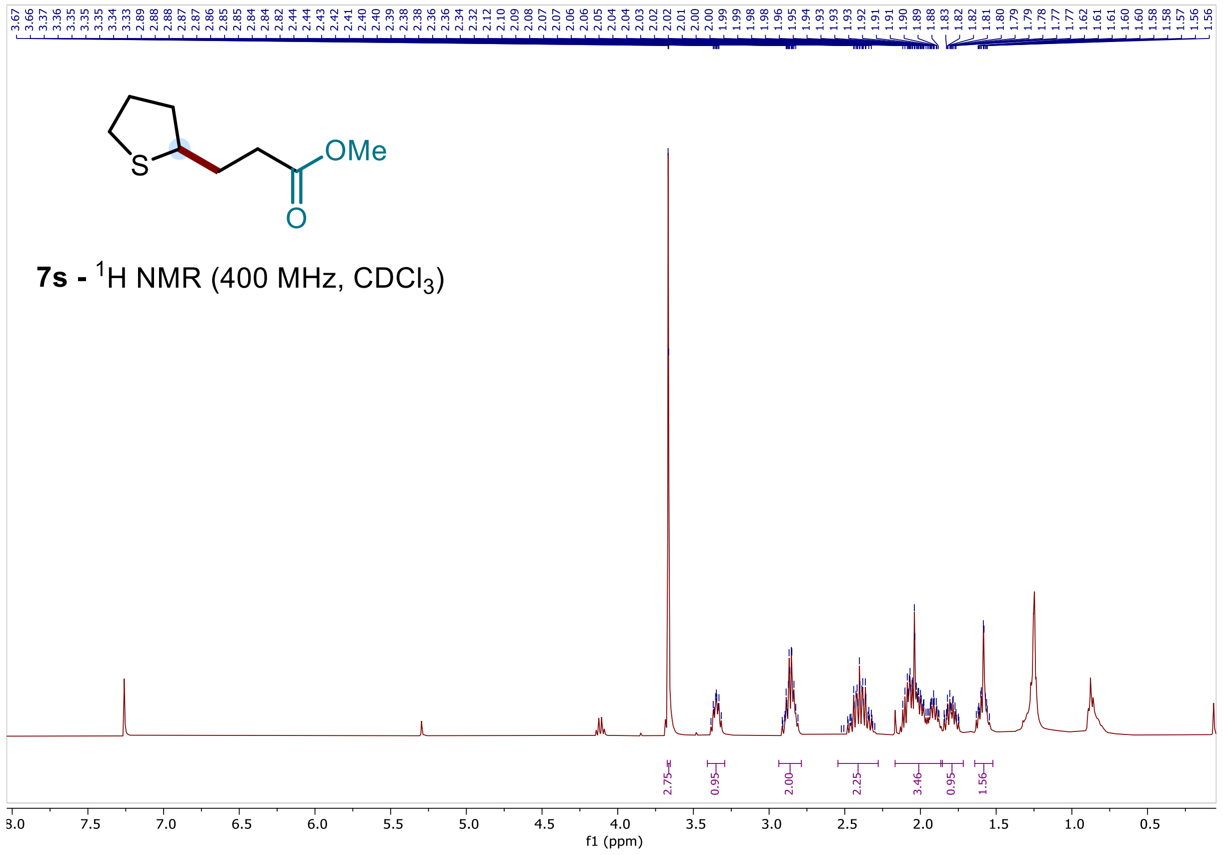

OMe
S
O
7s - ^{1}H NMR (400 MHz, CDCl$_3$)
f1 (ppm)

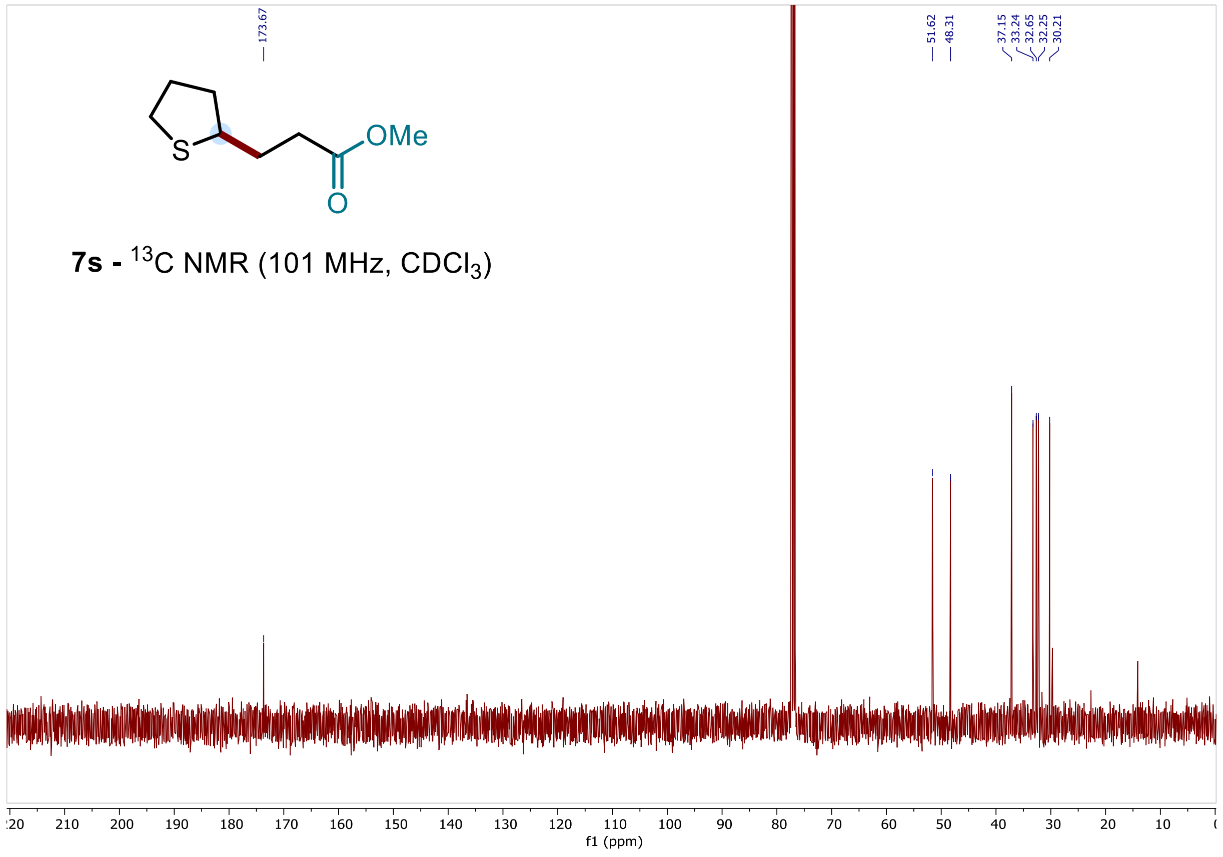

OMe
S
O
7s - ^{13}C NMR (101 MHz, CDCl$_3$)
173.67
51.62
48.31
37.15
33.24
32.65
32.25
30.21
f1 (ppm)

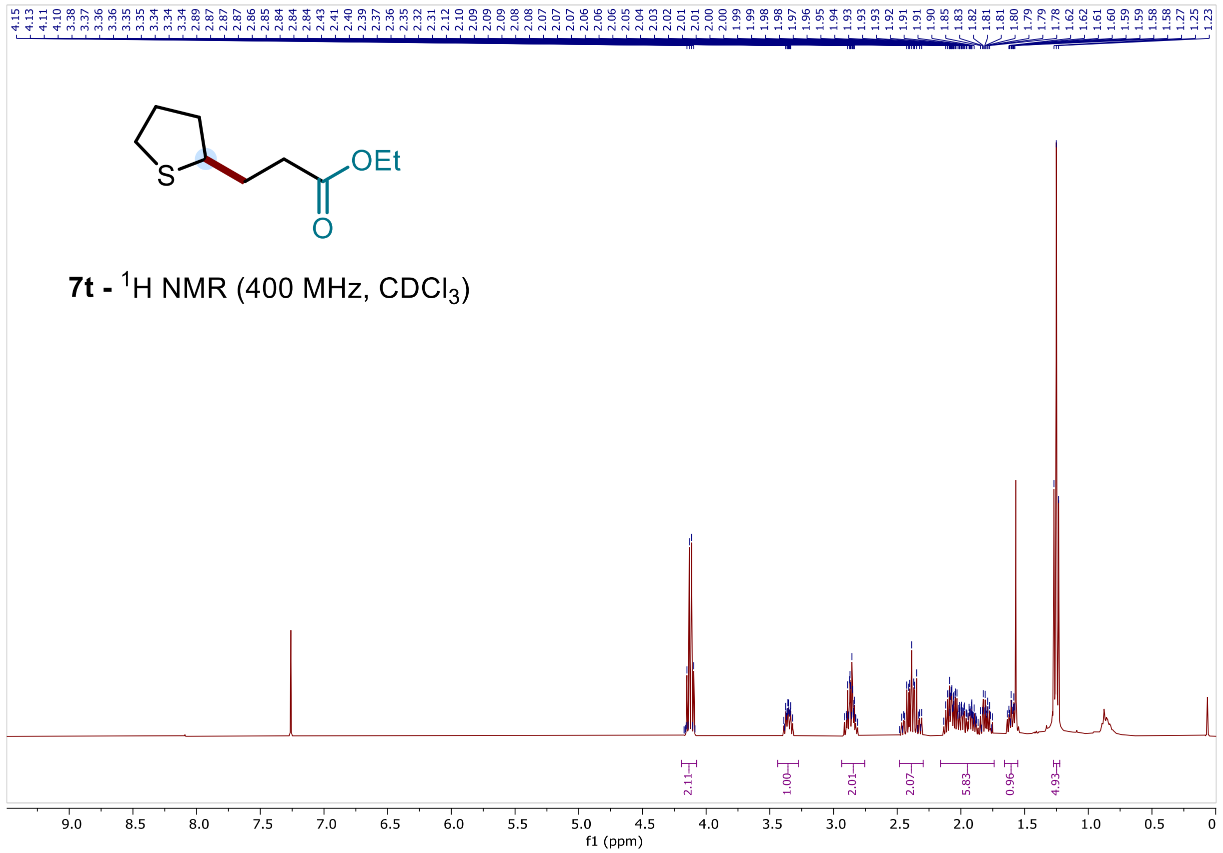
OEt
S
O
7t - ^{1}H NMR (400 MHz, CDCl$_3$)
2.11
1.00
2.01
2.07
5.83
0.96
4.93
f1 (ppm)

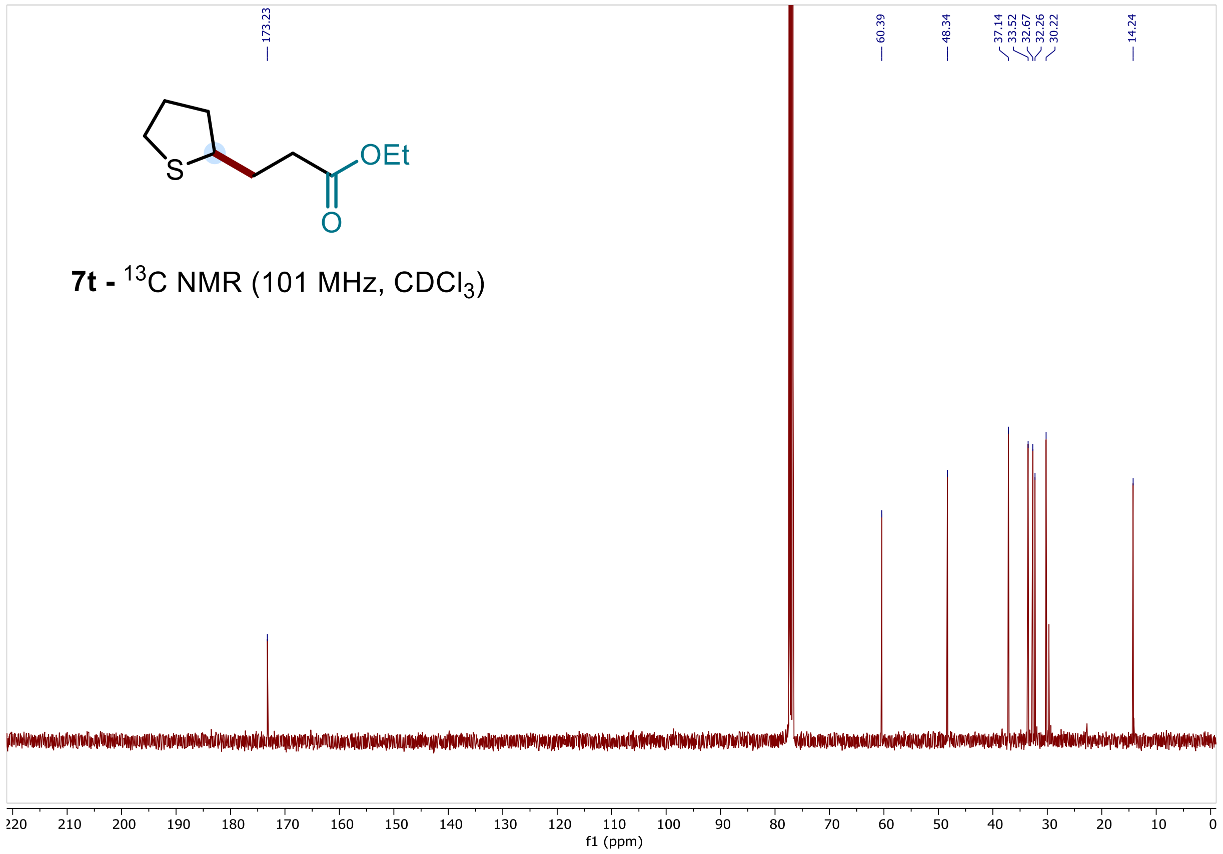
173.23
60.39
48.34
37.14
33.52
32.67
32.26
30.22
14.24
OEt
S
O
7t - ^{13}C NMR (101 MHz, CDCl$_3$)
f1 (ppm)

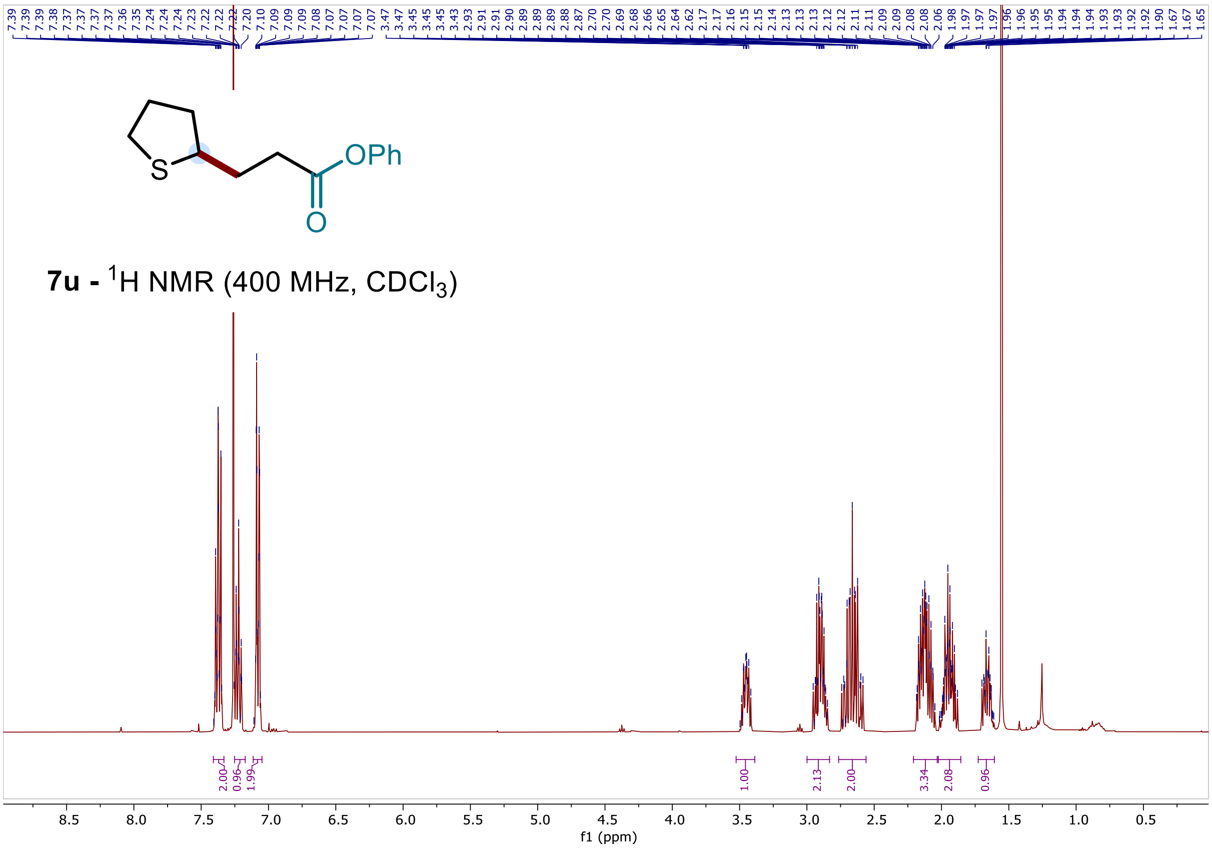

OPh
S
O
7u - 1H NMR (400 MHz, CDCl3)
f1 (ppm)

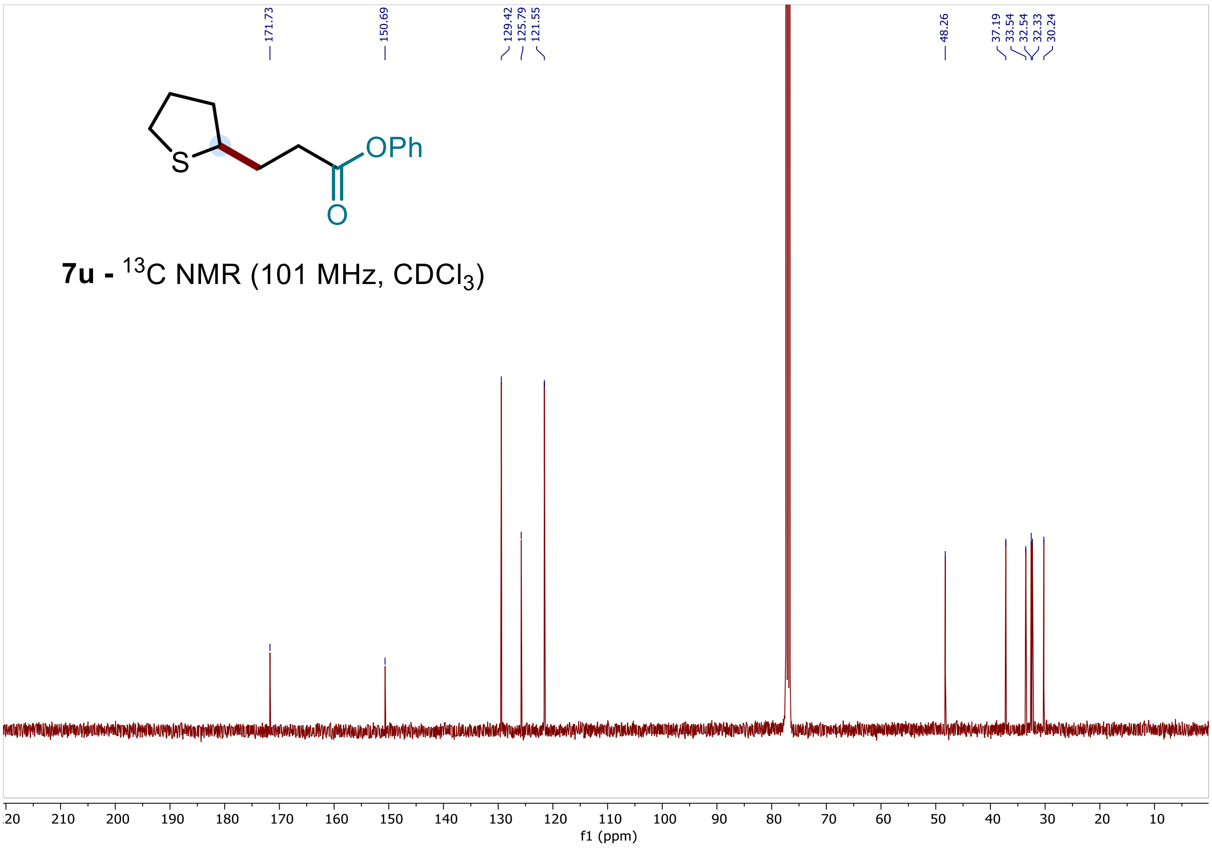

171.73
150.69
129.42
125.79
121.55
48.26
37.19
33.54
32.54
32.33
30.24
OPh
S
O
7u - 13C NMR (101 MHz, CDCl3)
f1 (ppm)

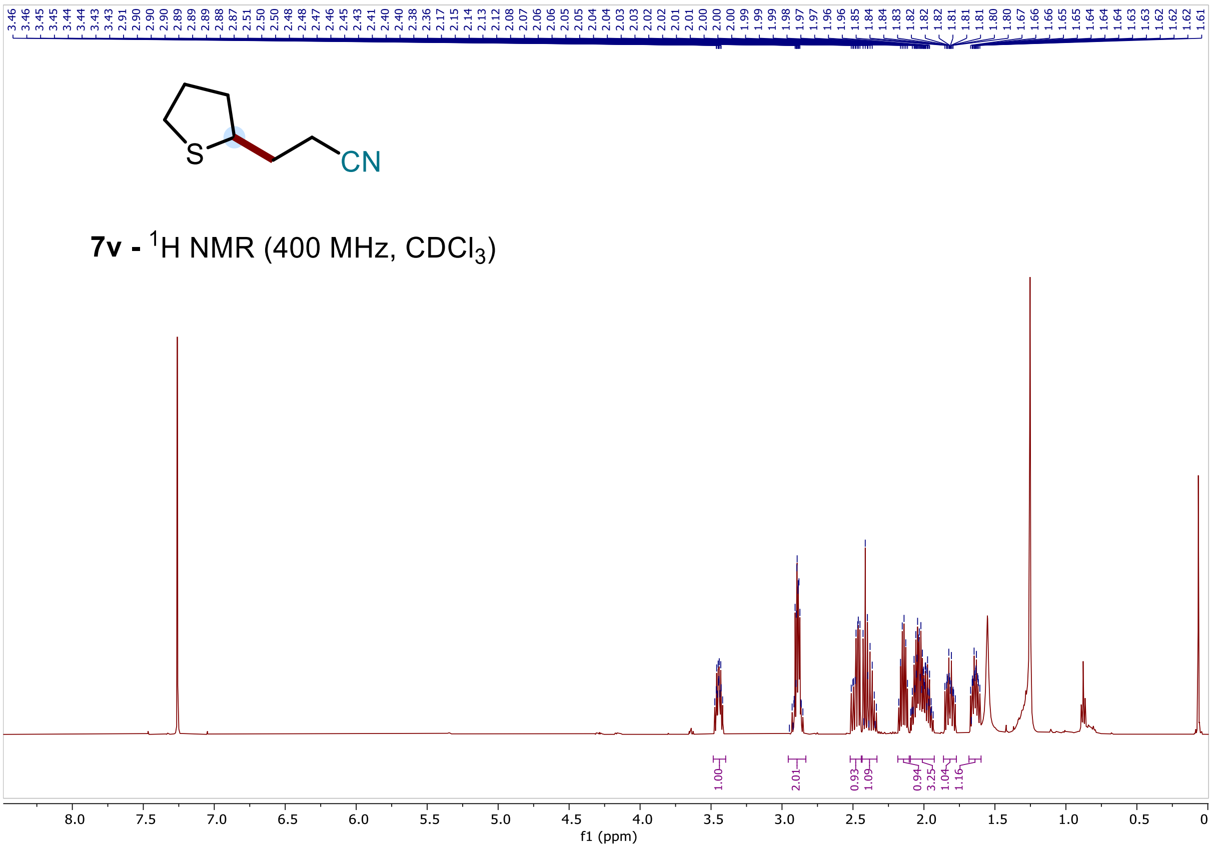

CN
7v - ¹H NMR (400 MHz, CDCl₃)
f1 (ppm)

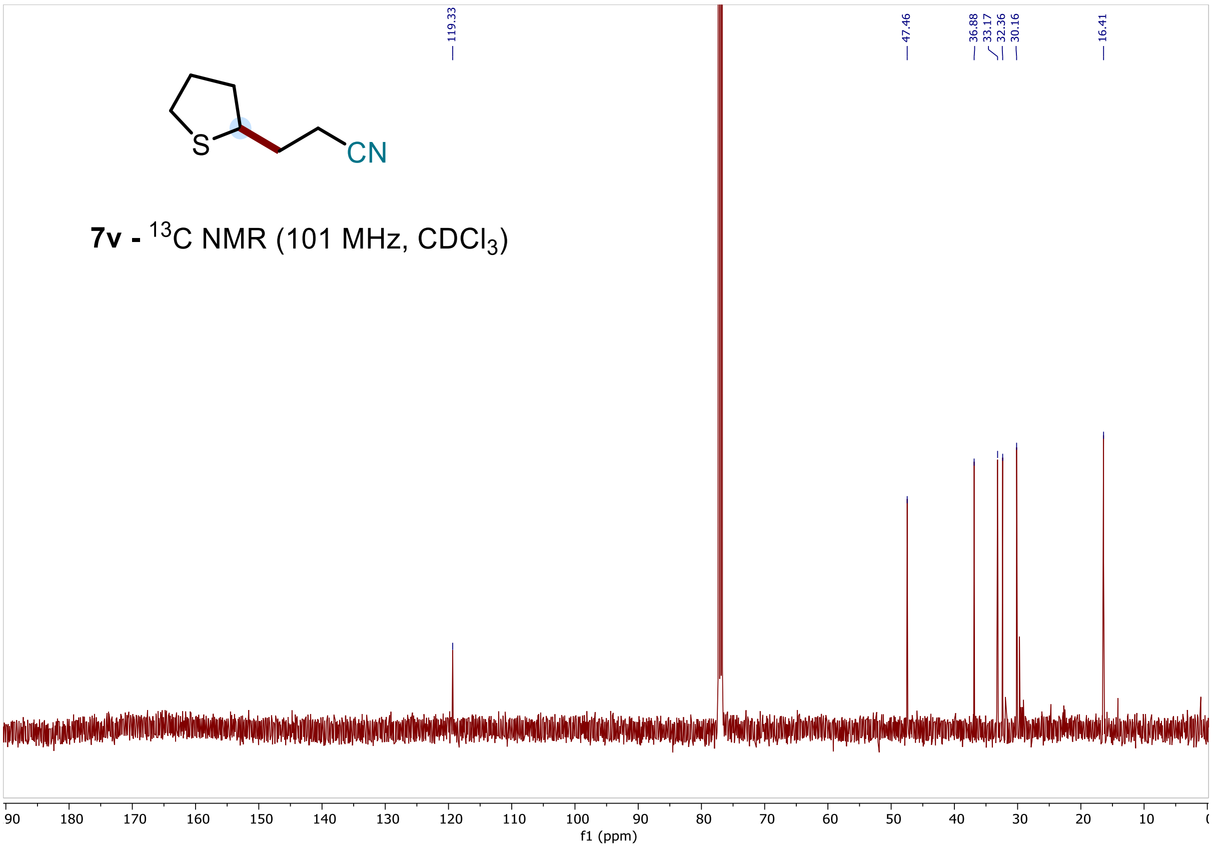

CN
7v - ¹³C NMR (101 MHz, CDCl₃)
119.33
47.46
36.88
33.17
32.36
30.16
16.41
f1 (ppm)

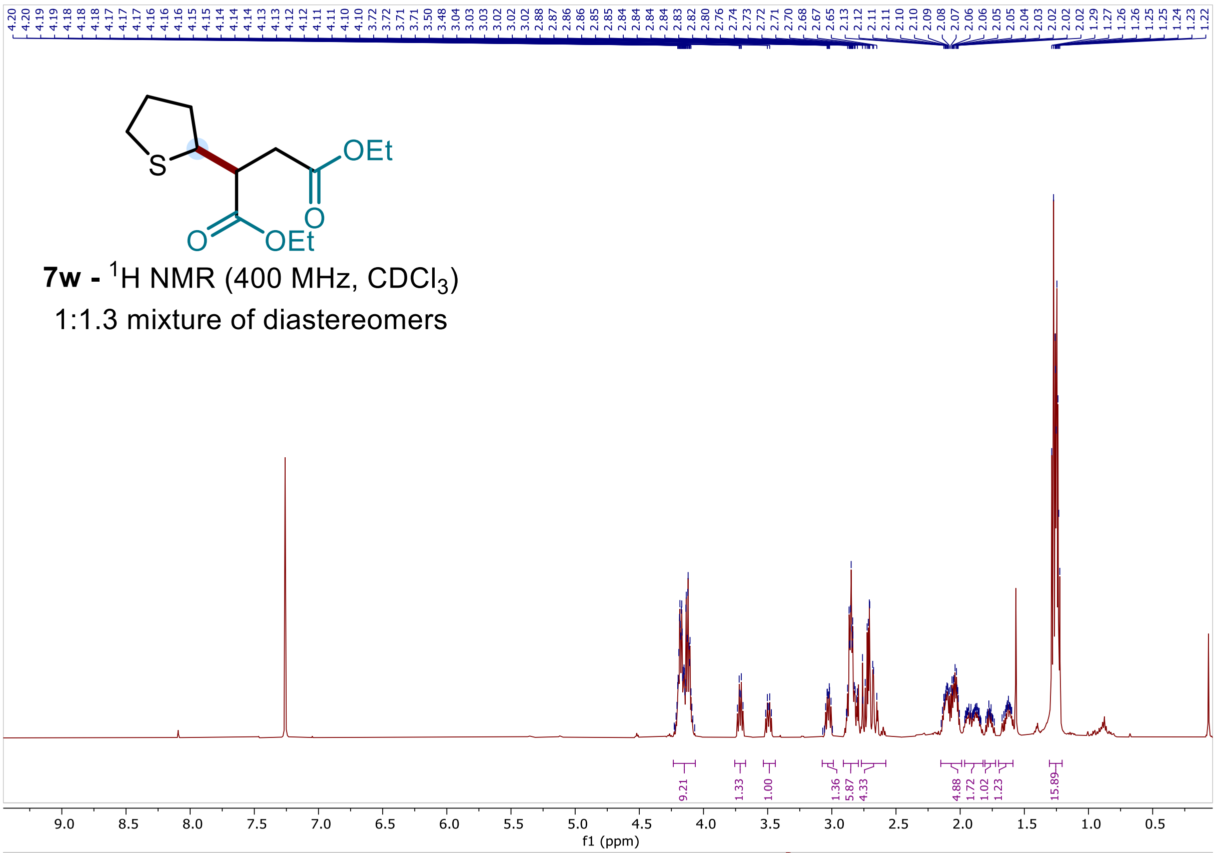
S
OEt
O
O
OEt
7w - ¹H NMR (400 MHz, CDCl₃)
1:1.3 mixture of diastereomers
f1 (ppm)

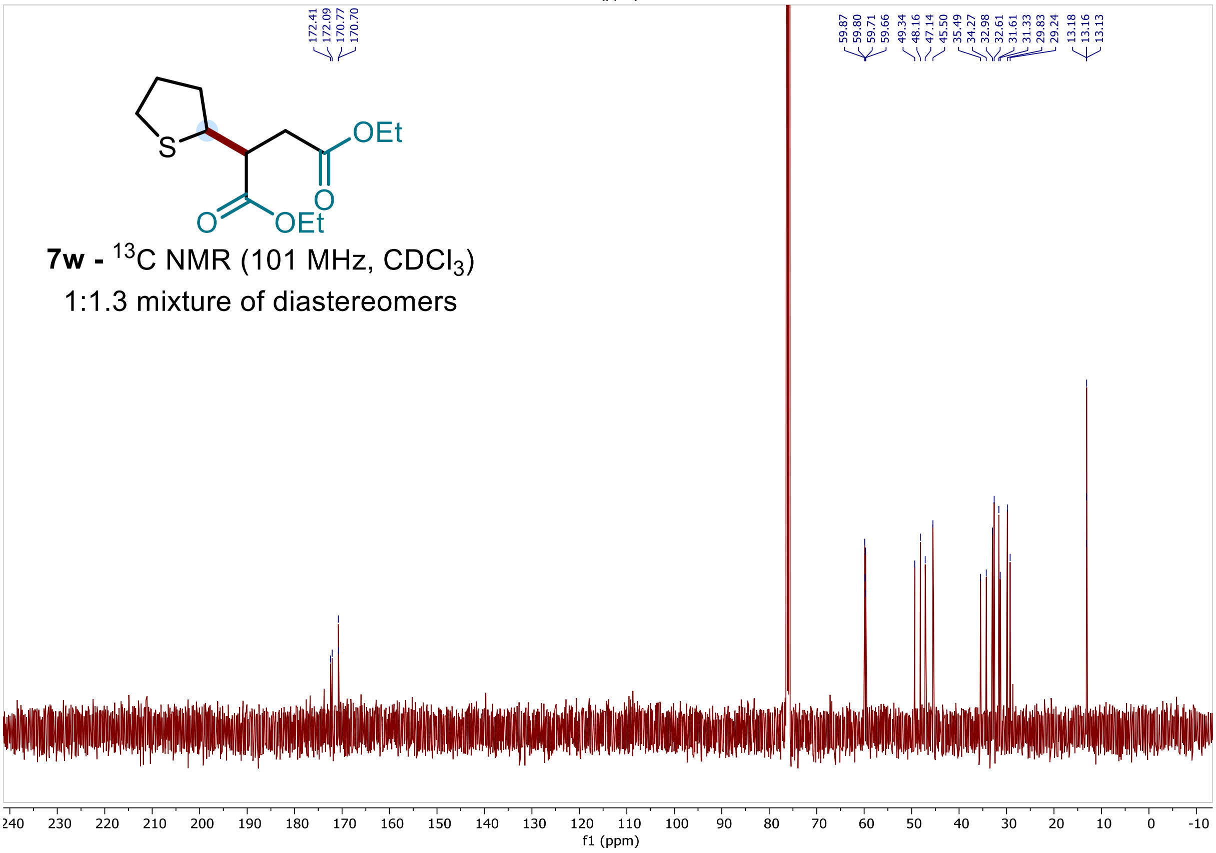
S
OEt
O
O
OEt
7w - ¹³C NMR (101 MHz, CDCl₃)
1:1.3 mixture of diastereomers
f1 (ppm)

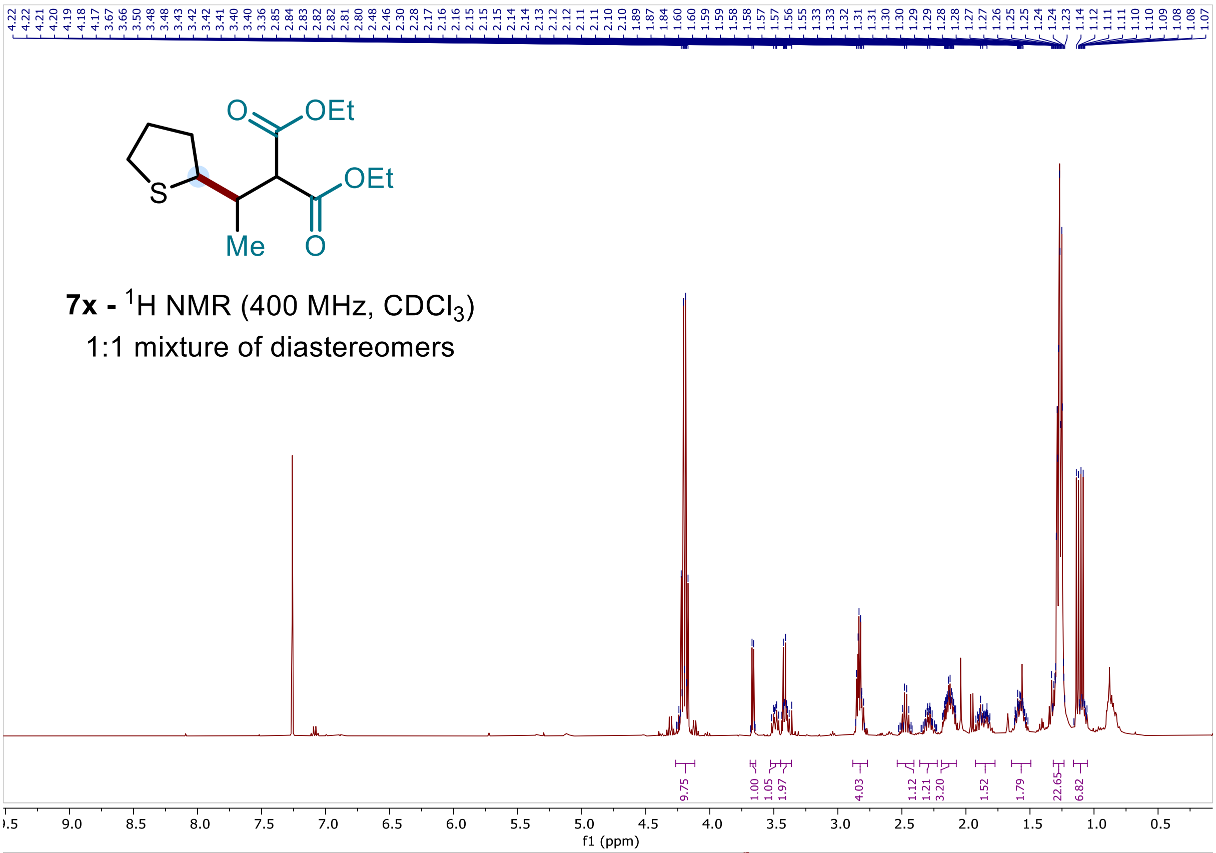
7x - 1H NMR (400 MHz, CDCl3)
1:1 mixture of diastereomers
f1 (ppm)

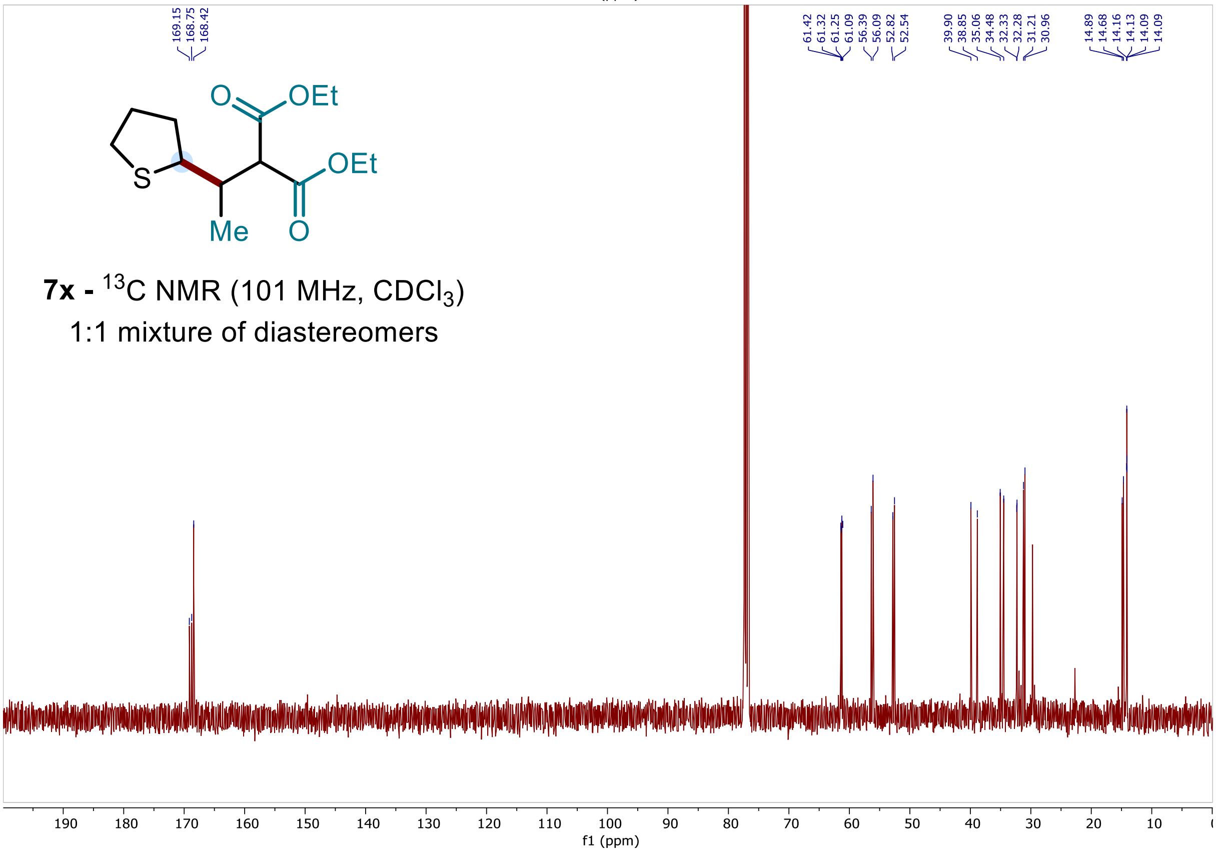
7x - 13C NMR (101 MHz, CDCl3)
1:1 mixture of diastereomers
f1 (ppm)

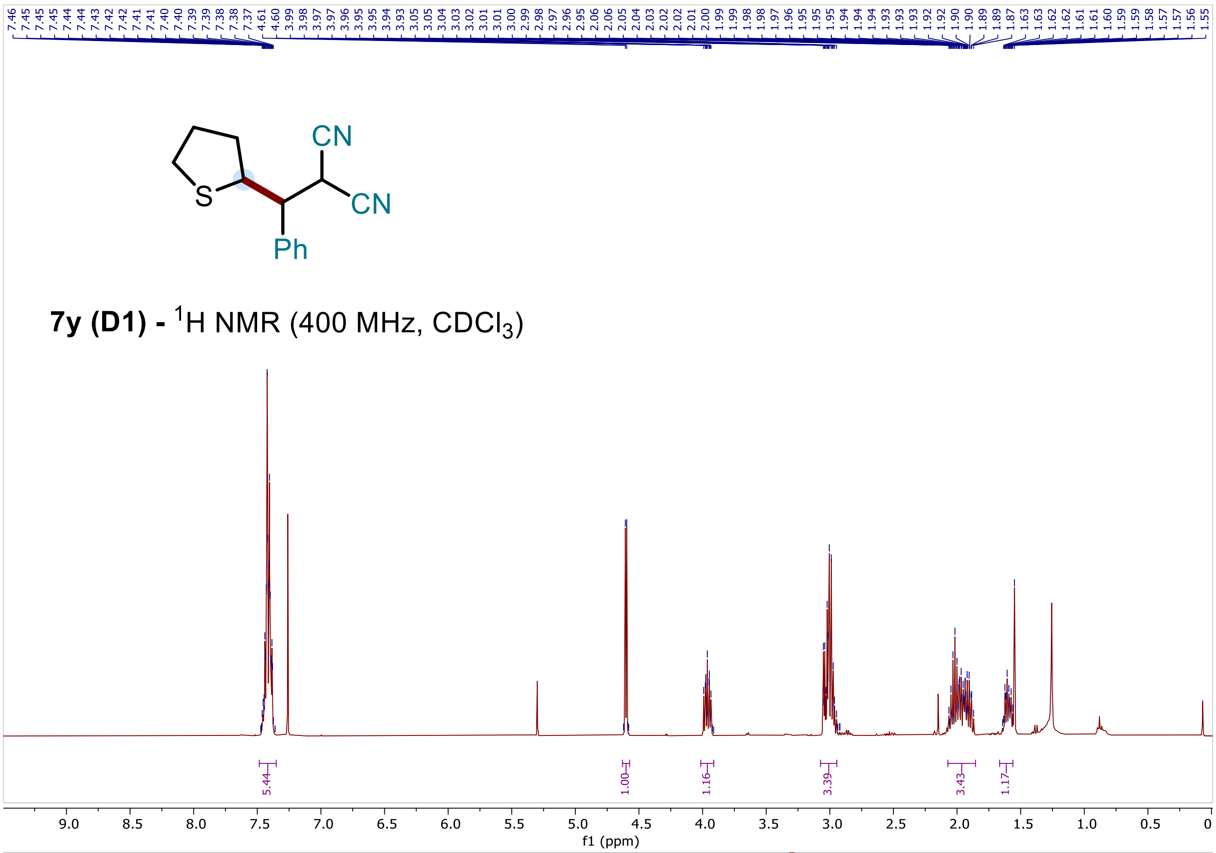
7y (D1) - ^{1}H NMR (400 MHz, CDCl$_3$)
CN
CN
S
Ph
5.44
1.00
1.16
3.39
3.43
1.17
f1 (ppm)

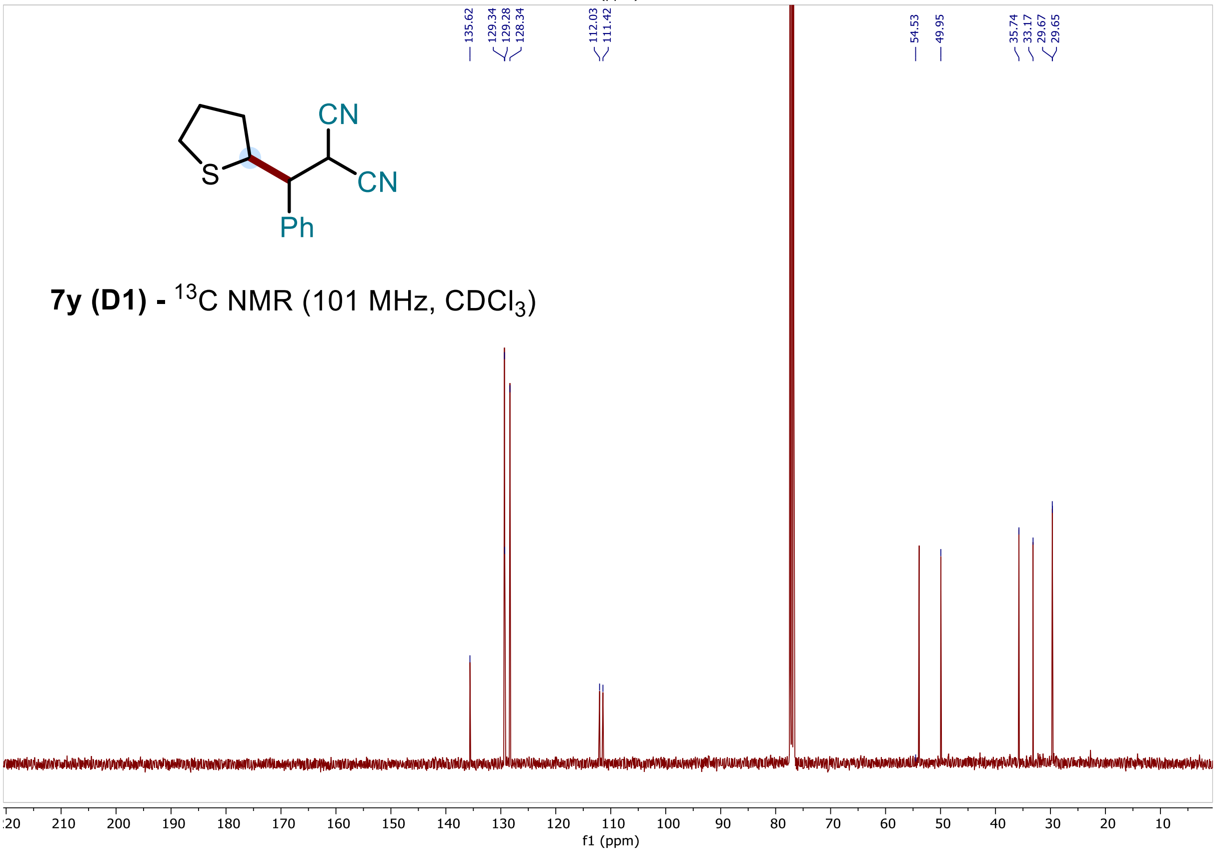
7y (D1) - ^{13}C NMR (101 MHz, CDCl$_3$)
CN
CN
S
Ph
135.62
129.34
129.28
128.34
112.03
111.42
54.53
49.95
35.74
33.17
29.67
29.65
f1 (ppm)

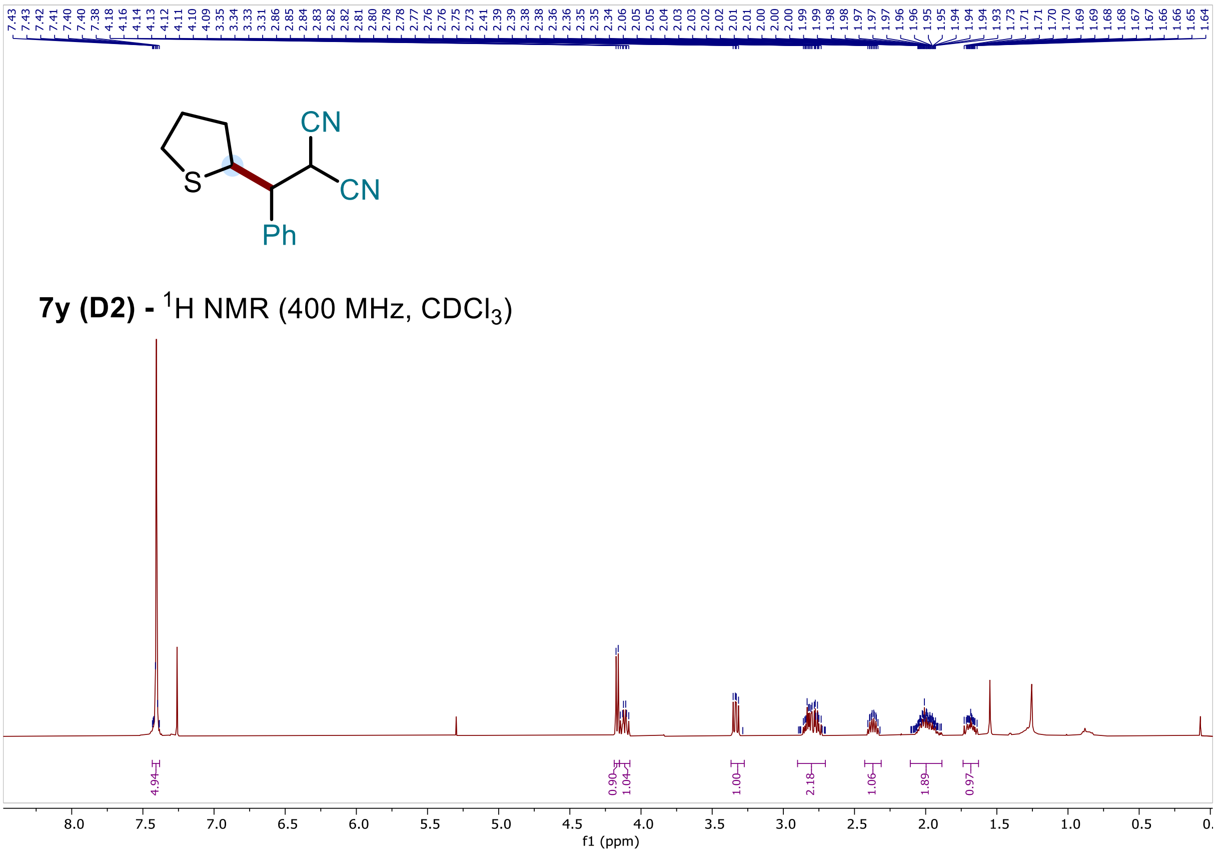
7y (D2) - ^{1}H NMR (400 MHz, $CDCl_3$)
CN
CN
S
Ph
4.94
0.90
1.04
1.00
2.18
1.06
1.89
0.97
f1 (ppm)

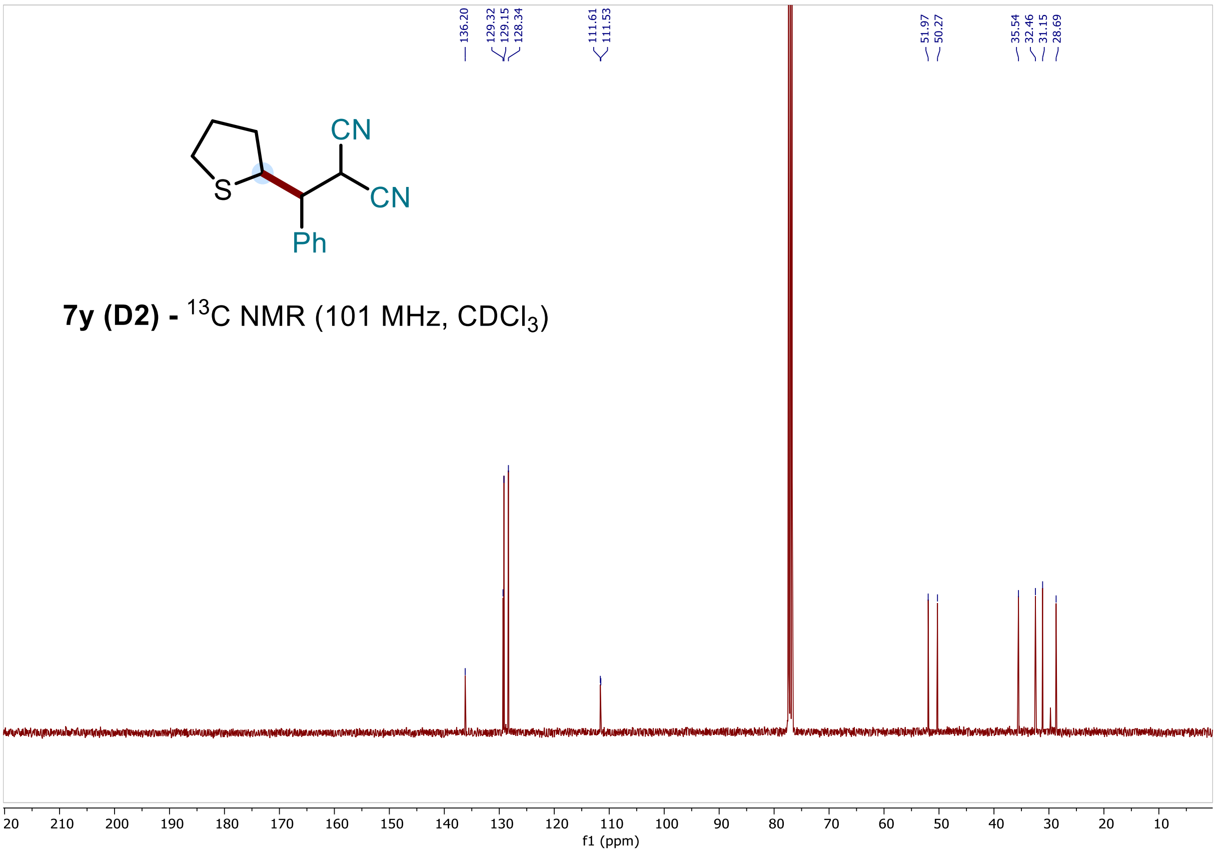
7y (D2) - ^{13}C NMR (101 MHz, $CDCl_3$)
CN
CN
S
Ph
136.20
129.32
129.15
128.34
111.61
111.53
51.97
50.27
35.54
32.46
31.15
28.69
f1 (ppm)

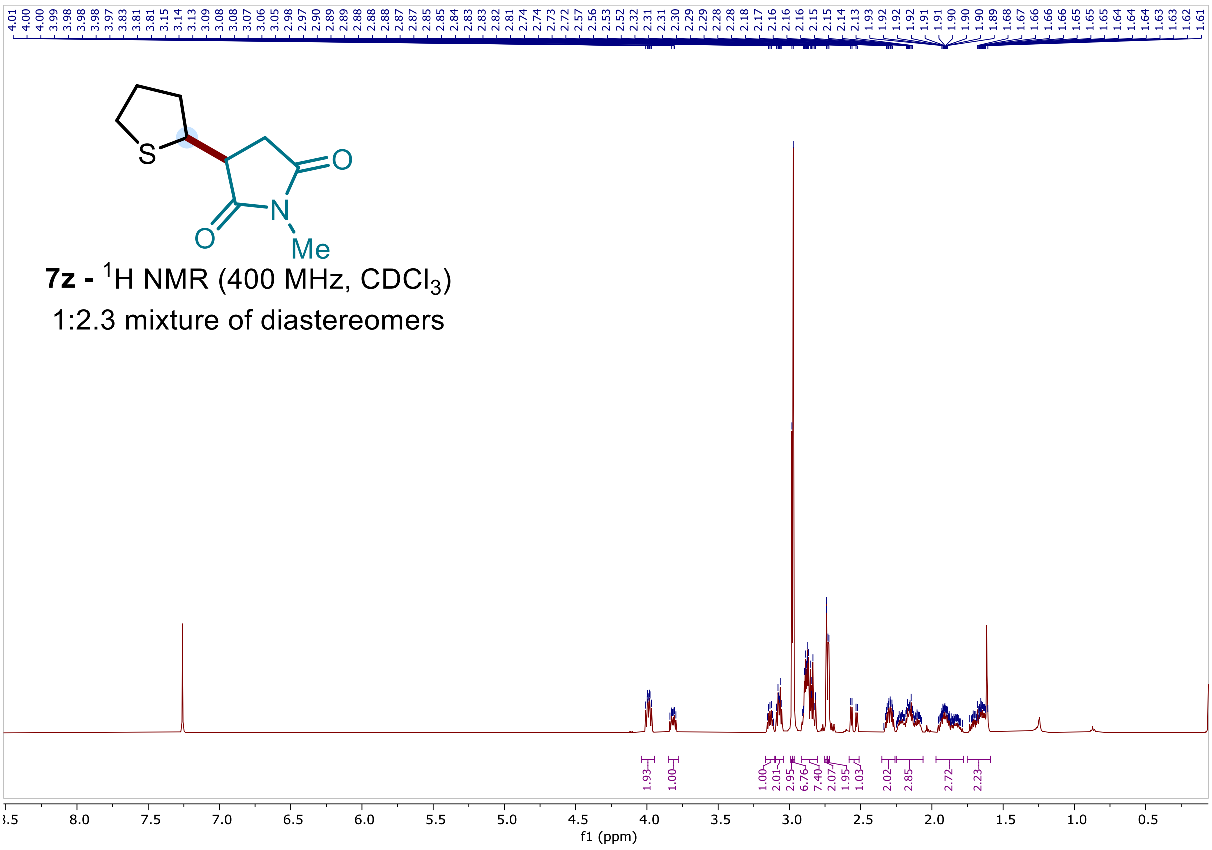
4.01 4.00 4.00 3.99 3.98 3.98 3.98 3.97 3.83 3.81 3.15 3.14 3.13 3.09 3.08 3.08 3.07 3.06 3.05 2.98 2.97 2.90 2.89 2.89 2.88 2.88 2.88 2.87 2.87 2.85 2.85 2.84 2.83 2.83 2.82 2.81 2.74 2.74 2.73 2.72 2.57 2.56 2.53 2.52 2.32 2.31 2.31 2.30 2.29 2.29 2.28 2.28 2.18 2.17 2.16 2.16 2.16 2.15 2.15 2.14 2.13 1.93 1.92 1.92 1.92 1.91 1.91 1.90 1.90 1.90 1.89 1.68 1.67 1.66 1.66 1.65 1.65 1.65 1.64 1.64 1.63 1.63 1.62 1.61
S
O
N
Me
O
7z - ¹H NMR (400 MHz, CDCl₃)
1:2.3 mixture of diastereomers
1.93 1.00 1.00 2.01 2.95 6.76 7.40 2.07 1.95 1.03 2.02 2.85 2.72 2.23
8.0 7.5 7.0 6.5 6.0 5.5 5.0 4.5 4.0 3.5 3.0 2.5 2.0 1.5 1.0 0.5
f1 (ppm)

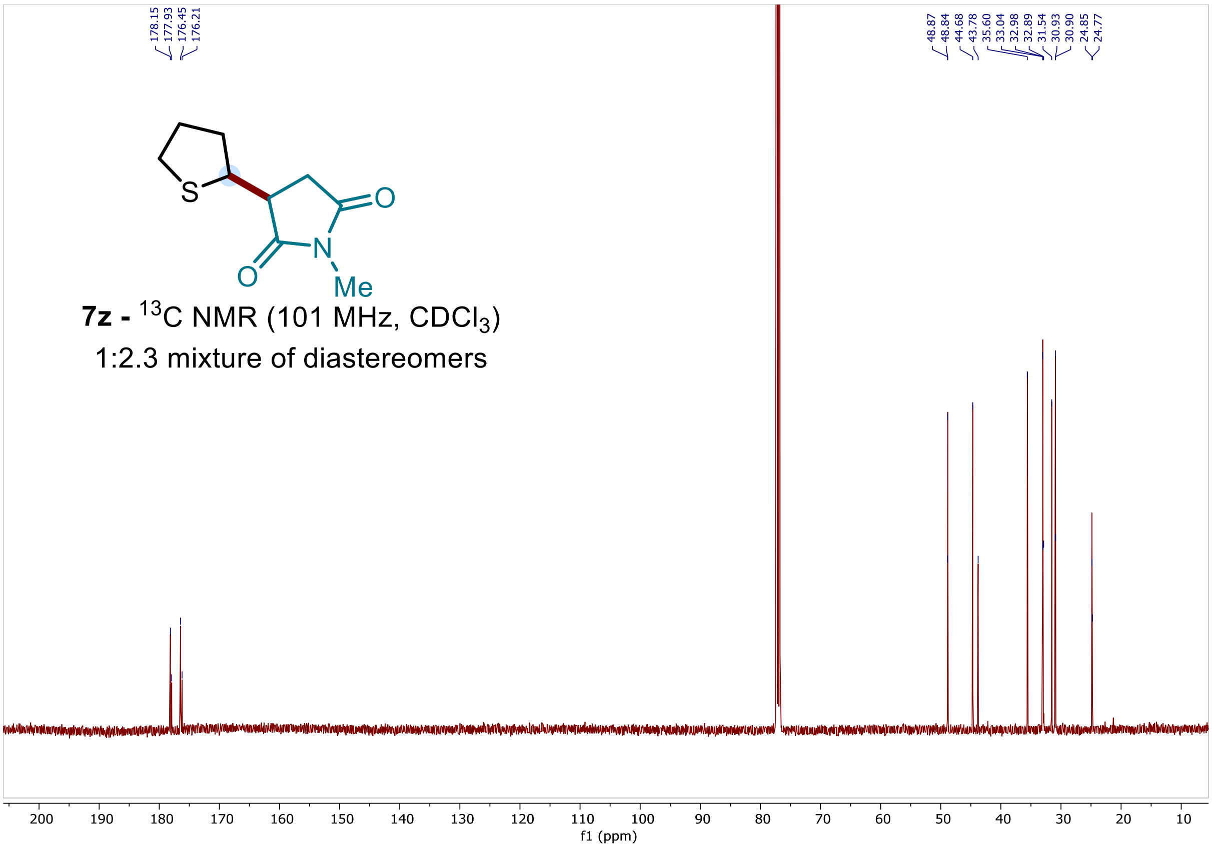
178.15 177.93 176.45 176.21
48.87 48.84 44.68 43.78 35.60 33.04 32.98 32.89 31.54 30.93 30.90 24.85 24.77
S
O
N
Me
O
7z - ¹³C NMR (101 MHz, CDCl₃)
1:2.3 mixture of diastereomers
200 190 180 170 160 150 140 130 120 110 100 90 80 70 60 50 40 30 20 10
f1 (ppm)

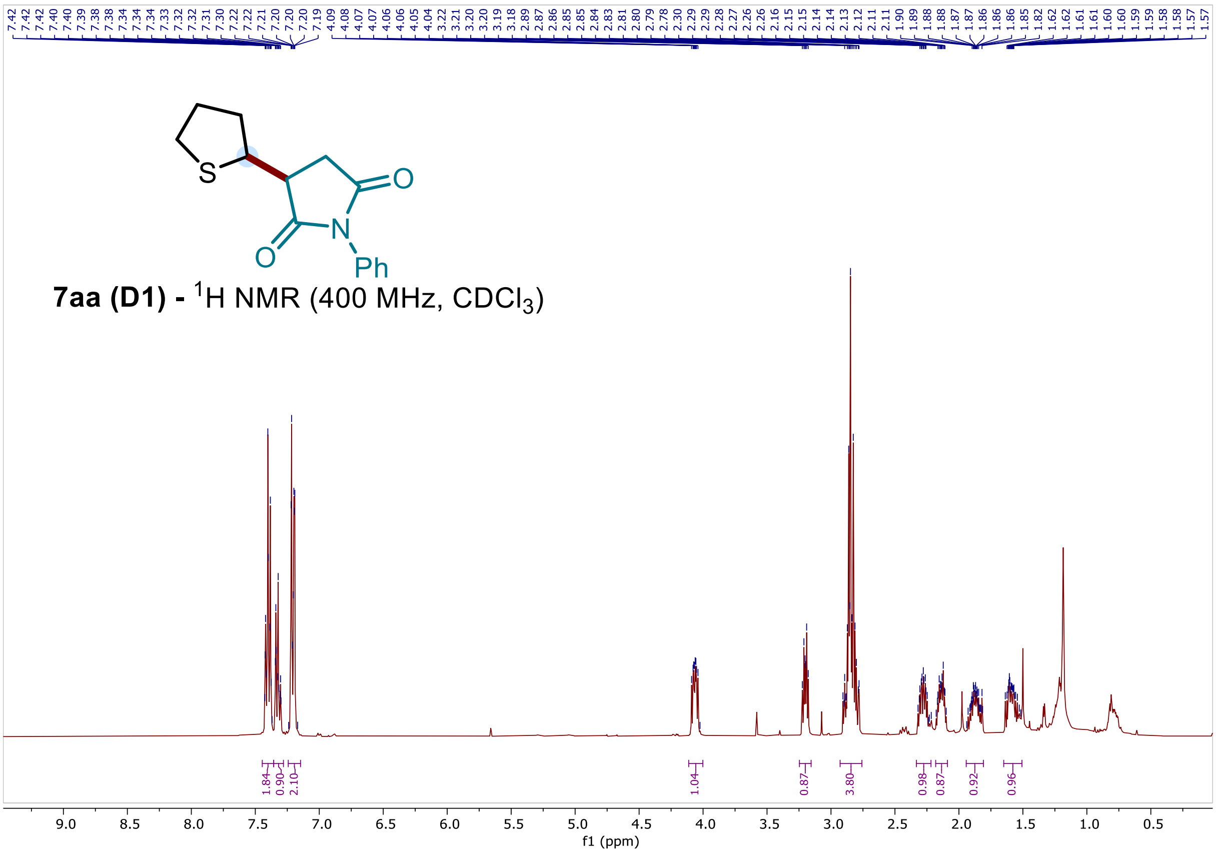
7aa (D1) - 1H NMR (400 MHz, CDCl3)
S
O
N
O
Ph
f1 (ppm)

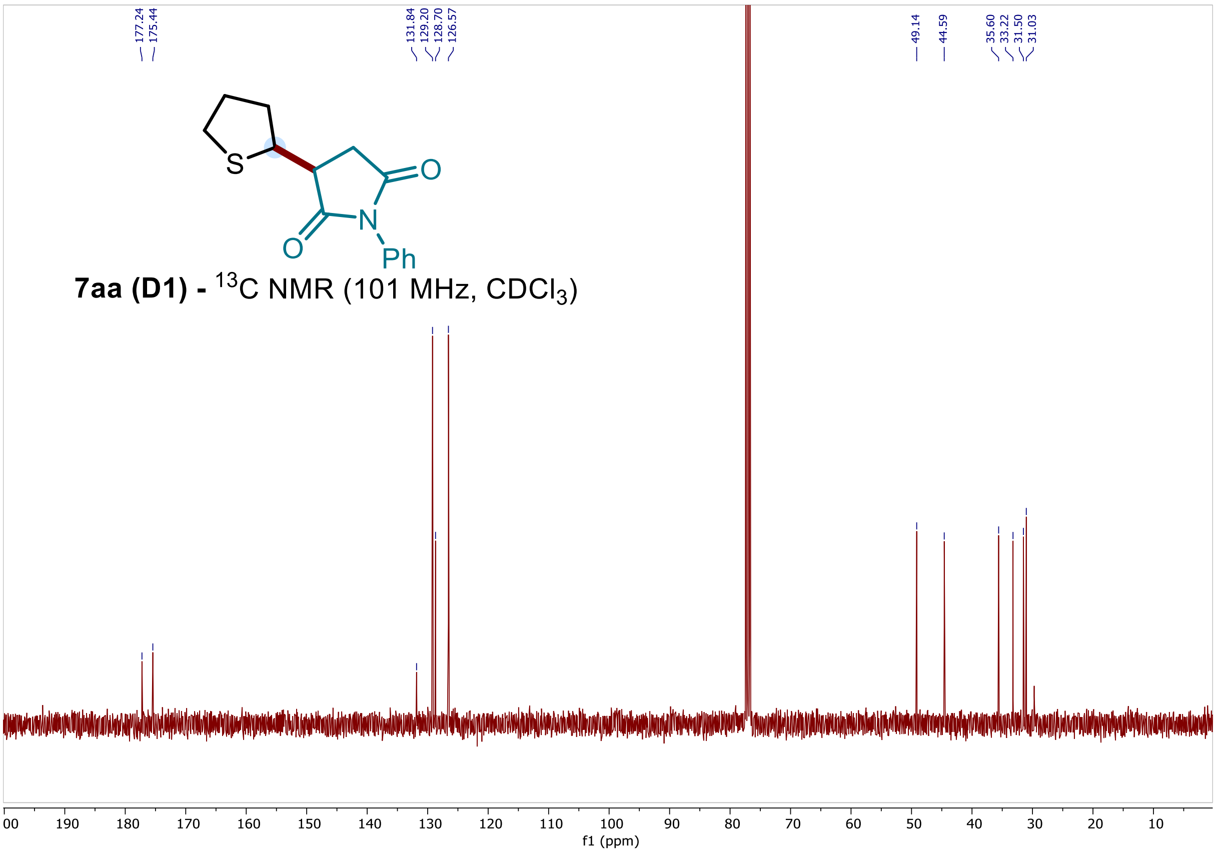
7aa (D1) - 13C NMR (101 MHz, CDCl3)
177.24
175.44
131.84
129.20
128.70
126.57
49.14
44.59
35.60
33.22
31.50
31.03
f1 (ppm)

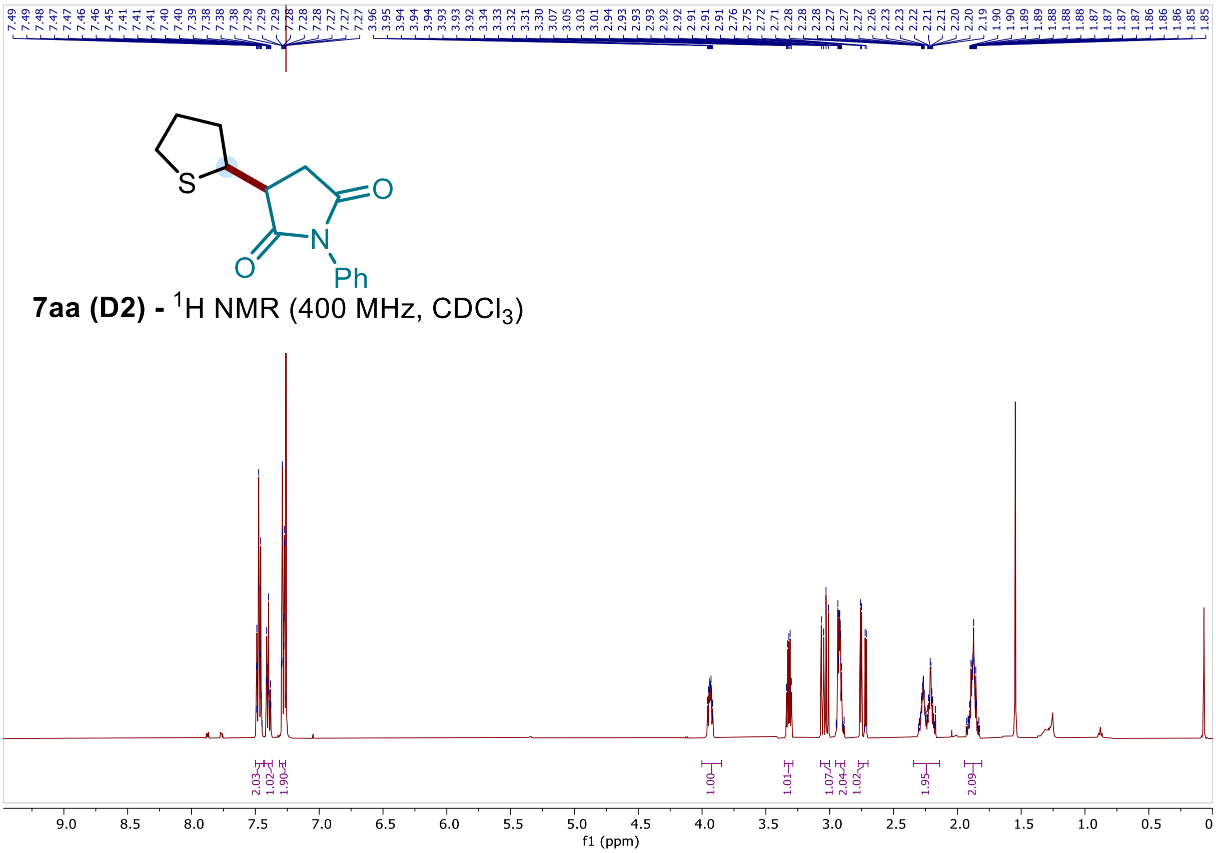
7aa (D2) - 1H NMR (400 MHz, CDCl3)
f1 (ppm)

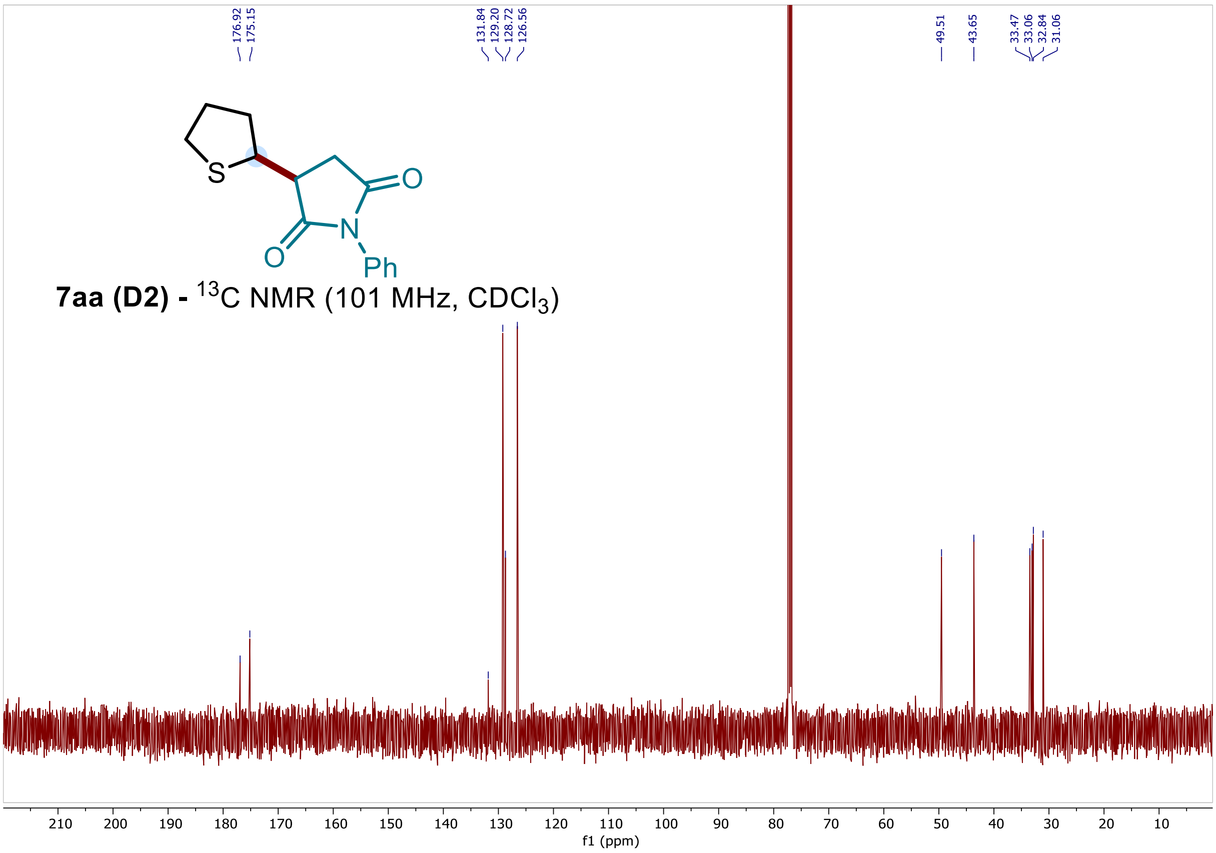
7aa (D2) - 13C NMR (101 MHz, CDCl3)
176.92
175.15
131.84
129.20
128.72
126.56
49.51
43.65
33.47
33.06
32.84
31.06
f1 (ppm)

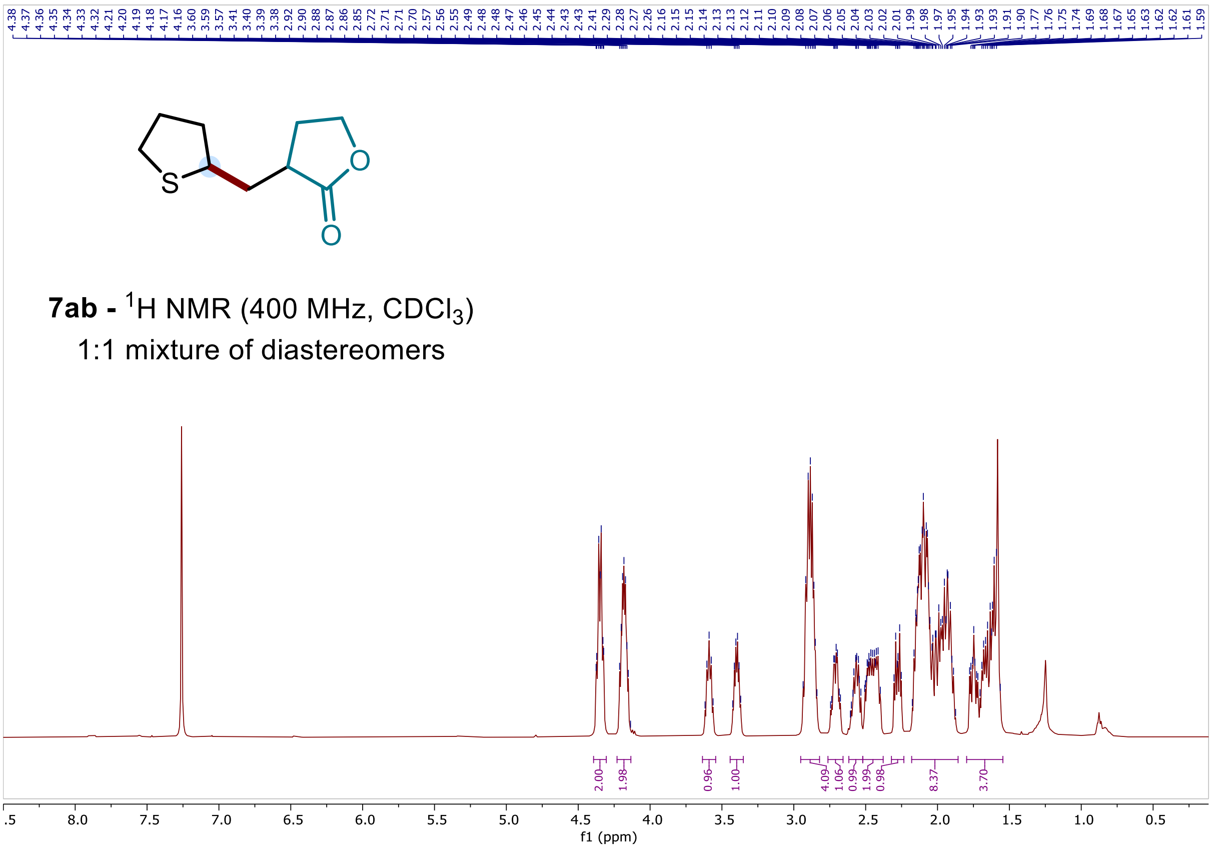

7ab - ^{1}H NMR (400 MHz, $CDCl_3$)
1:1 mixture of diastereomers
f1 (ppm)

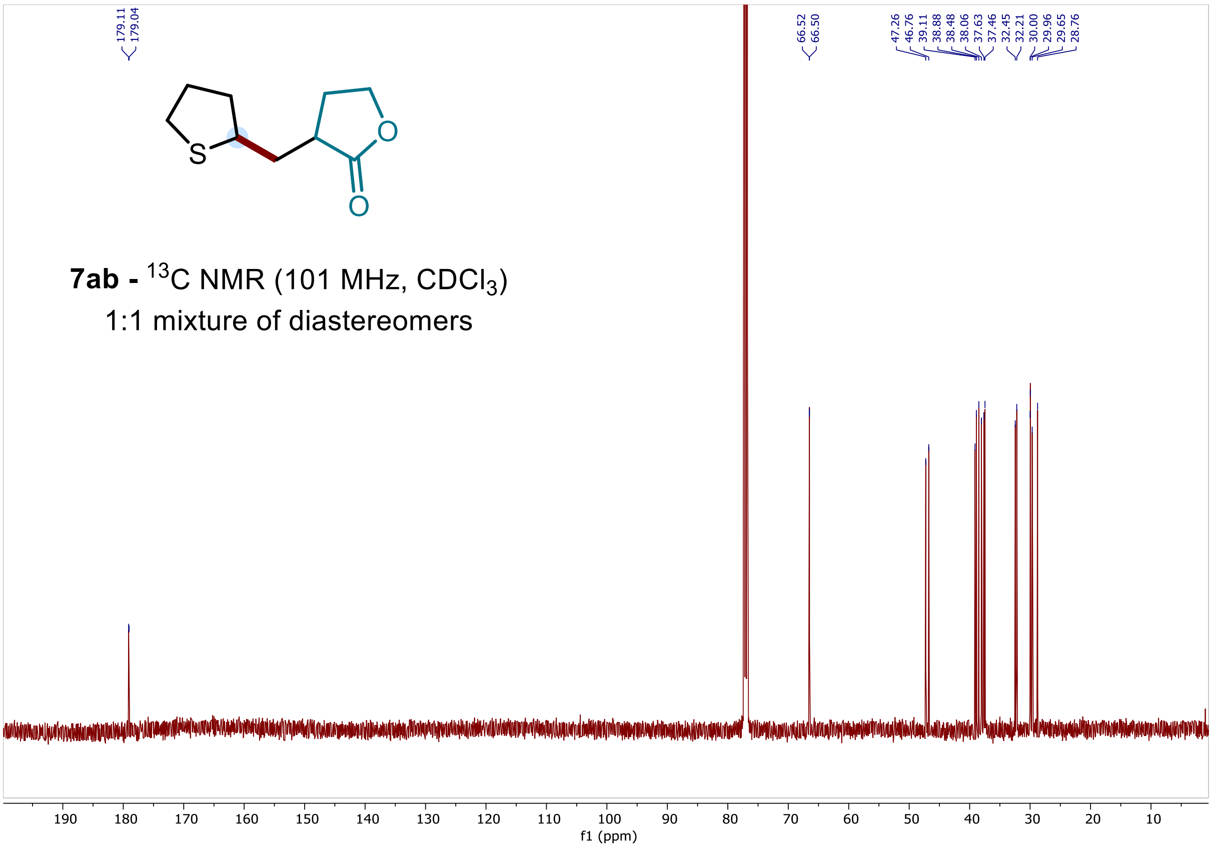

7ab - ^{13}C NMR (101 MHz, $CDCl_3$)
1:1 mixture of diastereomers
179.11
179.04
66.52
66.50
47.26
46.76
39.11
38.88
38.48
38.06
37.63
37.46
32.45
32.21
30.00
29.96
29.65
28.76
f1 (ppm)

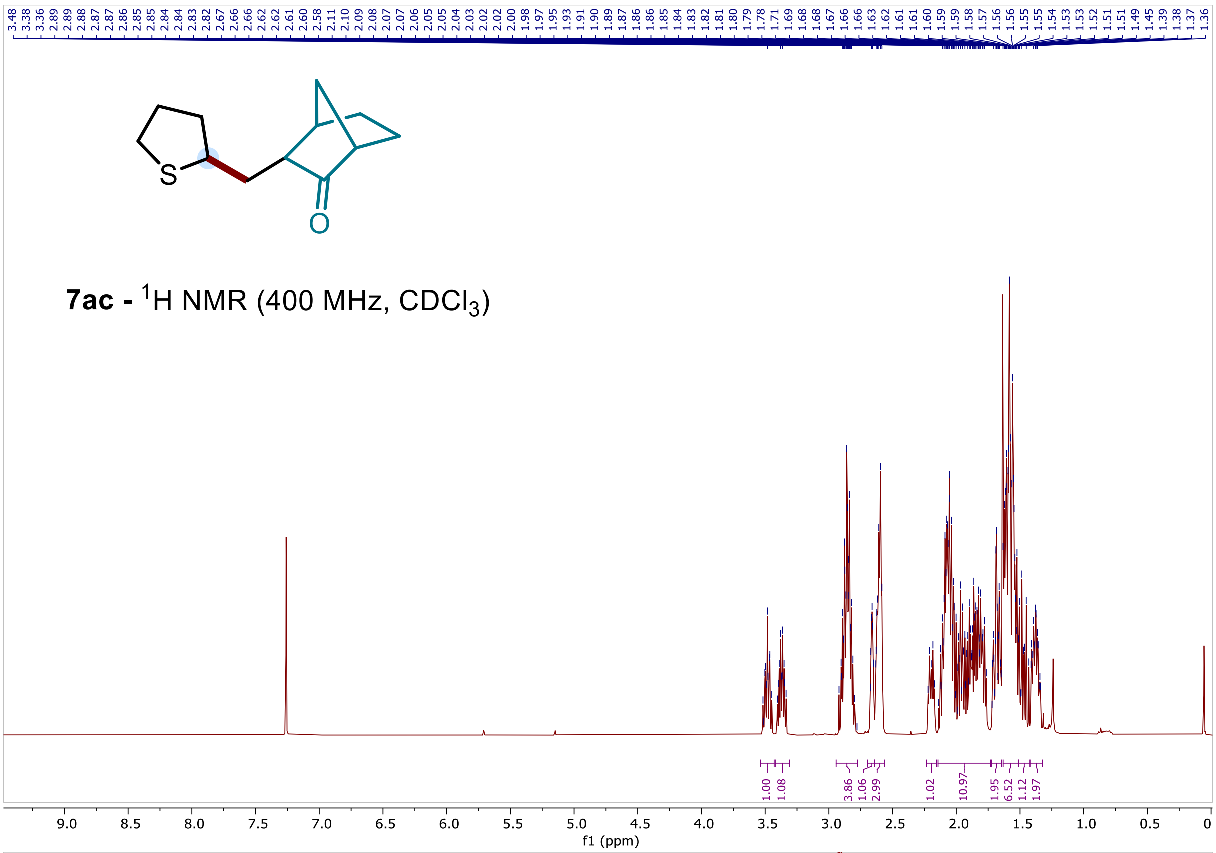
7ac - ^{1}H NMR (400 MHz, $CDCl_3$)
f1 (ppm)

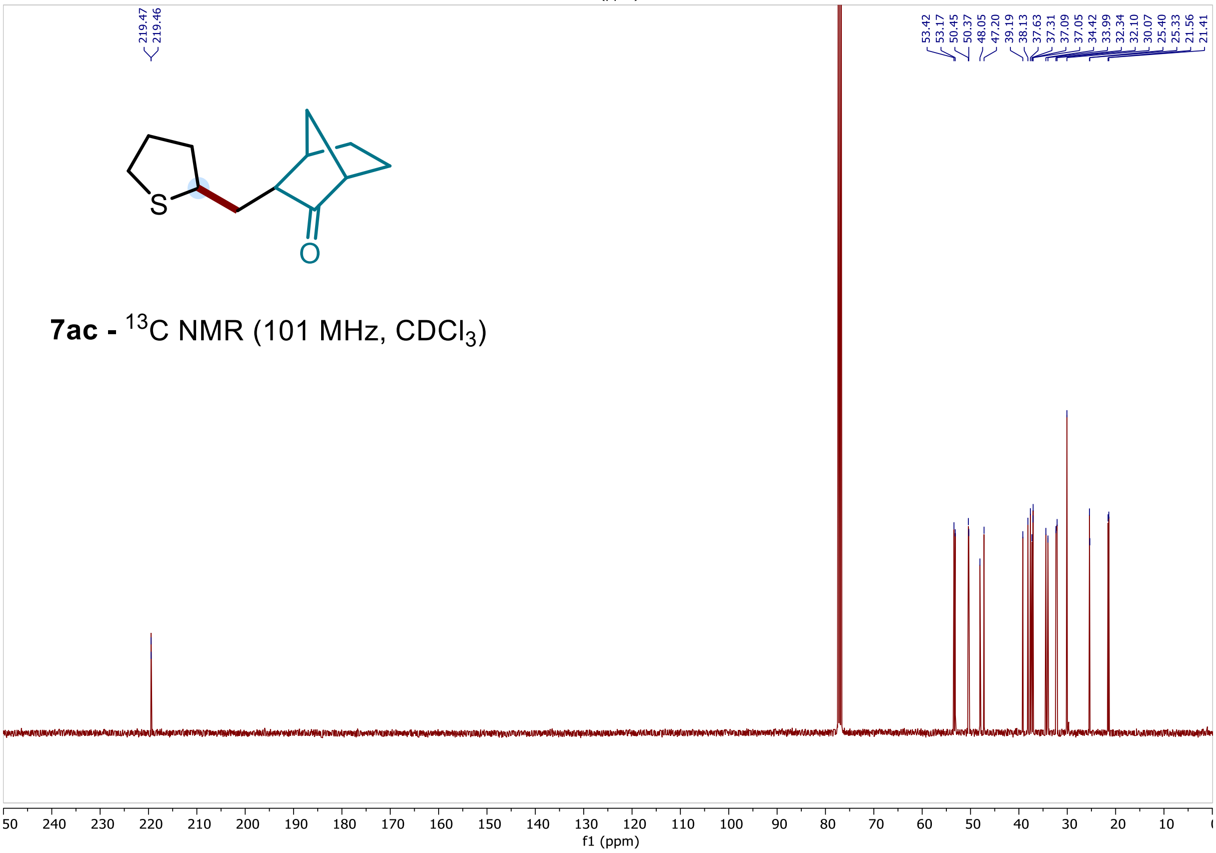
7ac - ^{13}C NMR (101 MHz, $CDCl_3$)
f1 (ppm)

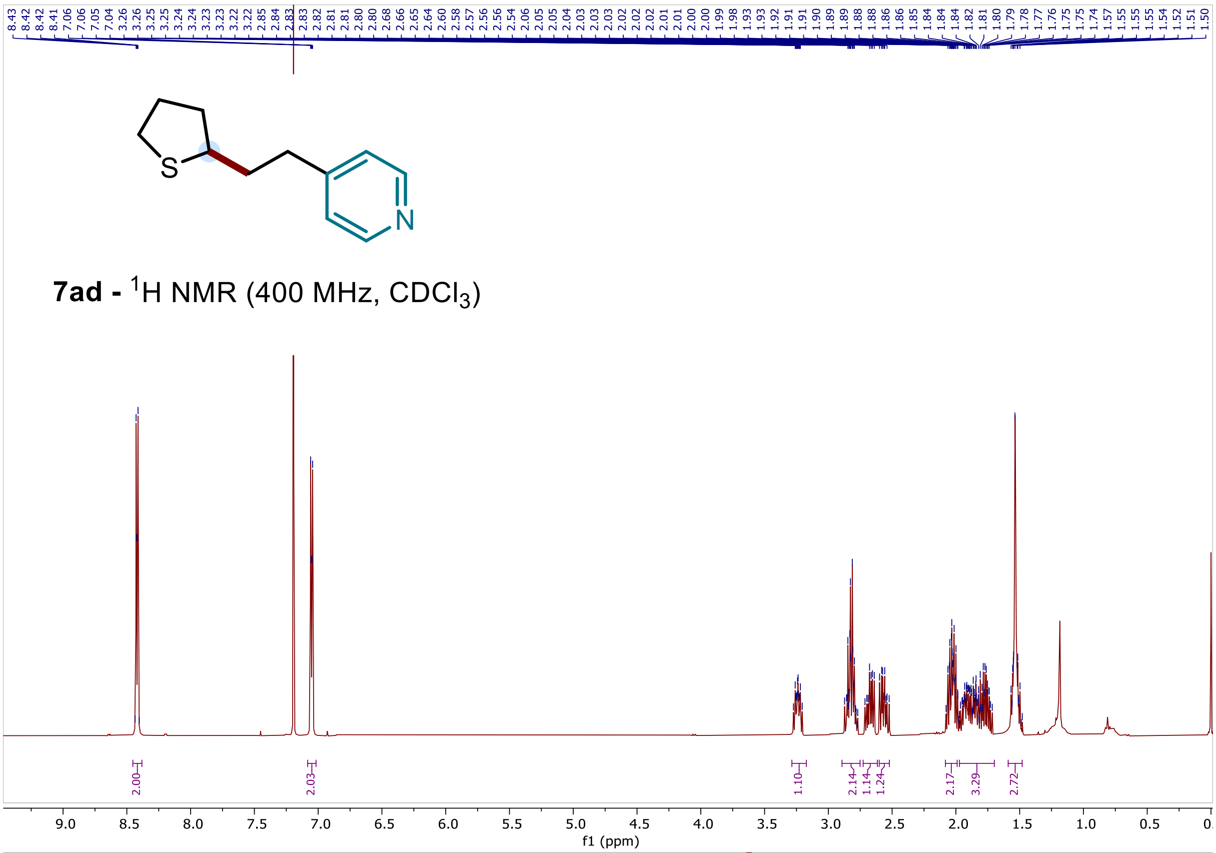
7ad - ^{1}H NMR (400 MHz, $CDCl_3$)
S
N
2.00
2.03
1.10
2.14
1.14
1.24
2.17
3.29
2.72
f1 (ppm)

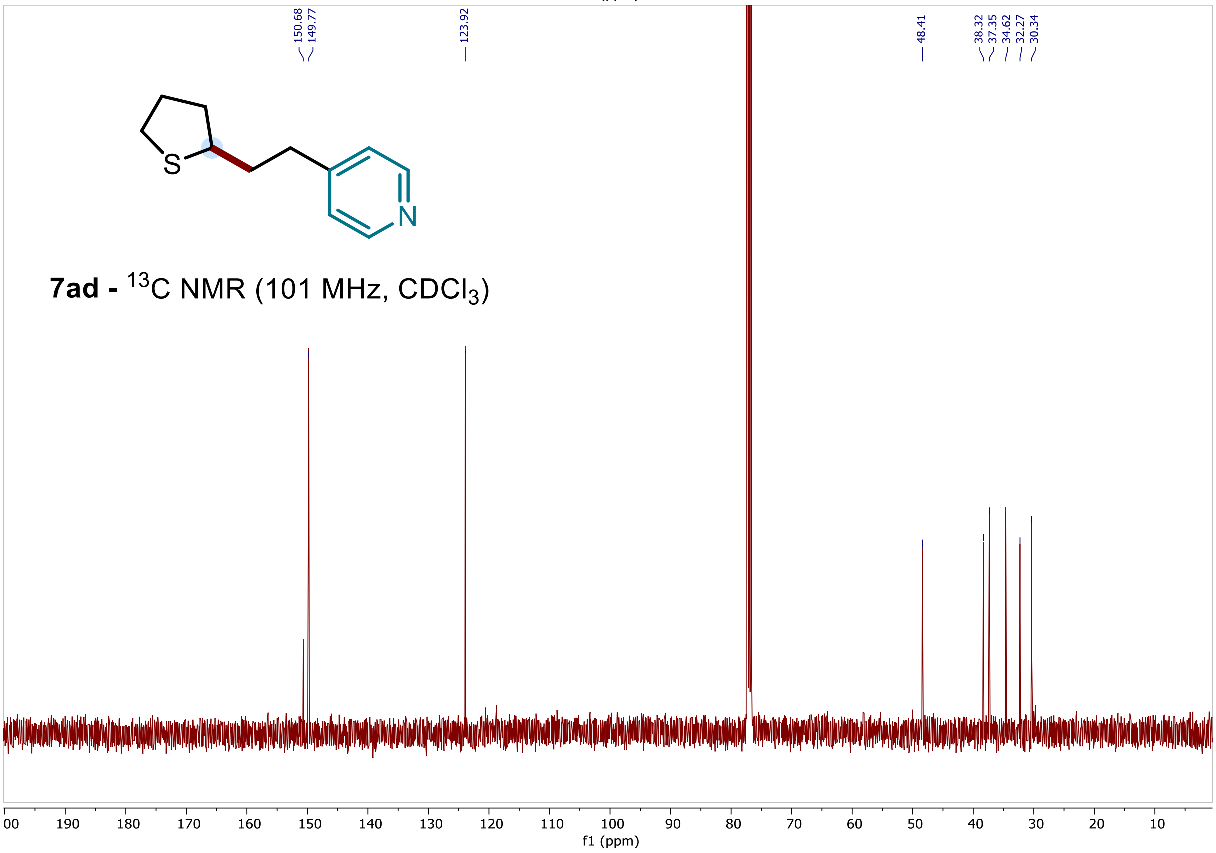
7ad - ^{13}C NMR (101 MHz, $CDCl_3$)
S
N
150.68
149.77
123.92
48.41
38.32
37.35
34.62
32.27
30.34
f1 (ppm)

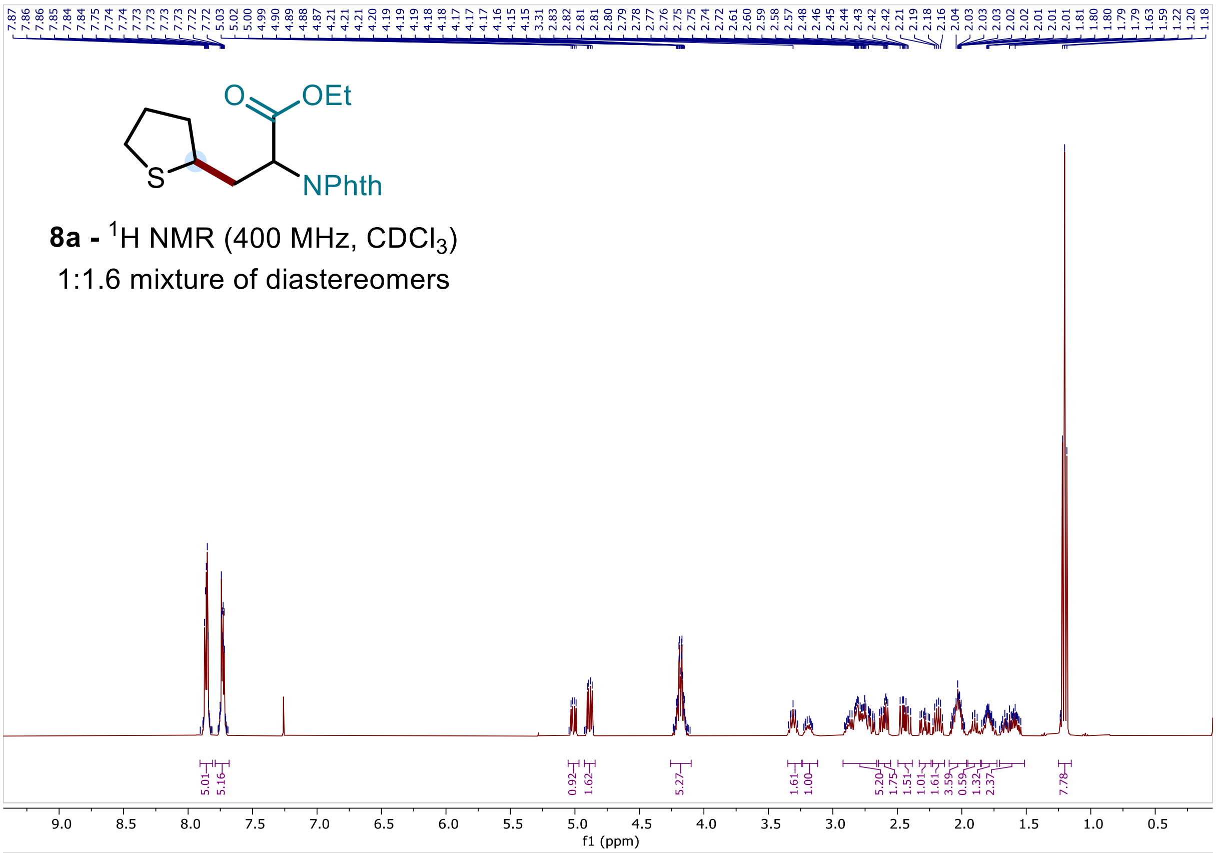
7.87 7.86 7.85 7.84 7.84 7.75 7.74 7.74 7.73 7.73 7.73 7.73 7.72 7.72 5.03 5.02 5.00 4.99 4.90 4.89 4.88 4.87 4.21 4.21 4.21 4.20 4.19 4.19 4.19 4.18 4.18 4.17 4.17 4.17 4.16 4.15 4.15 3.31 2.83 2.82 2.81 2.81 2.80 2.79 2.78 2.77 2.76 2.75 2.75 2.74 2.72 2.61 2.60 2.59 2.58 2.57 2.48 2.46 2.45 2.44 2.43 2.42 2.42 2.21 2.19 2.18 2.16 2.04 2.03 2.03 2.03 2.02 2.02 2.01 2.01 1.81 1.80 1.80 1.79 1.63 1.59 1.22 1.20 1.18
O
OEt
S
NPhth
8a - 1H NMR (400 MHz, CDCl3)
1:1.6 mixture of diastereomers
5.01 5.16 0.92 1.62 5.27 1.61 1.00 5.20 1.75 1.51 1.01 1.61 3.59 0.59 1.32 2.37 7.78
9.0 8.5 8.0 7.5 7.0 6.5 6.0 5.5 5.0 4.5 4.0 3.5 3.0 2.5 2.0 1.5 1.0 0.5
f1 (ppm)

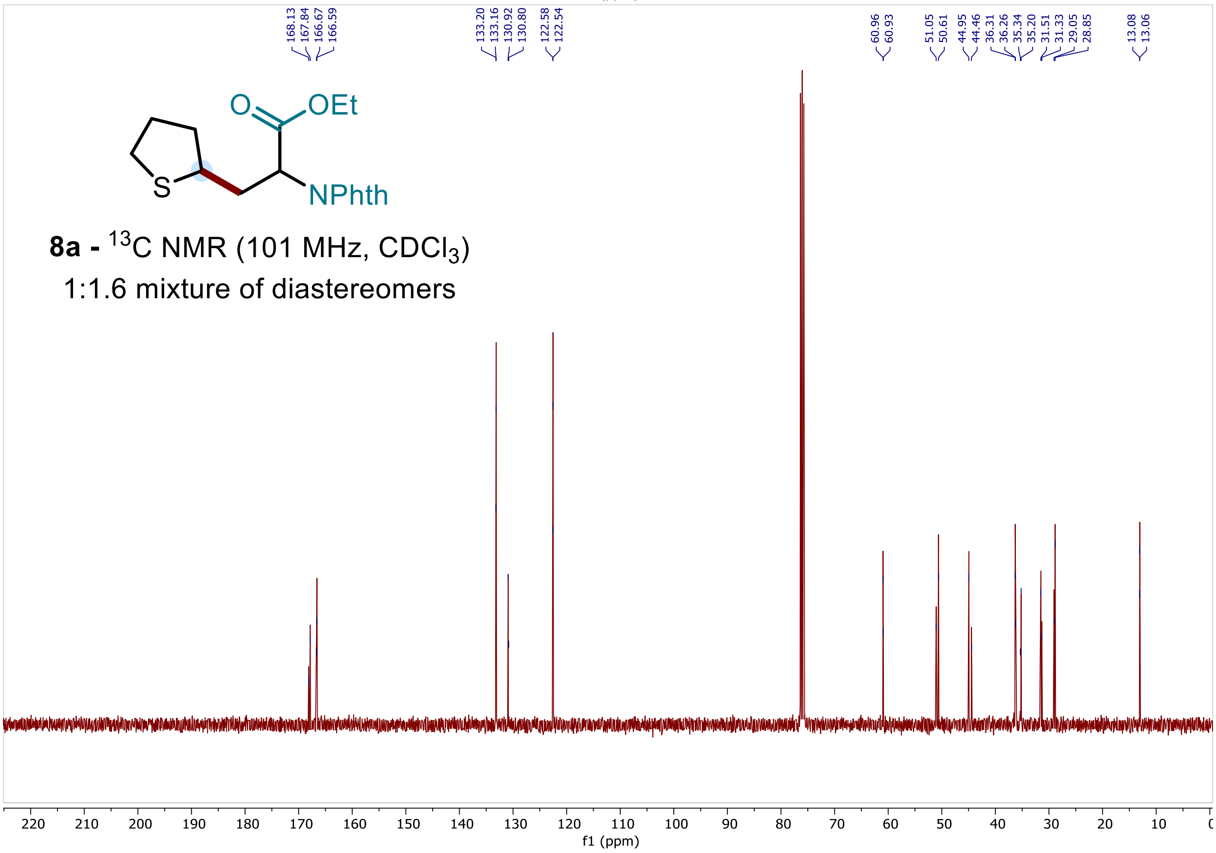
168.13 167.84 166.67 166.59 133.20 133.16 130.92 130.80 122.58 122.54 60.96 60.93 51.05 50.61 44.95 44.46 36.31 36.26 35.34 35.20 31.51 31.33 29.05 28.85 13.08 13.06
O
OEt
S
NPhth
8a - 13C NMR (101 MHz, CDCl3)
1:1.6 mixture of diastereomers
220 210 200 190 180 170 160 150 140 130 120 110 100 90 80 70 60 50 40 30 20 10 0
f1 (ppm)

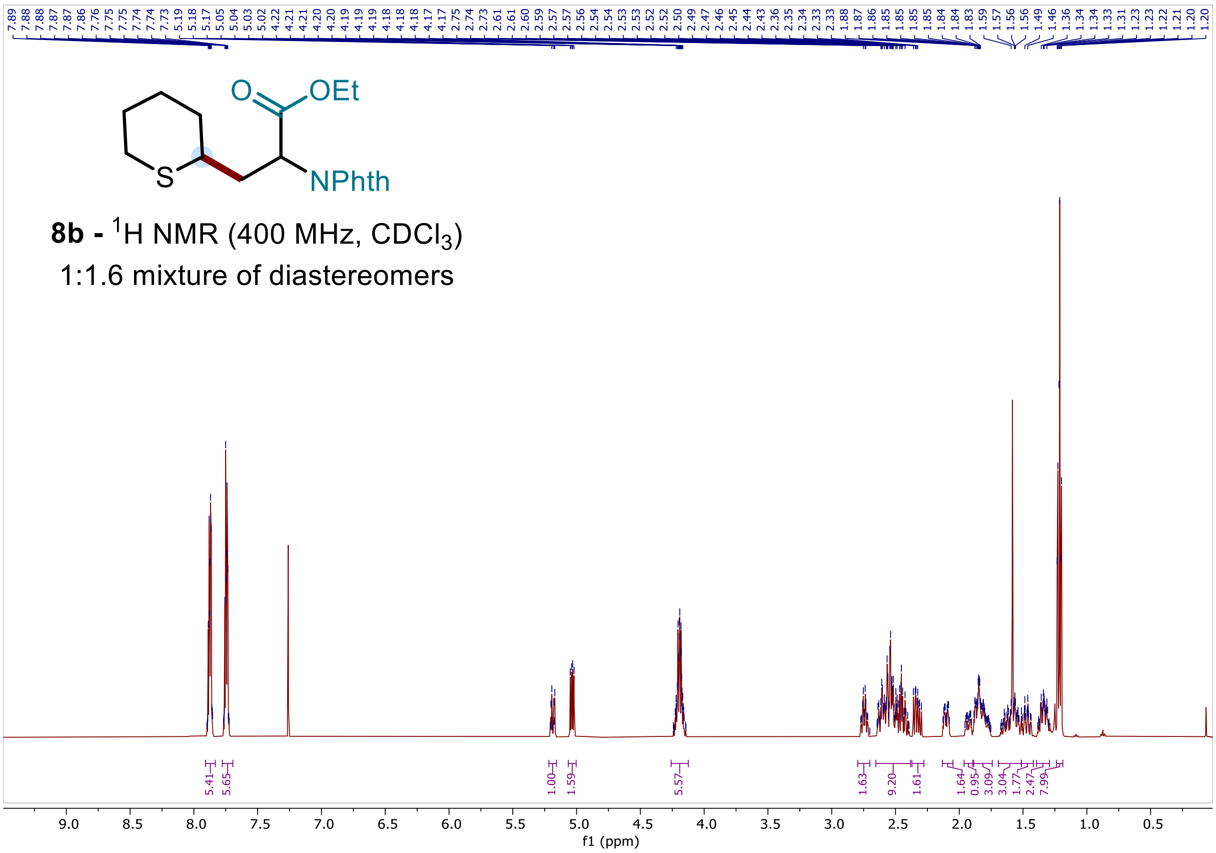

7.89 7.88 7.88 7.87 7.87 7.86 7.76 7.75 7.75 7.74 7.74 7.73 5.19 5.18 5.17 5.05 5.04 5.03 5.02 4.22 4.21 4.21 4.20 4.20 4.19 4.19 4.18 4.18 4.17 4.17 2.75 2.74 2.73 2.61 2.61 2.60 2.59 2.57 2.57 2.56 2.54 2.54 2.53 2.53 2.52 2.52 2.50 2.49 2.47 2.46 2.45 2.44 2.43 2.36 2.35 2.34 2.33 2.33 1.88 1.87 1.86 1.85 1.85 1.85 1.84 1.84 1.83 1.59 1.57 1.56 1.56 1.49 1.46 1.36 1.34 1.34 1.33 1.31 1.23 1.23 1.22 1.21 1.20 1.20
O
OEt
S
NPhth
8b - ^{1}H NMR (400 MHz, $CDCl_3$)
1:1.6 mixture of diastereomers
5.41 5.65 1.00 1.59 5.57 1.63 9.20 1.61 1.64 0.95 3.09 3.04 1.77 2.47 7.99
9.0 8.5 8.0 7.5 7.0 6.5 6.0 5.5 5.0 4.5 4.0 3.5 3.0 2.5 2.0 1.5 1.0 0.5
f1 (ppm)

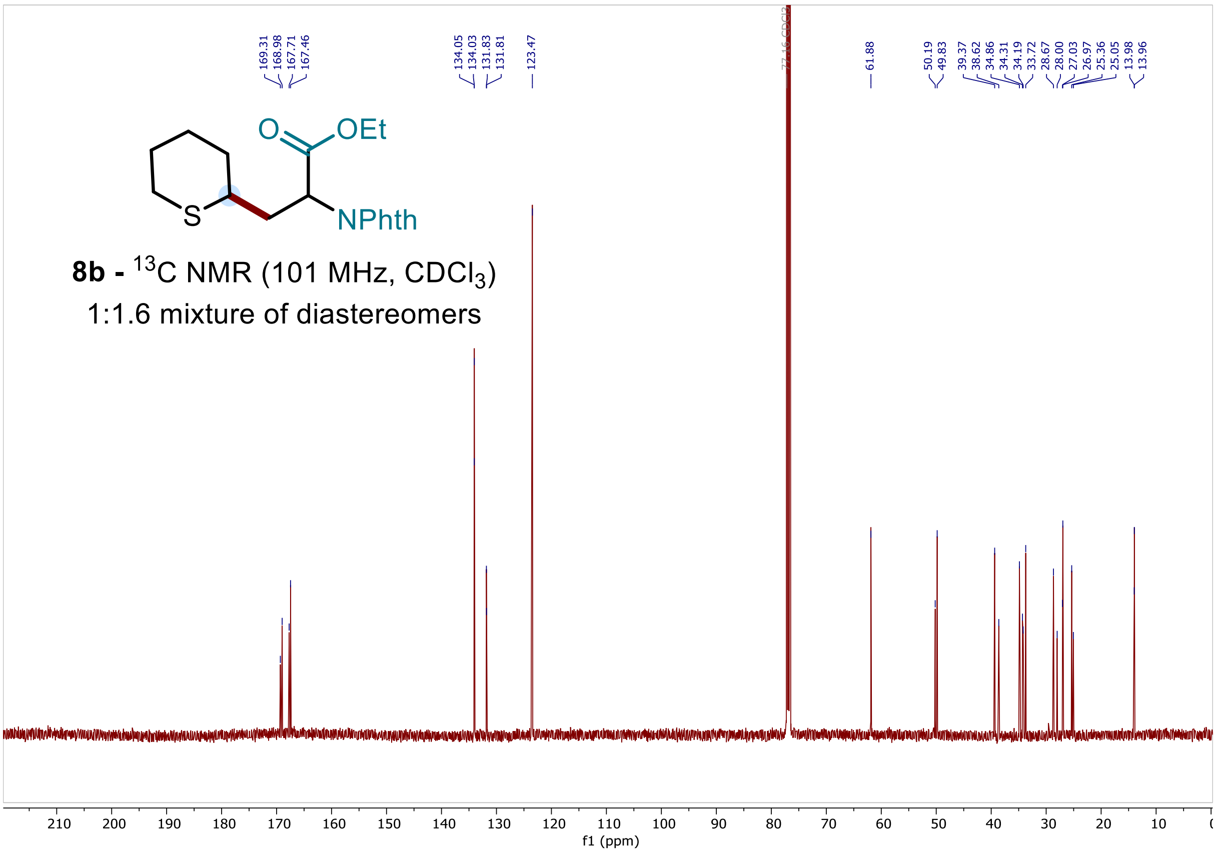

169.31 168.98 167.71 167.46
134.05 134.03 131.83 131.81
123.47
61.88
50.19 49.83 39.37 38.62 34.86 34.31 34.19 33.72 28.67 28.00 27.03 26.97 25.36 25.05 13.98 13.96
O
OEt
S
NPhth
8b - ^{13}C NMR (101 MHz, $CDCl_3$)
1:1.6 mixture of diastereomers
210 200 190 180 170 160 150 140 130 120 110 100 90 80 70 60 50 40 30 20 10
f1 (ppm)

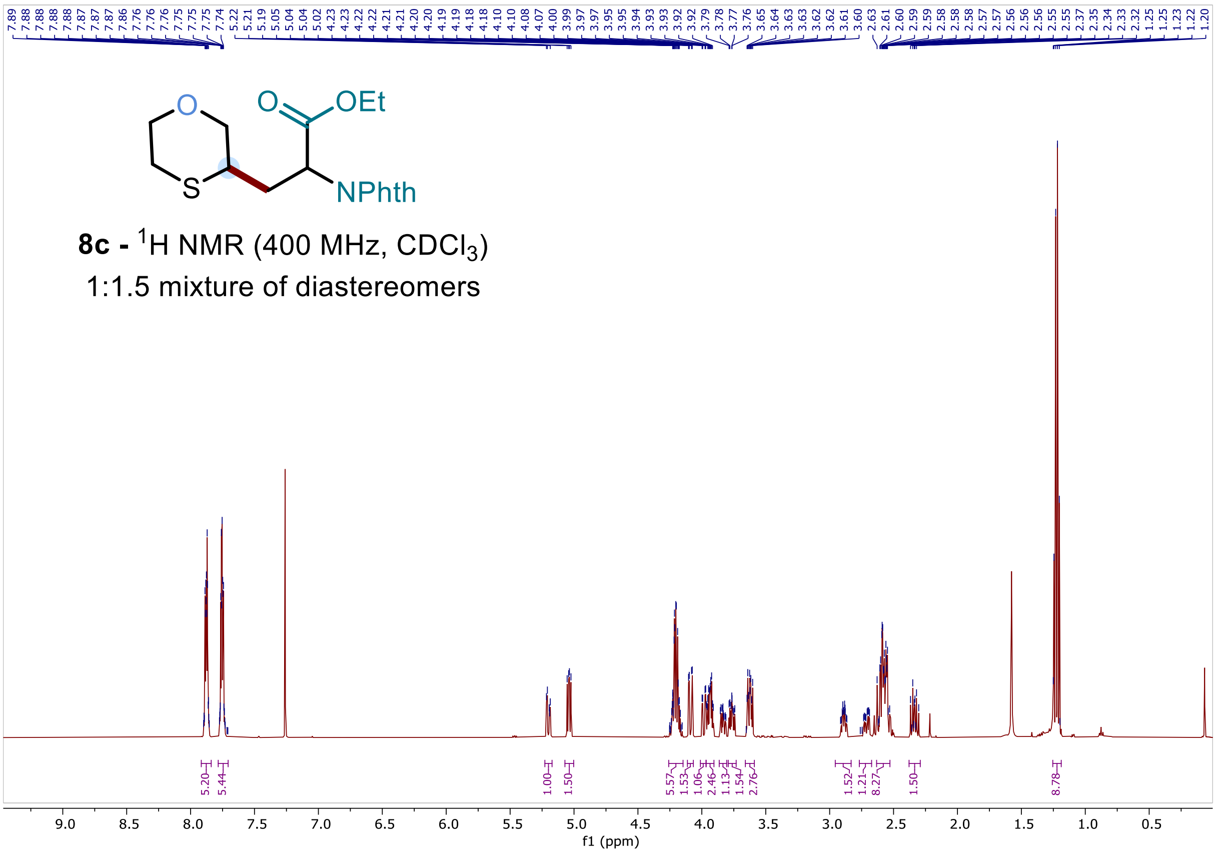
O
OEt
S
NPhth
8c - 1H NMR (400 MHz, CDCl3)
1:1.5 mixture of diastereomers
f1 (ppm)

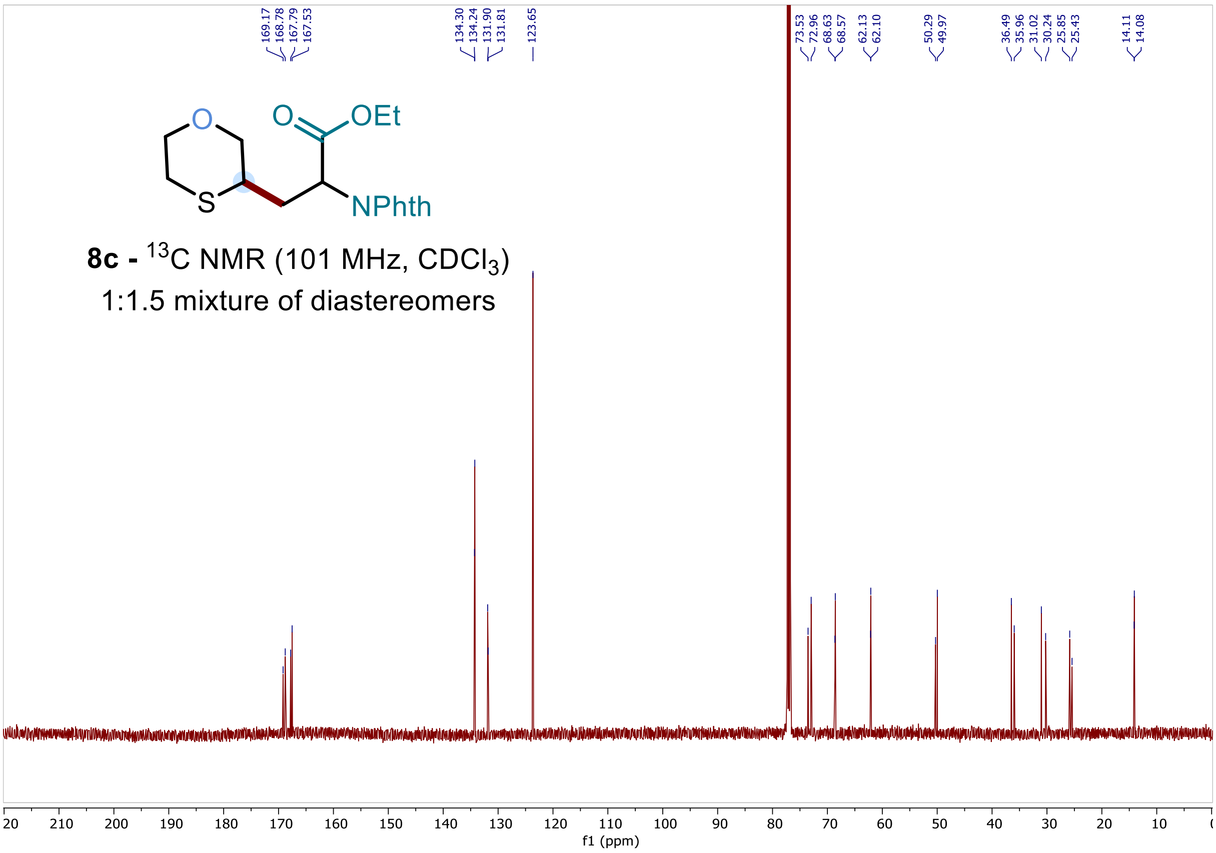
169.17
168.78
167.79
167.53
134.30
134.24
131.90
131.81
123.65
73.53
72.96
68.63
68.57
62.13
62.10
50.29
49.97
36.49
35.96
31.02
30.24
25.85
25.43
14.11
14.08
O
OEt
S
NPhth
8c - 13C NMR (101 MHz, CDCl3)
1:1.5 mixture of diastereomers
f1 (ppm)

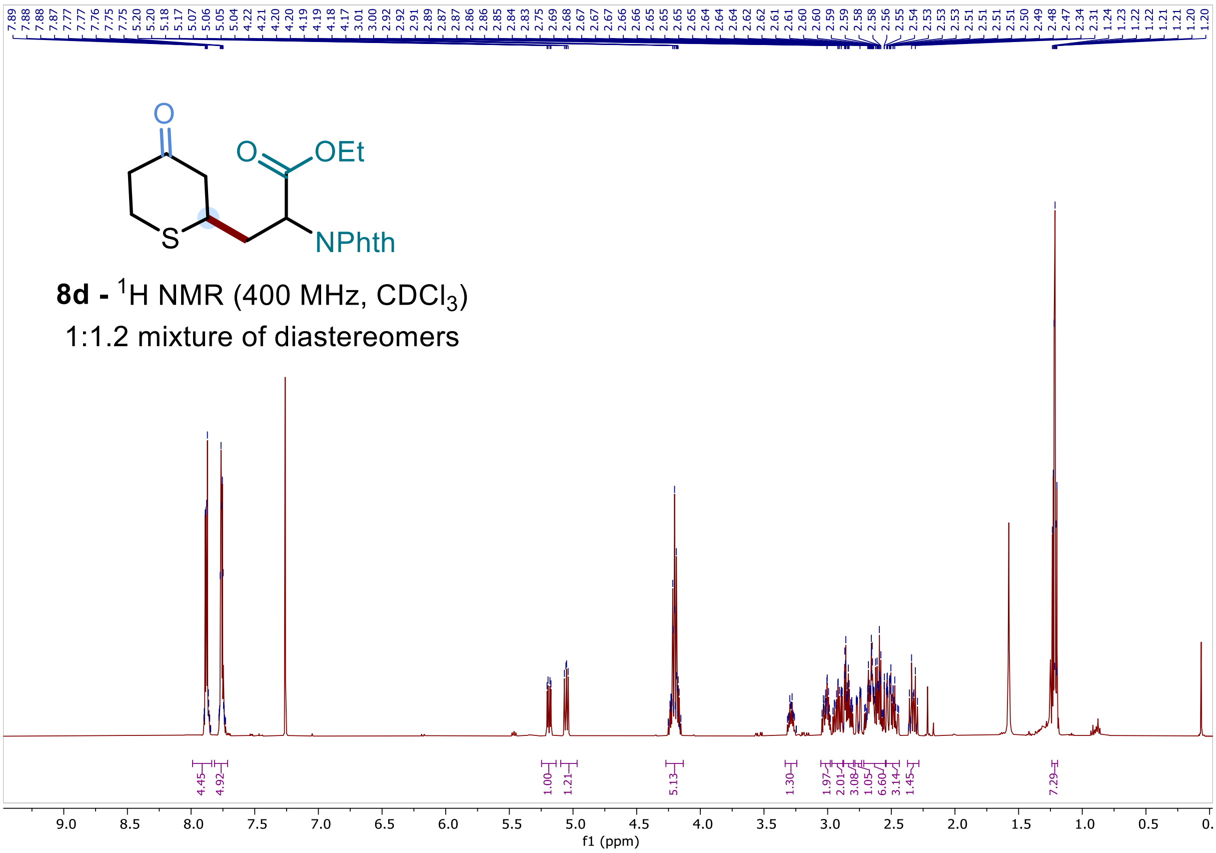

O
OEt
S
NPhth
8d - ¹H NMR (400 MHz, CDCl₃)
1:1.2 mixture of diastereomers
f1 (ppm)

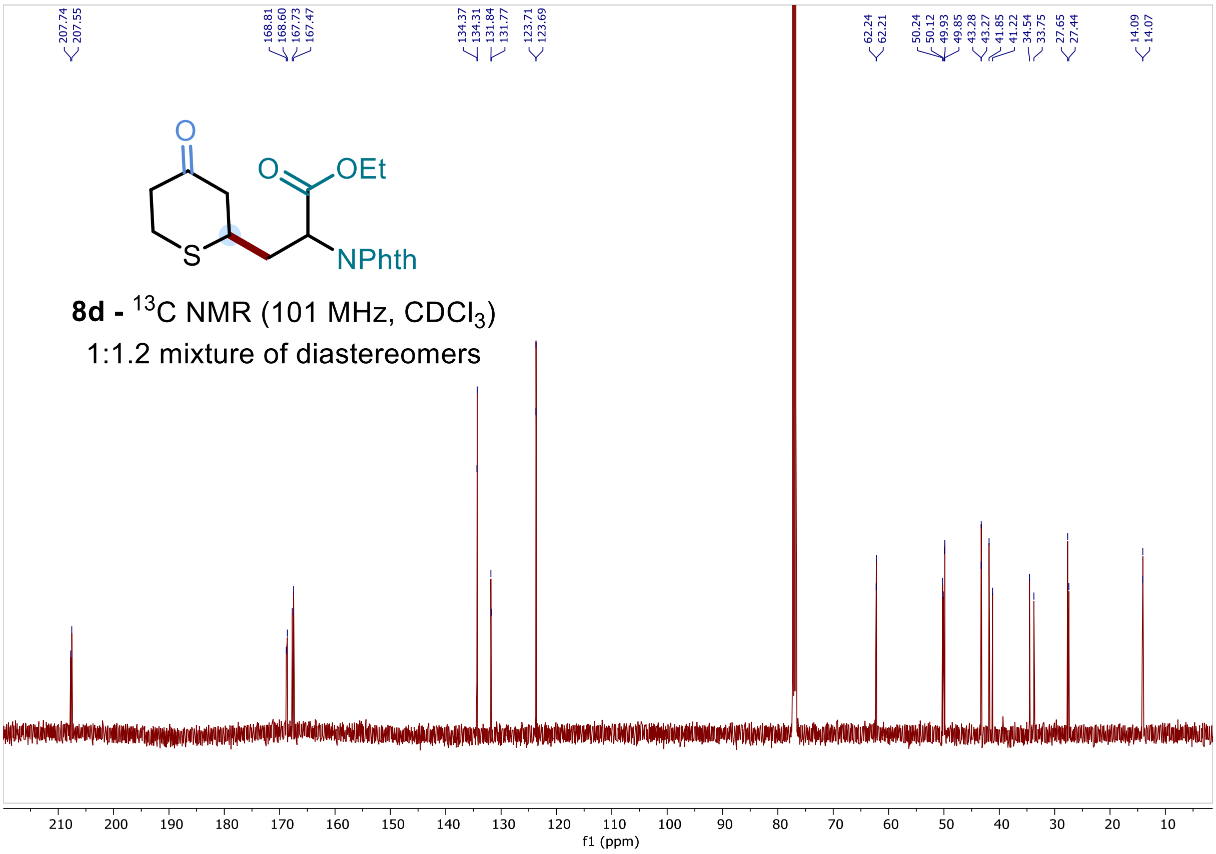

O
OEt
S
NPhth
8d - ¹³C NMR (101 MHz, CDCl₃)
1:1.2 mixture of diastereomers
f1 (ppm)

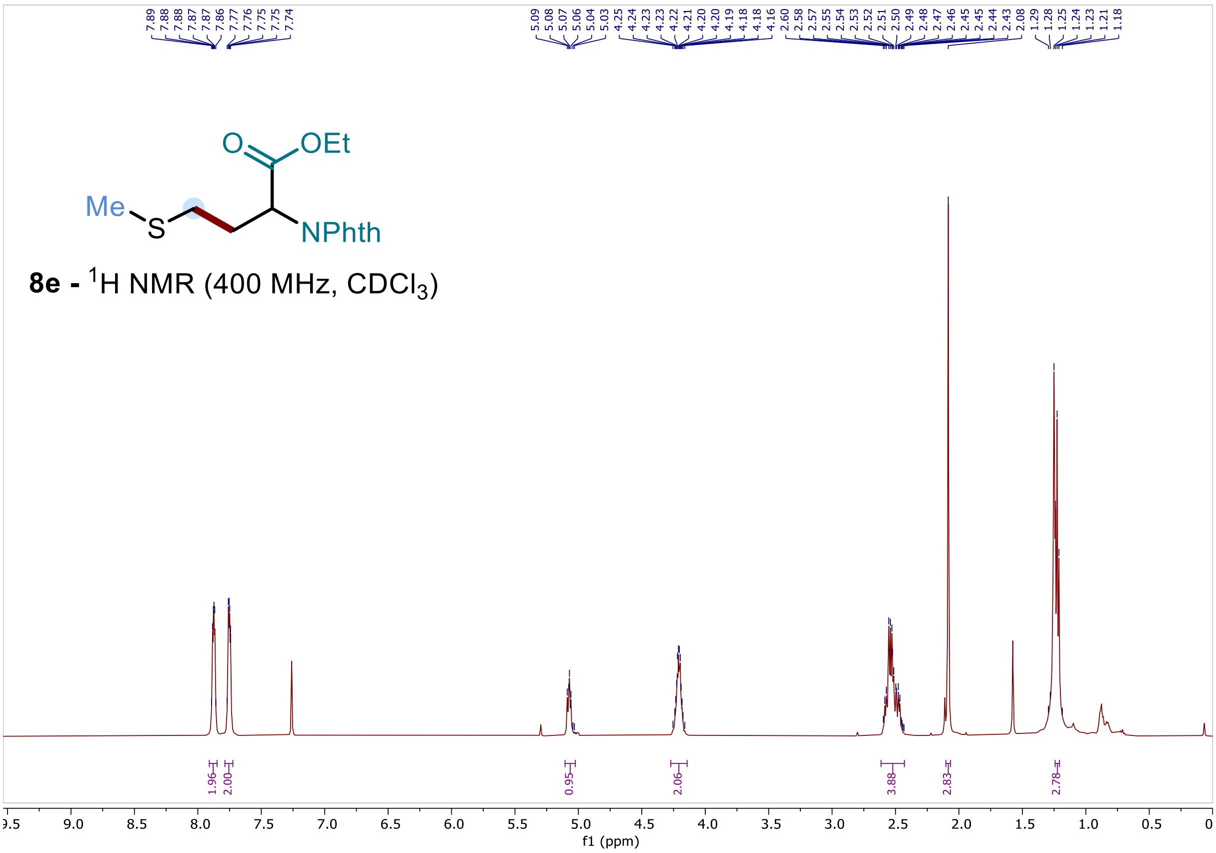

8e - ^{1}H NMR (400 MHz, $CDCl_3$)

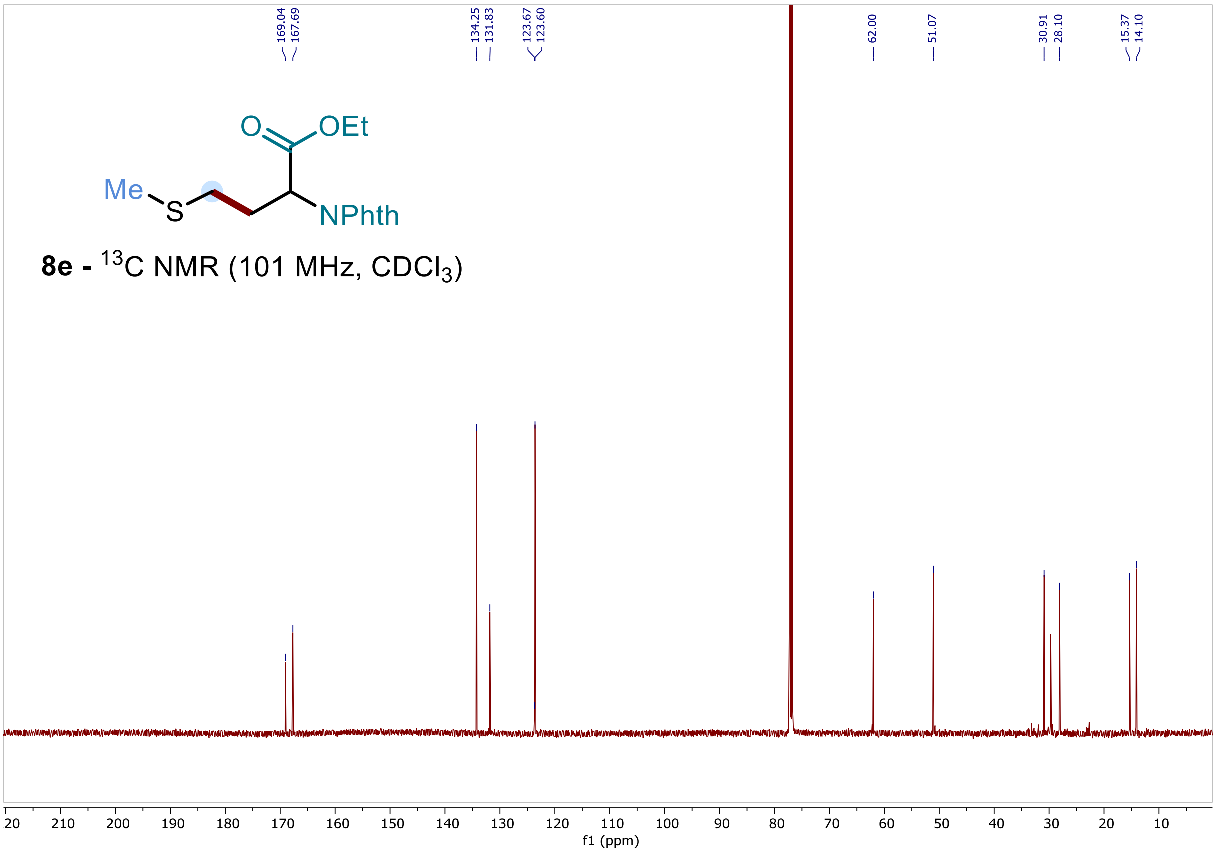

8e - ^{13}C NMR (101 MHz, $CDCl_3$)

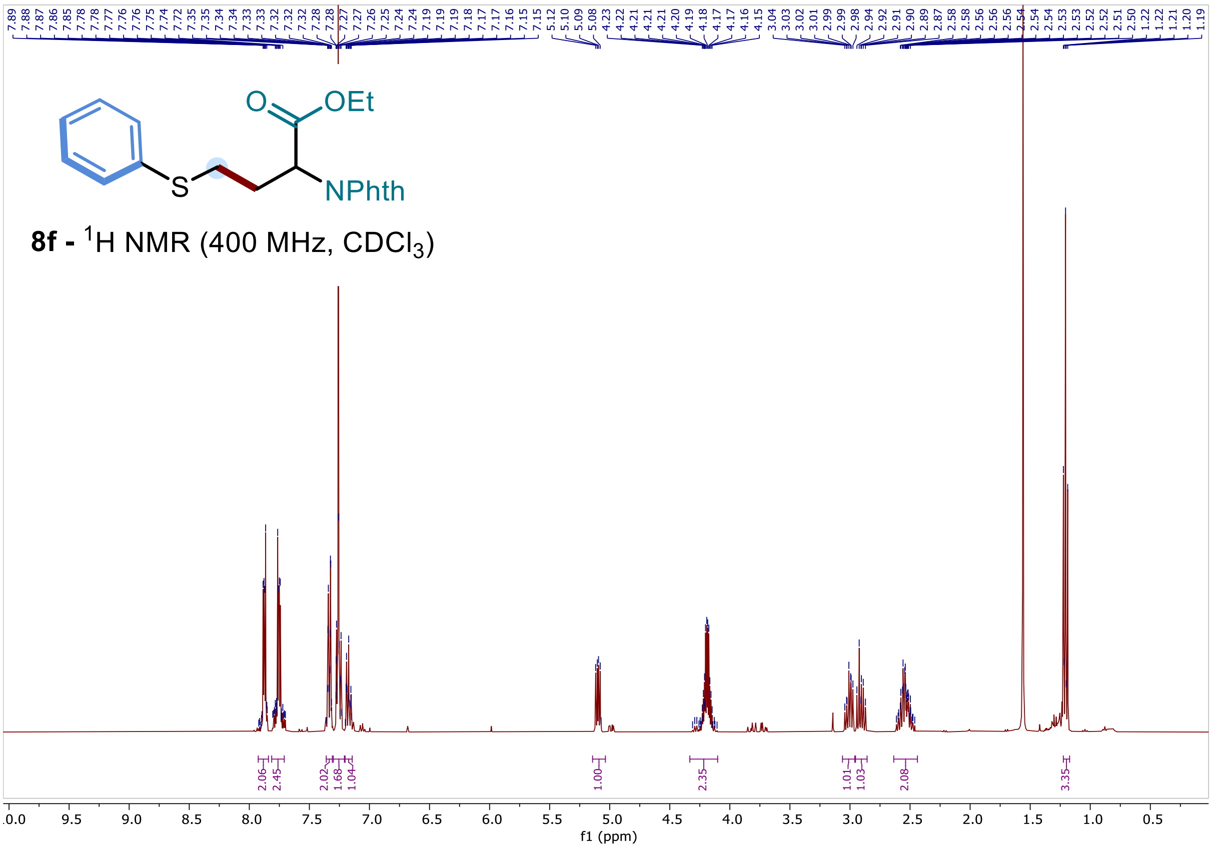
O
OEt
S
NPhth
8f - ^{1}H NMR (400 MHz, CDCl$_3$)
f1 (ppm)

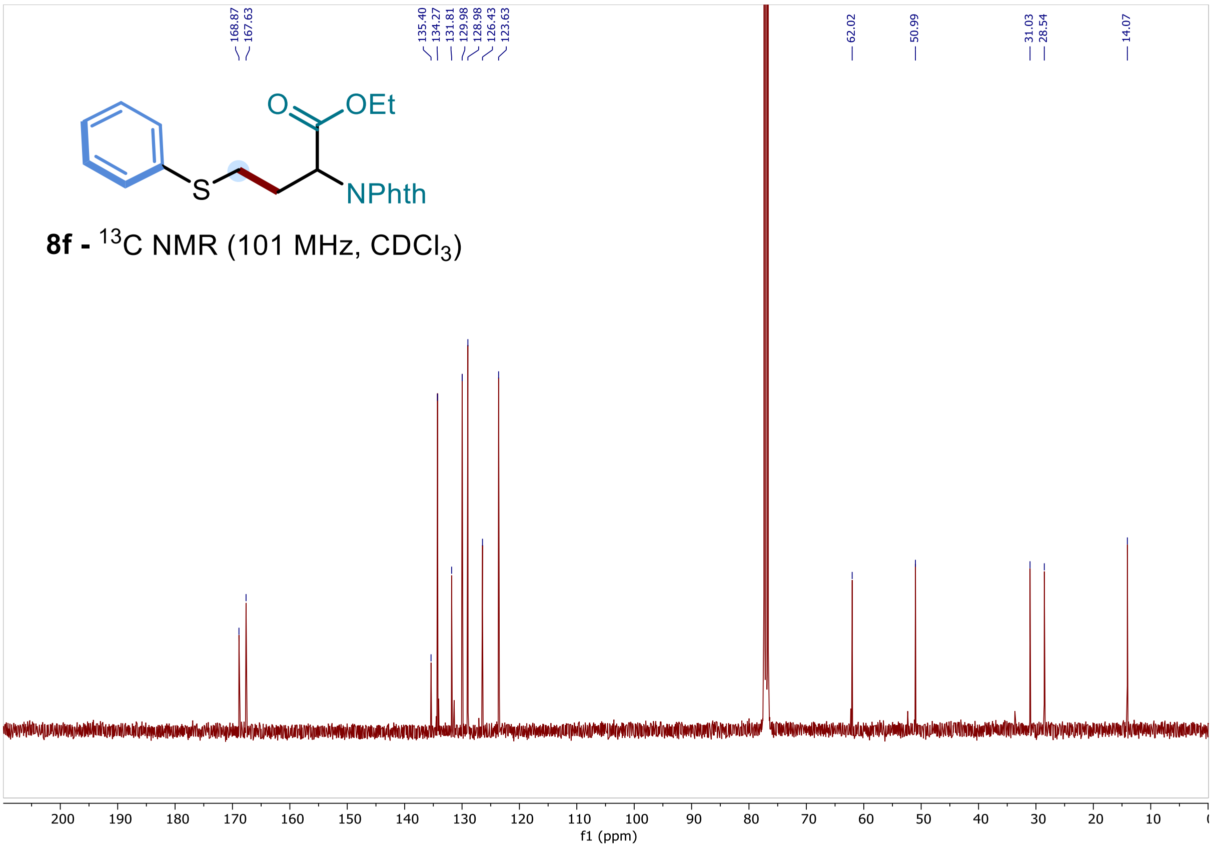
O
OEt
S
NPhth
8f - ^{13}C NMR (101 MHz, CDCl$_3$)
168.87
167.63
135.40
134.27
131.81
129.98
128.98
126.43
123.63
62.02
50.99
31.03
28.54
14.07
f1 (ppm)

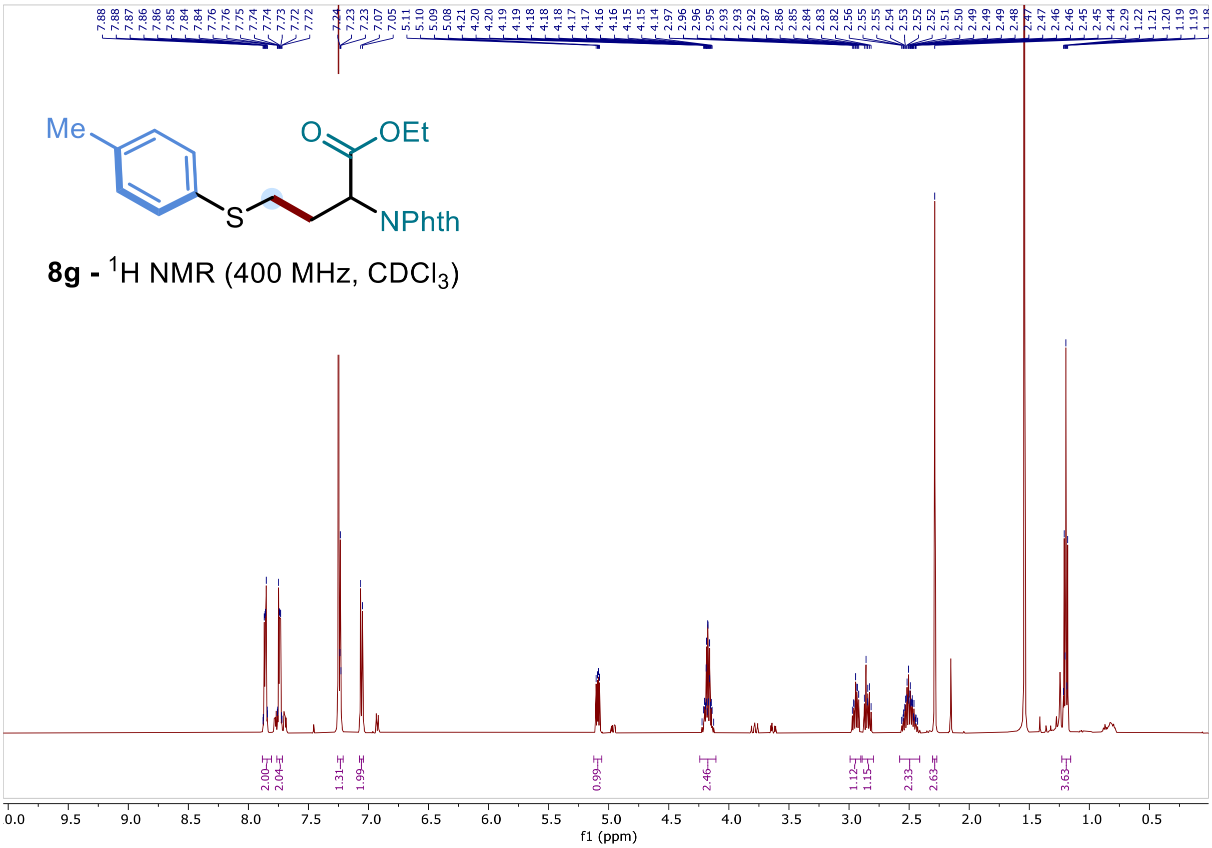
Me
O
OEt
S
NPhth
8g - ^{1}H NMR (400 MHz, CDCl$_3$)
f1 (ppm)

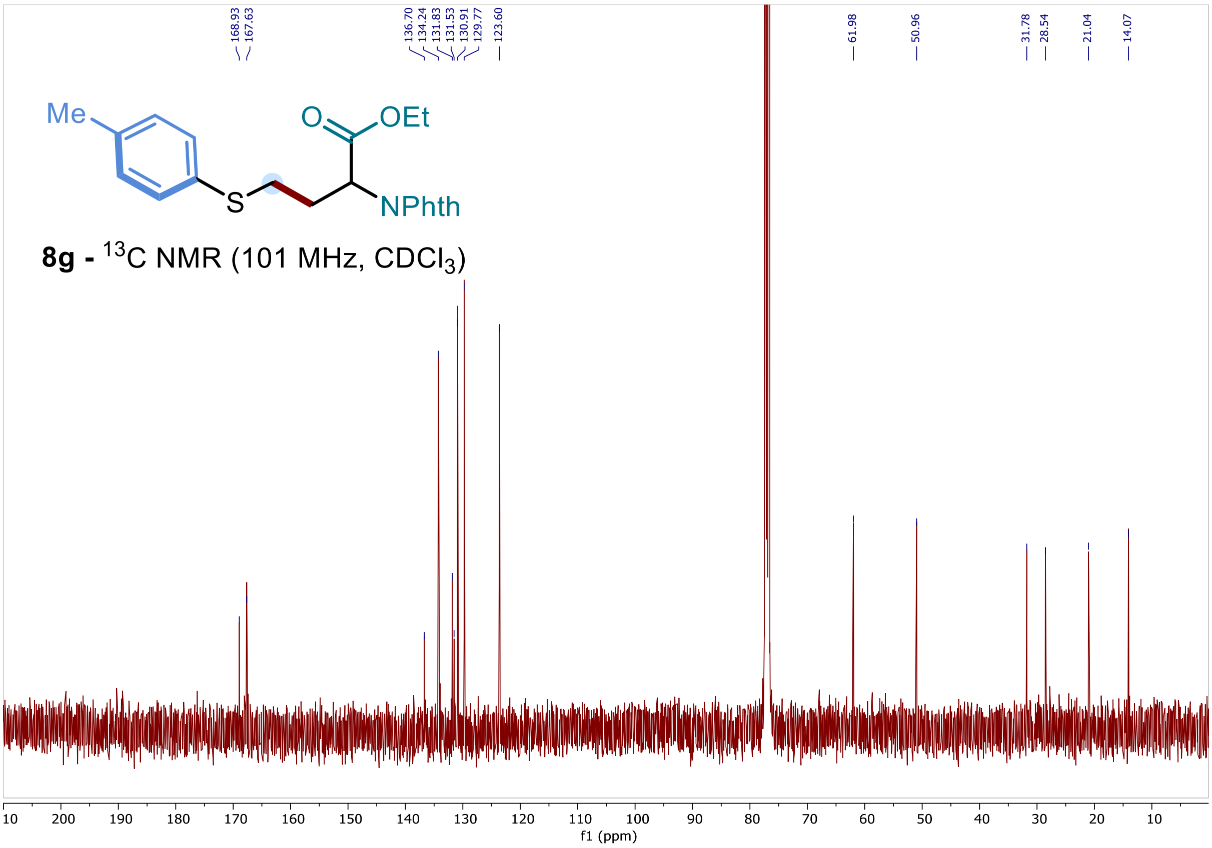
Me
O
OEt
S
NPhth
8g - ^{13}C NMR (101 MHz, CDCl$_3$)
168.93
167.63
136.70
134.24
131.83
131.53
130.91
129.77
123.60
61.98
50.96
31.78
28.54
21.04
14.07
f1 (ppm)

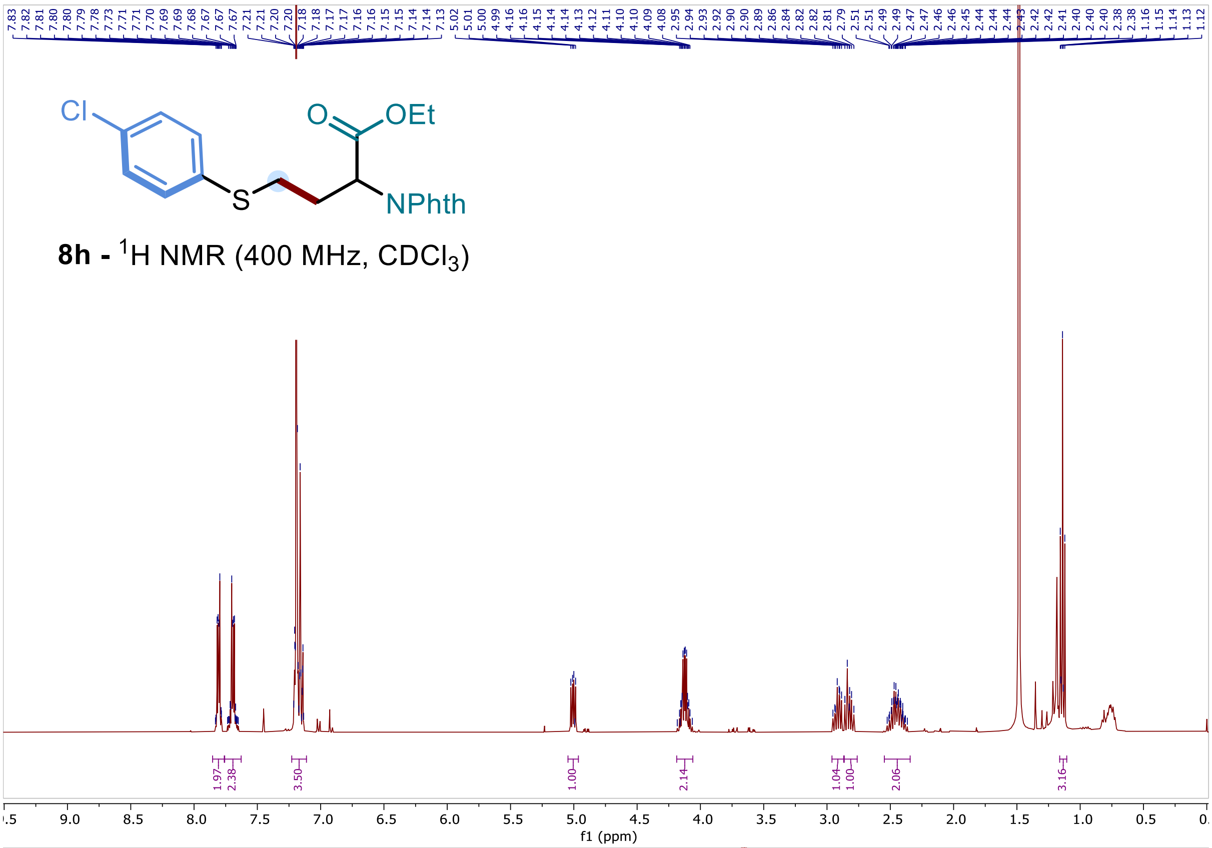

Cl
O
OEt
S
NPhth
8h - ^{1}H NMR (400 MHz, CDCl3)
1.97
2.38
3.50
1.00
2.14
1.04
1.00
2.06
3.16
f1 (ppm)

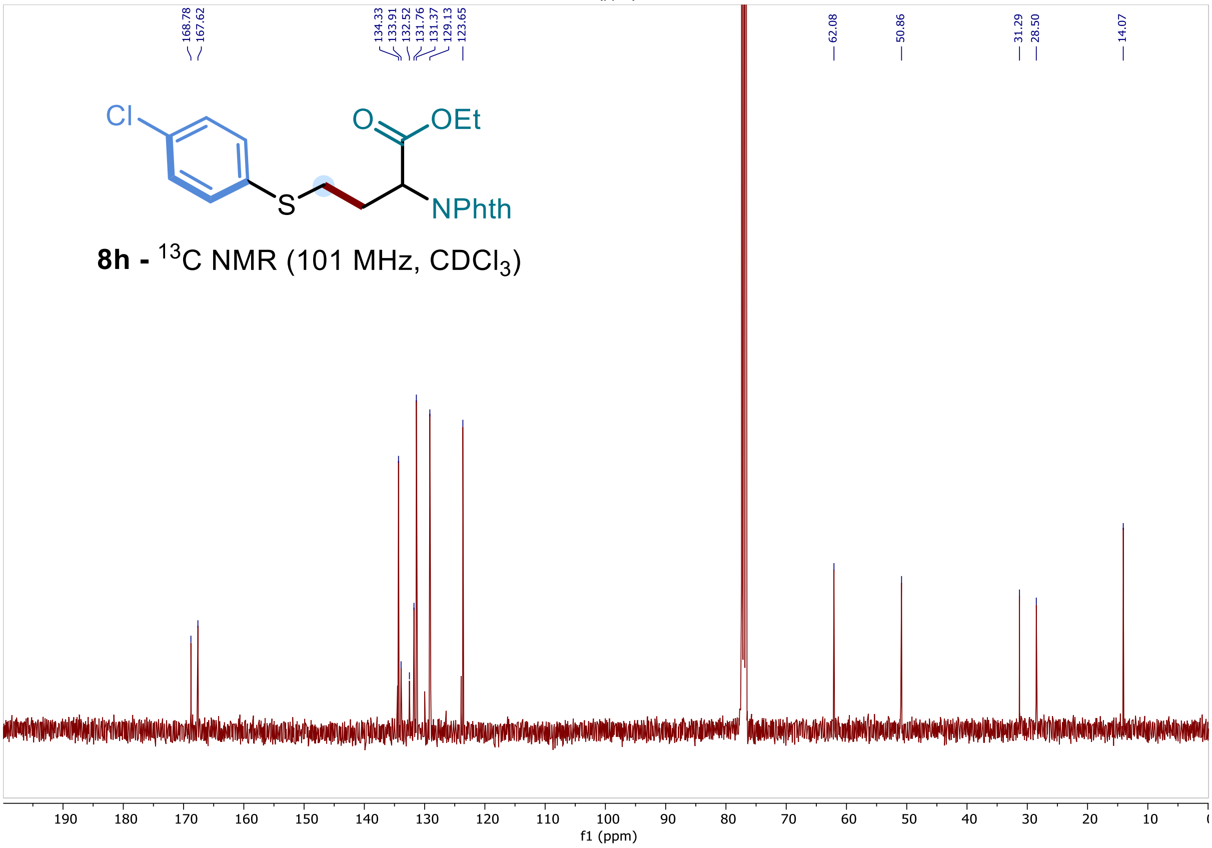

168.78
167.62
134.33
133.91
132.52
131.76
131.37
129.13
123.65
62.08
50.86
31.29
28.50
14.07
Cl
O
OEt
S
NPhth
8h - ^{13}C NMR (101 MHz, CDCl3)
f1 (ppm)

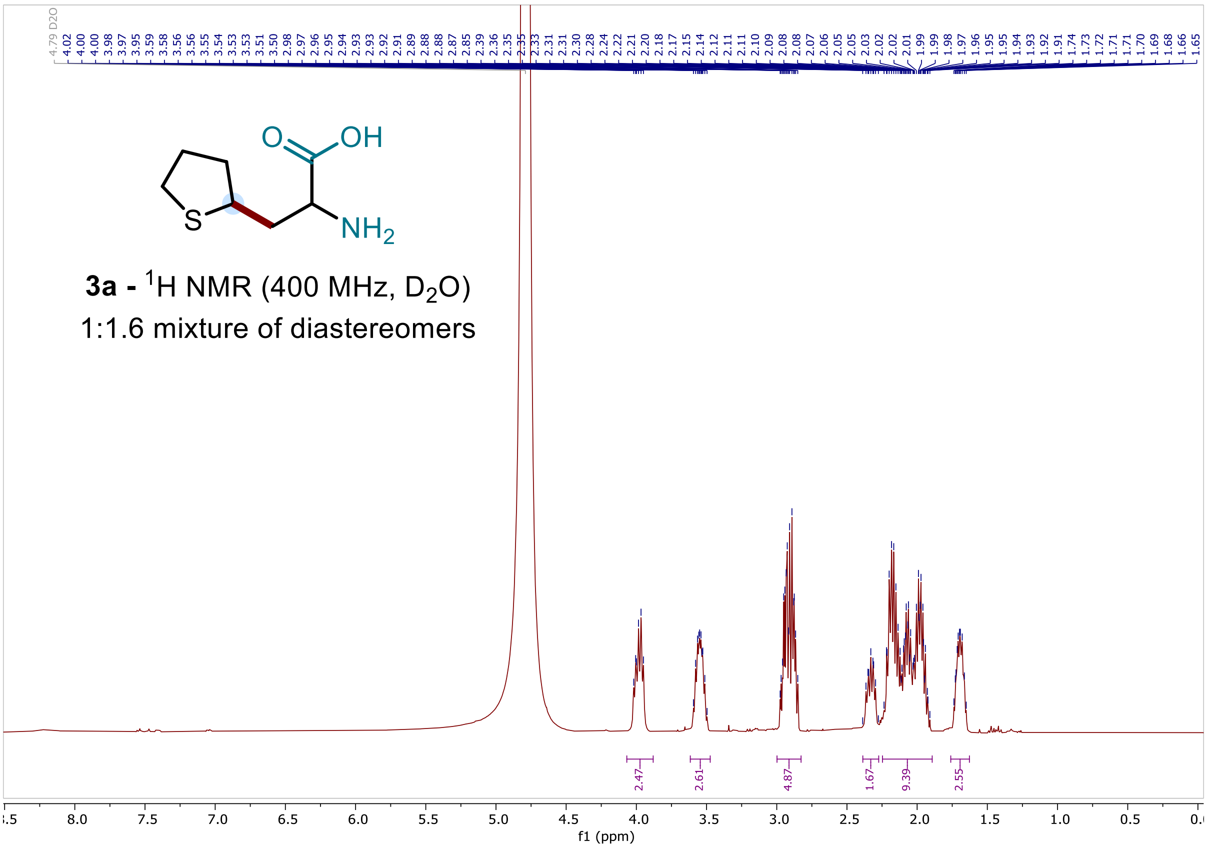
3a - 1H NMR (400 MHz, D2O)
1:1.6 mixture of diastereomers
f1 (ppm)

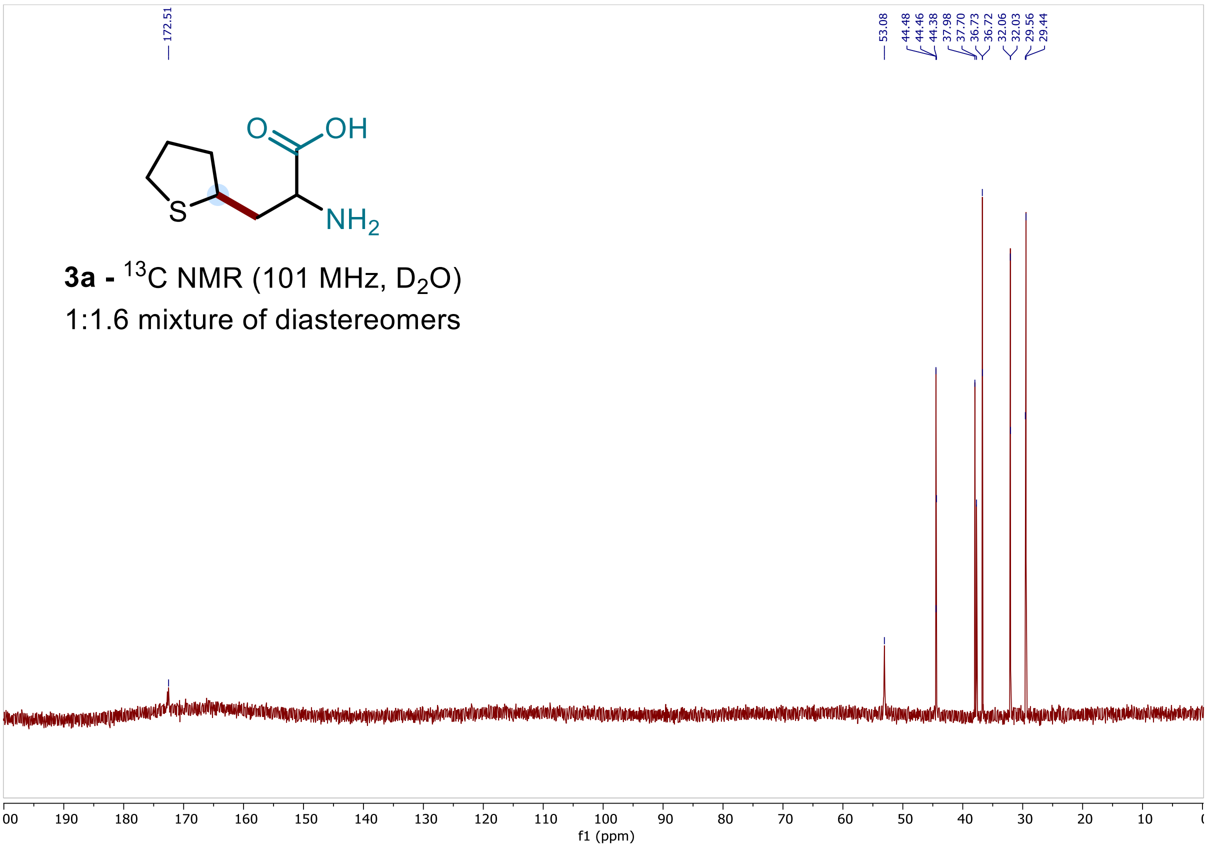
3a - 13C NMR (101 MHz, D2O)
1:1.6 mixture of diastereomers
172.51
53.08
44.48
44.46
44.38
37.98
37.70
36.73
36.72
32.06
32.03
29.56
29.44
f1 (ppm)

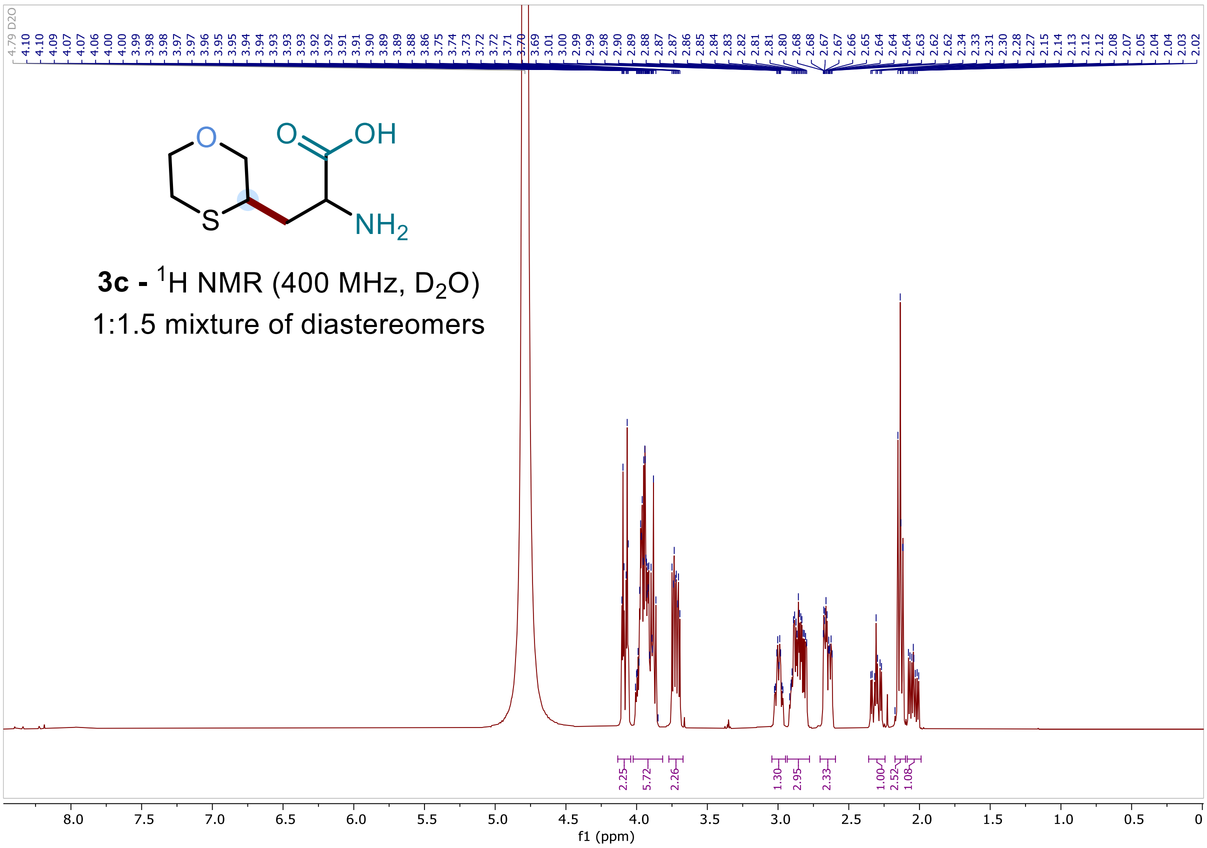
O
OH
S
NH2
3c - 1H NMR (400 MHz, D2O)
1:1.5 mixture of diastereomers
f1 (ppm)

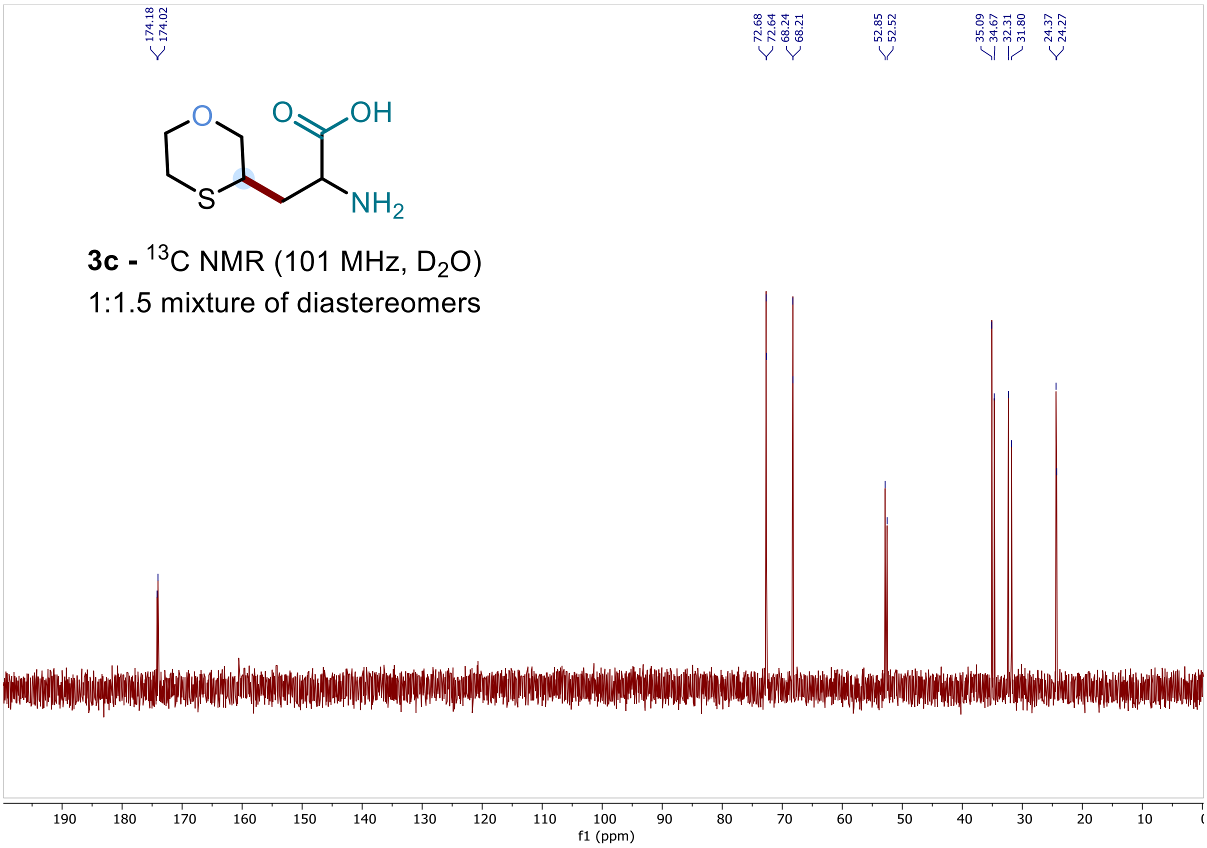
174.18
174.02
72.68
72.64
68.24
68.21
52.85
52.52
35.09
34.67
32.31
31.80
24.37
24.27
O
OH
S
NH2
3c - 13C NMR (101 MHz, D2O)
1:1.5 mixture of diastereomers
f1 (ppm)

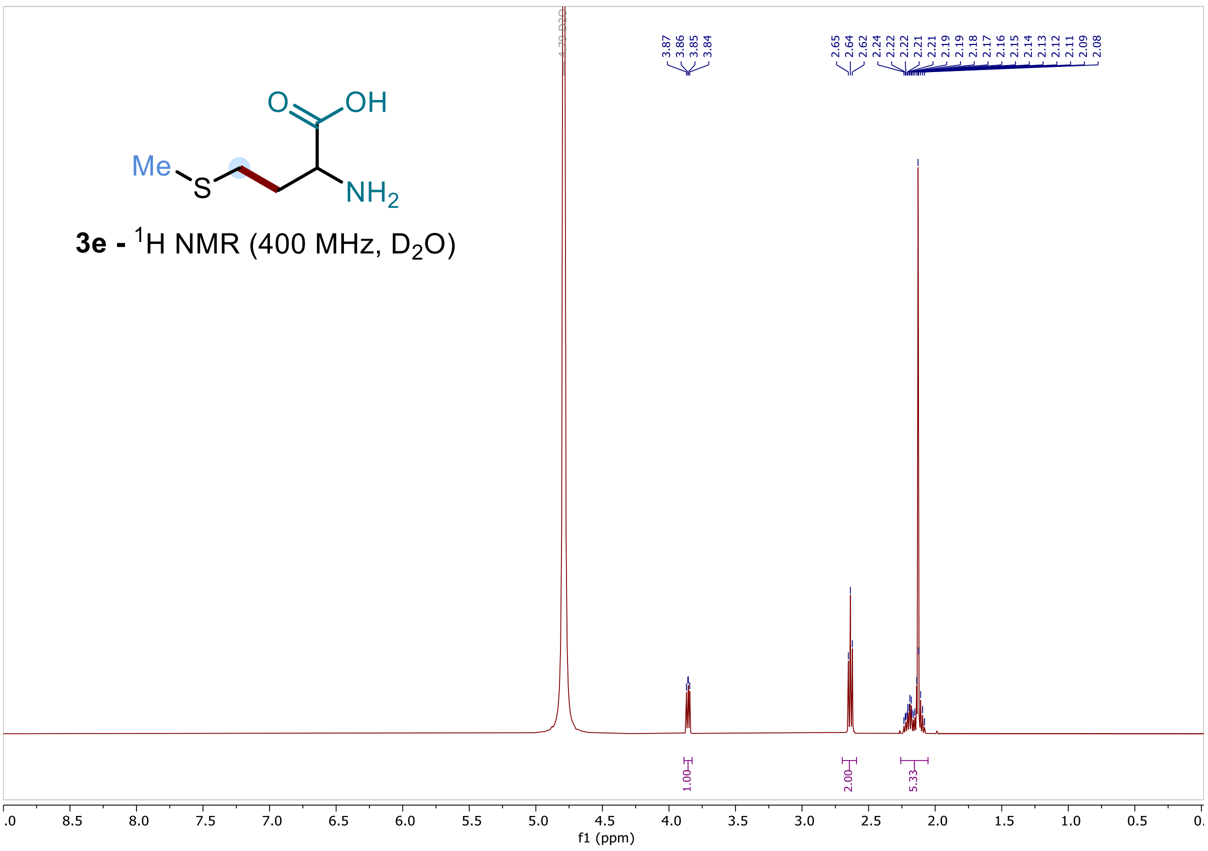
3e - 1H NMR (400 MHz, D2O)
O
OH
Me
S
NH2
3.87
3.86
3.85
3.84
2.65
2.64
2.62
2.24
2.22
2.22
2.21
2.21
2.19
2.19
2.18
2.17
2.16
2.15
2.14
2.13
2.12
2.11
2.09
2.08
1.00
2.00
5.33
f1 (ppm)

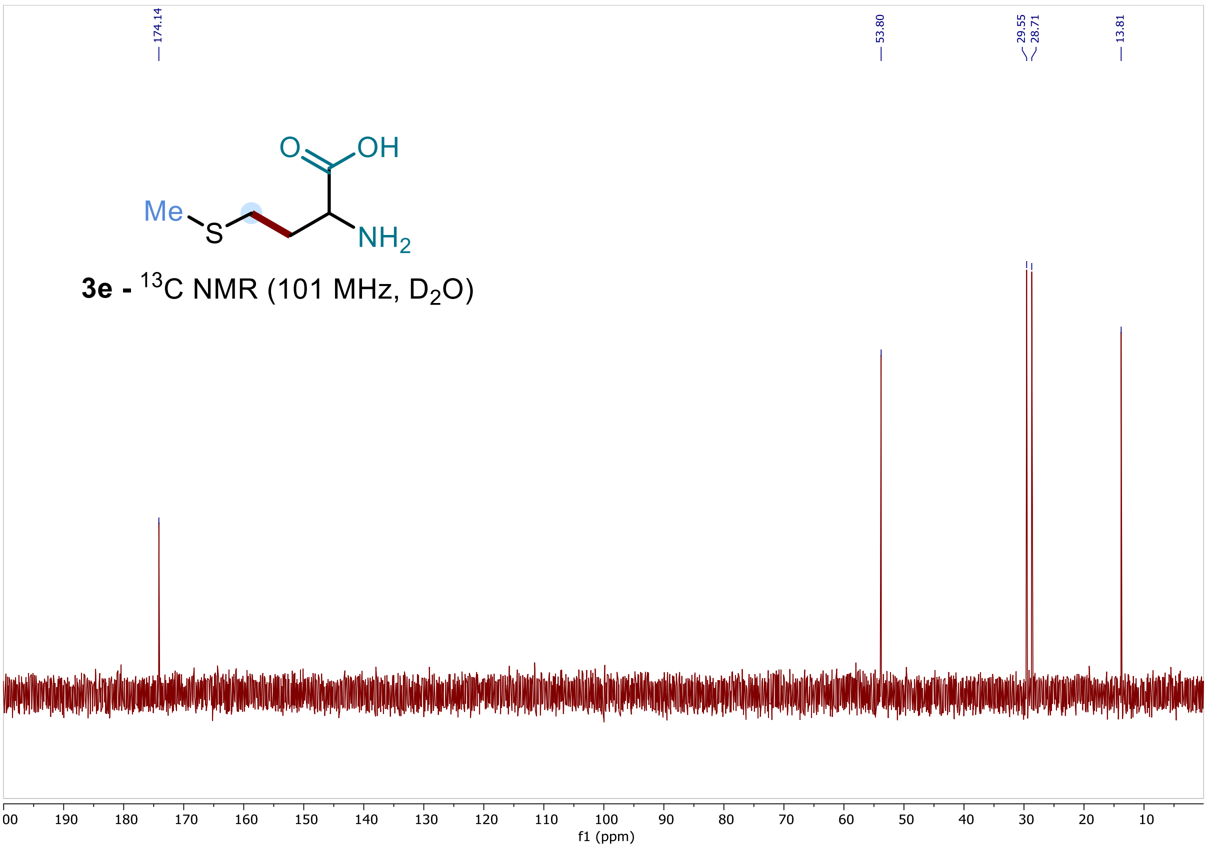
3e - 13C NMR (101 MHz, D2O)
O
OH
Me
S
NH2
174.14
53.80
29.55
28.71
13.81
f1 (ppm)

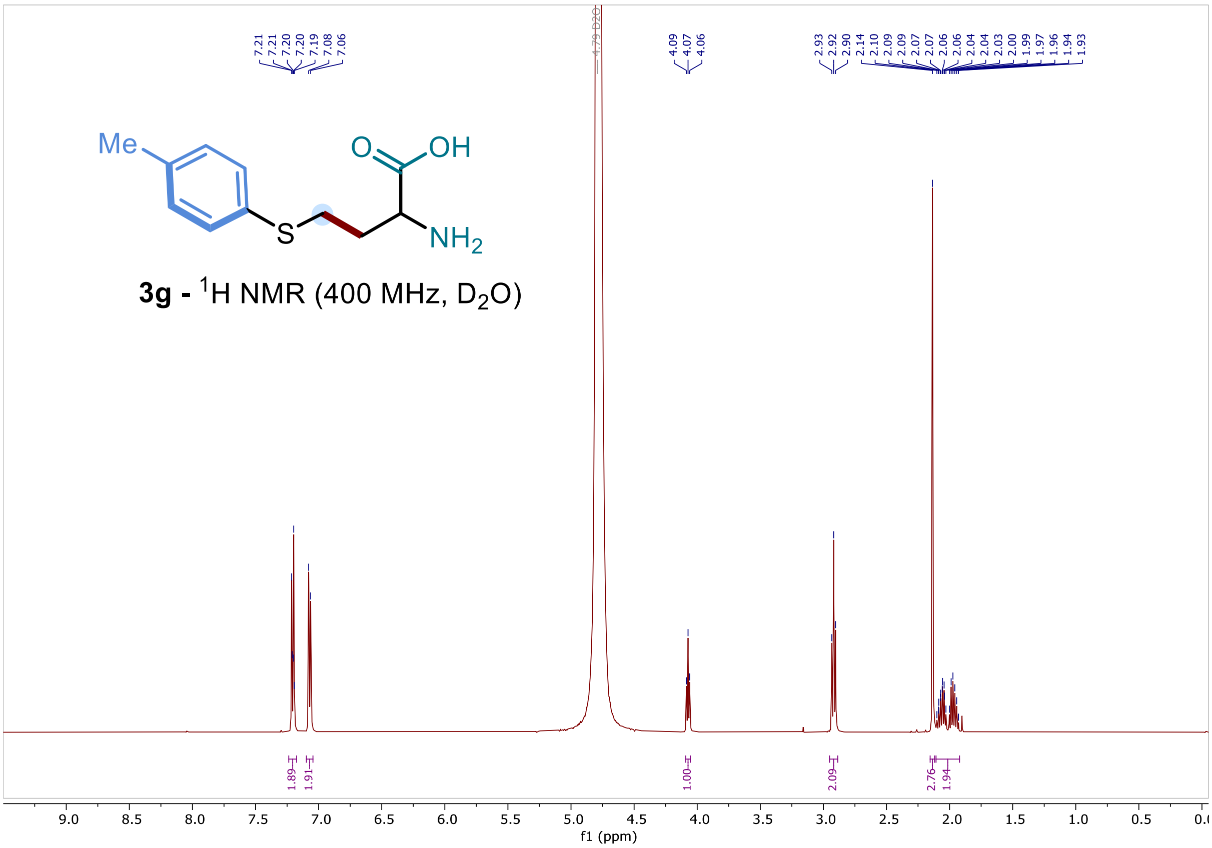
Me
O
OH
S
NH2
3g - 1H NMR (400 MHz, D2O)
7.21
7.21
7.20
7.20
7.19
7.08
7.06
4.79 D2O
4.09
4.07
4.06
2.93
2.92
2.90
2.14
2.10
2.09
2.09
2.07
2.07
2.06
2.06
2.04
2.04
2.03
2.00
1.99
1.97
1.96
1.94
1.93
1.89
1.91
1.00
2.09
2.76
1.94
9.0
8.5
8.0
7.5
7.0
6.5
6.0
5.5
5.0
4.5
4.0
3.5
3.0
2.5
2.0
1.5
1.0
0.5
f1 (ppm)

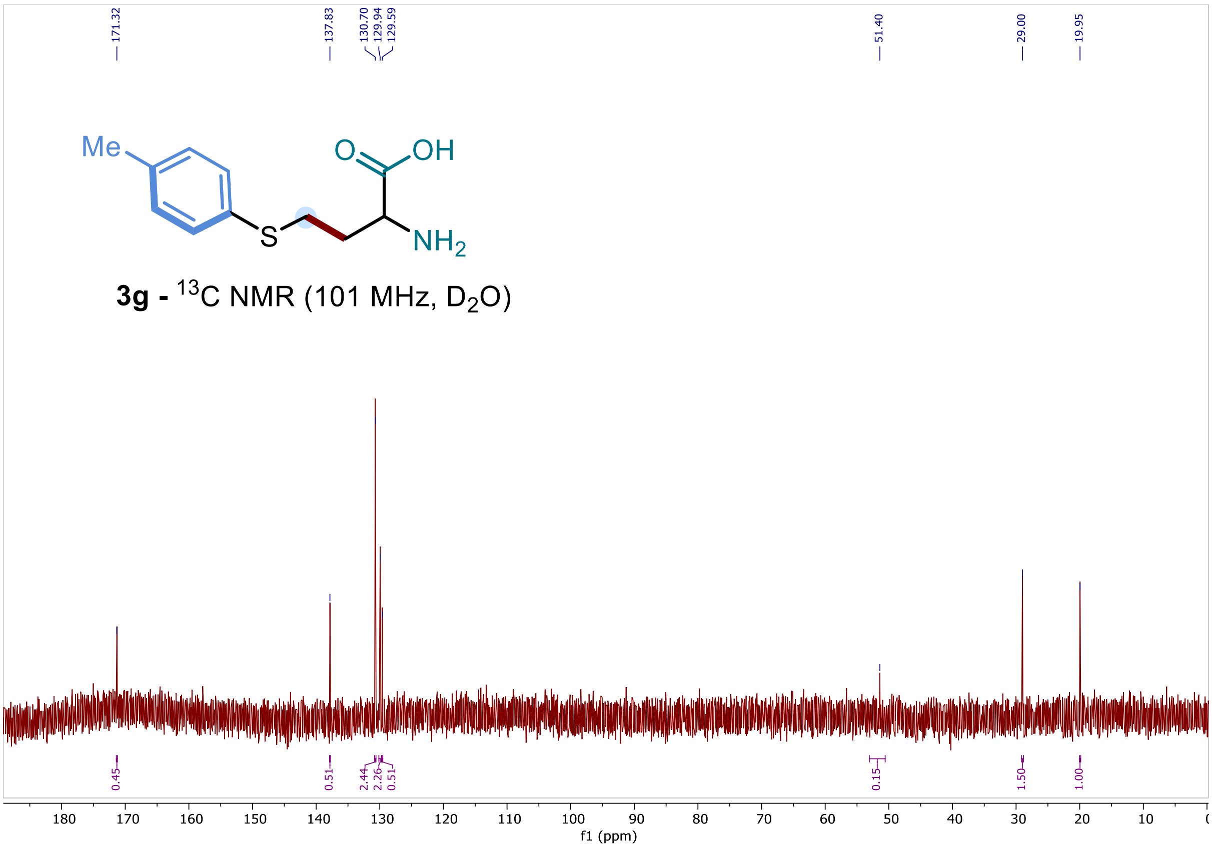
171.32
137.83
130.70
129.94
129.59
51.40
29.00
19.95
Me
O
OH
S
NH2
3g - 13C NMR (101 MHz, D2O)
0.45
0.51
2.44
2.26
0.51
0.15
1.50
1.00
180
170
160
150
140
130
120
110
100
90
80
70
60
50
40
30
20
10
f1 (ppm)

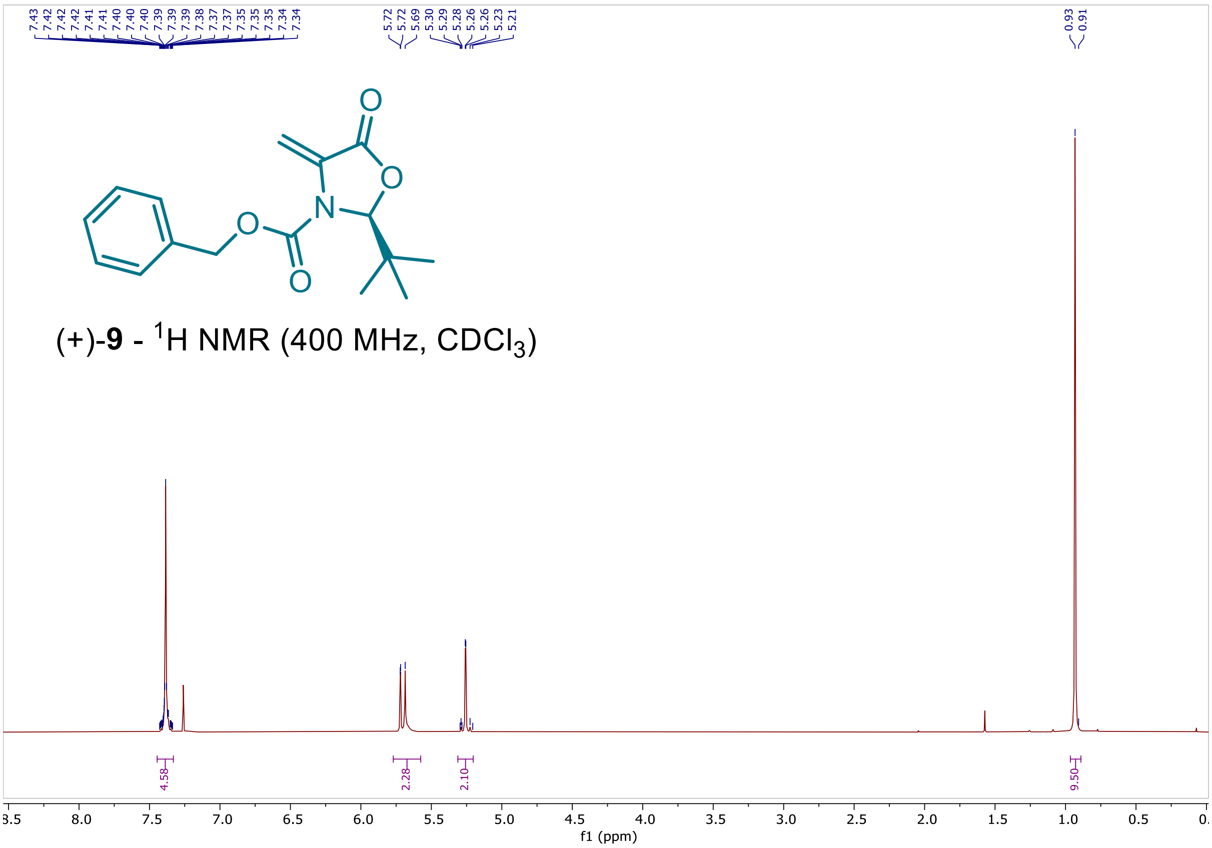

(+)-**9** - ^{1}H NMR (400 MHz, $CDCl_3$)

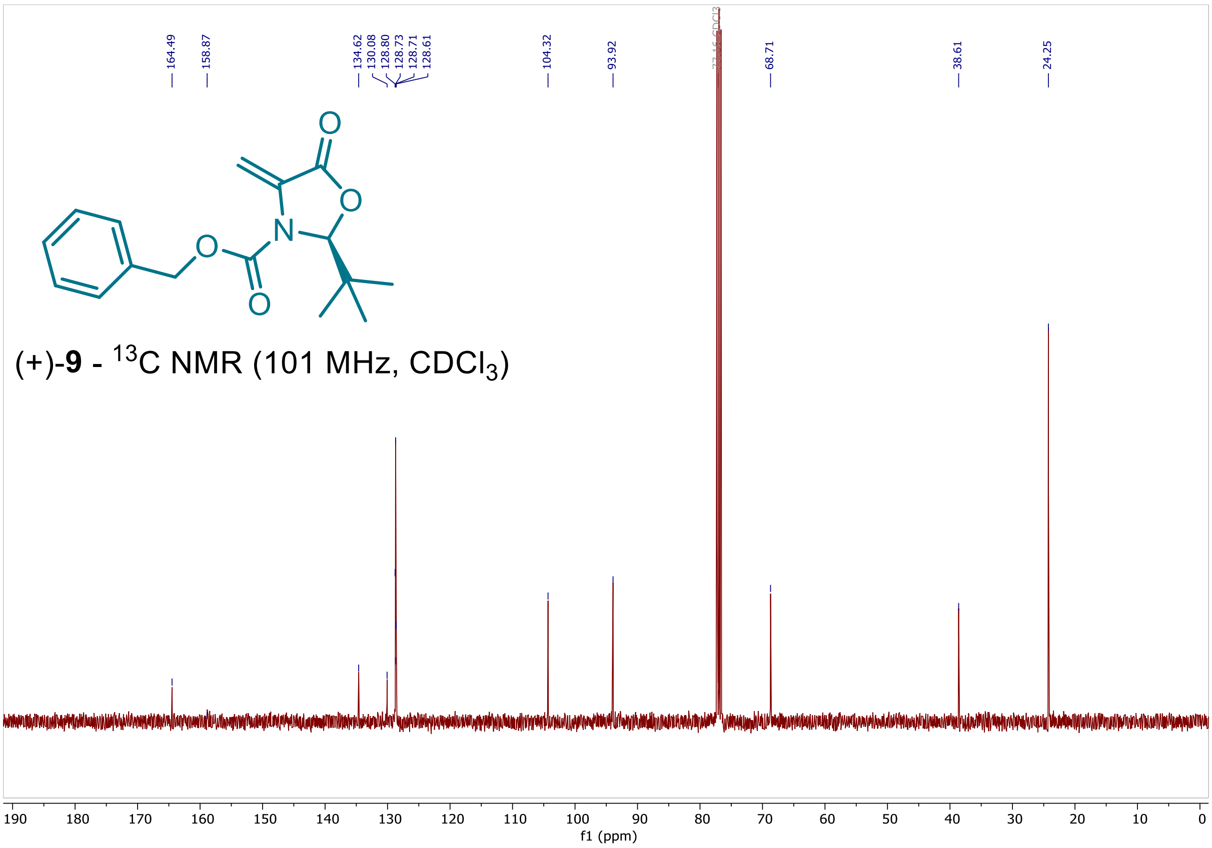

(+)-**9** - ^{13}C NMR (101 MHz, $CDCl_3$)

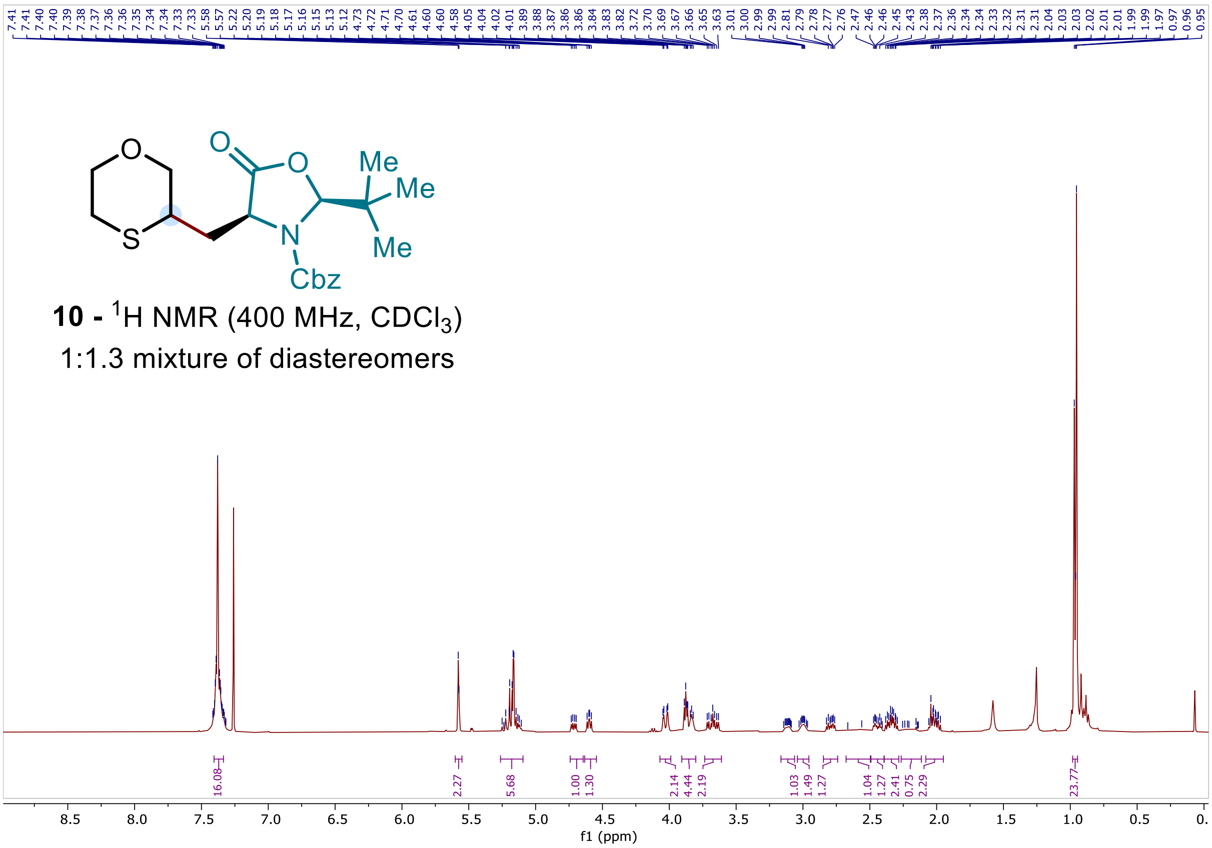
10 - 1H NMR (400 MHz, CDCl3)
1:1.3 mixture of diastereomers
f1 (ppm)

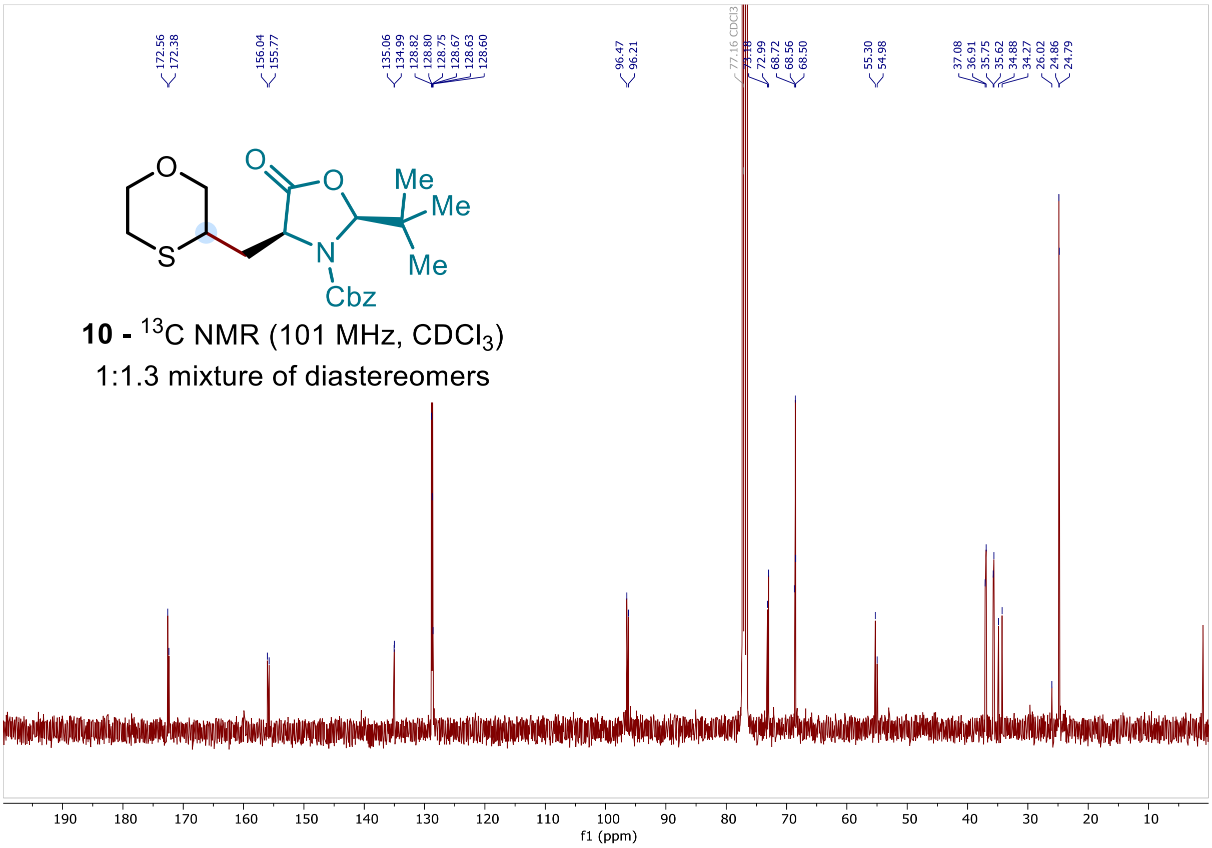
10 - 13C NMR (101 MHz, CDCl3)
1:1.3 mixture of diastereomers
f1 (ppm)

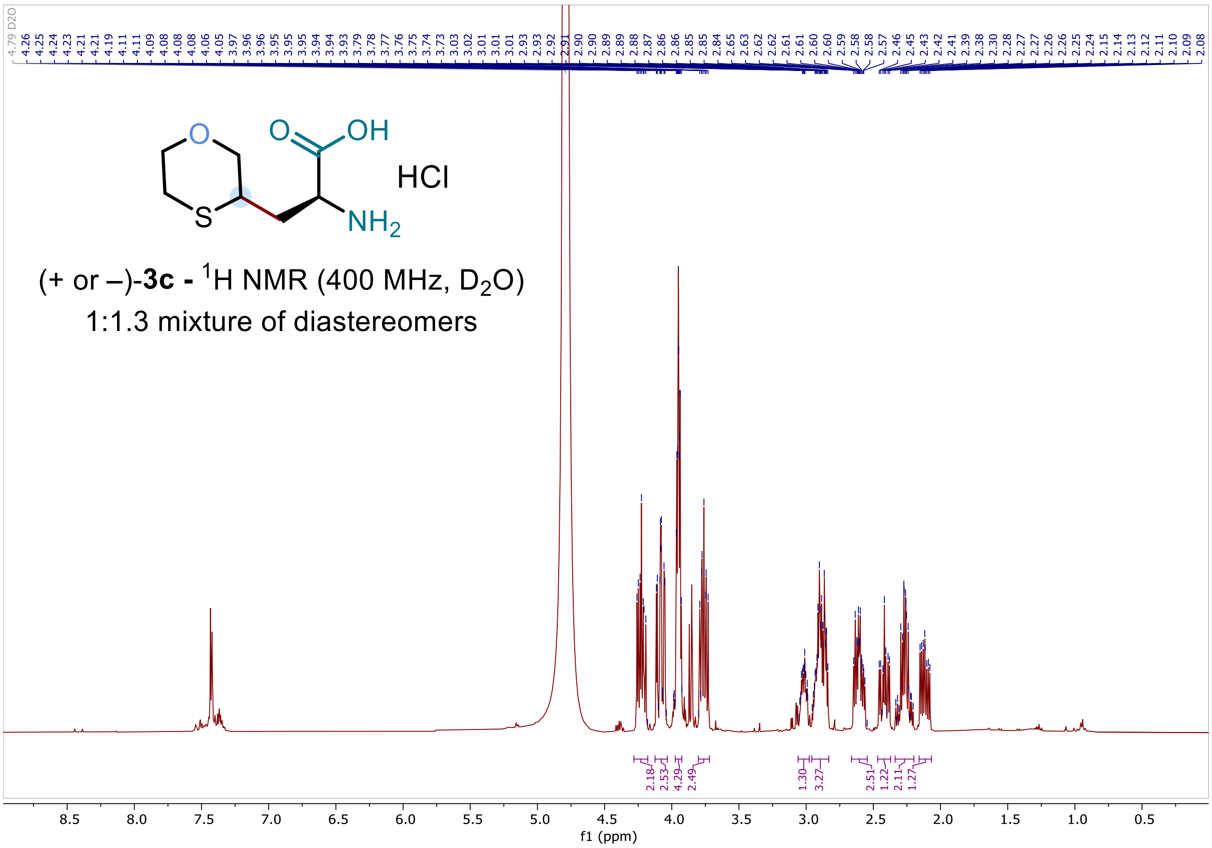

(+ or –)-**3c** - ^{1}H NMR (400 MHz, D_2O)

1:1.3 mixture of diastereomers

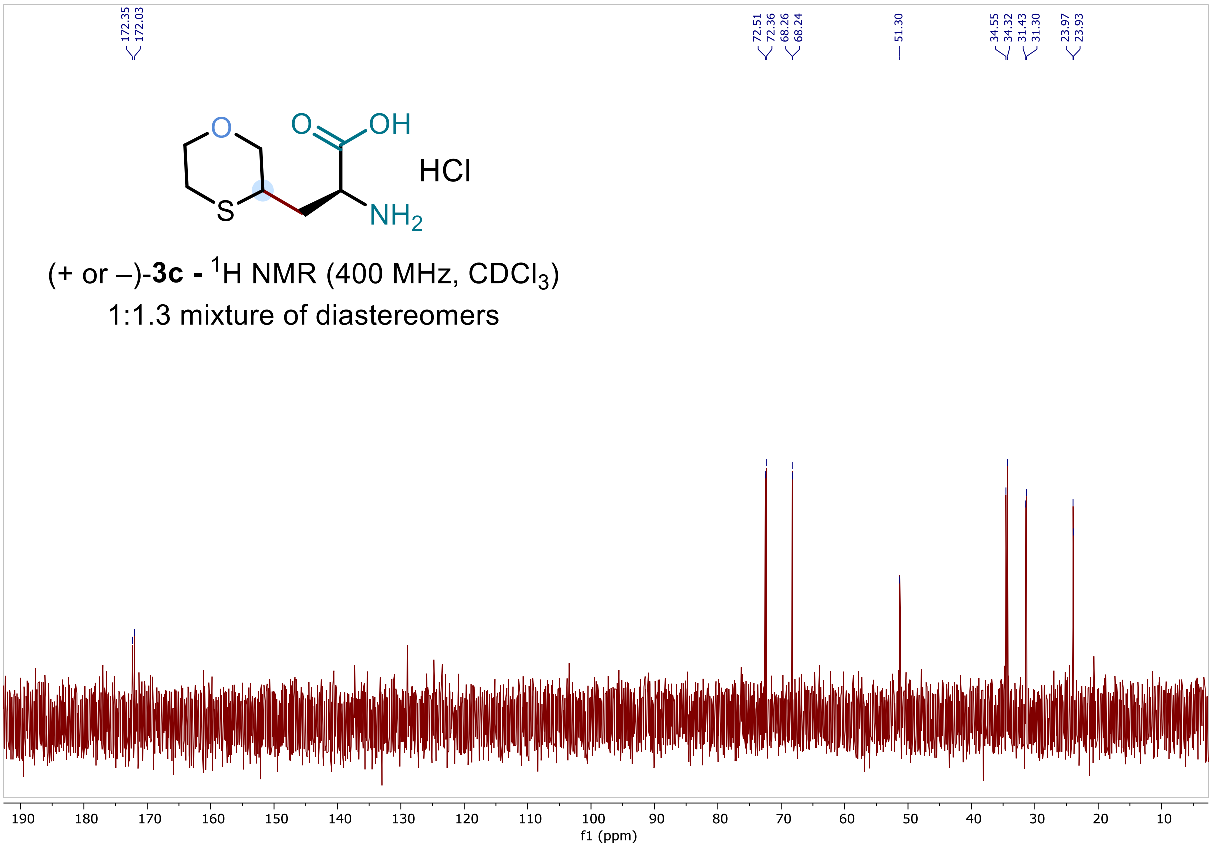

(+ or –)-**3c** - ^{1}H NMR (400 MHz, $CDCl_3$)

1:1.3 mixture of diastereomers

Dipeptide Starting Materials

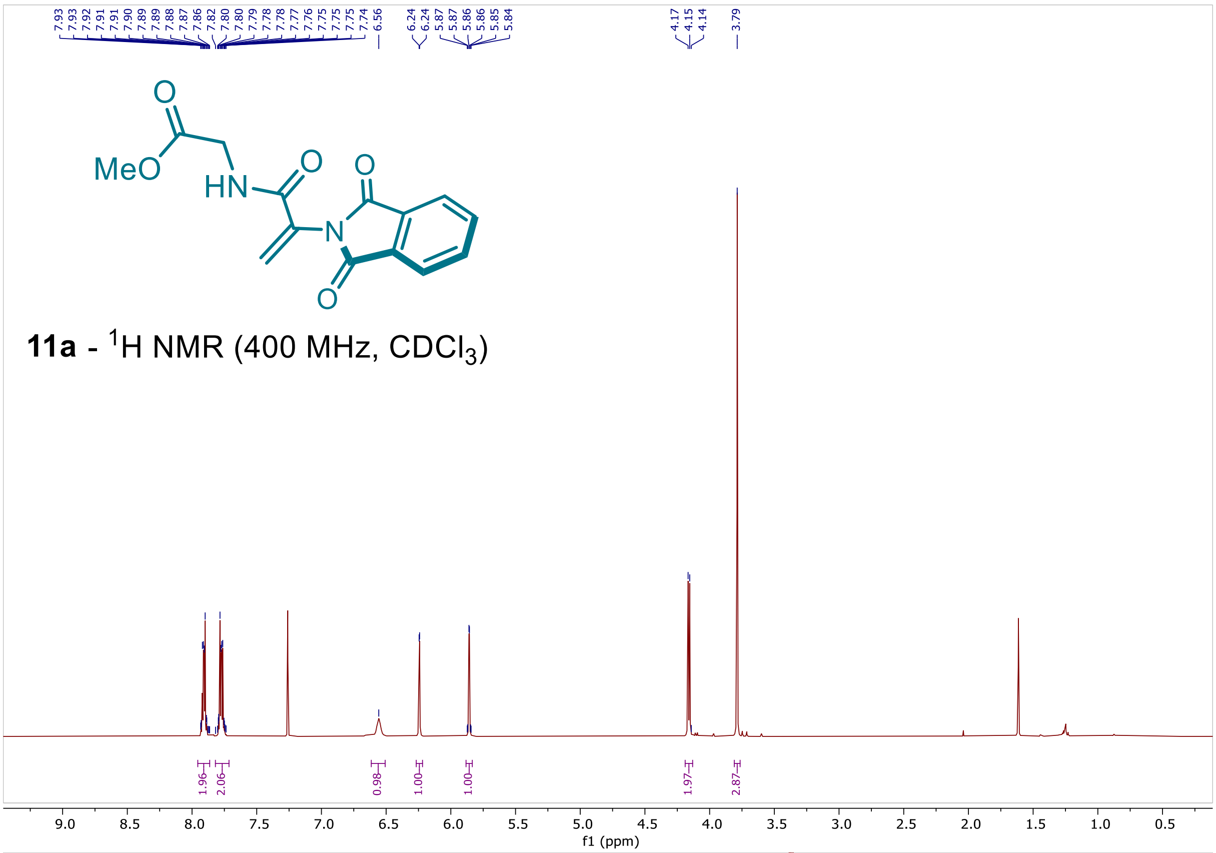

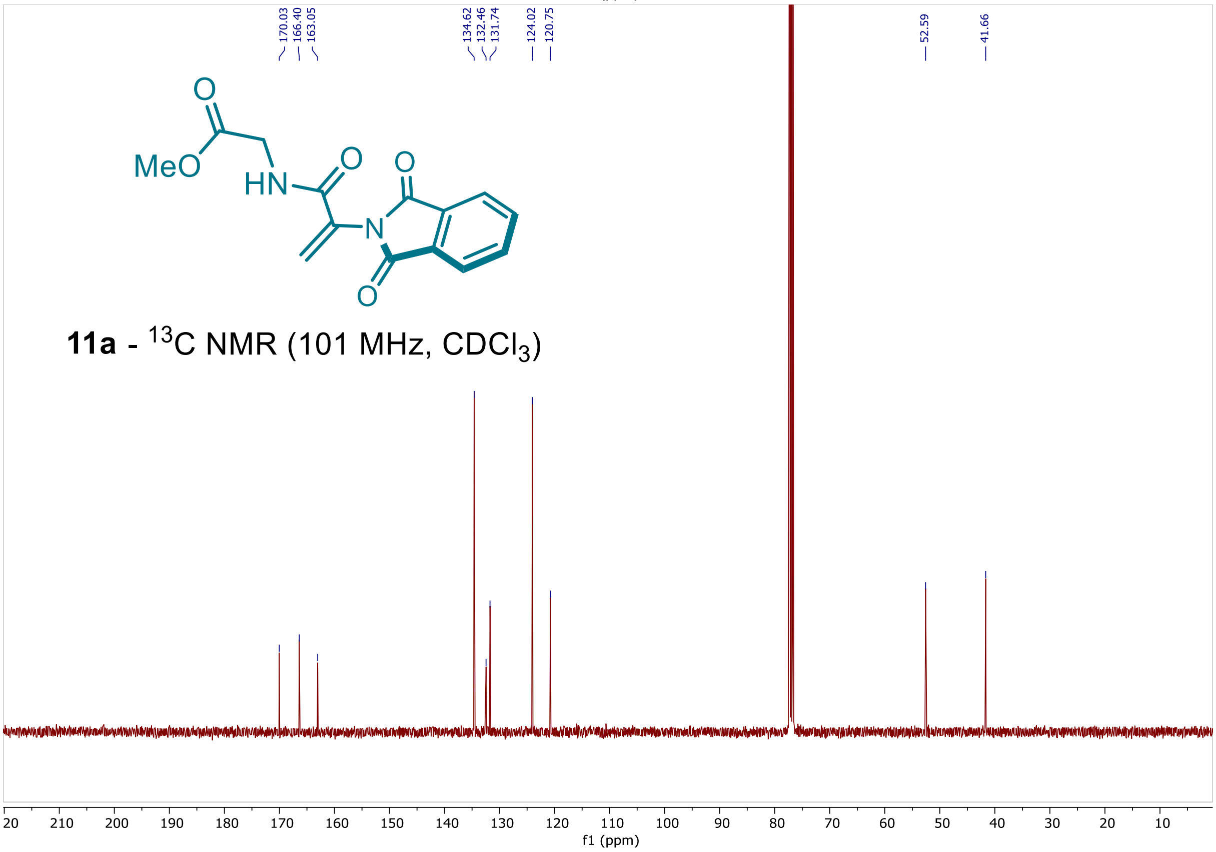

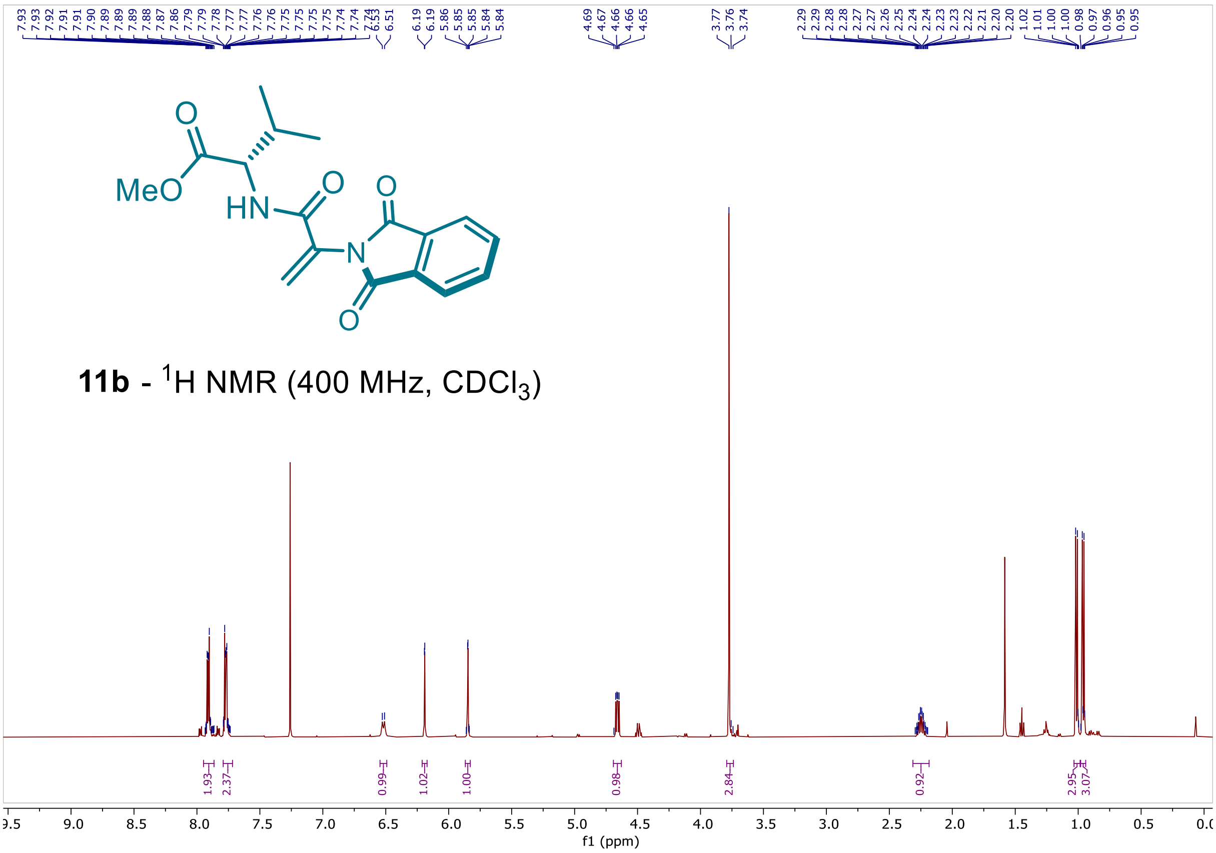

11b - ^{1}H NMR (400 MHz, $CDCl_3$)

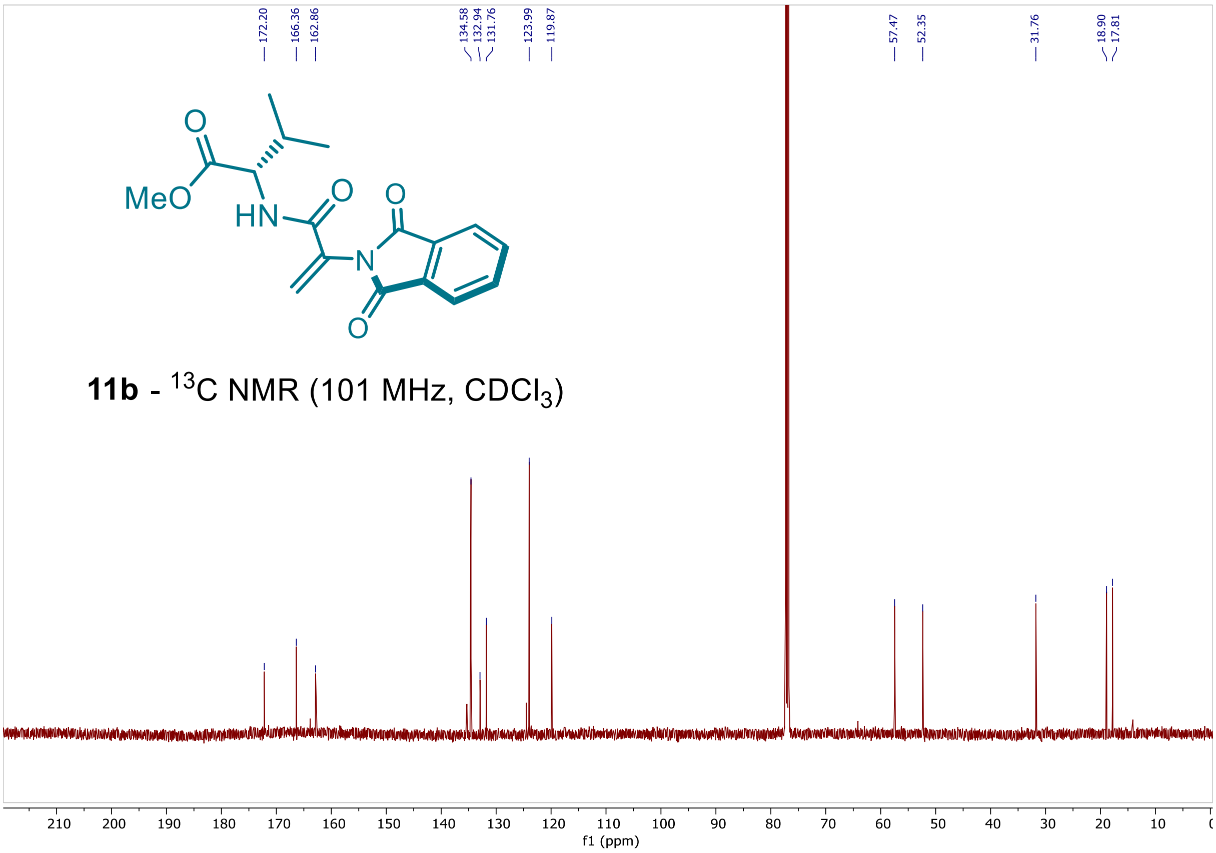

11b - ^{13}C NMR (101 MHz, $CDCl_3$)

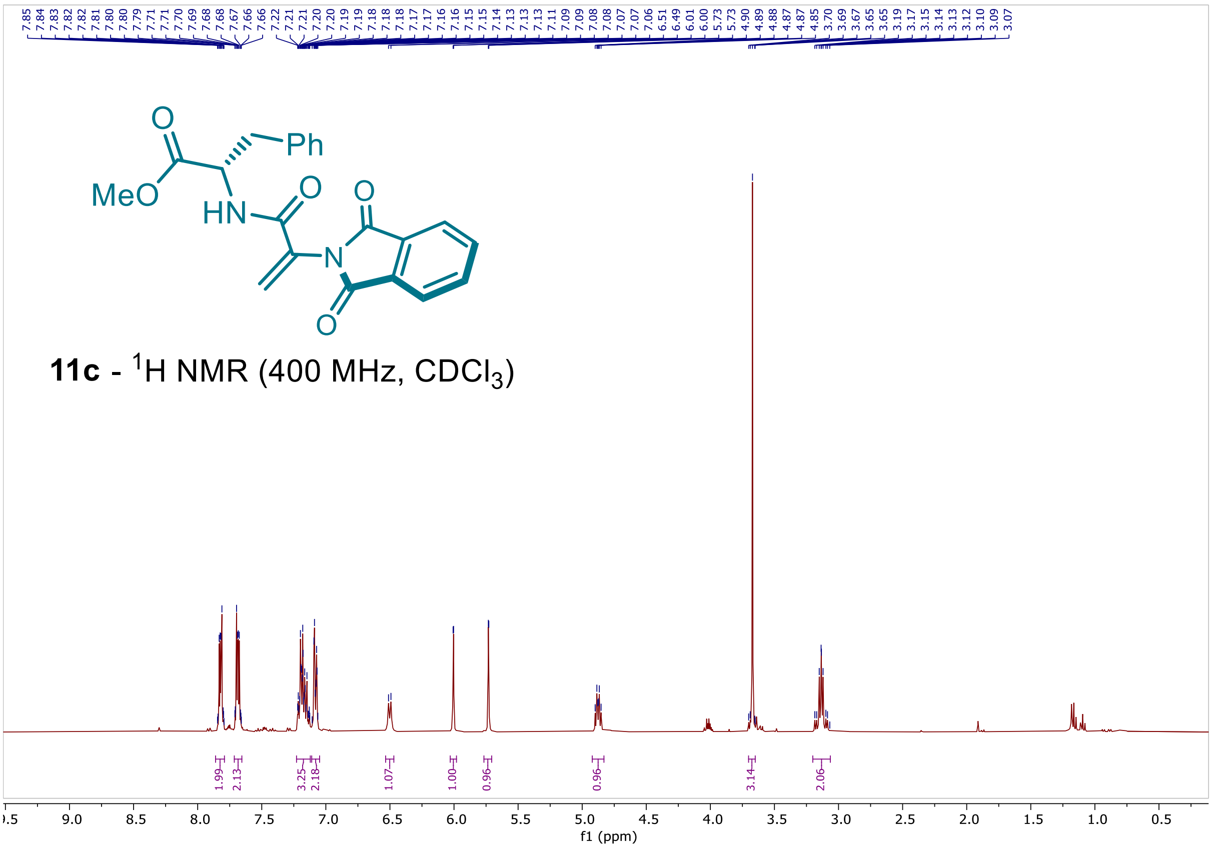

11c - 1H NMR (400 MHz, CDCl3)
MeO
HN
N
Ph
f1 (ppm)

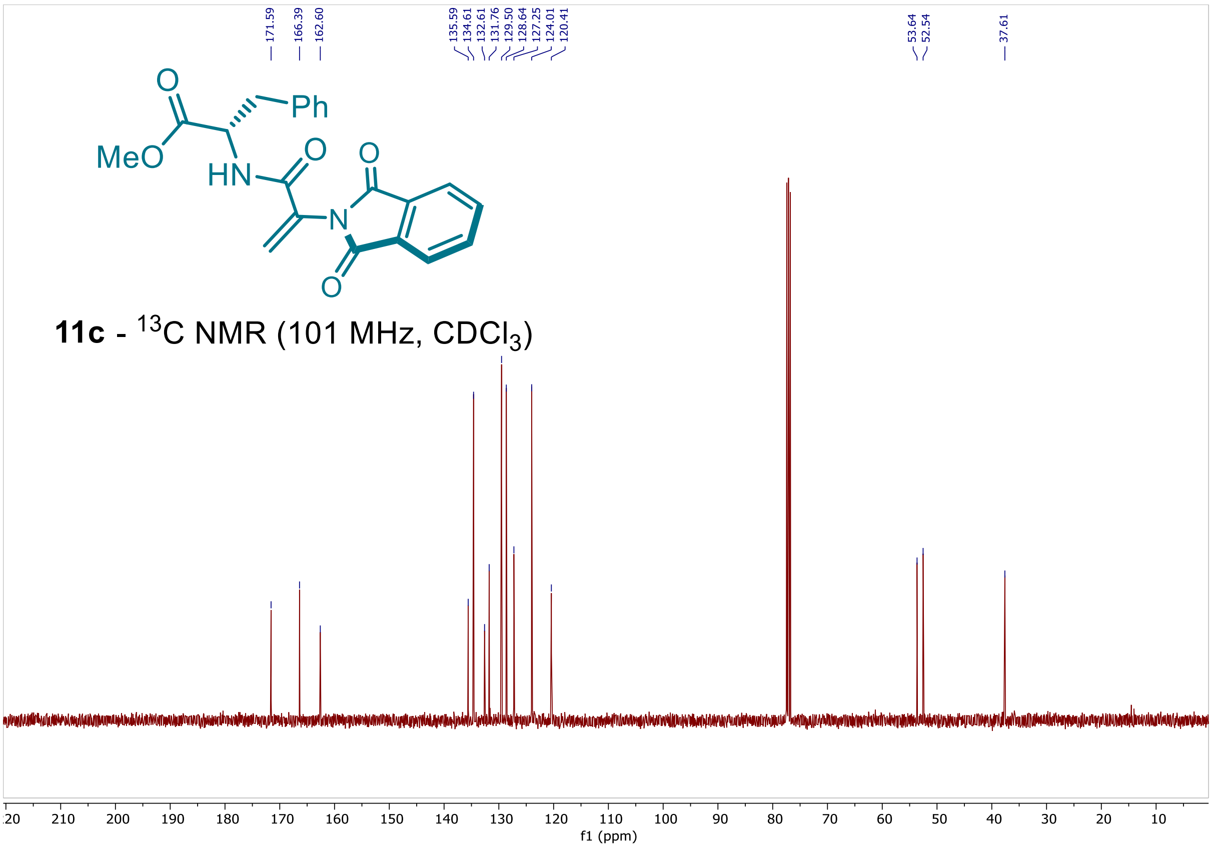

11c - 13C NMR (101 MHz, CDCl3)
171.59
166.39
162.60
53.64
52.54
37.61
f1 (ppm)

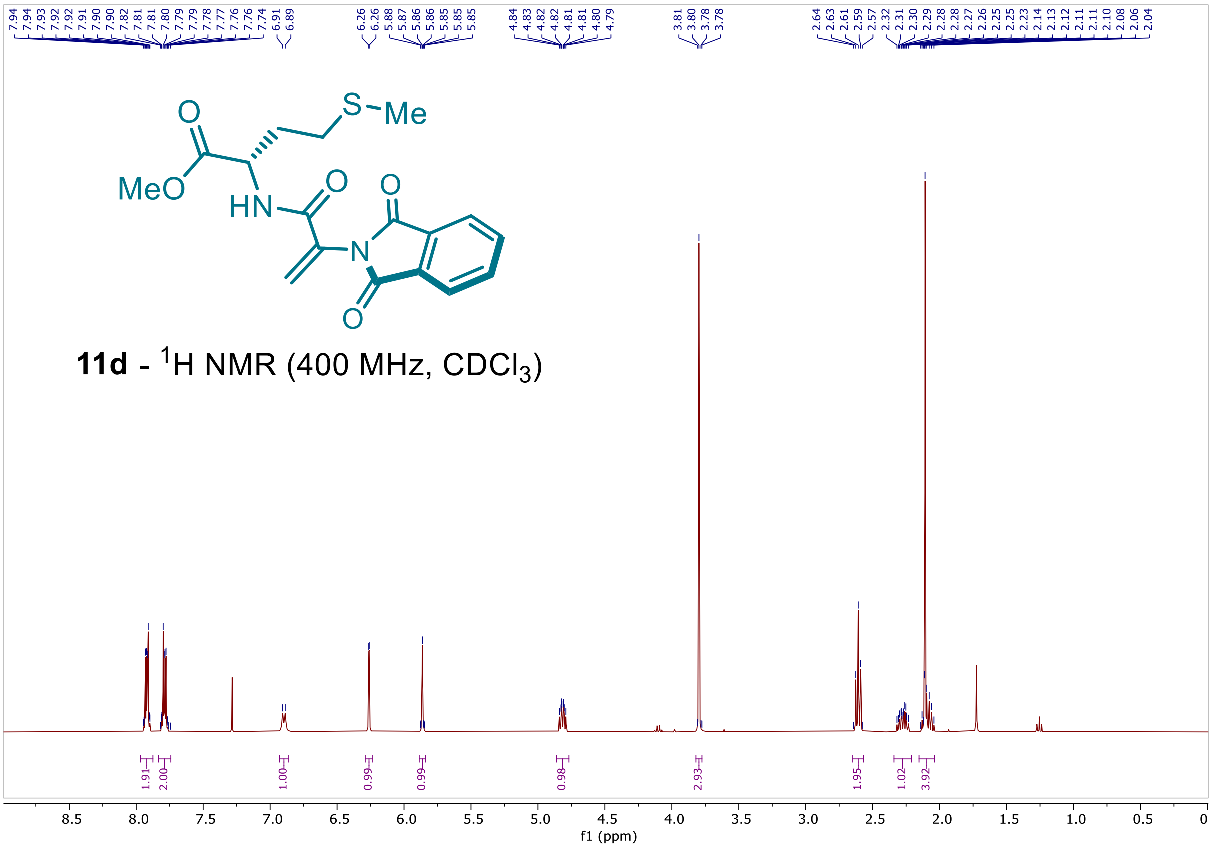
7.94 7.94 7.93 7.92 7.92 7.91 7.90 7.90 7.82 7.81 7.81 7.80 7.79 7.79 7.78 7.77 7.76 7.76 7.74 6.91 6.89
6.26 6.26 5.88 5.87 5.86 5.86 5.85 5.85 5.85
4.84 4.83 4.82 4.82 4.81 4.81 4.80 4.79
3.81 3.80 3.78 3.78
2.64 2.63 2.61 2.59 2.57 2.32 2.31 2.30 2.29 2.28 2.28 2.27 2.26 2.25 2.25 2.23 2.14 2.13 2.12 2.11 2.11 2.10 2.08 2.06 2.04
O
S
Me
MeO
HN
O
O
N
O
11d - ^{1}H NMR (400 MHz, CDCl$_3$)
1.91 2.00 1.00 0.99 0.99 0.98 2.93 1.95 1.02 3.92
8.5 8.0 7.5 7.0 6.5 6.0 5.5 5.0 4.5 4.0 3.5 3.0 2.5 2.0 1.5 1.0 0.5 0
f1 (ppm)

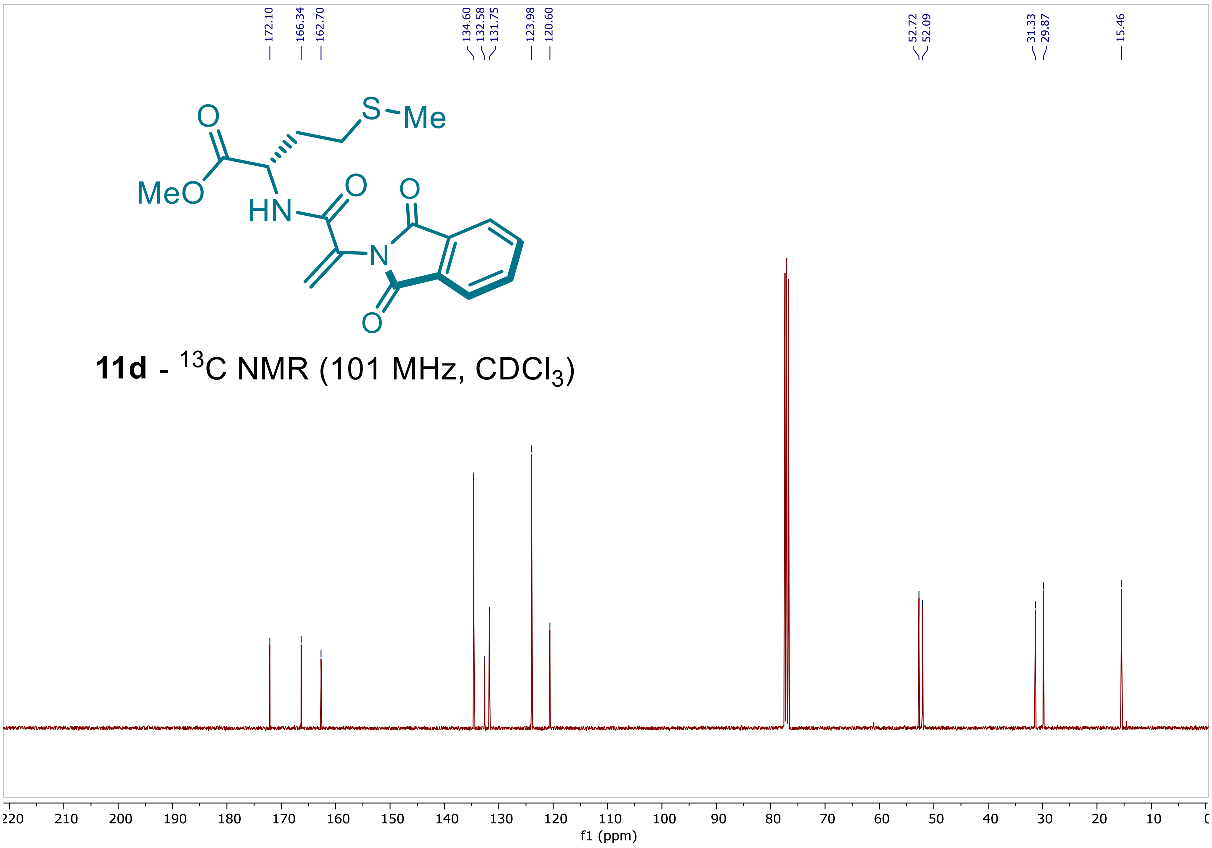
172.10 166.34 162.70
134.60 132.58 131.75 123.98 120.60
52.72 52.09
31.33 29.87
15.46
O
S
Me
MeO
HN
O
O
N
O
11d - ^{13}C NMR (101 MHz, CDCl$_3$)
220 210 200 190 180 170 160 150 140 130 120 110 100 90 80 70 60 50 40 30 20 10 0
f1 (ppm)

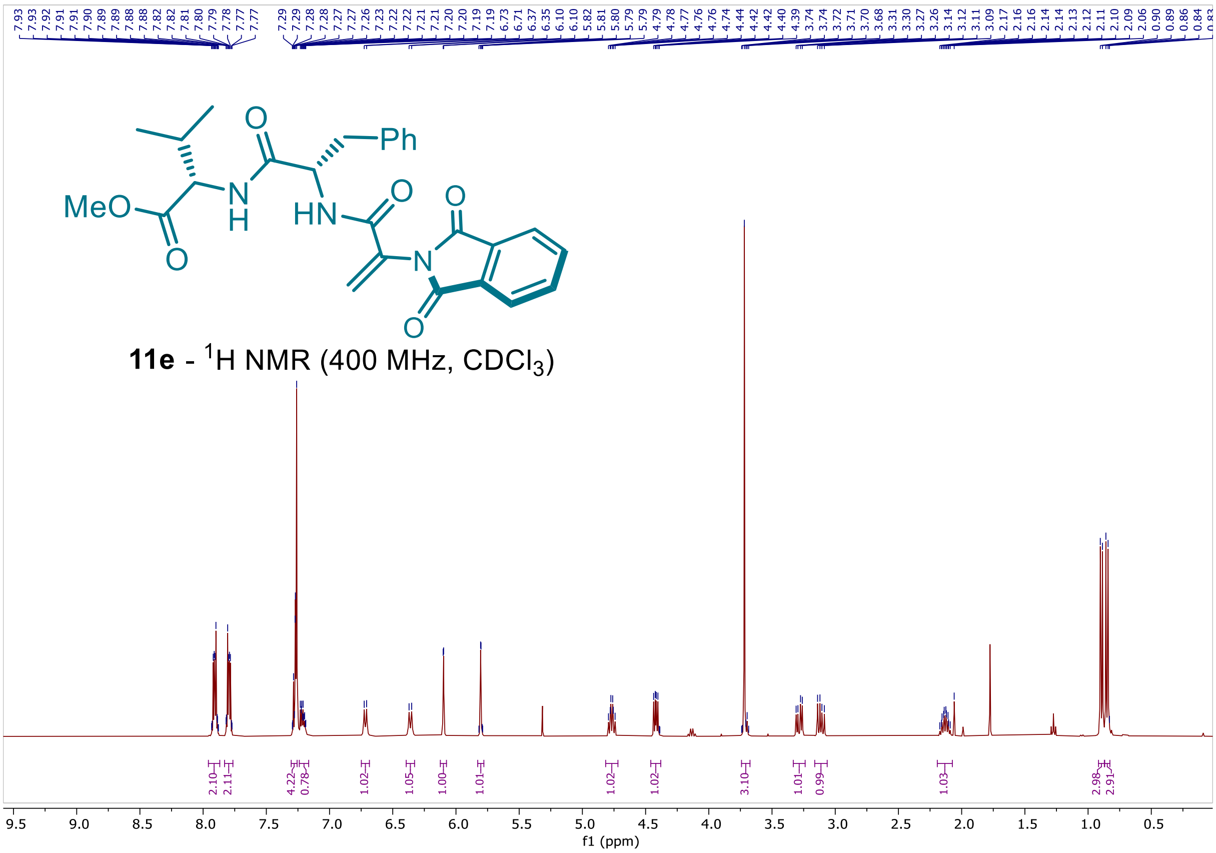
MeO
N
H
HN
N
Ph
O
O
O
O
O
11e - 1H NMR (400 MHz, CDCl3)
f1 (ppm)

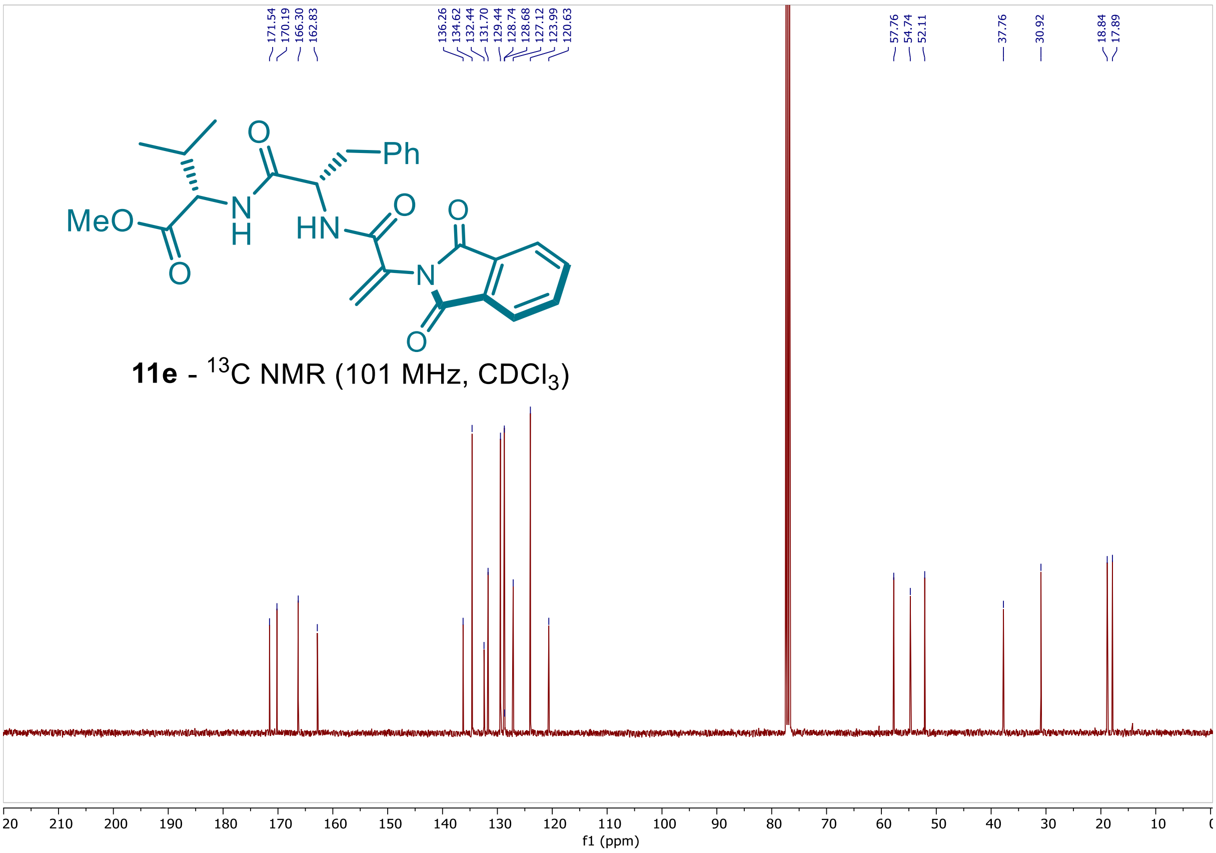
MeO
N
H
HN
N
Ph
O
O
O
O
O
11e - 13C NMR (101 MHz, CDCl3)
f1 (ppm)

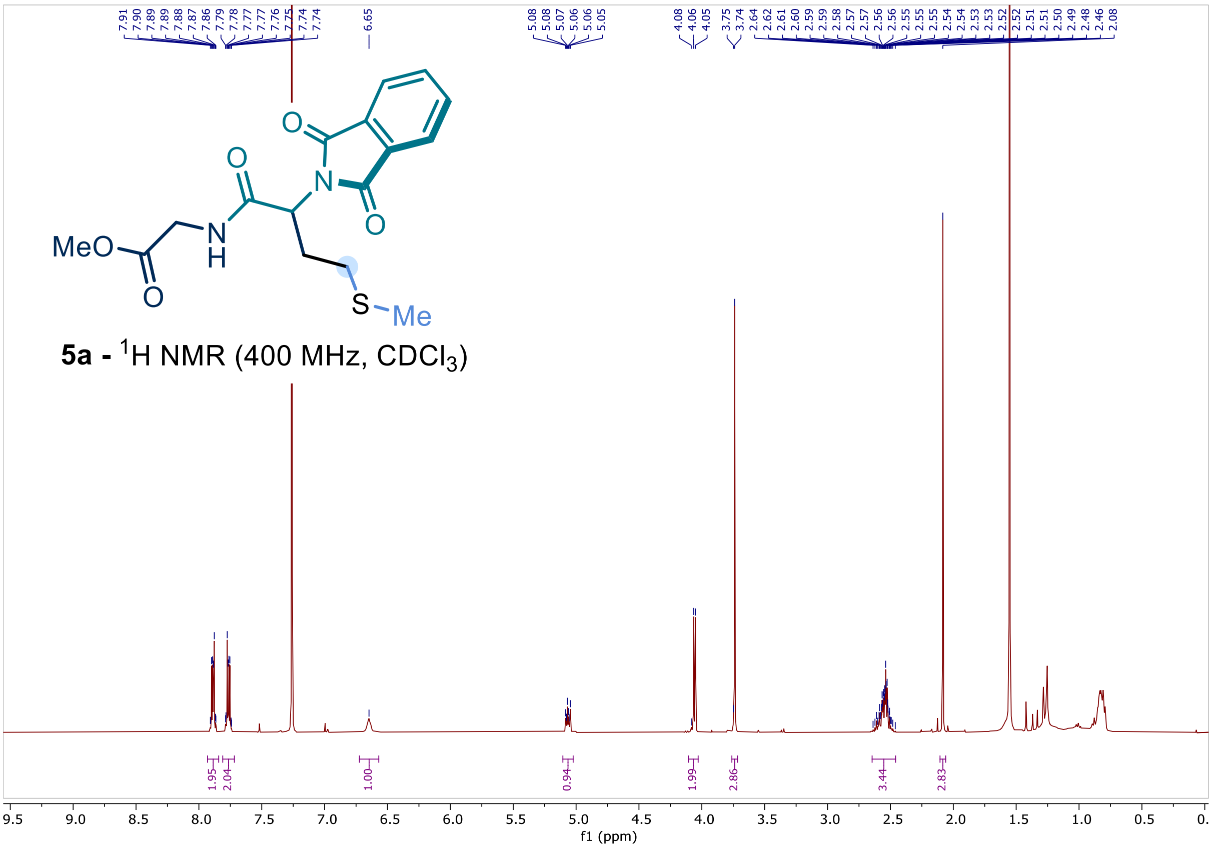

5a - ^{1}H NMR (400 MHz, $CDCl_3$)

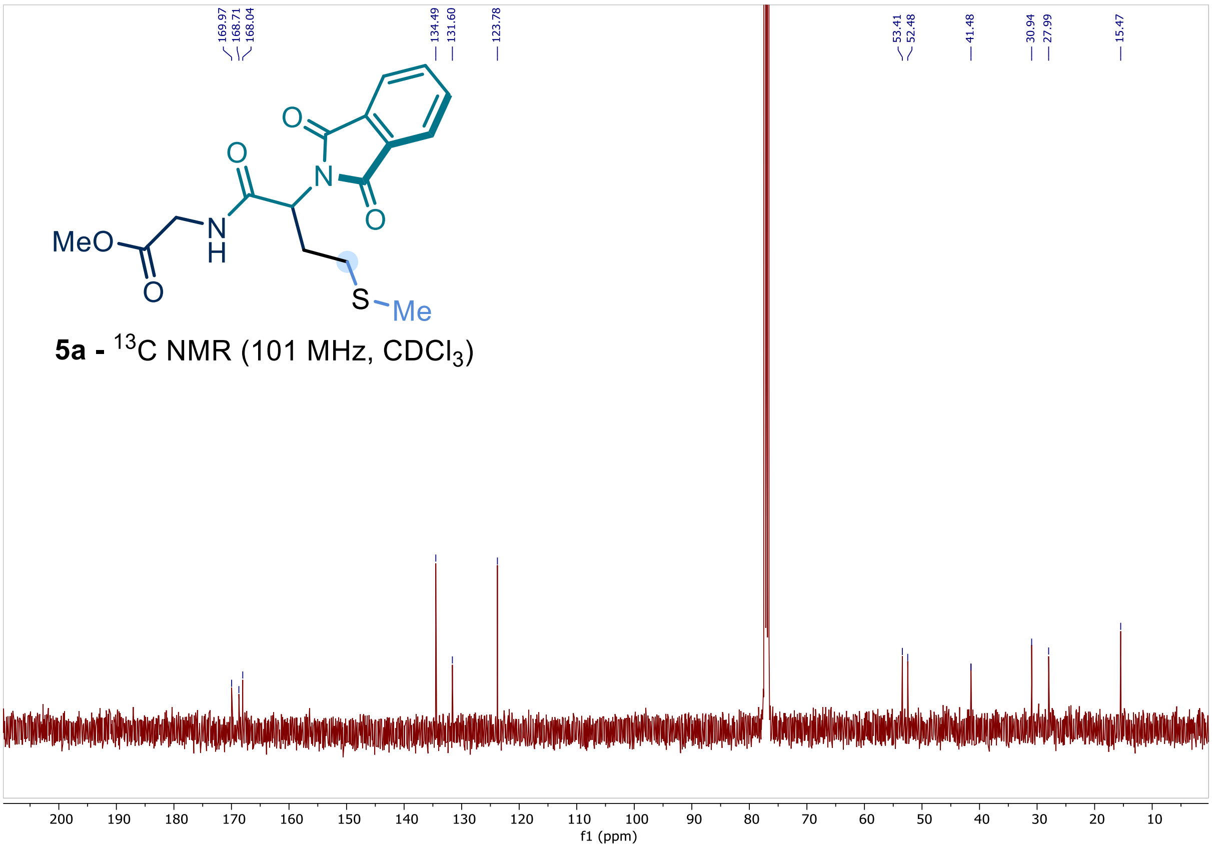

5a - ^{13}C NMR (101 MHz, $CDCl_3$)

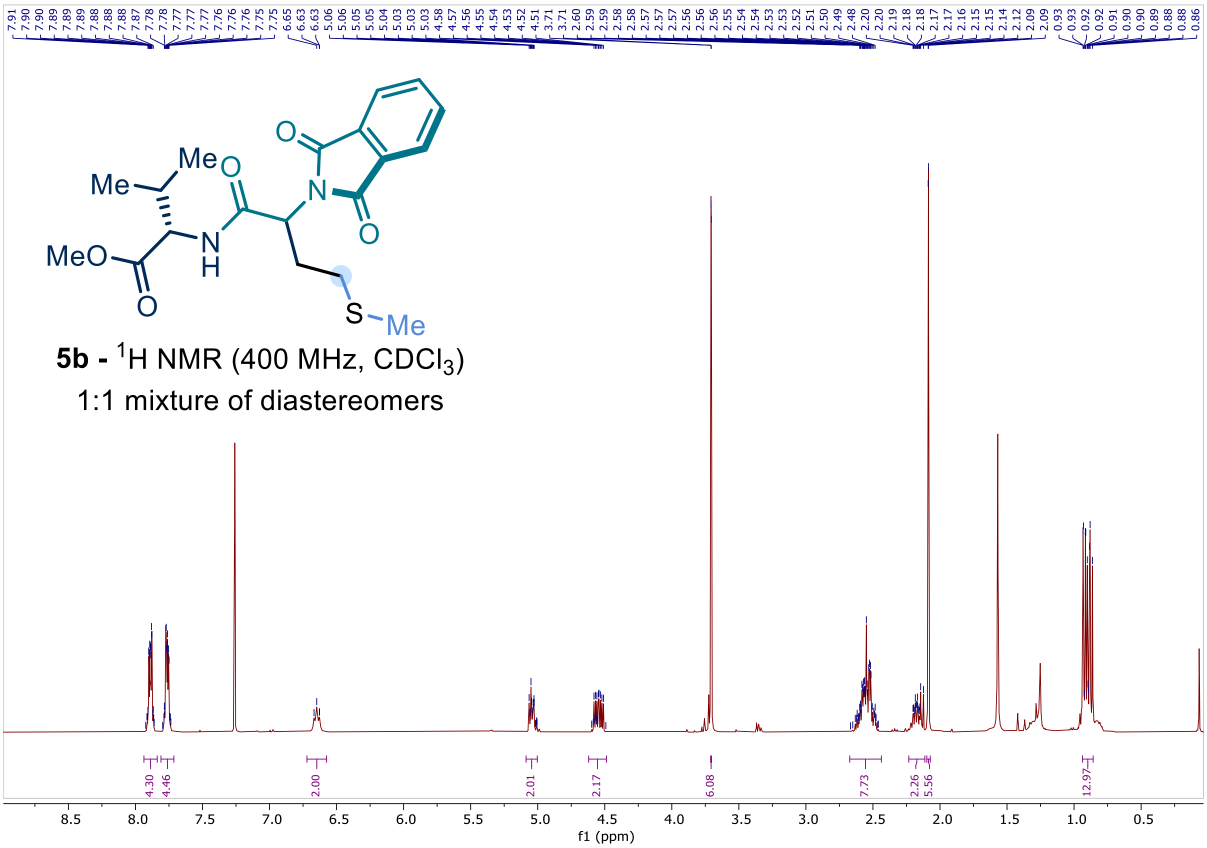

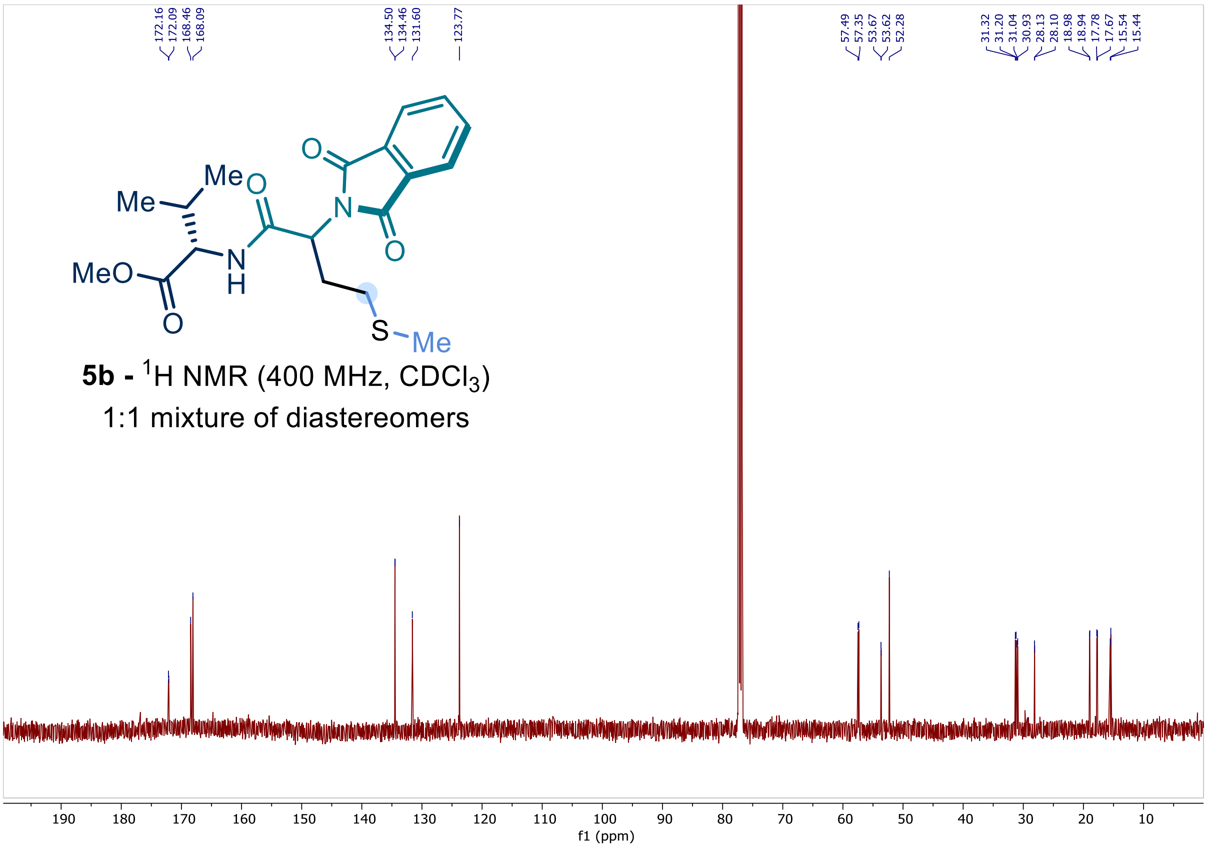

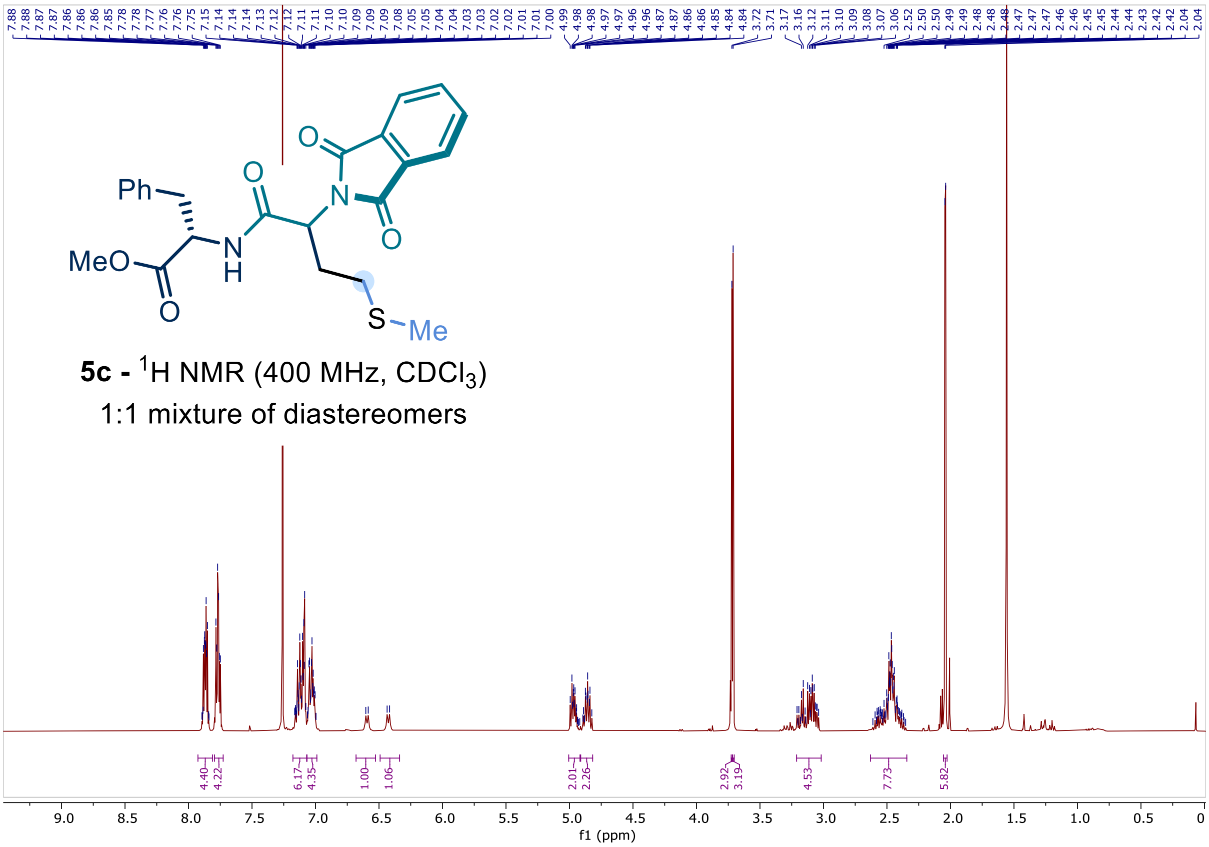
Ph
MeO
O
N
H
O
O
N
O
S
Me
5c - 1H NMR (400 MHz, CDCl3)
1:1 mixture of diastereomers
f1 (ppm)

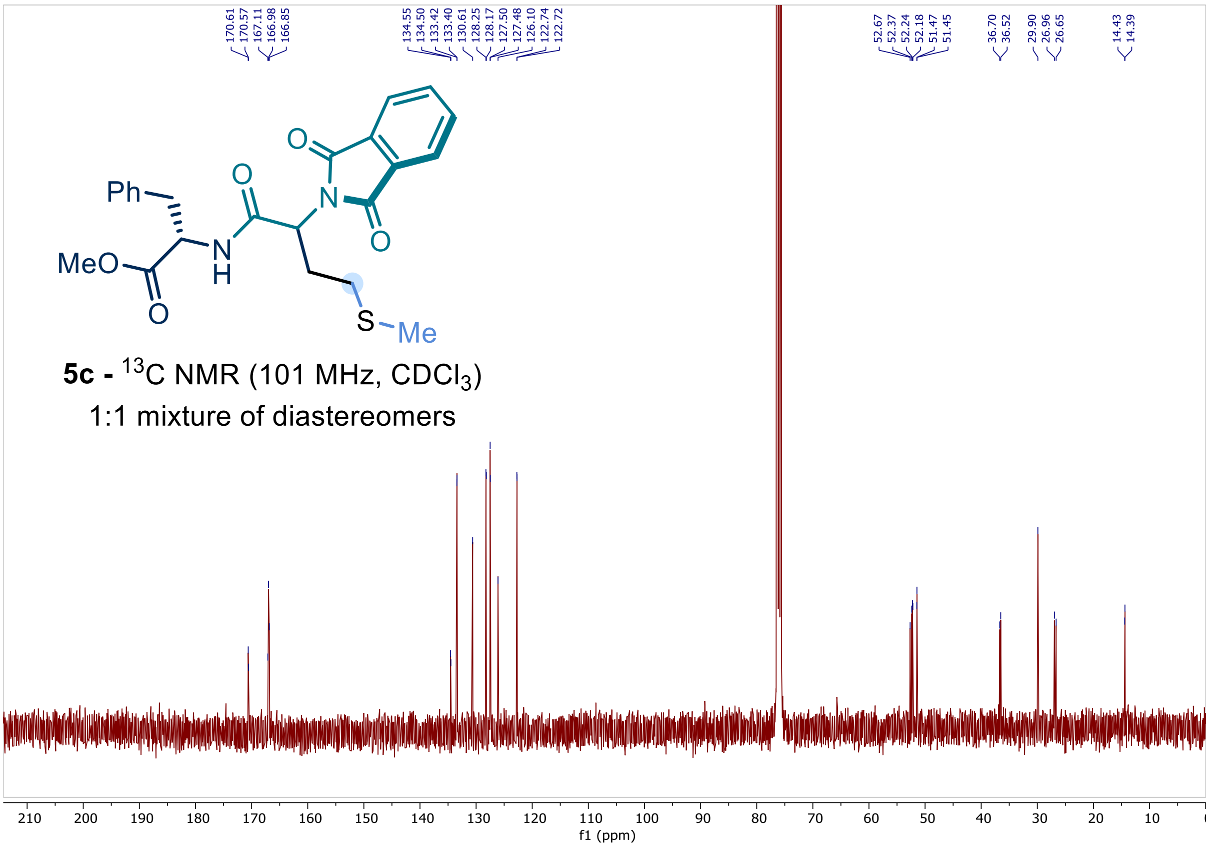
Ph
MeO
O
N
H
O
O
N
O
S
Me
5c - 13C NMR (101 MHz, CDCl3)
1:1 mixture of diastereomers
170.61
170.57
167.11
166.98
166.85
134.55
134.50
133.42
133.40
130.61
128.25
128.17
127.50
127.48
126.10
122.74
122.72
52.67
52.37
52.24
52.18
51.47
51.45
36.70
36.52
29.90
26.96
26.65
14.43
14.39
f1 (ppm)

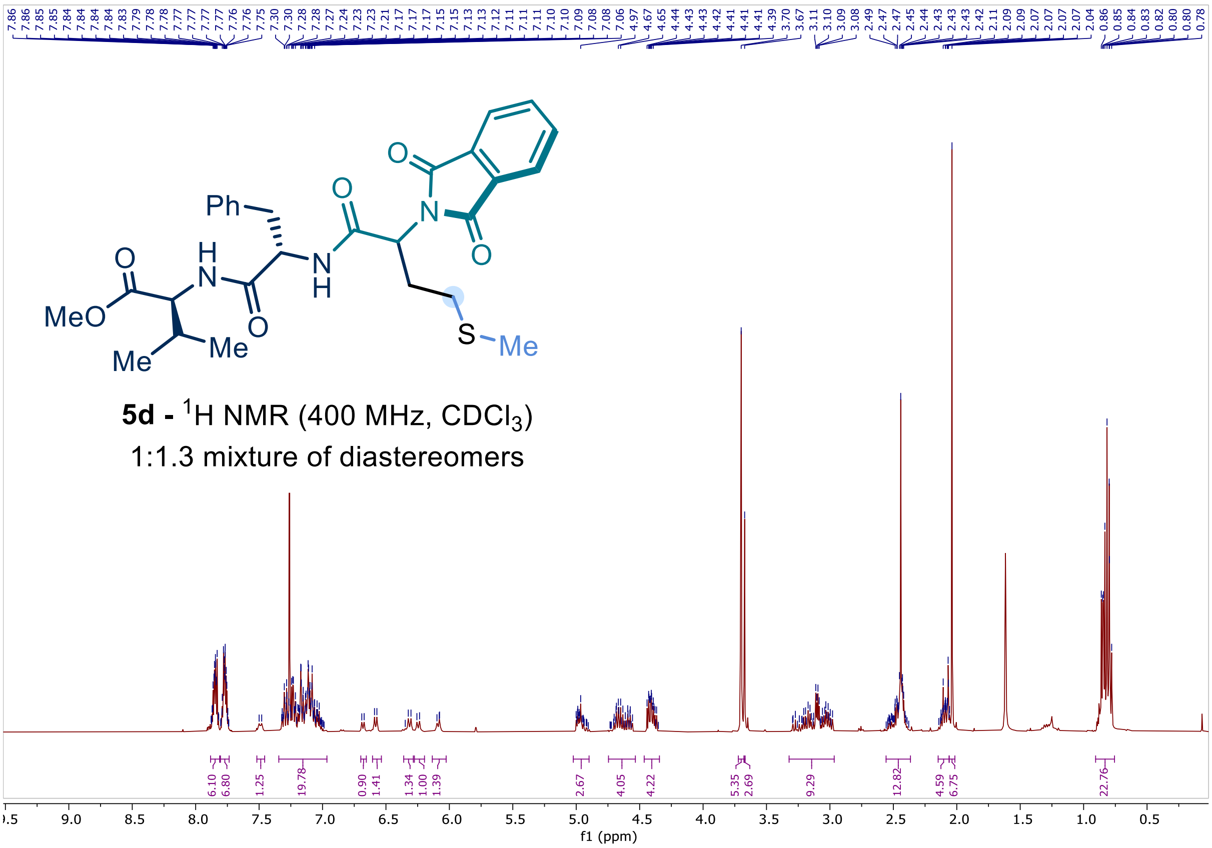
5d - ¹H NMR (400 MHz, CDCl₃)
1:1.3 mixture of diastereomers

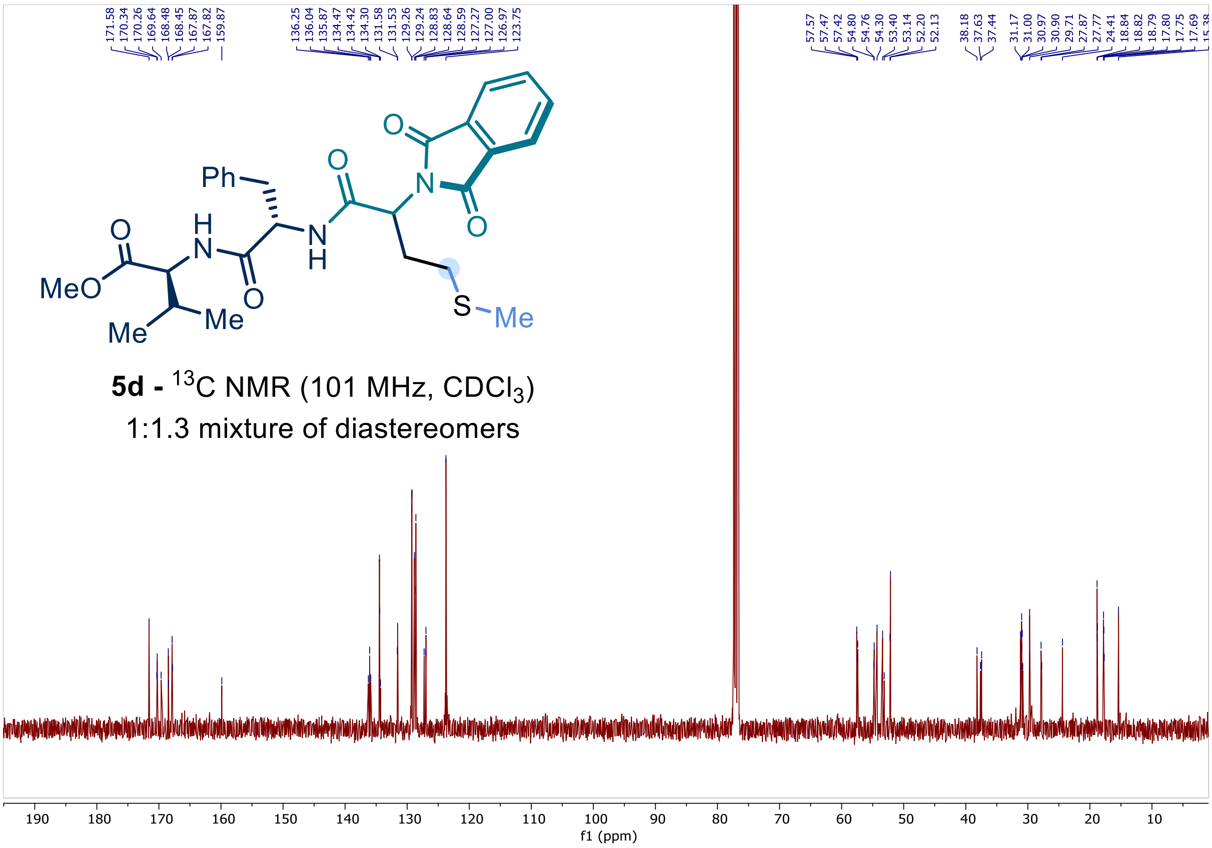
5d - ¹³C NMR (101 MHz, CDCl₃)
1:1.3 mixture of diastereomers

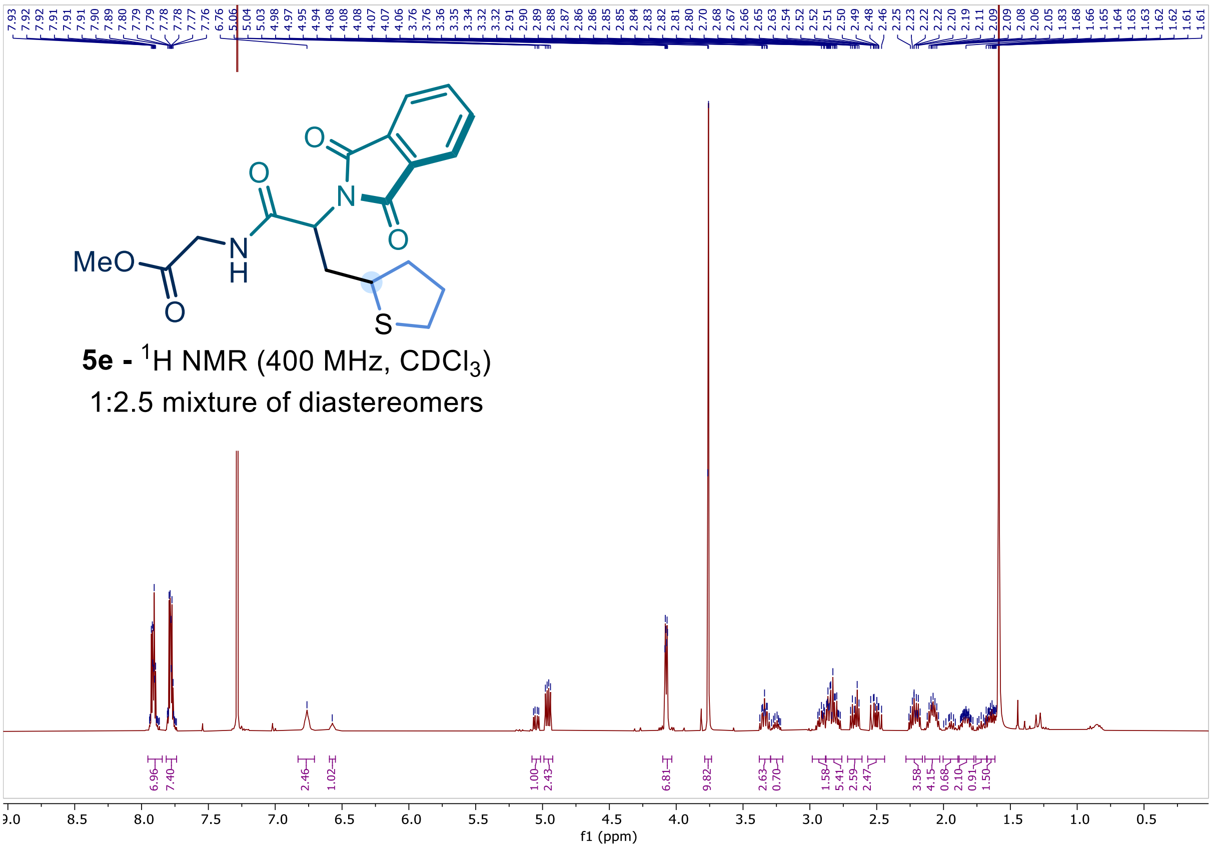
7.93 7.92 7.91 7.91 7.90 7.89 7.80 7.79 7.79 7.78 7.78 7.77 7.76 6.76 5.06 5.04 5.03 4.98 4.97 4.95 4.94 4.08 4.08 4.08 4.07 4.07 4.06 3.76 3.76 3.36 3.35 3.34 3.32 3.32 2.91 2.90 2.89 2.88 2.87 2.86 2.86 2.85 2.85 2.84 2.83 2.82 2.81 2.80 2.70 2.68 2.67 2.66 2.65 2.63 2.54 2.52 2.52 2.51 2.50 2.49 2.48 2.46 2.25 2.23 2.22 2.22 2.20 2.19 2.11 2.09 2.09 2.08 2.06 2.05 1.83 1.68 1.66 1.65 1.64 1.63 1.63 1.62 1.61 1.61
MeO
N
H
O
S
5e - 1H NMR (400 MHz, CDCl3)
1:2.5 mixture of diastereomers
6.96 7.40 2.46 1.02 1.00 2.43 6.81 9.82 2.63 0.70 1.58 5.41 2.59 2.47 3.58 4.15 0.68 2.10 0.91 1.50
9.0 8.5 8.0 7.5 7.0 6.5 6.0 5.5 5.0 4.5 4.0 3.5 3.0 2.5 2.0 1.5 1.0 0.5
f1 (ppm)

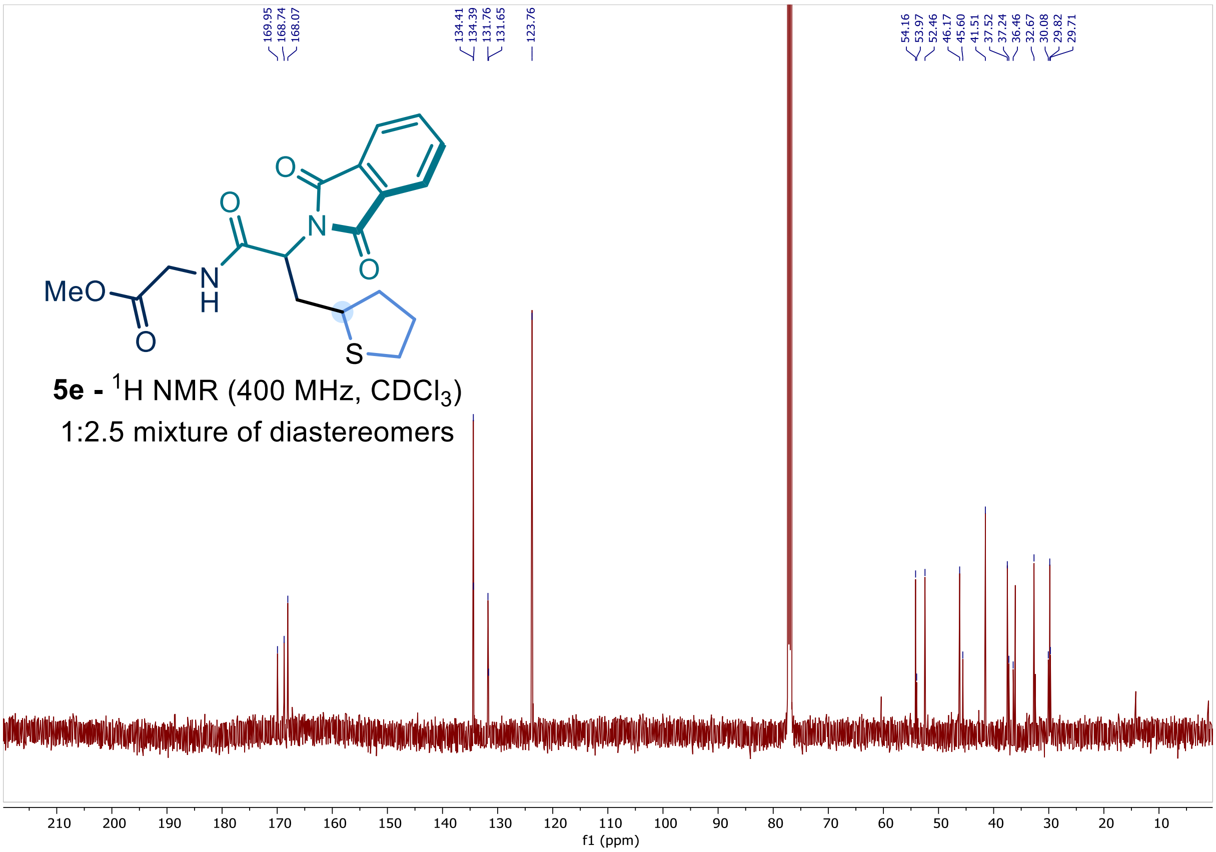
169.95 168.74 168.07
134.41 134.39 131.76 131.65
123.76
54.16 53.97 52.46 46.17 45.60 41.51 37.52 37.24 36.46 32.67 30.08 29.82 29.71
MeO
N
H
O
S
5e - 1H NMR (400 MHz, CDCl3)
1:2.5 mixture of diastereomers
210 200 190 180 170 160 150 140 130 120 110 100 90 80 70 60 50 40 30 20 10
f1 (ppm)

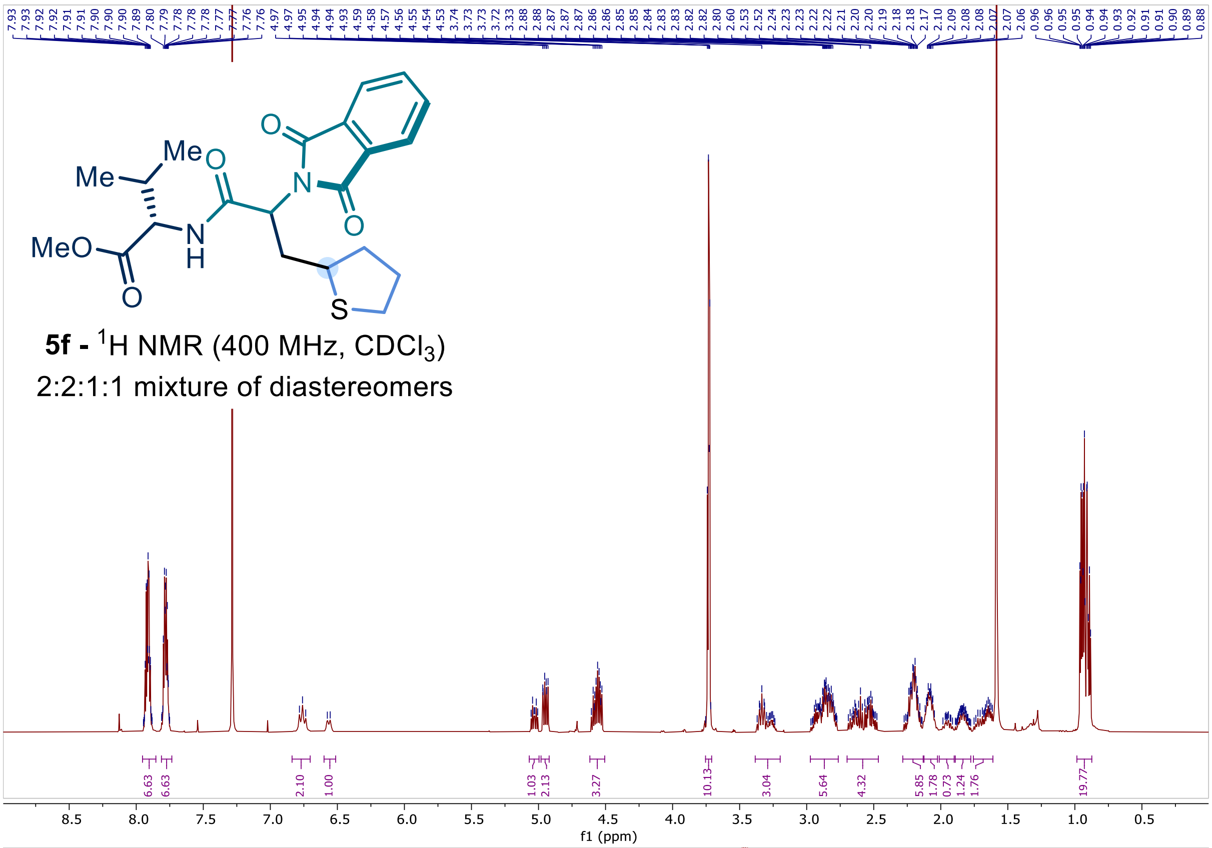

Me
Me
O
O
N
O
MeO
N
H
O
S
5f - ^{1}H NMR (400 MHz, $CDCl_3$)
2:2:1:1 mixture of diastereomers
f1 (ppm)

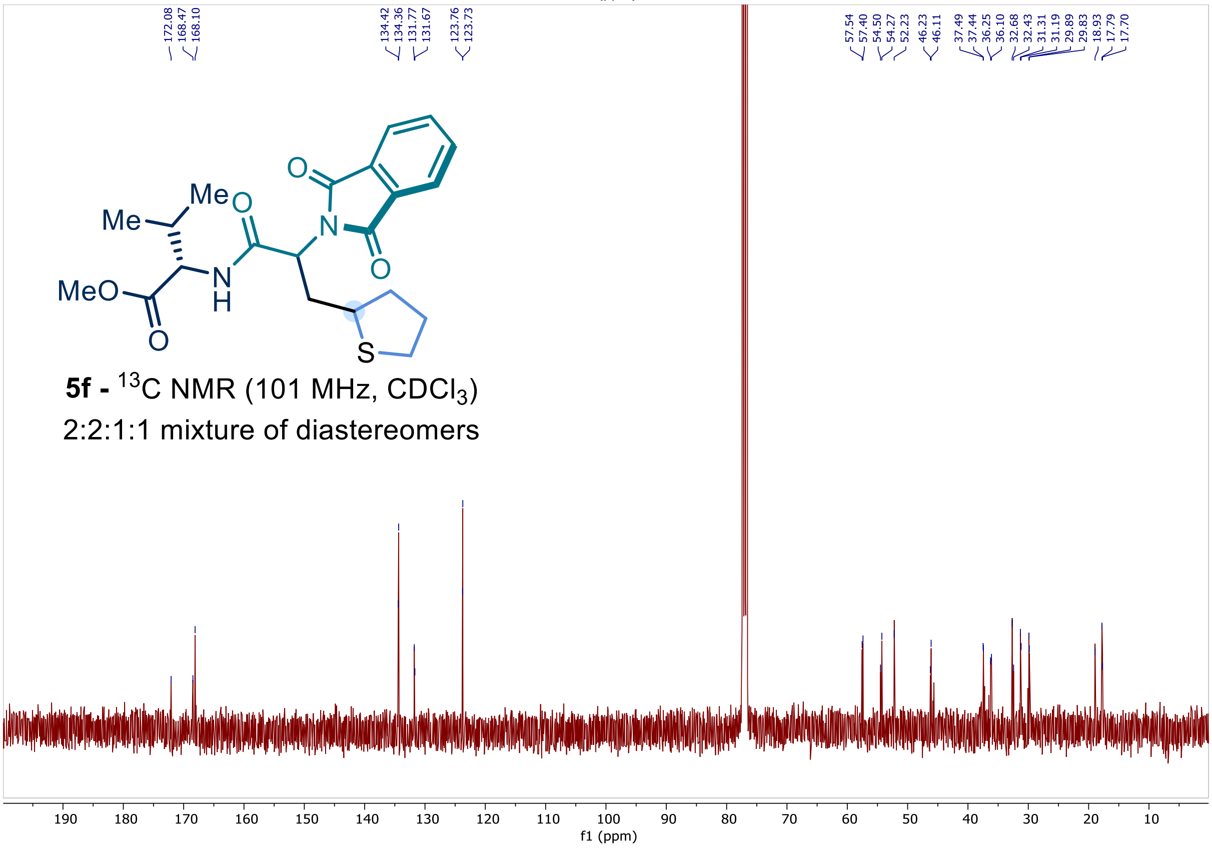

Me
Me
O
O
N
O
MeO
N
H
O
S
5f - ^{13}C NMR (101 MHz, $CDCl_3$)
2:2:1:1 mixture of diastereomers
f1 (ppm)

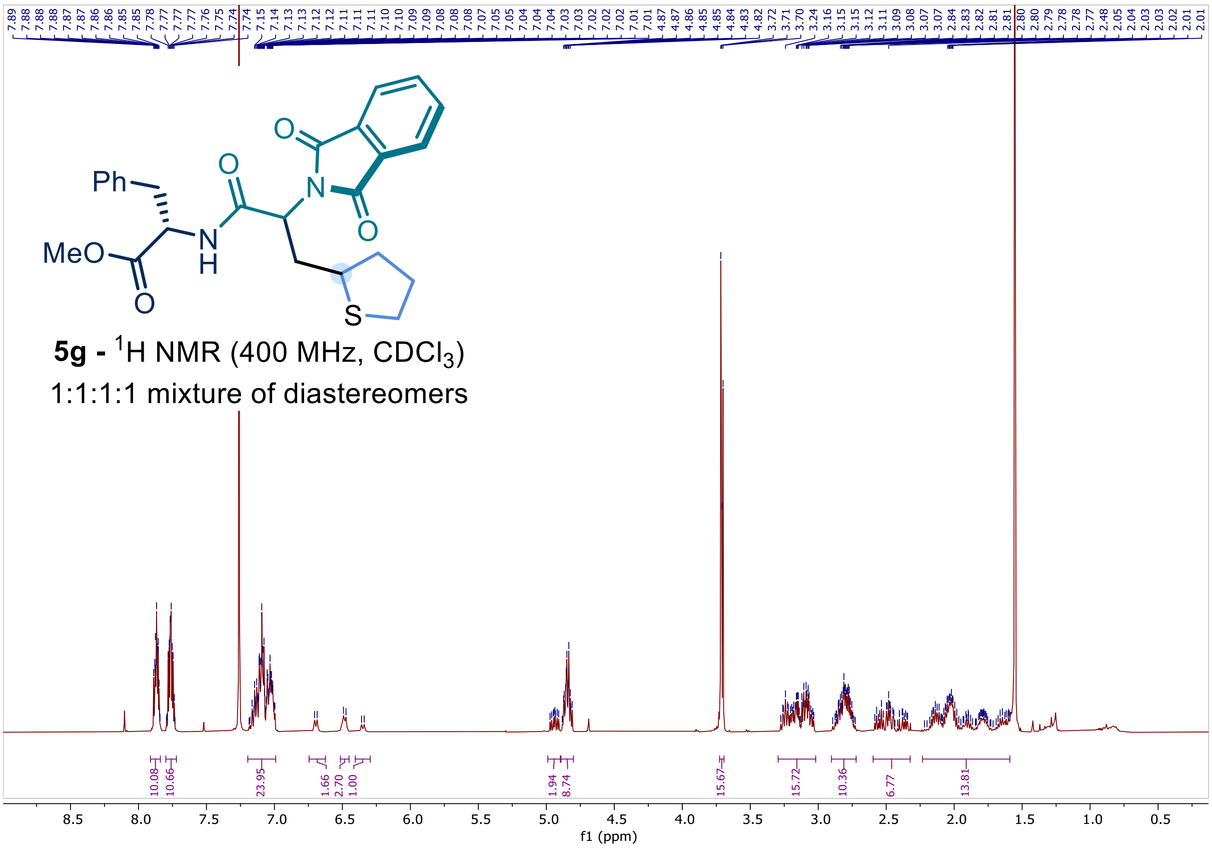
Ph
MeO
N
H
O
N
S
5g - ^{1}H NMR (400 MHz, $CDCl_3$)
1:1:1:1 mixture of diastereomers
f1 (ppm)

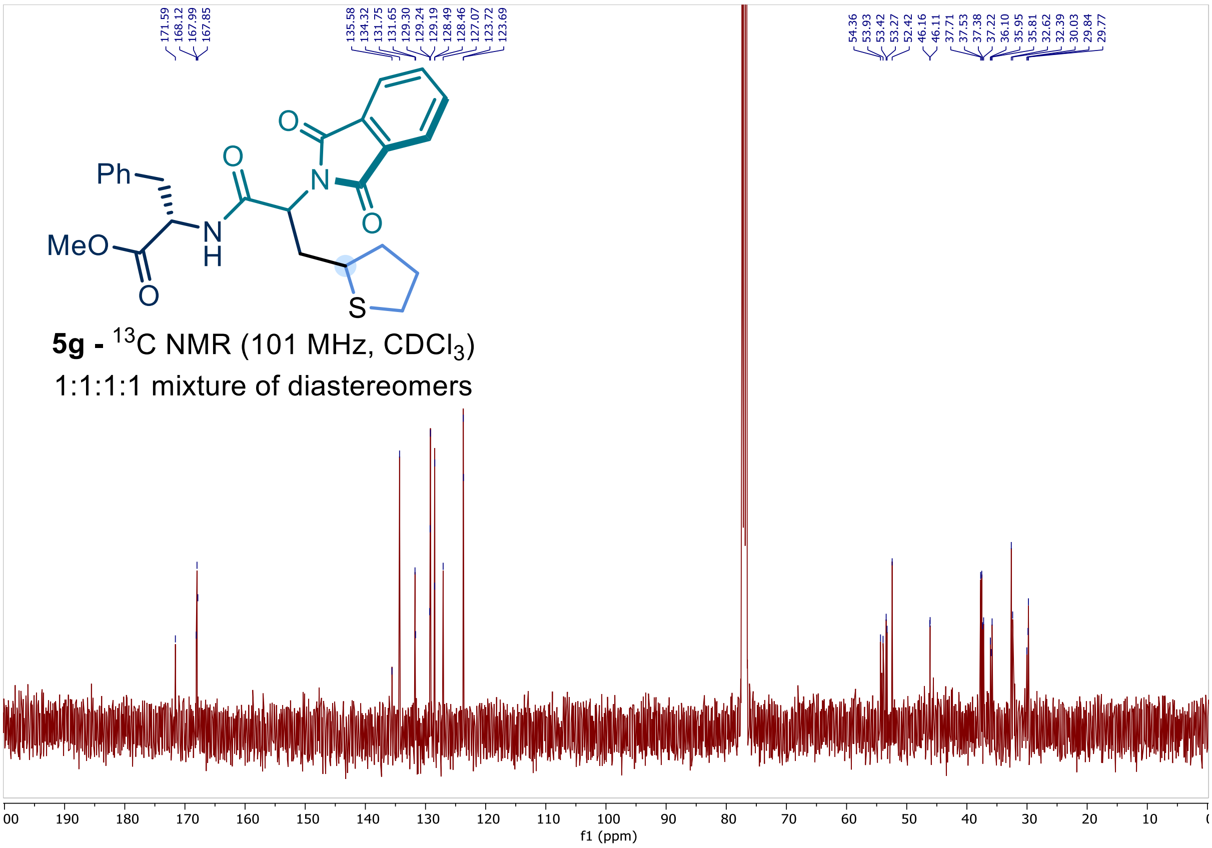
Ph
MeO
N
H
O
N
S
5g - ^{13}C NMR (101 MHz, $CDCl_3$)
1:1:1:1 mixture of diastereomers
171.59
168.12
167.99
167.85
135.58
134.32
131.75
131.65
129.30
129.24
129.19
128.49
128.46
127.07
123.72
123.69
54.36
53.93
53.42
53.27
52.42
46.16
46.11
37.71
37.53
37.38
37.22
36.10
35.95
35.81
32.62
32.39
30.03
29.84
29.77
f1 (ppm)

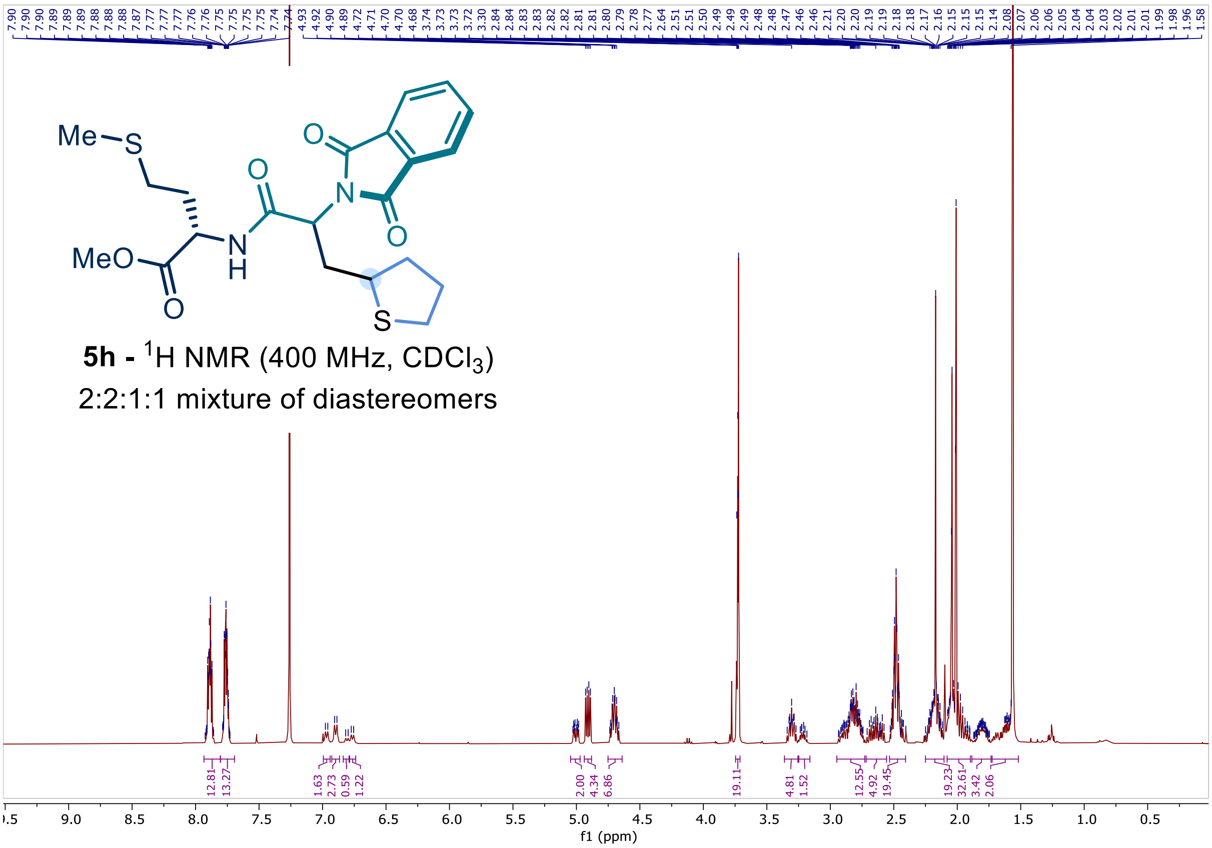
Me
S
MeO
O
N
H
O
N
O
O
S
5h - 1H NMR (400 MHz, CDCl3)
2:2:1:1 mixture of diastereomers
f1 (ppm)

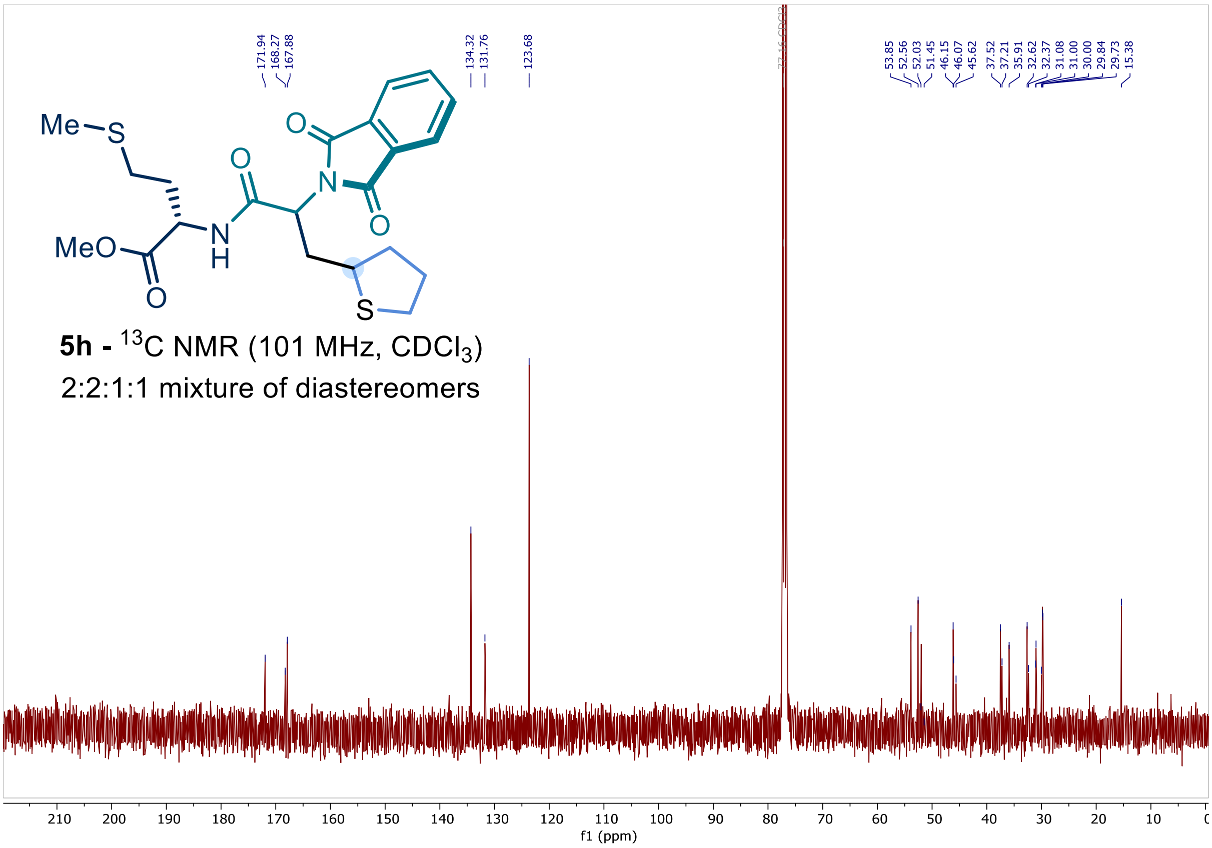
171.94
168.27
167.88
134.32
131.76
123.68
53.85
52.56
52.03
51.45
46.15
46.07
45.62
37.52
37.21
35.91
32.62
32.37
31.08
31.00
30.00
29.84
29.73
15.38
5h - 13C NMR (101 MHz, CDCl3)
2:2:1:1 mixture of diastereomers
f1 (ppm)

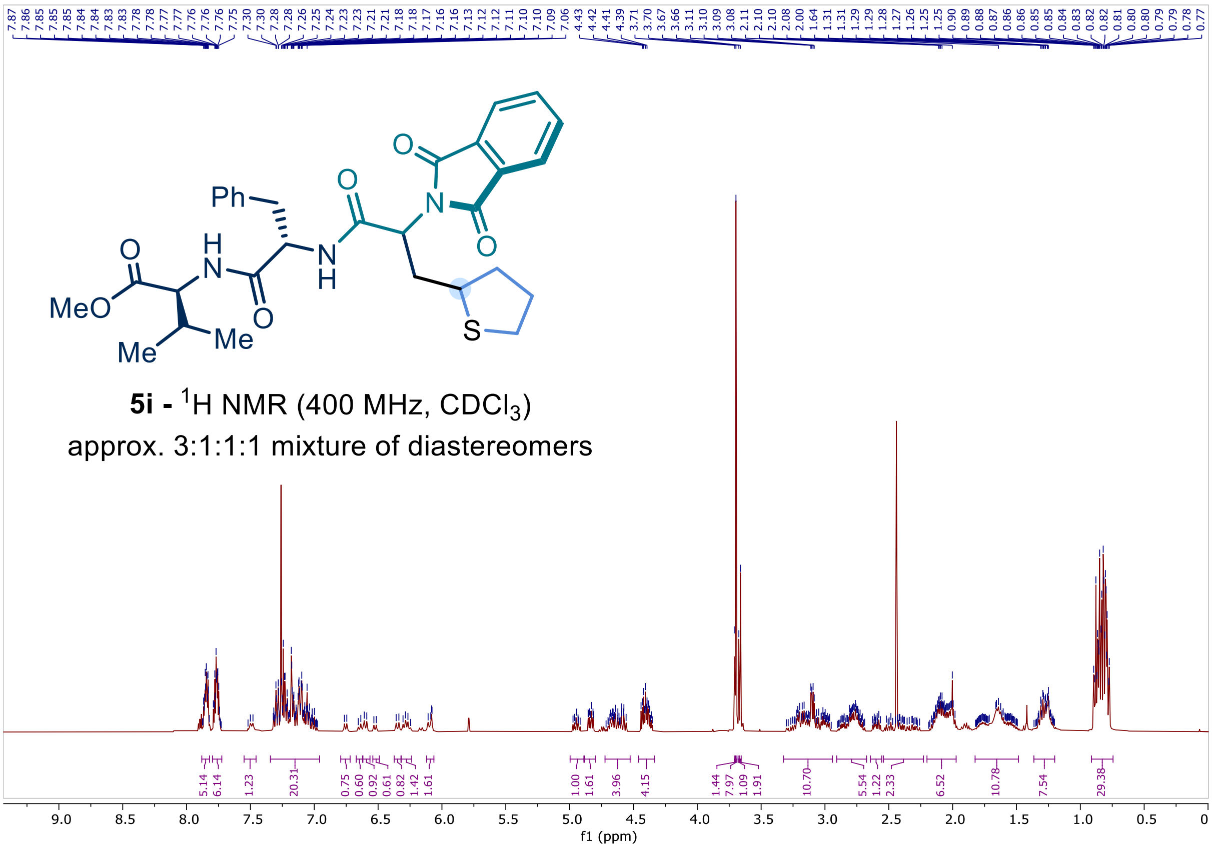
Ph
MeO
Me
Me
5i - ¹H NMR (400 MHz, CDCl₃)
approx. 3:1:1:1 mixture of diastereomers
f1 (ppm)

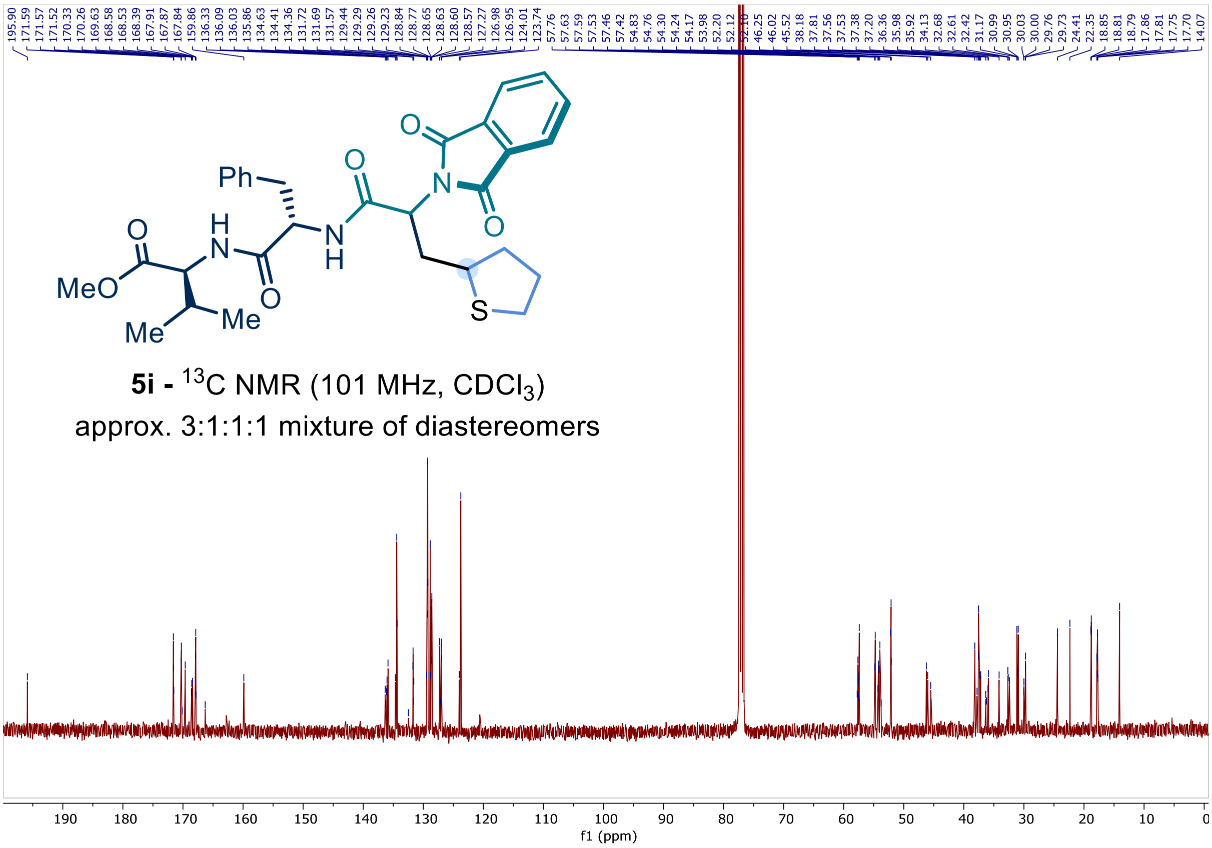
Ph
MeO
Me
Me
5i - ¹³C NMR (101 MHz, CDCl₃)
approx. 3:1:1:1 mixture of diastereomers
f1 (ppm)

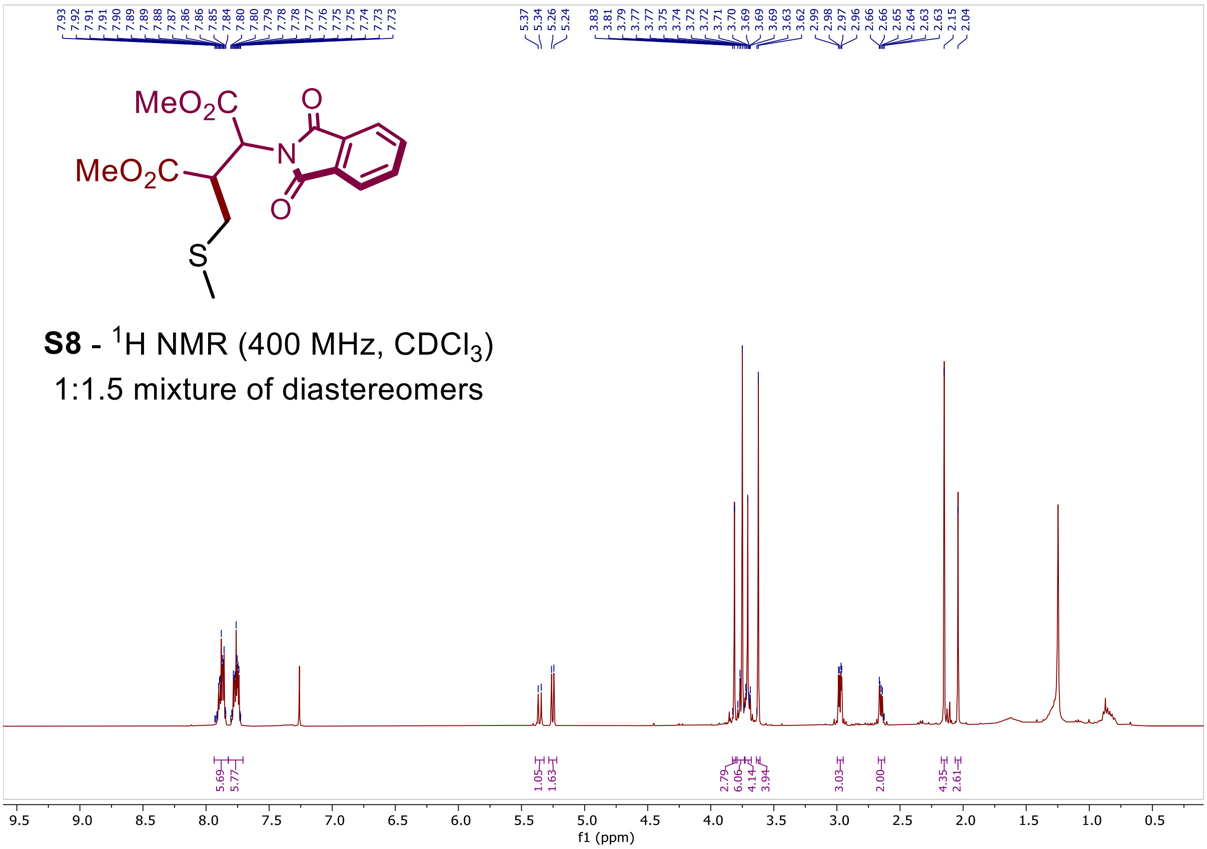
S8 - 1H NMR (400 MHz, CDCl3)
1:1.5 mixture of diastereomers
f1 (ppm)

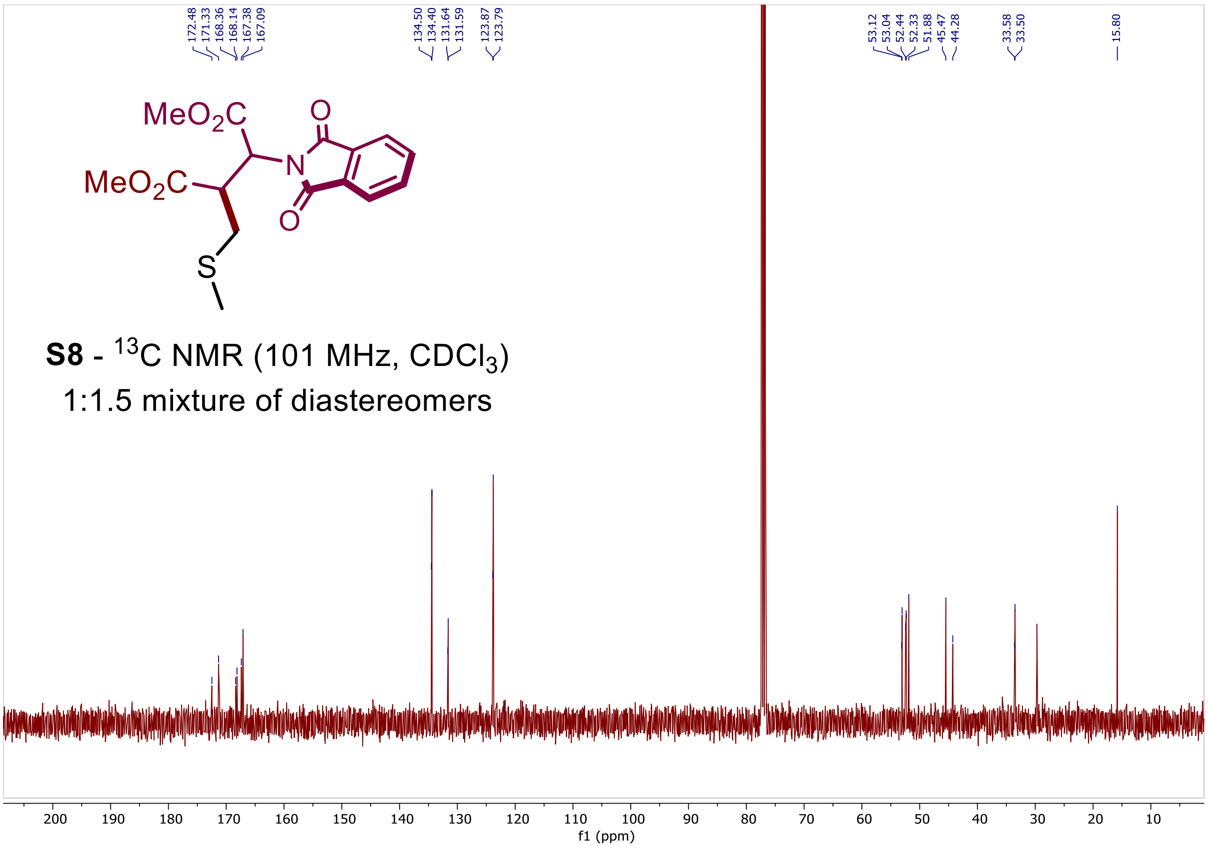
S8 - 13C NMR (101 MHz, CDCl3)
1:1.5 mixture of diastereomers
f1 (ppm)

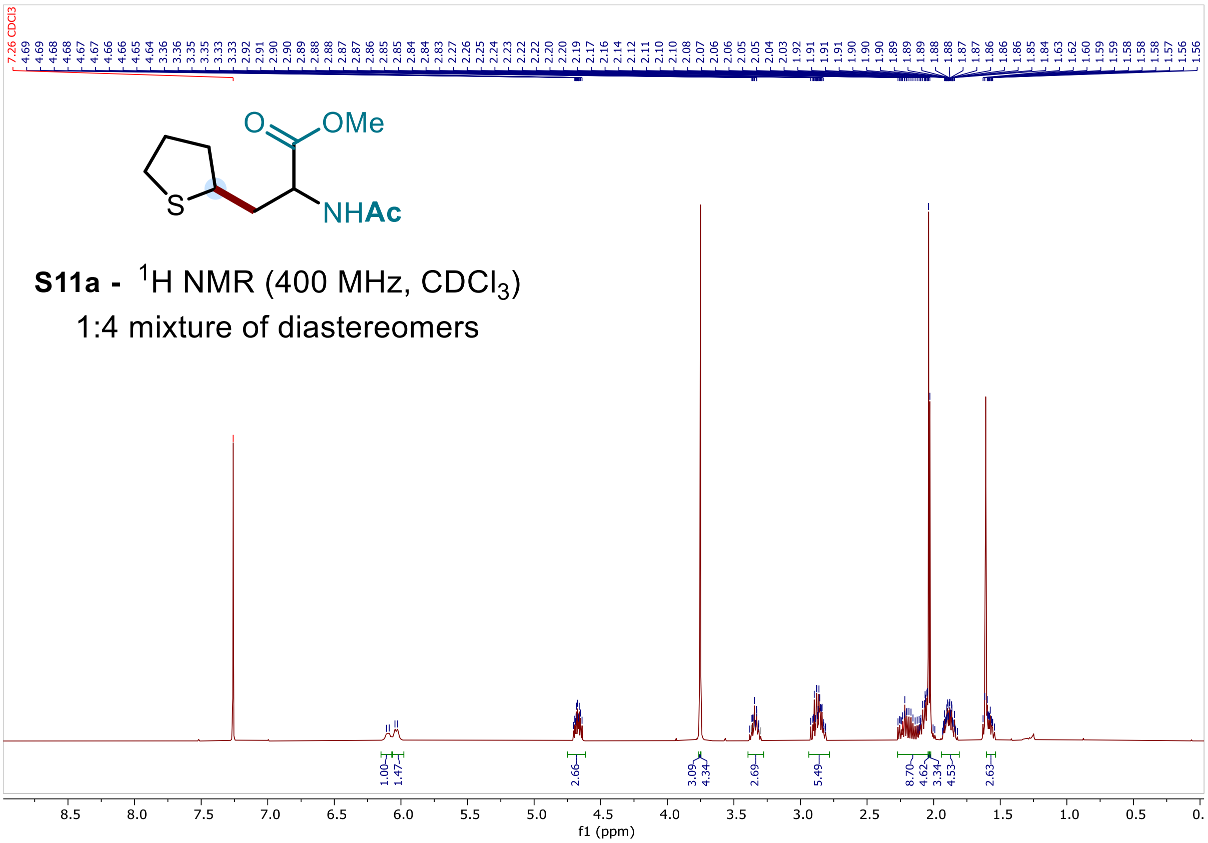
OMe
NHAc
S
S11a - ^{1}H NMR (400 MHz, $CDCl_3$)
1:4 mixture of diastereomers
f1 (ppm)

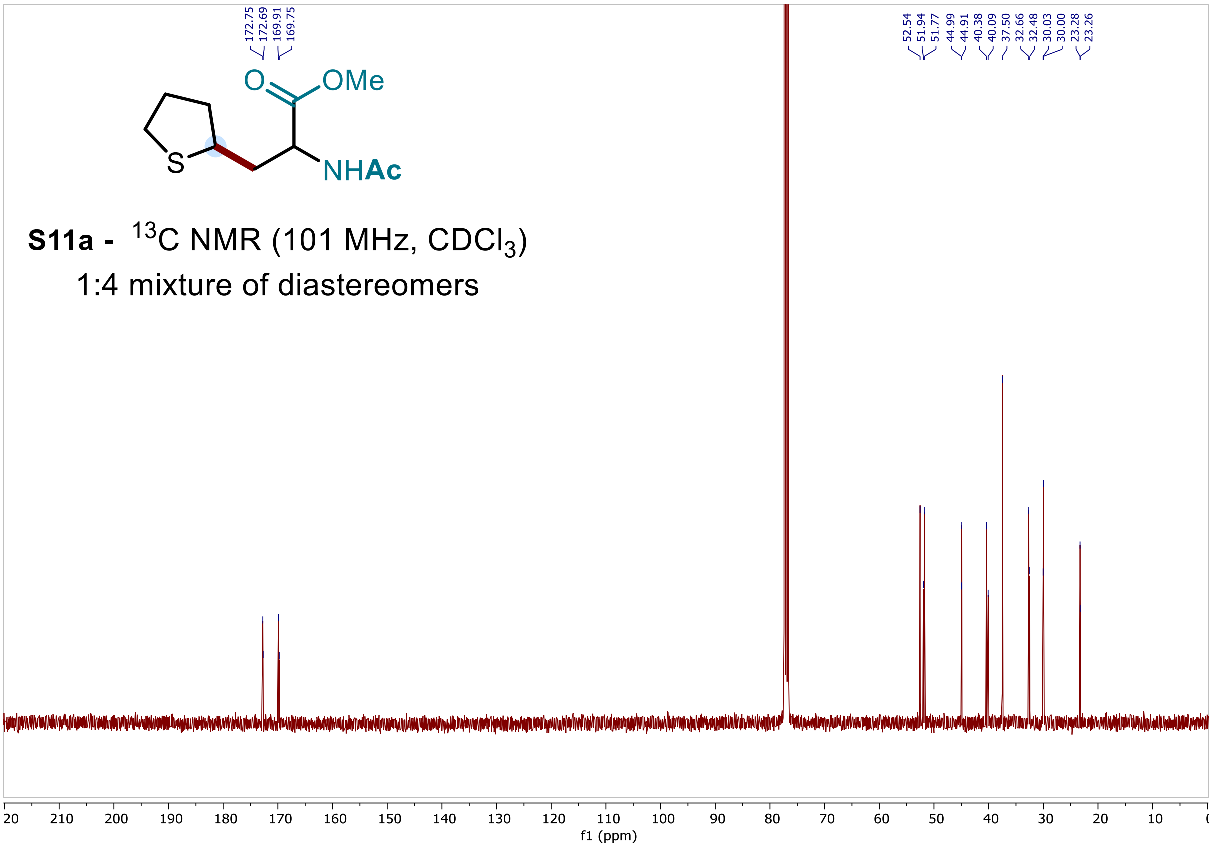
OMe
NHAc
S
S11a - ^{13}C NMR (101 MHz, $CDCl_3$)
1:4 mixture of diastereomers
f1 (ppm)

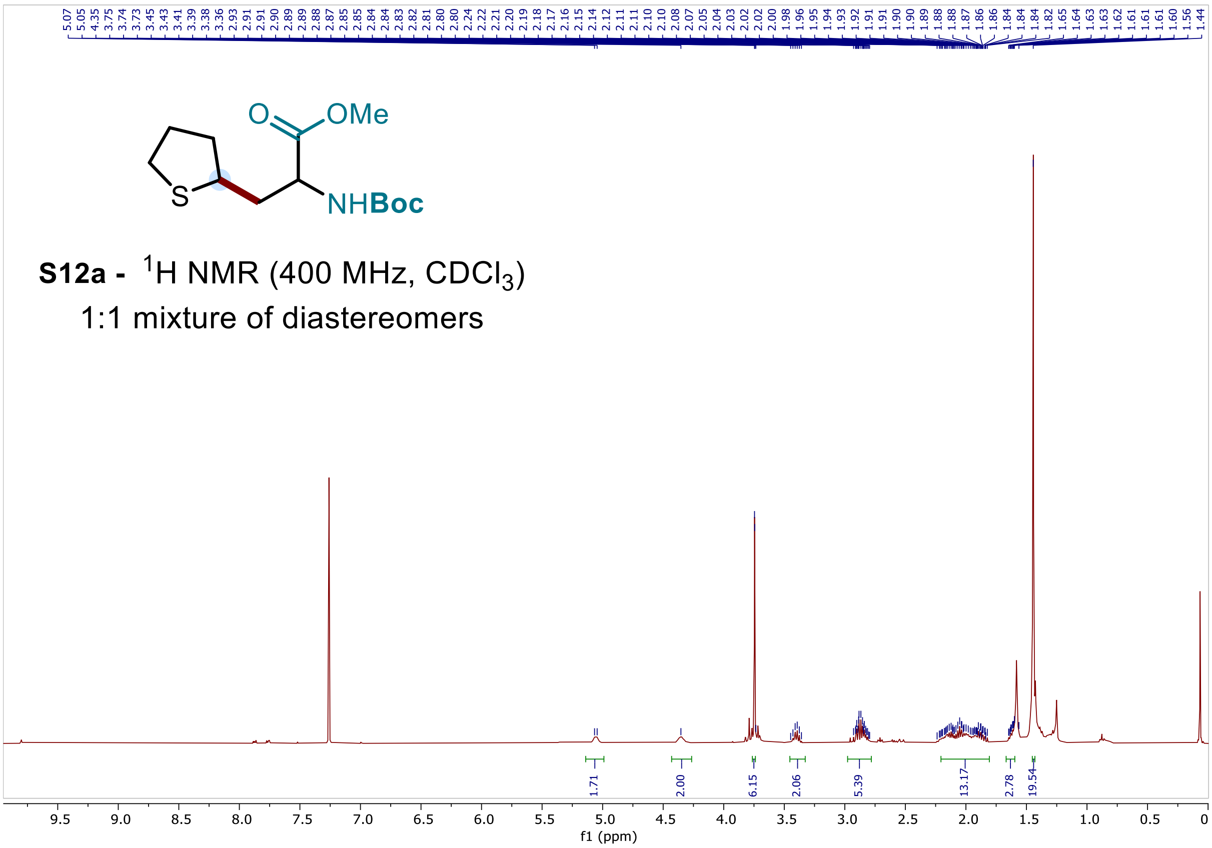

O
OMe
S
NHBoc
S12a - ^{1}H NMR (400 MHz, $CDCl_3$)
1:1 mixture of diastereomers
f1 (ppm)

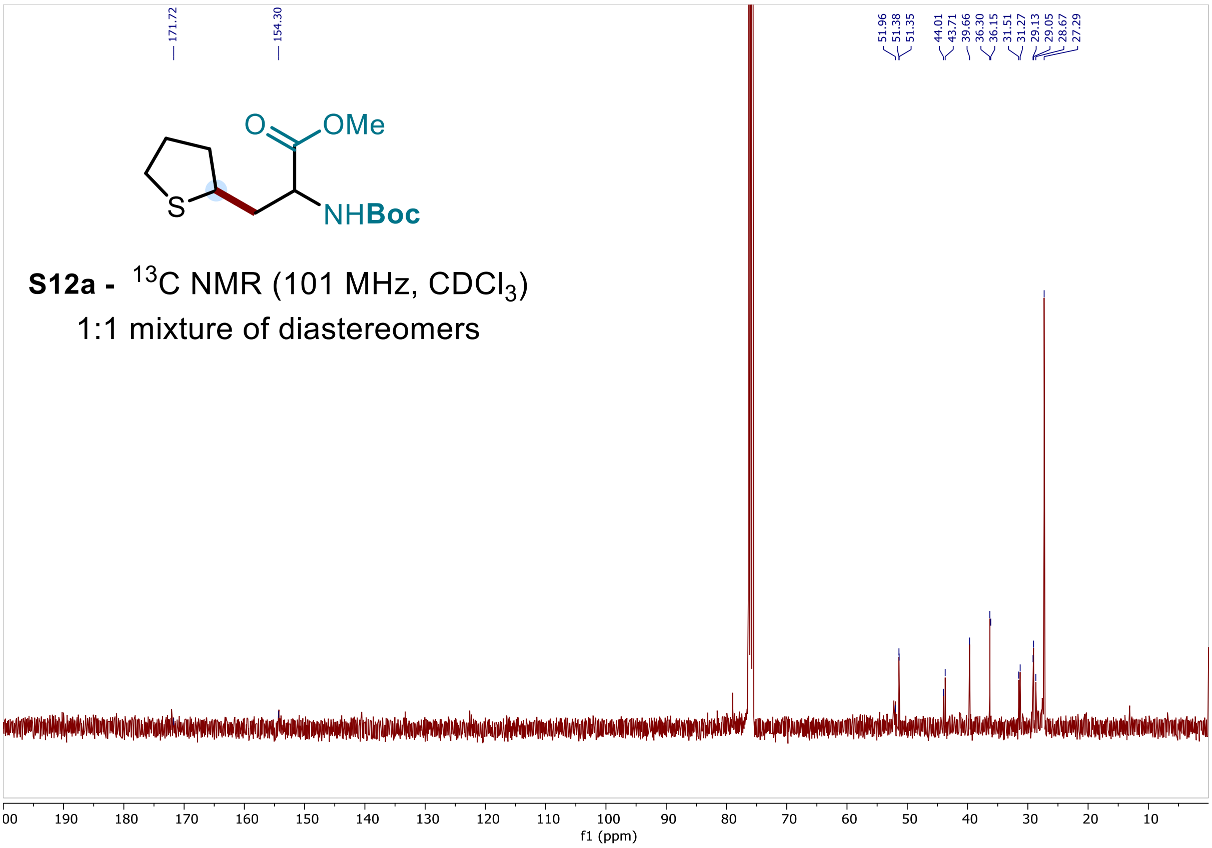

O
OMe
S
NHBoc
S12a - ^{13}C NMR (101 MHz, $CDCl_3$)
1:1 mixture of diastereomers
f1 (ppm)

Computational Analysis

Computational Methods

All calculations were performed using Gaussian 16 revision A03.[13] Each step was modelled using relaxed potential energy scans (SI Figures SX1, SX4 and SX5; reaction coordinates are defined in each Figure legend) performed using the B3LYP functional,[14–16] the 6-31+G(d,p) basis sets on all atoms, Grimme's empirical dispersion correction[17,18] and polarizable continuum solvation[19] to model water solvation (ε=80). For the excited-state H-transfer step, time-dependent density functional (TDDFT) single point calculations solving for 10 states were performed on structures taken from the potential energy scans, using both B3LYP and the M06-2X hybrid functional[20] (which has been shown to perform well for charge transfer calculations[21]. and for triplet energies and singlet-to-triplet transitions[22–24]). Spin-orbit couplings were computed using PySOC[25] at the B3LYP/6-31+G(d,p) level of theory.

Supplementary Figures

Defined reactants

THT:

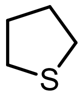

FMN:

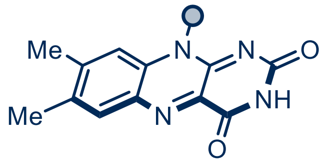

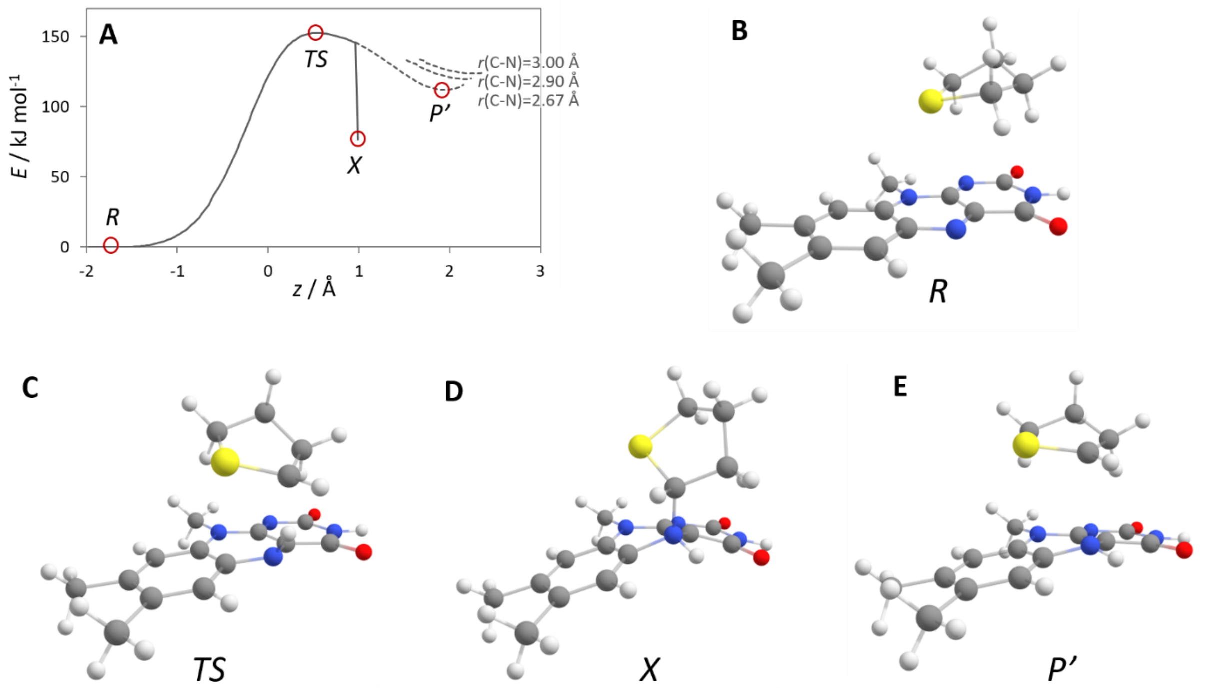

Figure S10. Ground state potential energy scan for H-transfer (PCET) from THT to FMN (A), and structures along the scan (B-E). The scan was not completed on the product side as the two high-energy fragments spontaneously combine via C-N bond formation (structure X), but nevertheless the ground state reaction is very endothermic: keeping the C-N distance fixed to the value at the transition state produces a product (*P’*) with and energy 112 kJ mol^{-1}, and increasing the C-N distance increases this energy further. The reaction coordinate for the scan was defined as the difference between the breaking and forming bonds: z = [r(C_D-H) – r(N_A-H)] where C_D and N_A are the donor carbon and acceptor nitrogen atoms, respectively and H is the transferring atom.

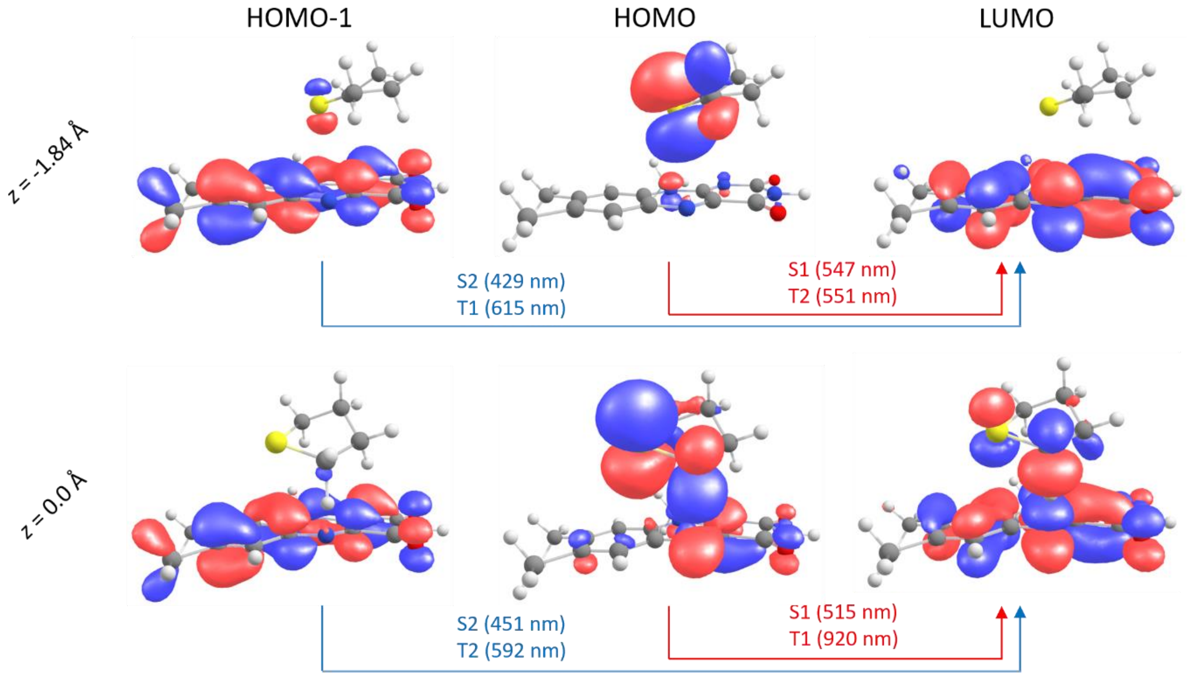

Figure S11. Molecular orbitals for the major electronic transitions corresponding to the S1, T1, S2 and T2 states calculated using TDDFT with B3LYP/6-31+G(d,p), for the reactant structure (z = -1.84 Å) and the structure with transferring H equidistant from the donor C and acceptor N atoms (z = 0.0 Å). eT from THT to FMN corresponds to transition from the highest occupied molecular orbital (HOMO) to the lowest unoccupied molecular orbital (LUMO) which are localised on the THT and Flavin, respectively, in the reactant structure.

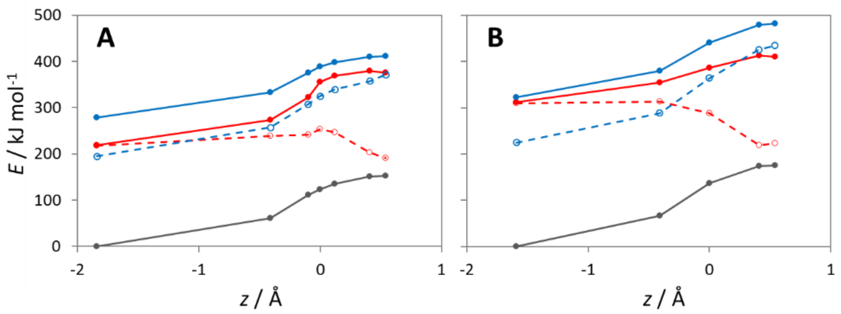

Figure S12. Computed energies of structures along H-transfer scans for PCET from THT to FMN using B3LYP (**A**) and M06-2X (**B**). Dark grey: energies on the ground state surface; red: energies on the FMN*/THT surface; blue: energies on the FMN^-/THT^+ surface; singlet surfaces are shown as solid lines, triplet surfaces as dotted lines.

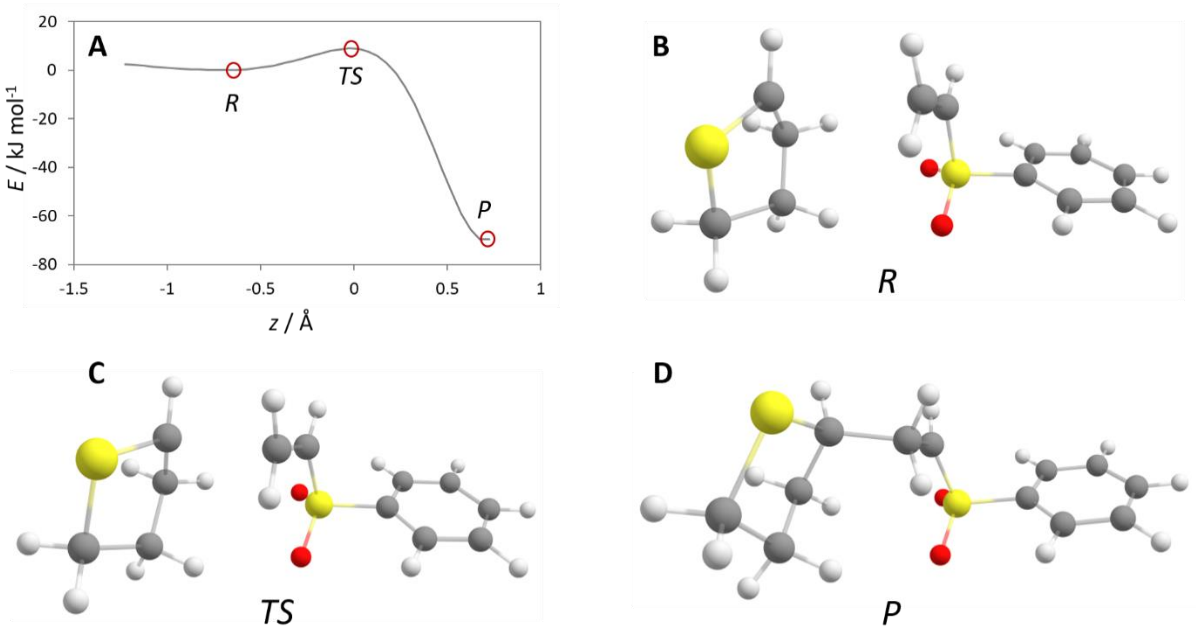

Figure S13. Potential energy scan for C-C bond formation between sulfide radical **III** and alkene **2 (R)**, to form intermediate **V** (P) and structures along the scan (**B**-**D**). A relaxed scan of the C-C bond was performed, but reaction coordinate in **A** is defined as the difference between the C-C distance for the forming bond, r(C-C), and the average value in the (R) and product (P) structures: z = [r(C-C)$_R$ + r(C-C)$_P$]/2 – r(C-C).

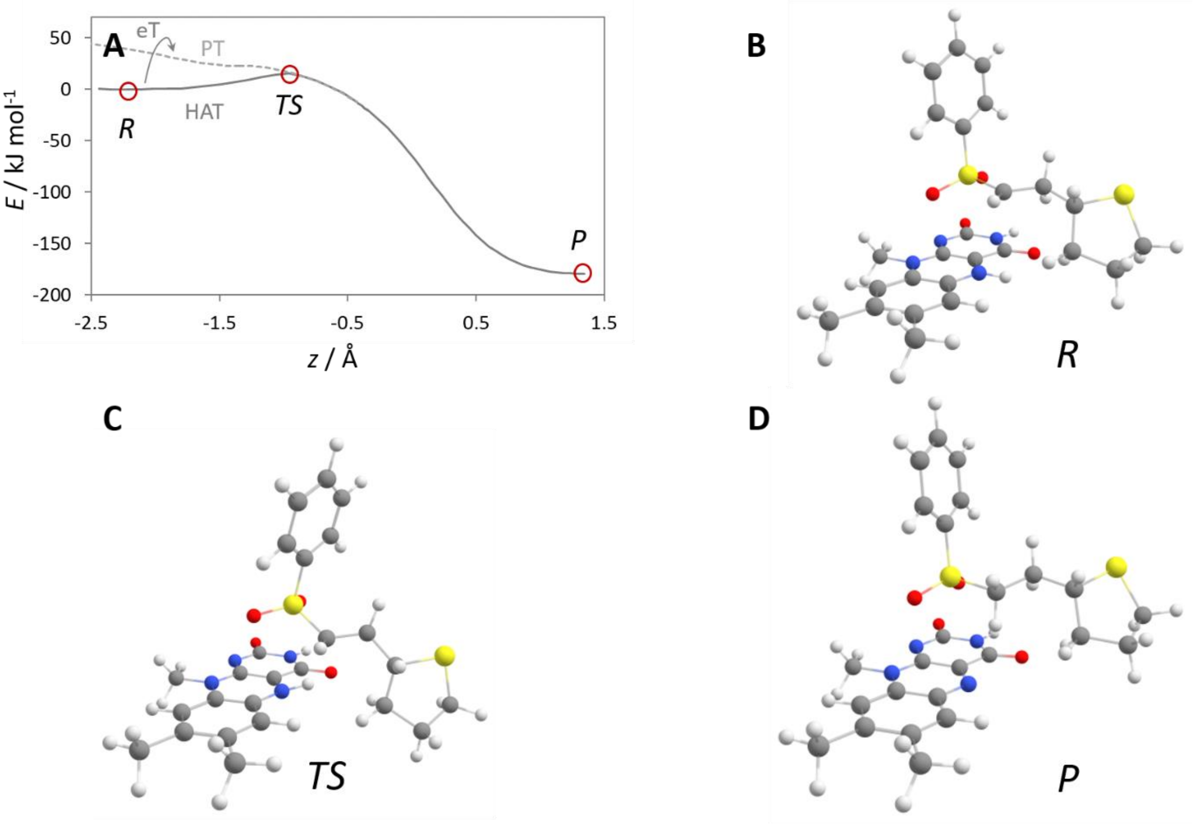

Figure S14. Potential energy scans for H-transfer between Flavin-H· species **IV** and intermediate **V** (**A**), by either eT-PT (light grey, dotted line) or hydrogen atom transfer, HAT (dark grey, solid line), and structures along the HAT scan (**B**-**D**). The reactant state (*R*) is a diradical, as confirmed by the Mulliken spin densities of +/-0.985 on **IV** and **V**. The reaction coordinate for the scans was defined as the difference between the breaking and forming bonds: $z = [r(C_D\text{-}H) – r(N_A\text{-}H)]$ where C_D and N_A are the donor carbon and acceptor nitrogen atoms, respectively and H is the transferring atom.

Table S1. Ground (S0) and excited state energies on the singlet and triplet RF*/1a (excited Flavin) and II/I (reduced Flavin) electronic surfaces, computed at the B3LYP/6-31+G(d,p) and M06-2X/6-31+G(d,p) levels of theory.

		E_{rel} / kJ mol^{-1}			
z / Å	S0	Singlet RF*/1a	Triplet RF*/1a	Singlet	Triplet II/I
		B3LYP/6-31+G(d,p)			
-1.84	0	279.0	194.6	218.7	217.1
-0.41	60.8	332.8	256.9	273.2	238.4
-0.10	111.0	376.0	307.5	322.1	241.8
0	122.8	388.3	324.8	355.1	252.8
0.12	134.8	398.5	339.5	368.8	246.3
0.41	151.3	410.4	357.2	379.8	202.8
0.54	152.6	411.5	218.9	375.3	191.4
		M06-2X/6-31+G(d,p)[1]			
-1.59	0	322.3	224.7	312.3	309.0
-0.41	66.6	379.6	288.7	355.3	313.3
0	137.4	441.5	364.1	386.8	288.2
0.41	173.6	479.2	425.8	412.9	218.9
0.54	174.8	482.5	435.0	411.0	222.8

[1] Structures were re-energy minimised, with fixed values of the H-transfer coordinate z for all states except the reactant state.

Table S2. Spin-orbit couplings (SOC), $\langle S_i|S_j \rangle$, calculated at the B3LYP/6-31+G(d,p) level of theory for the isolated Flavin molecule and Flavin/1a encounter complex, between singlet states *i* and triplet states *j*.

		$\langle S_i\|S_j \rangle$	
i	*j*	*Flavin*	*RF +1a*
0	*1*	*0.270*	*1.213*
0	*2*	*0.070*	*2.064*
0	*3*	*14.700*	*0.316*
0	*4*	*29.053*	*14.695*
0	*5*	*0.194*	*29.361*
1	*1*	*0.056*	*3.505*
1	*2*	*0.382*	*3.803*
1	*3*	*4.327*	*1.334*
1	*4*	*10.035*	*2.846*
1	*5*	*0.559*	*1.663*
2	*1*	*9.054*	*3.250*
2	*2*	*5.483*	*5.589*
2	*3*	*0.615*	*0.535*
2	*4*	*3.103*	*3.952*
2	*5*	*5.208*	*9.838*

References

[1] Y. SATO, H. NAKAI, T. MIZOGUCHI, M. KAWANISHI, Y. HATANAKA, Y. KANAOKA, *Chem. Pharm. Bull.* **1982**, *30*, 1263–1270.

[2] E. Alfonzo, S. M. Hande, *ACS Catal.* **2020**, *10*, 12590–12595.

[3] B. M. Trost, G. R. Dake, *J. Am. Chem. Soc.* **1997**, *119*, 7595–7596.

[4] T. Soldatović, Ž. D. Bugarčić, *J. Inorg. Biochem.* **2005**, *99*, 1472–1479.

[5] V. Hugenberg, R. Fröhlich, G. Haufe, *Org. Biomol. Chem.* **2010**, *8*, 5682–5691.

[6] H. Liu, V. R. Pattabiraman, J. C. Vederas, *Org. Lett.* **2007**, *9*, 4211–4214.

[7] D. Ulbrich, C. G. Daniliuc, G. Haufe, *J. Fluor. Chem.* **2016**, *188*, 65–75.

[8] Z. Wang, X. Yu, B.-X. Tian, D. T. Payne, W.-L. Yang, Y.-Z. Liu, J. S. Fossey, W.-P. Deng, *Chem. – A Eur. J.* **2015**, *21*, 10457–10465.

[9] L. A. Carpino, *J. Am. Chem. Soc.* **1993**, *115*, 4397–4398.

[10] S. Ray, M. G. B. Drew, A. K. Das, A. Banerjee, *Tetrahedron* **2006**, *62*, 7274–7283.

[11] S. Spisani, G. Cavicchioni, *Bioorg. Chem.* **2000**, *28*, 252–259.

[12] J. Lakowicz, *Principles of Fluorescence Spectroscopy*, **2006**.

[13] M. J. Frisch, G. W. Trucks, H. B. Schlegel, G. E. Scuseria, M. a. Robb, J. R. Cheeseman, G. Scalmani, V. Barone, G. a. Petersson, H. Nakatsuji, X. Li, M. Caricato, a. V. Marenich, J. Bloino, B. G. Janesko, R. Gomperts, B. Mennucci, H. P. Hratchian, J. V. Ortiz, a. F. Izmaylov, J. L. Sonnenberg, Williams, F. Ding, F. Lipparini, F. Egidi, J. Goings, B. Peng, A. Petrone, T. Henderson, D. Ranasinghe, V. G. Zakrzewski, J. Gao, N. Rega, G. Zheng, W. Liang, M. Hada, M. Ehara, K. Toyota, R. Fukuda, J. Hasegawa, M. Ishida, T. Nakajima, Y. Honda, O. Kitao, H. Nakai, T. Vreven, K. Throssell, J. a. Montgomery Jr., J. E. Peralta, F. Ogliaro, M. J. Bearpark, J. J. Heyd, E. N. Brothers, K. N. Kudin, V. N. Staroverov, T. a. Keith, R. Kobayashi, J. Normand, K. Raghavachari, a. P. Rendell, J. C. Burant, S. S. Iyengar, J. Tomasi, M. Cossi, J. M. Millam, M. Klene, C. Adamo, R. Cammi, J. W. Ochterski, R. L. Martin, K. Morokuma, O. Farkas, J. B. Foresman, D. J. Fox, **2016**, Gaussian 16, Revision C.01, Gaussian, Inc., Wallin.

[14] S. H. Vosko, L. Wilk, M. Nusair, *Can. J. Phys.* **1980**, *58*, 1200–1211.

[15] C. Lee, W. Yang, R. G. Parr, *Phys. Rev. B* **1988**, *37*, 785–789.

[16] A. D. Becke, *Phys. Rev. A* **1988**, *38*, 3098–3100.

[17] S. Grimme, S. Ehrlich, L. Goerigk, *J. Comput. Chem.* **2011**, *32*, 1456–1465.

[18] S. Grimme, J. Antony, S. Ehrlich, H. Krieg, *J. Chem. Phys.* **2010**, *132*, 154104.

[19] R. Improta, V. Barone, G. Scalmani, M. J. Frisch, *J. Chem. Phys.* **2006**, *125*, 54103.

[20] W. Ying, V. Pragya, J. Xinsheng, T. D. G., H. Xiao, *Proc. Natl. Acad. Sci.* **2018**, *115*, 10257–10262.

[21] A. S. Tiwary, A. K. Mukherjee, *Chem. Phys. Lett.* **2014**, *610–611*, 19–22.

[22] B. K. Ong, K. L. Woon, A. Ariffin, *Synth. Met.* **2014**, *195*, 54–60.

[23] D. Jacquemin, I. Duchemin, A. Blondel, X. Blase, *J. Chem. Theory Comput.* **2017**, *13*, 767–783.

[24] D. Jacquemin, E. A. Perpète, I. Ciofini, C. Adamo, *J. Chem. Theory Comput.* **2010**, *6*, 1532–1537.

[25] X. Gao, S. Bai, D. Fazzi, T. Niehaus, M. Barbatti, W. Thiel, *J. Chem. Theory Comput.* **2017**, *13*, 515–524.
